# BPLLDA: Predicting lncRNA-Disease Associations Based on Simple Paths With Limited Lengths in a Heterogeneous Network

**DOI:** 10.3389/fgene.2018.00411

**Published:** 2018-10-16

**Authors:** Xiaofang Xiao, Wen Zhu, Bo Liao, Junlin Xu, Changlong Gu, Binbin Ji, Yuhua Yao, Lihong Peng, Jialiang Yang

**Affiliations:** ^1^College of Information Science and Engineering, Hunan University, Changsha, China; ^2^School of Mathematics and Statistics, Hainan Normal University, Haikou, China; ^3^School of Computer Science, Hunan University of Technology, Zhuzhou, China; ^4^Icahn Institute for Genomics and Multiscale Biology, Icahn School of Medicine at Mount Sinai, New York, NY, United States

**Keywords:** disease similarity, lncRNA similarity, path with limited length, Gaussian interaction profile kernel similarity, leave-one-out cross validation, ROC curve

## Abstract

In recent years, it has been increasingly clear that long noncoding RNAs (lncRNAs) play critical roles in many biological processes associated with human diseases. Inferring potential lncRNA-disease associations is essential to reveal the secrets behind diseases, develop novel drugs, and optimize personalized treatments. However, biological experiments to validate lncRNA-disease associations are very time-consuming and costly. Thus, it is critical to develop effective computational models. In this study, we have proposed a method called BPLLDA to predict lncRNA-disease associations based on paths of fixed lengths in a heterogeneous lncRNA-disease association network. Specifically, BPLLDA first constructs a heterogeneous lncRNA-disease network by integrating the lncRNA-disease association network, the lncRNA functional similarity network, and the disease semantic similarity network. It then infers the probability of an lncRNA-disease association based on paths connecting them and their lengths in the network. Compared to existing methods, BPLLDA has a few advantages, including not demanding negative samples and the ability to predict associations related to novel lncRNAs or novel diseases. BPLLDA was applied to a canonical lncRNA-disease association database called LncRNADisease, together with two popular methods LRLSLDA and GrwLDA. The leave-one-out cross-validation areas under the receiver operating characteristic curve of BPLLDA are 0.87117, 0.82403, and 0.78528, respectively, for predicting overall associations, associations related to novel lncRNAs, and associations related to novel diseases, higher than those of the two compared methods. In addition, cervical cancer, glioma, and non-small-cell lung cancer were selected as case studies, for which the predicted top five lncRNA-disease associations were verified by recently published literature. In summary, BPLLDA exhibits good performances in predicting novel lncRNA-disease associations and associations related to novel lncRNAs and diseases. It may contribute to the understanding of lncRNA-associated diseases like certain cancers.

## Introduction

It is known that there are about 20,000 protein-coding genes, consisting of less than 2% of the human genome (Bertone et al., 2004; Claverie, 2005). Most DNA regions in the human genome are either not transcribable or transcribed into noncoding RNAs (ncRNAs), which are deemed to be transcriptional noises in a long period of time. However, many recent studies have suggested that ncRNAs play key regulatory roles in many important biological processes such as cell proliferation (Esteller, 2011). Based on their sizes, ncRNAs can be divided into long ncRNAs (lncRNAs) (Pauli et al., 2011) and small ncRNAs such as microRNAs (miRNAs) (Farazi et al., 2013), transfer RNAs (tRNAs) (Birney et al., 2007), and Piwi-interacting RNAs (piRNAs) (Li et al., 2013). LncRNAs are ncRNAs of lengths greater than 200 nucleotides (Mercer et al., 2009; Mitchell Guttman et al., 2013). Compared to protein-coding, RNAs, lncRNAs are less conservative among species (Harrow et al., 2012; Cabili et al., 2016), and have a relatively low expression level, more tissue-specific patterns (Guttman et al., 2010), and longer but less exons (Chen, 2015). Recently, more and more lncRNAs have been identified in eukaryotes from nematodes to human beings due to the advancement in sequencing technologies and computational methods (Awan et al., 2017).

Previous studies have suggested that lncRNAs are critical in cell proliferation, cell differentiation, chromatin remodeling, genome splicing, epigenetic regulation, transcription, and many other important biological processes (Guttman et al., 2009). The dysregulation of lncRNAs has also been associated with the development of many diseases, including diabetes (Pasmant et al., 2011), cardiovascular diseases (Congrains et al., 2012), HIV (Zhang et al., 2013), neurological disorders (Johnson, 2012), and several cancers such as lung cancer (Ji et al., 2003; Zhang et al., 2003), breast cancer (Barsyte-Lovejoy et al., 2006; Gupta et al., 2010), and prostate cancer (Kok et al., 2002; Szell et al., 2008). As a result, it has become a hot topic recently to identify lncRNA-disease associations, and many important disease-associated lncRNAs have been discovered. For example, breast cancer metastasis patients have about 100 to 2,000 times higher *HOTAIR* expression than that of the healthy people, based on a quantitative PCR study (Gupta et al., 2010). *HOTAIR* is also related to metastasis and progression of other cancers, such as liver cancer (Hrdlickova et al., 2014), lung cancer (Li et al., 2014), colorectal cancer (Res, 2011; Maass et al., 2014), gastric cancer (Li et al., 2014; Liu et al., 2014), and so on. Therefore, HOTAIR is deemed to be a potential biomarker for cancers (Maass et al., 2014). In addition, the dysfunction of lncRNA *H19* is found in several diseases, such as bladder cancer (Ariel et al., 2000). The downregulation of *H19* also significantly reduces the clonogenic and anchored nondependent growth of breast cancer cells based on a knock-down study (Barsyte-Lovejoy et al., 2006).

Known lncRNA-disease associations have been stored in a few databases, including LncRNADisease (Chen et al., 2013), Lnc2Cancer (Ning et al., 2016), MNDR (Wang et al., 2013), and so on, which are the basis for predicting novel associations using efficient computational methods. The computational models to predict lncRNA-disease associations are generally divided into two categories including machine learning-based models and network-based models (Chen et al., 2017). Machine learning-based models usually train predictors from features based on training samples and test their performances based on cross-validation or independent data. For example, Chen et al. developed Laplacian Regularized Least Squares for LncRNA-Disease Association (LRLSLDA) for inferring candidates of disease-associated lncRNAs by applying a semisupervised learning framework (Chen and Yan, 2013). LRLSLDA assumes that similar diseases tend to correlate with functionally similar lncRNAs, and vice versa. Thus, known lncRNA-disease associations and lncRNA expression profiles are combined to prioritize disease-associated lncRNA candidates by LRLSLDA, which does not require negative samples (i.e., confirmed uncorrelated lncRNA-disease associations). However, LRLSLDA faces difficulty in optimizing the best model parameters. Zhao T. et al. (2015) proposed a naïve Bayesian classifier, which exploits various information related to cancer-associated lncRNAs, including regulome, genome, transcriptome, and multiomic data. As a result, 707 potential cancer-related lncRNAs were identified. However, this method requires negative samples, which are usually unknown. In contrast, network-based methods take the advantage of the lncRNA-disease association network, the disease similarity network, and the lncRNA similarity network to study the connectivity of lncRNAs and diseases. For instance, Sun et al. (2014) developed RWRlncD, which infers potential lncRNA-disease associations by a random walk with restart (RWR) on the lncRNA functional similarity network. However, the method cannot predict lncRNAs related to novel diseases (i.e., diseases with no known associated lncRNA). Gu et al. (2017) provided a global network random walk model for predicting lncRNA-disease associations (GrwLDA), which performs RWR on both lncRNA functional similarity network and disease similarity network. However, GrwLDA also faces a dilemma in optimizing model parameters.

In this study, we have proposed a novel method BPLLDA to predict lncRNA-disease associations based on paths connecting them with limited lengths in a heterogeneous network. Specifically, BPLLDA first establishes a heterogeneous network consisting of the known lncRNA-disease association network, the disease similarity network, and the lncRNA similarity network. It then calculates the association between a disease and an lncRNA by the paths connecting them and their lengths. BPLLDA does not require negative samples and is capable of predicting novel diseases and novel lncRNAs.

## Materials and methods

### lncRNA-disease associations

The lncRNA-disease association data were retrieved from the database LncRNADisease (Chen et al., 2013; Sun et al., 2014). After eliminating identical lncRNA-disease entries from distinct pieces of evidence, there were 352 experimentally confirmed lncRNA-disease associations, containing 156 lncRNAs and 190 diseases (see Supplementary Figure 1 and Supplementary Tables 2, 3). We summarize some basic characteristics (e.g., the average degree) of the dataset in Table 1. We then established the lncRNA-disease association network, whose adjacency matrix is denoted by LD. That is, *LD*(*i, j*) is set to 1 if lncRNA *l*(*i*) is associated with disease *d*(*j*), and 0 if otherwise. Before presenting the details of BPLLDA, we first introduced two important notations, namely, disease semantic similarity and lncRNA functional similarity.

**Table 1:** The basic characteristics of the lncRNA-disease association dataset.

**Total of lncRNAs**	**Total of diseases**	**Total of associations**	**Average degree of lncRNAs**	**Average degree of diseases**	**Max degree of lncRNAs**	**Max degree of diseases**	**Min degree of lncRNAs/diseases**
156	190	352	2.3	1.9	41	15	1

### Disease semantic similarity

**Figure 1:**
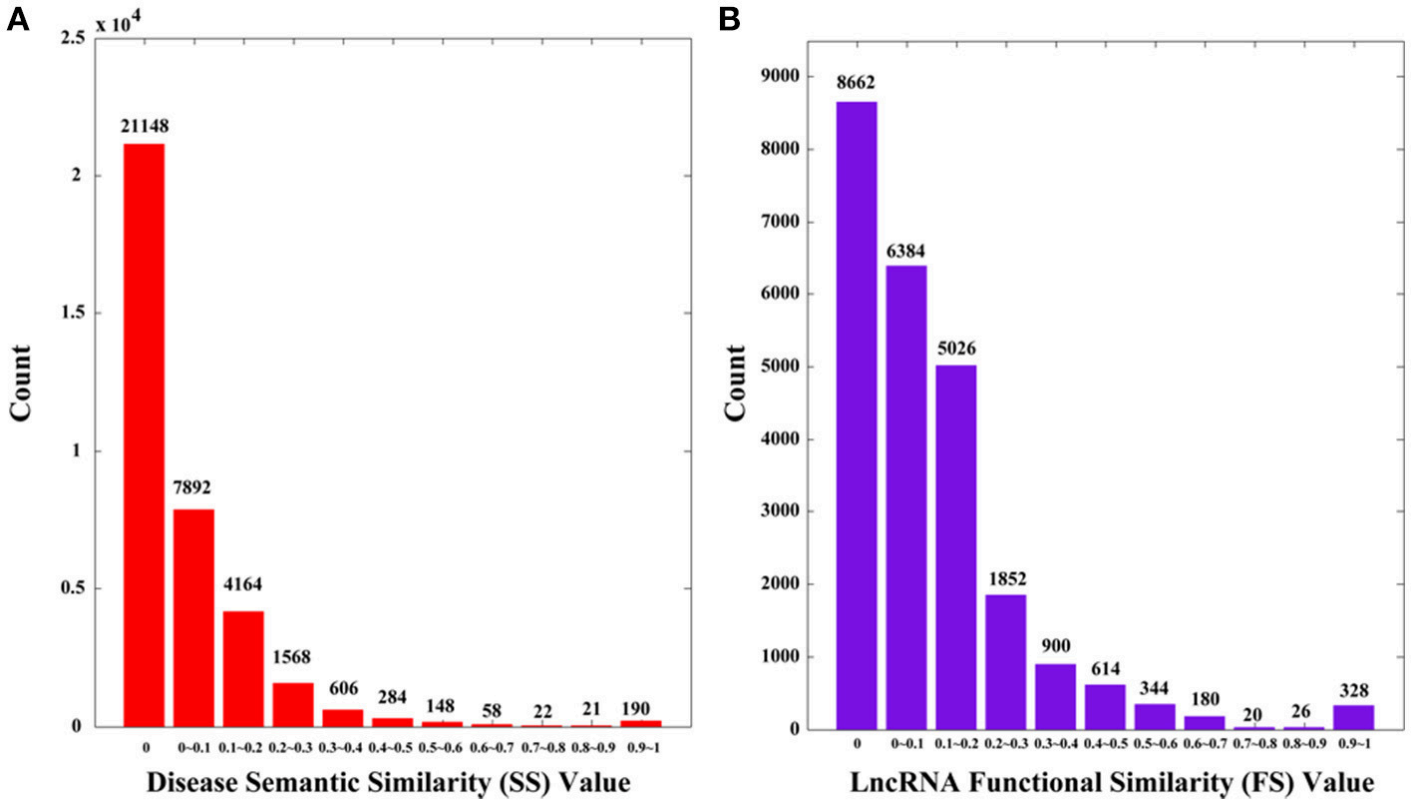
The distributions of disease semantic and lncRNA functional similarity. **(A)** Disease semantic similarity (SS) distribution. **(B)** lncRNA functional similarity (FS) distribution. The *x*-axis indicates the intervals of similarity values and the *y*-axis indicates the numbers of values in the interval. The actual values are also marked above the histograms.

The Disease Ontology (DO) is an open source ontology of human diseases (http://www.disease-ontology.org/). The terms in DO are diseases or disease-correlated concepts, which are organized in a directed acyclic graph (DAG). On the basis of Disease Ontology, Li et al. (2011) provided an R package called DOSim to calculate the disease semantic similarity, and we adopted this method in this study. Specifically, we used a symmetric matrix SS to record semantic similarity values among diseases, in which *SS*(*i, j*) represents semantic similarity between disease *d*(*i*) and *d*(*j*) as calculated by DOSim. We plot the distribution of *SS* in Figure 1A. There are overall 36100 (190 × 190) values, among which 21148 values (58.58%) are 0 s.

### lncRNA functional similarity

We adopted a similar method to Sun et al. for measuring the functional similarity between two lncRNAs (Wang et al., 2010; Sun et al., 2014). Specifically, suppose lncRNA *l*(*i*) is associated with a disease set *D*_*i*_ = {*d*_*ik*_| 1 ≤ *k* ≤ *m*} and lncRNA *l*(*j*) is associated with *D*_*j*_ = {*d*_*jl*_| 1 ≤ *l* ≤ *n*}. The method first calculates the semantic similarity between a disease, say *d*_*i*1_, and a disease group, say *D*_*j*_, as

$$\begin{array}{l} \text{SIM}\left(d_{i1},D_{j}\right)=(SS(d_{i1},d)). \end{array}$$

Then, the functional similarity between *l*(*i*) and *l*(*j*) is calculated as

$$\begin{array}{l} FS\left(l\left(i\right),l\left(j\right)\right)=\;\frac{\sum_{1\leq k\leq m}\text{SIM}\left(d_{ik},D_{j}\right)+\sum_{1\leq l\leq n}\text{SIM}\left(d_{jl},D_{i}\right)}{m+n}. \end{array}$$

It is clear that the lncRNA functional similarity matrix *FS* is symmetric. Similarly, we plot the distribution of *FS* in Figure 1B. There are 24336 (156 × 156) values, among which 8662 (35.59%) are 0 s.

### Gaussian interaction profile kernel similarity for lncRNAs

There are many zeros in *FS* due to the fact that lncRNA-disease associations are rather incomplete. To avoid such scenario, we introduced the Gaussian interaction profile kernel similarity between lncRNA *l*(*i*) and *l*(*i*) as

$$\begin{array}{l} \text{GL}\left(l\left(i\right),l\left(j\right)\right)=\text{exp}\left(-\gamma_{l}||IP\left(l\left(i\right)\right)-IP\left(l\left(j\right)\right)|{|}^{2}\right), \end{array}$$

where *IP*(*l*(*i*)) and *IP*(*l*(*j*)) are the vectors in the *i*th and *j*th row of the lncRNA-disease association matrix *LD*. The parameter γ_*l*_ is a regulation parameter of the kernel bandwidth with $\gamma_{l}=\;{\gamma^{\prime }}_{l}/\left(\frac{1}{\ln}\overset{\ln}{\underset{i=1}{\sum }}||IP\left(l\left(i\right)\right)|{|}^{2}\right)$, where *ln* is the number of all lncRNAs studied and ${\gamma^{\prime }}_{l}$ is usually set to 1 according to van Laarhoven et al. (2011).

### Gaussian interaction profile kernel similarity for diseases

Similarly, we defined the Gaussian interaction profile kernel similarity for diseases as

$$\begin{array}{l} \text{GD}\left(d\left(i\right),d\left(j\right)\right)=\text{exp}\left(-\gamma_{d}||IP\left(d\left(i\right)\right)-IP\left(d\left(j\right)\right)|{|}^{2}\right) \end{array}$$

with $\gamma_{\text{d}}=\;{\gamma^{\prime }}_{\text{d}}/\left(\frac{1}{\text{dn}}\overset{dn}{\underset{\text{i}=1}{\sum }}||\text{IP}\left(\text{d}\left(\text{i}\right)\right)|{|}^{2}\right)$, where IP(*d*(*i*)) and *IP*(*d*(*i*)) are the binary vectors in the *i*th and *j*th column of the adjacency matrix *LD* and *dn* is the numbers of diseases. Clearly, *GD* is also symmetric.

### Integrated similarity between lncRNAs and between diseases

**Figure 2:**
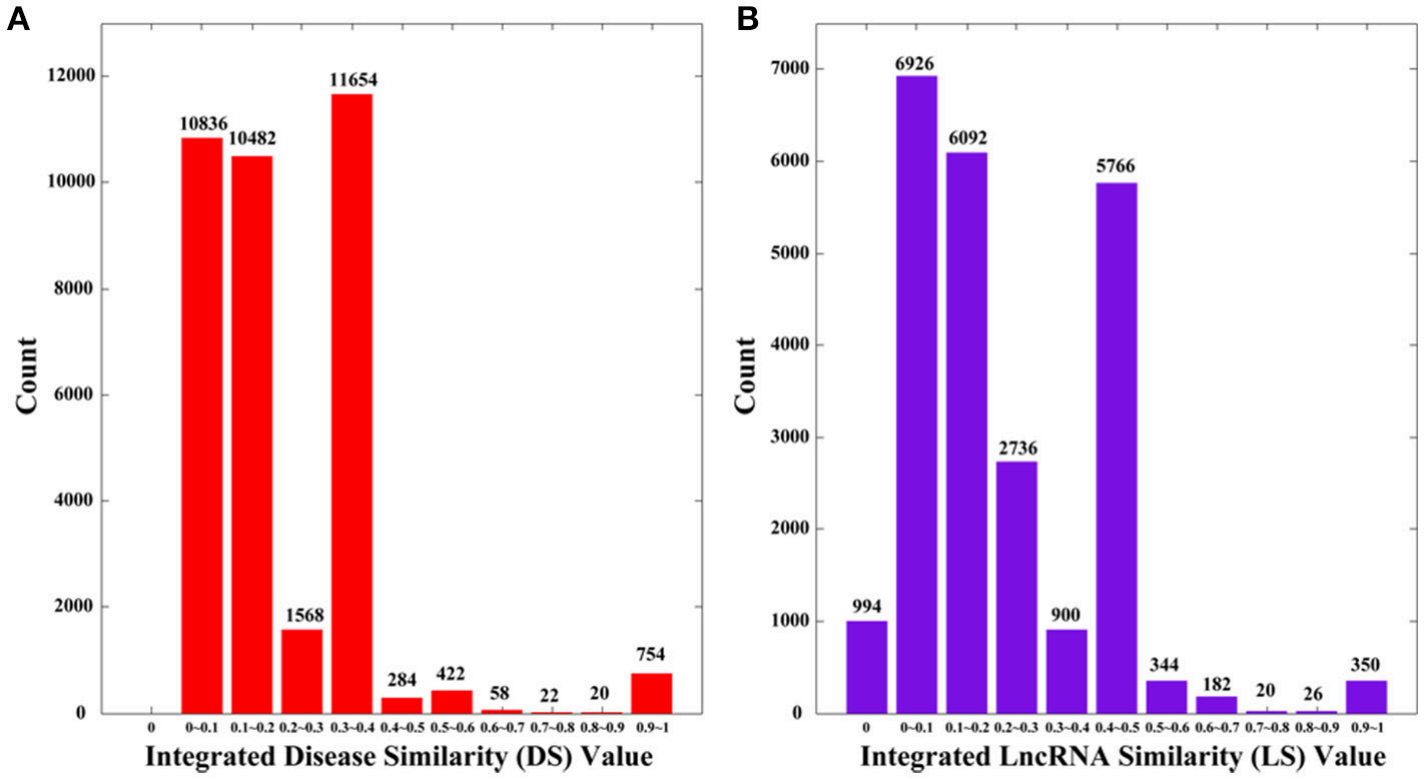
The distributions of integrated similarities. **(A)** Distribution of the integrated similarity for diseases (DS). **(B)** Distribution of the integrated similarity for lncRNAs (LS). The *x*-axis indicates the intervals of similarity values and the *y*-axis indicates the numbers of values in the interval. The actual values are also marked above the histograms.

We integrated disease semantic similarity (lncRNA functional similarity) with the Gaussian interaction profile kernel similarity for diseases (lncRNAs) as follows:

$$\begin{array}{l} DS\left(d\left(i\right),d\left(j\right)\right)\\ =\;\{\begin{array}{l} GD\left(d\left(i\right),d\left(j\right)\right)\;if\;d(i)\in NS\;or\;d(j)\in NS\\ SS\left(d\left(i\right),d\left(j\right)\right)\text{ otherwise} \end{array}\\ LS\left(l\left(i\right),l\left(j\right)\right)\\ =\;\{\begin{array}{l} GL\left(l\left(i\right),l\left(j\right)\right)\;if\;l(i)\in NF\;or\;l(j)\in NF\\ FS\left(l\left(i\right),l\left(j\right)\right)\text{ otherwise} \end{array} \end{array}$$

where NS is the set of diseases with no sematic similarity with any other disease, and NF is the set of lncRNAs with no functional similarity with any other lncRNAs. By definition, *DS* and *LS* are symmetric. We plot the distributions of *DS* and *LS* in Figure 2, in which the numbers of 0 s are greatly reduced compared to *SS* and *FS*.

### BPLLDA

**Figure 3:**
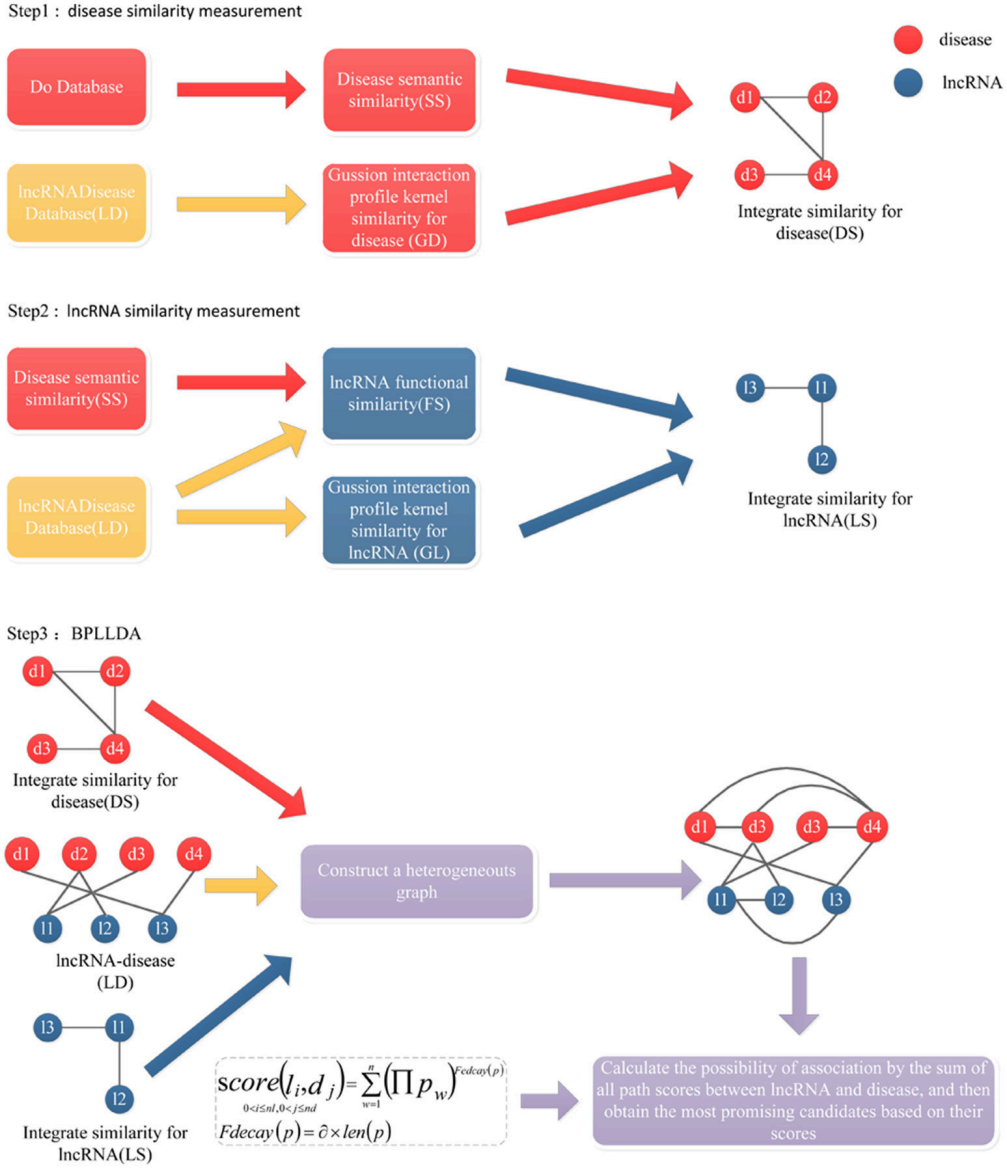
The flowchart of BPLLDA. It consists of three steps: (1) disease similarity measurement, (2) lncRNA similarity measurement, and (3) the BPLLDA algorithm.

The general workflow of BPLLDA is illustrated in Figure 3, in which a heterogeneous network is first constructed with nodes denoting lncRNAs or diseases. For any two diseases *d*(*i*) and *d*(*j*), the weight of the edge between them is defined to be

$$\begin{array}{l} WD\left(d\left(i\right),d\left(j\right)\right)\\ =\{\begin{array}{l} \;0\;if\;DS\left(d\left(i\right),d\left(j\right)\right)\;<T\\ DS\left(d\left(i\right),d\left(j\right)\right)\;otherwise \end{array}\;, \end{array}$$

where *T* is a threshold value to avoid all diseases being connected (You et al., 2017). Similarly, the weight of the edge between two lncRNAs *l*(*i*) and *l*(*j*) is

$$WL\left(l\left(i\right),l\left(j\right)\right)=\{\begin{array}{l} 0\;if\;LS\left(l\left(i\right),l\left(j\right)\right)\;<T\\ LS\left(l\left(i\right),l\left(j\right)\right)\;otherwise \end{array}\;.$$

The weight of an edge between an lncRNA *l*(*i*) and a disease *d*(*j*) is *LD*(*l*(*i*), *d*(*j*)), that is, the weight is 1 if they are associated and 0 if otherwise. We tuned *T* from 0.1 to 0.5 with interval 0.1 by a leave-one-out cross-validation (LOOCV) process and finally chose *T* to be 0.2.

For a given lncRNA node *l*(*i*) and a disease node *d*(*j*), we performed a depth-first search (Hopcroft and Tarjan, 1974) to identify all noncyclic paths between them. To avoid long paths, we restricted the maximum number of edges in the path to be τ. Similarly, we performed an LOOCV search for τ being 1 to 4 and decided τ to be 3. Intuitively, *l*(*i*) and *d*(*j*) tend to be associated if there are many paths with high edge weights connecting them. Therefore, a score measuring their association confidence can be defined using the paths together with a decay function *F*_decay_(*p*_*w*_):

$$\text{score}(l(i),d(j))=\;\sum_{w=1}^{n}{\left(\prod p_{w}\right)}^{F_{\text{decay}}\left(p_{w}\right)}$$

where *p* = {*p*_1_, *p*_2_, …, *p*_*n*_} is the set of paths connecting *l*(*i*) and *d*(*j*), and ∏*p*_*w*_ denotes the product of the weights of all edges in the path *p*_*w*_. Generally speaking, long paths will have little contribution to the total score. So the decay function *F*_decay_(*p*) is denoted as

$$\begin{array}{l} F_{\text{decay}}\left(p_{w}\right)=\;\alpha \times \text{len}\left(p_{w}\right), \end{array}$$

where the decay factor α is set to 2.26 based on a previous study (Ba-Alawi et al., 2016; You et al., 2017) and *len*(*p*_*w*_) is the length of the path *p*_*w*_. Clearly, the higher the *score*(*l*(*i*), *d*(*j*)), the more likely that *l*(*i*) and *d*(*j*) will be associated.

### Analysis of the computational complexity

We analyzed the time complexity and space complexity of BPLLDA. Recall that there are *m* diseases and *n* lncRNAs with *m* > *n*. The algorithm mainly consists of two steps. First, a heterogeneous network was constructed, for which two matrices were established. So the time complexity and space complexity are O(*m*^2^) respectively in this step. Then, BPLLDA infers the probability of an lncRNA-disease association based on paths with limited lengths in the network. We performed a depth-first search to identify all noncyclic paths between nodes and the time complexity is O((*m* + *n*)^2^) on each node. Because there are *m* diseases, the time complexity is O(*m*^3^) in this step. And the space complexity is O(*mn*) because we need to only save the prediction result. In summary, the time complexity and space complexity are at most O(*m*^3^) and O(*m*^2^), respectively, for BPLLDA.

## Results and discussions

### Performance of BPLLDA in predicting lncRNA-disease associations

We applied BPLLDA to a known lncRNA-disease association data LD, together with two popular methods GrwLDA (Gu et al., 2017) and LRLSLDA (Chen and Yan, 2013). The reason why we selected the two methods for comparison is that they can both predict novel lncRNAs and novel diseases. Specifically, two LOOCV methods namely global LOOCV and local LOOCV were adopted to evaluate their performances. Global LOOCV sets each experimentally confirmed lncRNA-disease association as a test sample once, but local LOOCV sets all associations of an lncRNA or those of a disease as test samples once. Other known lncRNA-disease associations are considered as training samples. The performances of the methods were evaluated by the area under the receiver operating characteristic (ROC) curve (AUC).

**Figure 4:**
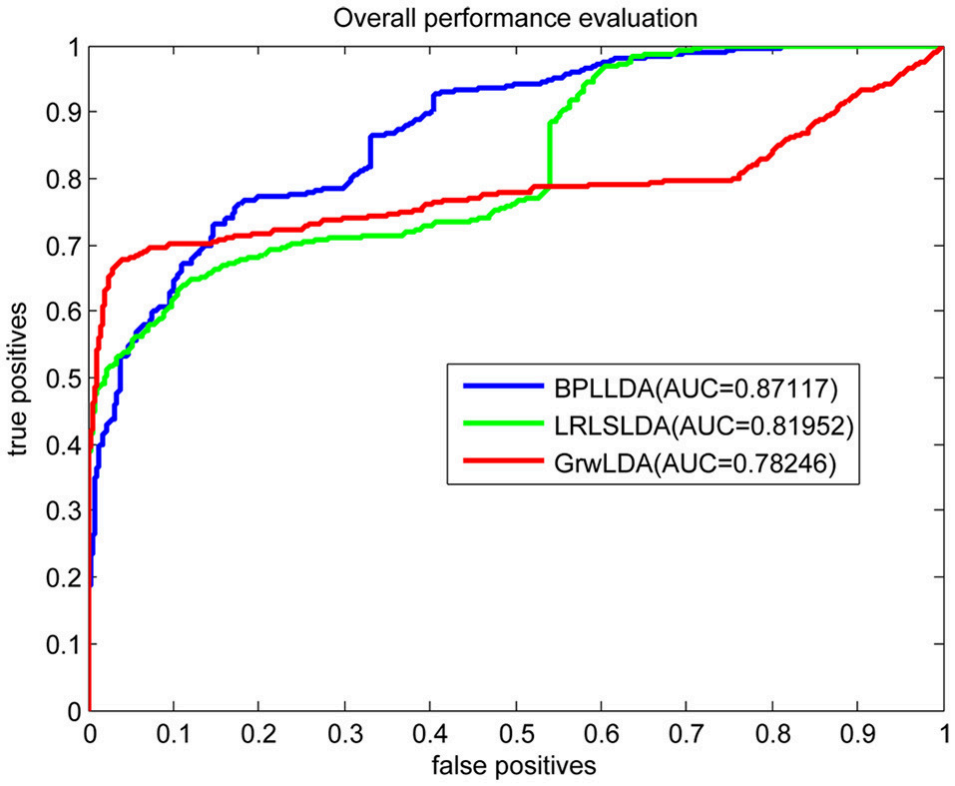
Performance evaluation of BPLLDA, LRLSLDA, and GrwLDA in predicting lncRNA-disease associations by global LOOCV.

**Figure 5:**
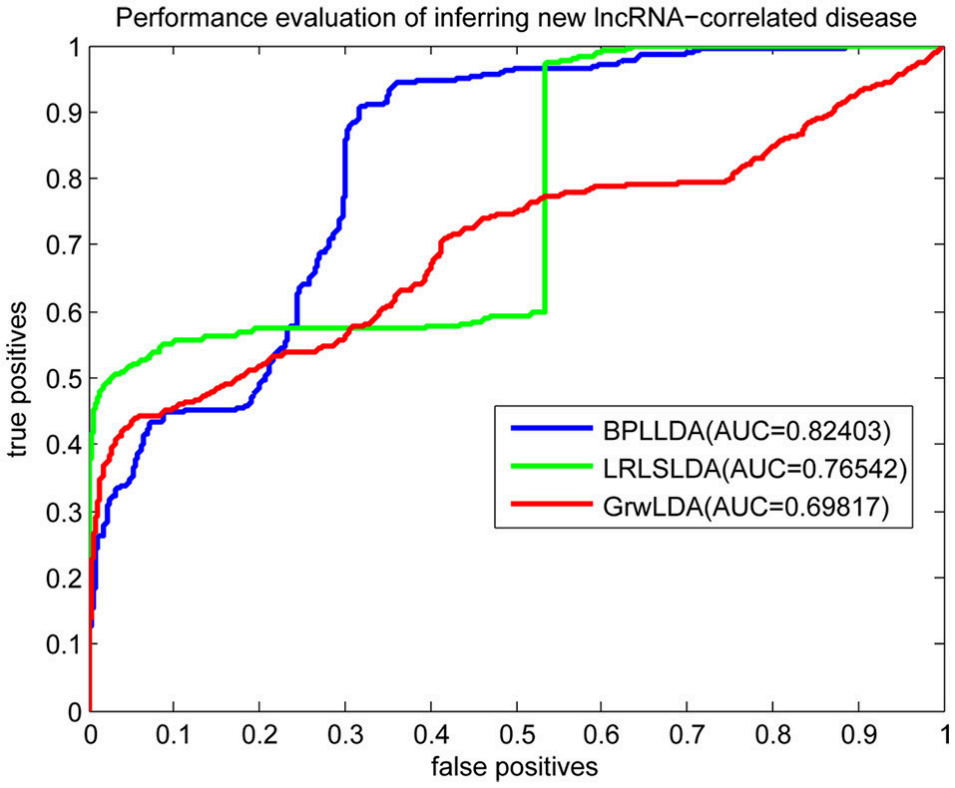
Performance evaluation of BPLLDA, LRLSLDA, and GrwLDA in predicting novel lncRNA-associated diseases.

**Figure 6:**
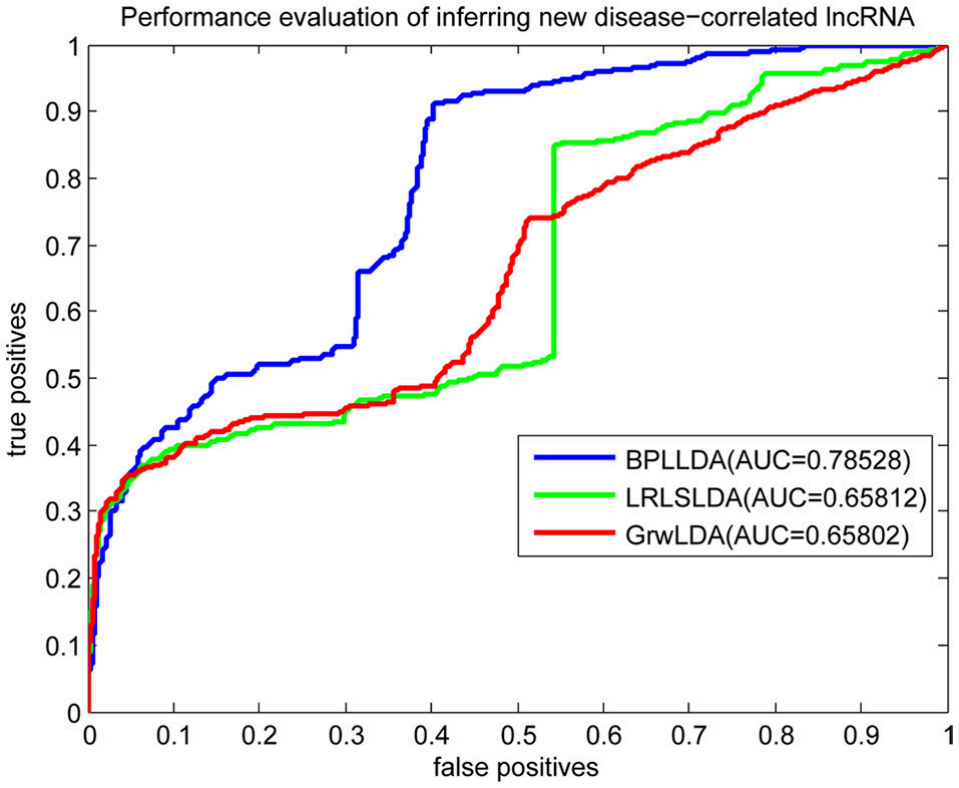
Performance evaluation of BPLLDA, LRLSLDA, and GrwLDA in predicting novel disease-associated lncRNAs.

As a result, we plotted the global LOOCV ROC curves and their associated AUCs of BPLLDA, GrwLDA, and LRLSLDA, respectively, in Figure 4. BPLLDA has an AUC of 0.87117, and outperformed LRLSLDA (0.81952) and GrwLDA (0.78246). Similarly, we plotted the local LOOCV ROC curves and AUCs of the three methods on novel lncRNAs in Figure 5. As can be seen, BPLLDA has an AUC of 0.82403, about 8 and 18% higher than that of LRLSLDA (0.76542) and GrwLDA (0.69817), respectively. Finally, the AUC of BPLLDA (0.78528) in predicting novel diseases is significantly higher than that of LRLSLDA (0.65812) with an increase of 19% and GrwLDA (0.65802) with an increase of 20% (see Figure 6). In summary, our method is better than LRLSLDA and GrwLDA in both lncRNA-disease association prediction and prediction related to novel lnRNAs and diseases.

Meanwhile, we list in Table 2 the precision versus the prediction scores in the global LOOCV. In general, the higher the score, the more likely the disease is related to the lncRNAs. The association confidence is greater than 0.9 when the prediction score is larger than 21.58.

**Table 2:** Precision of BPLLDA on global LOOCV.

**Prediction scores**	**1.002~9.929**	**10.028~17.601**	**21.580~24.391**	**25.778~37.757**
Precision	> = 0.134	> = 0.446	> = 0.933	1

### Effects of parameters

There are two model parameters in BPLLDA, including the maximum path length *L* and the weight threshold *T*. We tested the effects of these parameters on AUCs for LOOCV with *L* (*L* = 2, 3, 4) and *T* (*T* = 0.2, 0.4, 0.5), and we list the results in Table 3. As can be seen, the parameter *L* has significant effects on the performance of BPLLDA, and the best AUC is achieved at *L* = 3. In contrast, *T* has only minor effects on the performance of our method. To further illustrate this, we fixed *L* to be 3, and let *T* vary from 0.1 to 0.5 with interval 0.1 (see Table 4). The AUC values are between 0.85568 and 0.87117, only about 2% difference.

**Table 3:** Tuning two model parameters: the maximum path length L and the weight threshold T by LOOCV.

**L**	**2**	**3**	**4**
T = 0.2	0.83903	0.87117	*
T = 0.4	0.82043	0.85568	0.81205
T = 0.5	0.81761	0.85959	0.80830

*The value in each cell represents LOOCV AUC*.

* *T = 0.2 and L = 4 was not calculated because it takes more than 48 h*.

**Table 4:** The effects of T on AUC when fixing *L* = 3.

**T**	**0.1**	**0.2**	**0.3**	**0.4**	**0.5**
AUC	0.87102	0.87117	0.86889	0.85568	0.85959

### Effects of Gaussian interaction profile kernel similarity for lncRNAs and diseases

Disease similarity and lncRNA similarity are calculated by integrating disease semantic similarity, lncRNA functional similarity, as well as the Gaussian interaction profile kernel similarity for lncRNAs and diseases. We tested the effects of the Gaussian interaction profile kernel similarity for lncRNAs and diseases on LOOCV with *L* = 3 and *T* = 0.2 *with four* settings: (1) without using both the Gaussian interaction profile kernel similarity for lncRNAs and diseases; (2) only using the Gaussian interaction profile kernel similarity for lncRNAs; (3) only using the Gaussian interaction profile kernel similarity for diseases; (4) using both the Gaussian interaction profile kernel similarity for lncRNAs and diseases. The results are summarized in Table 5. As can be seen, the two similarities indeed have a significant influence on the LOOCV AUC. The best AUC (0.87117) was achieved when both similarities were adopted into our model.

**Table 5:** The effects of the Gaussian interaction profile kernel similarity for lncRNAs and diseases on LOOCV.

**No GD and GL**	**GL**	**GD**	**GL and GD**
0.78718	0.79036	0.80924	0.87117

*The value in each cell represents LOOCV AUC*.

### Case studies on predicted lncRNA-disease associations

**Figure 7:**
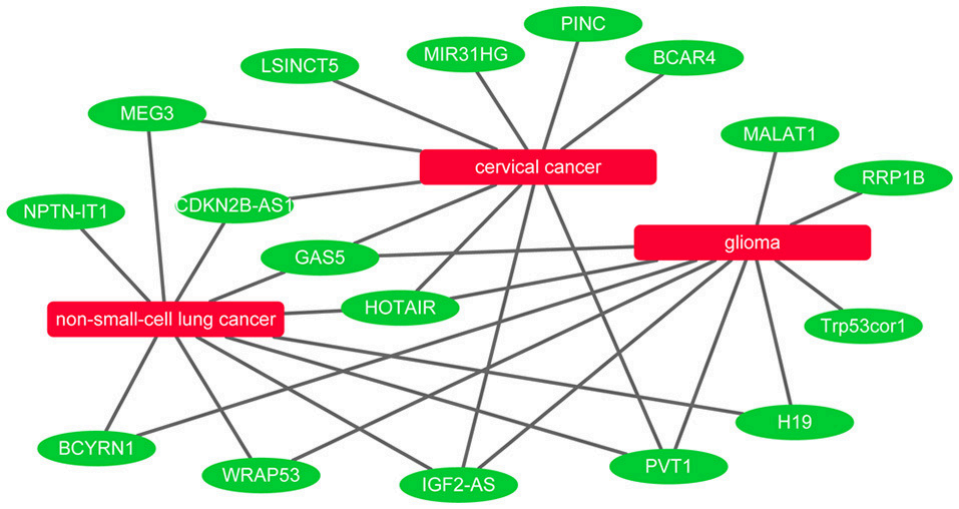
Network view of the top 10 predicted lncRNAs for cervical cancer, glioma, and non-small-cell lung cancer.

It is known that lncRNAs play critical roles in the development of many diseases. To further evaluate the ability of BPLLDA in inferring novel lncRNA-disease associations, we used all known lncRNA-disease associations in LD as training data and assessed the potential of predicted associations by our model. The novel lncRNA-disease associations were ranked according to the predicted score of BPLLDA. To validate the predictions, the newest LncRNADisease database was used, which curated 1766 distinct known lncRNA-disease associations among 888 lncRNAs and 328 diseases. Specifically, we listed the top five lncRNAs associated with three diseases, including cervical cancer, glioma, and non-small-cell lung cancer (NSCLC), respectively, in Table 6 and the paths of cervical cancer in Supplementary Table 1. For a better view, we also plotted the associations of the three diseases and their top 10 predicted lncRNAs in Figure 7.

**Table 6:** The top five lncRNA candidates predicted for cervical cancer, glioma, and non-small-cell lung cancer.

**Disease**	**lncRNA**	**Evidence**
Cervical cancer	MEG3	LncRNADisease (Zhang J. et al., 2016)
Cervical cancer	PVT1	LncRNADisease (Yang et al., 2016)
Cervical cancer	CDKN2B-AS1	LncRNADisease (Zhang D. et al., 2016)
Cervical cancer	HOTAIR	LncRNADisease (Huang et al., 2014)
Cervical cancer	GAS5	LncRNADisease (Cao et al., 2014)
Glioma	H19	LncRNADisease (Shi et al., 2014)
Glioma	MALAT1	LncRNADisease (Ma et al., 2015)
Glioma	PVT1	(Zou et al., 2017)
Glioma	HOTAIR	LncRNADisease (Ke et al., 2015)
Glioma	GAS5	LncRNADisease (Zhao X. et al., 2015)
Non-small-cell lung cancer	H19	LncRNADisease (Zhang E. et al., 2016)
Non-small-cell lung cancer	MEG3	LncRNADisease (Lu et al., 2013)
Non-small-cell lung cancer	HOTAIR	LncRNADisease (Liu X. H. et al., 2013)
Non-small-cell lung cancer	PVT1	LncRNADisease (Yang et al., 2014)
Non-small-cell lung cancer	CDKN2B-AS1	LncRNADisease (Nie et al., 2015)

Cervical cancer is a cancer in the cervix and its early symptoms are hard to uncover. As the second common cancer among women all over the world, cervical cancer causes numerous incidents of death in developing countries (Forouzanfar et al., 2011). It was reported that there are approximately 500,000 novel cases of cervical cancer diagnosed annually (Tewari et al., 2014). Therefore, there is an urgent need to explore its biological mechanisms and develop effective treatment strategies. Interestingly, all of the top five novel cervical cancer-associated lncRNAs predicted by BPLLDA were confirmed by the newest updates of the LncRNADisease database. For example, the top predicted lncRNA, *MEG3*, can inhibit tumor growth in cervical cancer by regulating miR-21-5p, which is regarded as a tumor suppressor (Zhang J. et al., 2016). Serum *PVT1* can accurately differentiate patients with cervical cancer from healthy controls (Yang et al., 2016). The high expression of *HOTAIR* is involved in cervical cancer progression and may be a potential target for diagnosis and gene therapy (Huang et al., 2014).

Glioma is considered to be the most common malignant tumor in the central nervous system and is characterized by aggressive blood vessel formation (Khasraw et al., 2010). Despite the continuous improvement of various treatments, including surgery, radiotherapy, and chemotherapy, the overall survival of patients with glioma is only about 12–14 months after diagnosis (Wang et al., 2015). The poor treatment effect is mainly due to the prominent tumor angiogenesis. Similarly, BPLLDA achieved good performance in predicting glioma-associated lncRNAs as all top five predicted lncRNAs were confirmed by the newest LncRNADisease database and literature. For example, it was shown that *H19* regulates the development of glioma by deriving miR-675 and offers an essential clue to understanding the key role of the lncRNA-miRNA functional network in glioma (Shi et al., 2014). The expression level of lncRNA *MALAT1* is significantly correlated with the overall survival of patients with glioma and can be used as a convictive prognostic biomarker for patients with glioma (Ma et al., 2015). In addition, *Gas5* inhibits tumor malignancy by downregulating miR-222, which may be a promising treatment for glioma (Zhao X. et al., 2015).

NSCLC, including adenocarcinoma and squamous cell carcinoma, is a predominant form of lung cancer (Siegel et al., 2012). Despite the progress in clinical and experimental oncology, the prognosis remains difficult. More and more evidence indicates that ncRNAs could take part in the pathogenesis of NSCLC. Similarly, the top five NSCLC-correlated lncRNA candidates predicted by BPLLDA were validated by literature. For example, *HOTAIR* is significantly upregulated in NSCLC tissues and partly regulates cell invasion and metastasis of NSCLC by *HOXA5* downregulation (Liu X. H. et al., 2013). So, *HOTAIR* is a potential therapeutic target for NSCLC intervention. In addition, patients with NSCLC with high *PVT1* expression have a significantly lower overall survival rate than those with low *PVT1* expression (Yang et al., 2014). Finally, the expression of *CDKN2B-AS1* (*ANRIL*) might damage cell proliferation and leads to cell apoptosis *in vitro* and *in vivo* (Nie et al., 2015), which is linked to the survival of patients with NSCLC.

### Case studies on predicted novel diseases and novel lncRNAs

To test the ability of BPLLDA in predicting novel disease-associated lncRNAs, all known lncRNA-disease associations correlated with a disease were eliminated. We selected two diseases: colorectal cancer and breast cancer (see Table 7). As can be seen, all top five predicted lncRNAs associated with colorectal cancer were confirmed by the newest LncRNADisease database, whereas four of the top five lncRNAs associated with breast cancer were also validated by the database or literature.

**Table 7:** The top five novel disease-correlated lncRNA candidates predicted for colorectal cancer and breast cancer.

**Disease**	**lncRNA**	**Evidence**
Colorectal cancer	H19	lncRNADisease (Tsang et al., 2010)
Colorectal cancer	CDKN2B-AS1	lncRNADisease (Sun et al., 2016)
Colorectal cancer	PVT1	lncRNADisease (Ping et al., 2018)
Colorectal cancer	MEG3	lncRNADisease (Zhu et al., 2018)
Colorectal cancer	MALAT1	lncRNADisease (Ji et al., 2014)
Breast cancer	H19	lncRNADisease (Vennin et al., 2015)
Breast cancer	CDKN2B-AS1	lncRNADisease (Xu et al., 2017)
Breast cancer	PVT1	lncRNADisease (Guan et al., 2007)
Breast cancer	MALAT1	lncRNADisease (Chou et al., 2016)
Breast cancer	B2 SINE RNA	Unconfirmed

Similarly, to test the ability of BPLLDA in predicting novel lncRNA-associated diseases, all known lncRNA-disease associations correlated with an lncRNA were removed. As two case studies, we selected two lncRNAs, *H19*, and *HOTAIR* (see Table 8). In both cases, four of the top five associated diseases were validated by the database and literature. In summary, BPLLDA achieves favorable performances in predicting novel disease-associated lncRNAs and novel lncRNA-associated diseases.

**Table 8:** The top five novel disease-correlated lncRNA candidates predicted for *H19* and *HOTAIR*.

**lncRNA**	**Disease**	**Evidence**
H19	Prostate cancer	lncRNADisease (Zhu et al., 2014)
H19	Tumor	(Matouk et al., 2007)
H19	Cancer	lncRNADisease (DeBaun et al., 2002)
H19	Breast cancer	lncRNADisease (Vennin et al., 2015)
H19	Decreased myogenesis	Unconfirmed
HOTAIR	Cancer	lncRNADisease (Gupta et al., 2010)
HOTAIR	Breast cancer	lncRNADisease (Xue et al., 2016)
HOTAIR	Hepatocellular carcinoma	lncRNADisease (Yang et al., 2011)
HOTAIR	Prostate cancer	lncRNADisease (Zhang et al., 2015)
HOTAIR	Tumor	Unconfirmed

## Conclusions

Many studies have demonstrated that lncRNAs are essential in many physiological processes related to human diseases. They could be important biomarkers for the diagnosis, prognosis, and treatment of these diseases. However, the biological experiments to validate lncRNA-disease associations are not only time consuming but also costly, which promotes the need for developing computational prediction models. In this study, we proposed BPLLDA, a novel computational method to predict lncRNA-disease associations based on simple paths with limited lengths in a heterogeneous network consisting of the lncRNA similarity network, the disease similarity network, and the lncRNA-disease association network. BPLLDA outperforms two compared methods in prediction accuracy, and most top predicted novel lncRNA-disease associations were validated by literature. However, there are a few limitations of BPLLDA. First, available experimentally validated lncRNA-disease associations are rather incomplete. Secondly, lncRNA similarity is computed on the basis of known lncRNA-disease associations. There is a problem of sparseness in the disease semantic similarity and lncRNA functional similarity, which is remedied by integrating the Gaussian interaction profile kernel similarity for diseases and lncRNAs, respectively. So, BPLLDA may result in biased predictions. Finally, the distance-decay function in BPLLDA is relatively simple and could be improved by machine learning methods.

## Author contributions

JY and BL: conceived the concept of the work and designed the experiments; XX, JX, BJ and YY: performed the literature search; XX, WZ, CG, and LP: collected and analyzed the data; XX and JY: wrote the paper, and all authors have approved the manuscript.

### Conflict of interest statement

The authors declare that the research was conducted in the absence of any commercial or financial relationships that could be construed as a potential conflict of interest.
