# Supplementary material for: BPLLDA: Predicting lncRNA-Disease Associations Based on Simple Paths With Limited Lengths in a Heterogeneous Network
Source: Front Genet. 2018 Oct 16;9:411. doi: 10.3389/fgene.2018.00411 (PMC6232683; doi:10.3389/fgene.2018.00411)
Supplement: Supplementary file 1 [file Table_1.DOCX]

Annotation: The positive number represents a disease and the negative number represents a lncRNA

36 -> 3 -> 4 -> -101

36 -> 3 -> 21 -> -73

36 -> 3 -> 21 -> -101

36 -> 3 -> 21 -> -154

36 -> 3 -> 27 -> -19

36 -> 3 -> 27 -> -20

36 -> 3 -> 27 -> -32

36 -> 3 -> 27 -> -62

36 -> 3 -> 27 -> -68

36 -> 3 -> 27 -> -73

36 -> 3 -> 27 -> -81

36 -> 3 -> 27 -> -98

36 -> 3 -> 27 -> -99

36 -> 3 -> 27 -> -101

36 -> 3 -> 27 -> -107

36 -> 3 -> 27 -> -124

36 -> 3 -> 27 -> -133

36 -> 3 -> 27 -> -145

36 -> 3 -> 29 -> -133

36 -> 3 -> 30 -> -1

36 -> 3 -> 30 -> -3

36 -> 3 -> 30 -> -32

36 -> 3 -> 30 -> -39

36 -> 3 -> 30 -> -63

36 -> 3 -> 30 -> -73

36 -> 3 -> 30 -> -81

36 -> 3 -> 30 -> -99

36 -> 3 -> 30 -> -101

36 -> 3 -> 30 -> -106

36 -> 3 -> 30 -> -117

36 -> 3 -> 30 -> -133

36 -> 3 -> 30 -> -137

36 -> 3 -> 30 -> -150

36 -> 3 -> 30 -> -156

36 -> 3 -> 31 -> -1

36 -> 3 -> 34 -> -32

36 -> 3 -> 34 -> -71

36 -> 3 -> 34 -> -113

36 -> 3 -> 38 -> -55

36 -> 3 -> 38 -> -56

36 -> 3 -> 38 -> -105

36 -> 3 -> 39 -> -101

36 -> 3 -> 40 -> -73

36 -> 3 -> 60 -> -64

36 -> 3 -> 73 -> -32

36 -> 3 -> 75 -> -1

36 -> 3 -> 75 -> -29

36 -> 3 -> 76 -> -81

36 -> 3 -> 77 -> -73

36 -> 3 -> 92 -> -133

36 -> 3 -> 100 -> -32

36 -> 3 -> 100 -> -38

36 -> 3 -> 103 -> -78

36 -> 3 -> 105 -> -32

36 -> 3 -> 105 -> -146

36 -> 3 -> 107 -> -73

36 -> 3 -> 107 -> -99

36 -> 3 -> 110 -> -56

36 -> 3 -> 117 -> -73

36 -> 3 -> 117 -> -101

36 -> 3 -> 122 -> -56

36 -> 3 -> 128 -> -32

36 -> 3 -> 128 -> -95

36 -> 3 -> 129 -> -32

36 -> 3 -> 136 -> -20

36 -> 3 -> 138 -> -20

36 -> 3 -> 138 -> -61

36 -> 3 -> 138 -> -73

36 -> 3 -> 138 -> -98

36 -> 3 -> 138 -> -133

36 -> 3 -> 148 -> -32

36 -> 3 -> 156 -> -32

36 -> 3 -> 156 -> -44

36 -> 3 -> 161 -> -78

36 -> 3 -> 179 -> -22

36 -> 3 -> 179 -> -147

36 -> 3 -> 180 -> -20

36 -> 3 -> 186 -> -99

36 -> 3 -> -32

36 -> 3 -> -32 -> -1

36 -> 3 -> -32 -> -3

36 -> 3 -> -32 -> -14

36 -> 3 -> -32 -> -17

36 -> 3 -> -32 -> -19

36 -> 3 -> -32 -> -20

36 -> 3 -> -32 -> -22

36 -> 3 -> -32 -> -29

36 -> 3 -> -32 -> -30

36 -> 3 -> -32 -> -34

36 -> 3 -> -32 -> -35

36 -> 3 -> -32 -> -37

36 -> 3 -> -32 -> -38

36 -> 3 -> -32 -> -39

36 -> 3 -> -32 -> -43

36 -> 3 -> -32 -> -55

36 -> 3 -> -32 -> -56

36 -> 3 -> -32 -> -62

36 -> 3 -> -32 -> -63

36 -> 3 -> -32 -> -64

36 -> 3 -> -32 -> -68

36 -> 3 -> -32 -> -71

36 -> 3 -> -32 -> -73

36 -> 3 -> -32 -> -78

36 -> 3 -> -32 -> -81

36 -> 3 -> -32 -> -86

36 -> 3 -> -32 -> -92

36 -> 3 -> -32 -> -94

36 -> 3 -> -32 -> -95

36 -> 3 -> -32 -> -98

36 -> 3 -> -32 -> -99

36 -> 3 -> -32 -> -101

36 -> 3 -> -32 -> -103

36 -> 3 -> -32 -> -105

36 -> 3 -> -32 -> -106

36 -> 3 -> -32 -> -107

36 -> 3 -> -32 -> -113

36 -> 3 -> -32 -> -117

36 -> 3 -> -32 -> -124

36 -> 3 -> -32 -> -125

36 -> 3 -> -32 -> -133

36 -> 3 -> -32 -> -137

36 -> 3 -> -32 -> -144

36 -> 3 -> -32 -> -145

36 -> 3 -> -32 -> -146

36 -> 3 -> -32 -> -147

36 -> 3 -> -32 -> -150

36 -> 3 -> -32 -> -154

36 -> 3 -> -32 -> -156

36 -> 3 -> -42 -> -1

36 -> 3 -> -42 -> -3

36 -> 3 -> -42 -> -4

36 -> 3 -> -42 -> -8

36 -> 3 -> -42 -> -9

36 -> 3 -> -42 -> -10

36 -> 3 -> -42 -> -19

36 -> 3 -> -42 -> -22

36 -> 3 -> -42 -> -24

36 -> 3 -> -42 -> -31

36 -> 3 -> -42 -> -33

36 -> 3 -> -42 -> -39

36 -> 3 -> -42 -> -44

36 -> 3 -> -42 -> -45

36 -> 3 -> -42 -> -46

36 -> 3 -> -42 -> -47

36 -> 3 -> -42 -> -49

36 -> 3 -> -42 -> -55

36 -> 3 -> -42 -> -56

36 -> 3 -> -42 -> -58

36 -> 3 -> -42 -> -60

36 -> 3 -> -42 -> -62

36 -> 3 -> -42 -> -63

36 -> 3 -> -42 -> -66

36 -> 3 -> -42 -> -69

36 -> 3 -> -42 -> -71

36 -> 3 -> -42 -> -77

36 -> 3 -> -42 -> -81

36 -> 3 -> -42 -> -84

36 -> 3 -> -42 -> -91

36 -> 3 -> -42 -> -96

36 -> 3 -> -42 -> -98

36 -> 3 -> -42 -> -105

36 -> 3 -> -42 -> -106

36 -> 3 -> -42 -> -107

36 -> 3 -> -42 -> -113

36 -> 3 -> -42 -> -117

36 -> 3 -> -42 -> -120

36 -> 3 -> -42 -> -124

36 -> 3 -> -42 -> -131

36 -> 3 -> -42 -> -132

36 -> 3 -> -42 -> -135

36 -> 3 -> -42 -> -136

36 -> 3 -> -42 -> -137

36 -> 3 -> -42 -> -141

36 -> 3 -> -42 -> -146

36 -> 3 -> -42 -> -147

36 -> 3 -> -42 -> -150

36 -> 3 -> -42 -> -156

36 -> 4 -> 3 -> -32

36 -> 4 -> 3 -> -42

36 -> 4 -> 7 -> -54

36 -> 4 -> 9 -> -6

36 -> 4 -> 14 -> -54

36 -> 4 -> 15 -> -68

36 -> 4 -> 19 -> -73

36 -> 4 -> 21 -> -73

36 -> 4 -> 21 -> -101

36 -> 4 -> 21 -> -154

36 -> 4 -> 22 -> -152

36 -> 4 -> 24 -> -49

36 -> 4 -> 25 -> -132

36 -> 4 -> 27 -> -19

36 -> 4 -> 27 -> -20

36 -> 4 -> 27 -> -32

36 -> 4 -> 27 -> -62

36 -> 4 -> 27 -> -68

36 -> 4 -> 27 -> -73

36 -> 4 -> 27 -> -81

36 -> 4 -> 27 -> -98

36 -> 4 -> 27 -> -99

36 -> 4 -> 27 -> -101

36 -> 4 -> 27 -> -107

36 -> 4 -> 27 -> -124

36 -> 4 -> 27 -> -133

36 -> 4 -> 27 -> -145

36 -> 4 -> 28 -> -81

36 -> 4 -> 29 -> -133

36 -> 4 -> 30 -> -1

36 -> 4 -> 30 -> -3

36 -> 4 -> 30 -> -32

36 -> 4 -> 30 -> -39

36 -> 4 -> 30 -> -63

36 -> 4 -> 30 -> -73

36 -> 4 -> 30 -> -81

36 -> 4 -> 30 -> -99

36 -> 4 -> 30 -> -101

36 -> 4 -> 30 -> -106

36 -> 4 -> 30 -> -117

36 -> 4 -> 30 -> -133

36 -> 4 -> 30 -> -137

36 -> 4 -> 30 -> -150

36 -> 4 -> 30 -> -156

36 -> 4 -> 32 -> -9

36 -> 4 -> 33 -> -145

36 -> 4 -> 38 -> -55

36 -> 4 -> 38 -> -56

36 -> 4 -> 38 -> -105

36 -> 4 -> 39 -> -101

36 -> 4 -> 40 -> -73

36 -> 4 -> 41 -> -6

36 -> 4 -> 42 -> -133

36 -> 4 -> 44 -> -29

36 -> 4 -> 44 -> -48

36 -> 4 -> 44 -> -81

36 -> 4 -> 44 -> -89

36 -> 4 -> 44 -> -99

36 -> 4 -> 44 -> -114

36 -> 4 -> 45 -> -73

36 -> 4 -> 48 -> -99

36 -> 4 -> 49 -> -15

36 -> 4 -> 55 -> -12

36 -> 4 -> 60 -> -64

36 -> 4 -> 64 -> -10

36 -> 4 -> 65 -> -4

36 -> 4 -> 66 -> -136

36 -> 4 -> 67 -> -135

36 -> 4 -> 68 -> -73

36 -> 4 -> 69 -> -77

36 -> 4 -> 73 -> -32

36 -> 4 -> 74 -> -112

36 -> 4 -> 75 -> -1

36 -> 4 -> 75 -> -29

36 -> 4 -> 76 -> -81

36 -> 4 -> 77 -> -73

36 -> 4 -> 79 -> -73

36 -> 4 -> 84 -> -73

36 -> 4 -> 87 -> -73

36 -> 4 -> 88 -> -83

36 -> 4 -> 89 -> -6

36 -> 4 -> 89 -> -73

36 -> 4 -> 89 -> -81

36 -> 4 -> 89 -> -83

36 -> 4 -> 89 -> -86

36 -> 4 -> 89 -> -99

36 -> 4 -> 89 -> -101

36 -> 4 -> 89 -> -114

36 -> 4 -> 89 -> -155

36 -> 4 -> 91 -> -101

36 -> 4 -> 92 -> -133

36 -> 4 -> 97 -> -15

36 -> 4 -> 103 -> -78

36 -> 4 -> 105 -> -32

36 -> 4 -> 105 -> -146

36 -> 4 -> 107 -> -73

36 -> 4 -> 107 -> -99

36 -> 4 -> 108 -> -80

36 -> 4 -> 110 -> -56

36 -> 4 -> 113 -> -73

36 -> 4 -> 116 -> -73

36 -> 4 -> 118 -> -73

36 -> 4 -> 120 -> -133

36 -> 4 -> 121 -> -101

36 -> 4 -> 121 -> -141

36 -> 4 -> 122 -> -56

36 -> 4 -> 123 -> -73

36 -> 4 -> 128 -> -32

36 -> 4 -> 128 -> -95

36 -> 4 -> 129 -> -32

36 -> 4 -> 130 -> -73

36 -> 4 -> 134 -> -99

36 -> 4 -> 136 -> -20

36 -> 4 -> 139 -> -15

36 -> 4 -> 142 -> -154

36 -> 4 -> 146 -> -20

36 -> 4 -> 148 -> -32

36 -> 4 -> 149 -> -73

36 -> 4 -> 150 -> -91

36 -> 4 -> 153 -> -2

36 -> 4 -> 160 -> -129

36 -> 4 -> 161 -> -78

36 -> 4 -> 162 -> -133

36 -> 4 -> 163 -> -8

36 -> 4 -> 170 -> -138

36 -> 4 -> 171 -> -13

36 -> 4 -> 172 -> -58

36 -> 4 -> 176 -> -33

36 -> 4 -> 177 -> -106

36 -> 4 -> 179 -> -22

36 -> 4 -> 179 -> -147

36 -> 4 -> 180 -> -20

36 -> 4 -> 181 -> -84

36 -> 4 -> 186 -> -99

36 -> 4 -> 188 -> -24

36 -> 4 -> 189 -> -73

36 -> 4 -> -101

36 -> 4 -> -101 -> -1

36 -> 4 -> -101 -> -3

36 -> 4 -> -101 -> -6

36 -> 4 -> -101 -> -17

36 -> 4 -> -101 -> -19

36 -> 4 -> -101 -> -20

36 -> 4 -> -101 -> -22

36 -> 4 -> -101 -> -26

36 -> 4 -> -101 -> -28

36 -> 4 -> -101 -> -29

36 -> 4 -> -101 -> -30

36 -> 4 -> -101 -> -32

36 -> 4 -> -101 -> -35

36 -> 4 -> -101 -> -36

36 -> 4 -> -101 -> -39

36 -> 4 -> -101 -> -41

36 -> 4 -> -101 -> -43

36 -> 4 -> -101 -> -48

36 -> 4 -> -101 -> -51

36 -> 4 -> -101 -> -56

36 -> 4 -> -101 -> -61

36 -> 4 -> -101 -> -62

36 -> 4 -> -101 -> -63

36 -> 4 -> -101 -> -64

36 -> 4 -> -101 -> -68

36 -> 4 -> -101 -> -73

36 -> 4 -> -101 -> -74

36 -> 4 -> -101 -> -75

36 -> 4 -> -101 -> -78

36 -> 4 -> -101 -> -81

36 -> 4 -> -101 -> -83

36 -> 4 -> -101 -> -86

36 -> 4 -> -101 -> -89

36 -> 4 -> -101 -> -92

36 -> 4 -> -101 -> -95

36 -> 4 -> -101 -> -98

36 -> 4 -> -101 -> -99

36 -> 4 -> -101 -> -100

36 -> 4 -> -101 -> -106

36 -> 4 -> -101 -> -107

36 -> 4 -> -101 -> -109

36 -> 4 -> -101 -> -111

36 -> 4 -> -101 -> -114

36 -> 4 -> -101 -> -117

36 -> 4 -> -101 -> -118

36 -> 4 -> -101 -> -119

36 -> 4 -> -101 -> -121

36 -> 4 -> -101 -> -122

36 -> 4 -> -101 -> -124

36 -> 4 -> -101 -> -125

36 -> 4 -> -101 -> -127

36 -> 4 -> -101 -> -133

36 -> 4 -> -101 -> -137

36 -> 4 -> -101 -> -144

36 -> 4 -> -101 -> -145

36 -> 4 -> -101 -> -146

36 -> 4 -> -101 -> -147

36 -> 4 -> -101 -> -150

36 -> 4 -> -101 -> -151

36 -> 4 -> -101 -> -154

36 -> 4 -> -101 -> -155

36 -> 4 -> -101 -> -156

36 -> 19 -> 2 -> -32

36 -> 19 -> 4 -> -101

36 -> 19 -> 6 -> -73

36 -> 19 -> 7 -> -54

36 -> 19 -> 9 -> -6

36 -> 19 -> 12 -> -32

36 -> 19 -> 13 -> -32

36 -> 19 -> 13 -> -73

36 -> 19 -> 14 -> -54

36 -> 19 -> 15 -> -68

36 -> 19 -> 16 -> -5

36 -> 19 -> 17 -> -32

36 -> 19 -> 18 -> -73

36 -> 19 -> 18 -> -89

36 -> 19 -> 21 -> -73

36 -> 19 -> 21 -> -101

36 -> 19 -> 21 -> -154

36 -> 19 -> 22 -> -152

36 -> 19 -> 24 -> -49

36 -> 19 -> 25 -> -132

36 -> 19 -> 26 -> -14

36 -> 19 -> 28 -> -81

36 -> 19 -> 29 -> -133

36 -> 19 -> 31 -> -1

36 -> 19 -> 32 -> -9

36 -> 19 -> 33 -> -145

36 -> 19 -> 37 -> -73

36 -> 19 -> 39 -> -101

36 -> 19 -> 40 -> -73

36 -> 19 -> 41 -> -6

36 -> 19 -> 42 -> -133

36 -> 19 -> 43 -> -73

36 -> 19 -> 43 -> -99

36 -> 19 -> 43 -> -101

36 -> 19 -> 45 -> -73

36 -> 19 -> 46 -> -32

36 -> 19 -> 48 -> -99

36 -> 19 -> 49 -> -15

36 -> 19 -> 50 -> -54

36 -> 19 -> 51 -> -2

36 -> 19 -> 52 -> -32

36 -> 19 -> 53 -> -133

36 -> 19 -> 54 -> -53

36 -> 19 -> 55 -> -12

36 -> 19 -> 56 -> -115

36 -> 19 -> 57 -> -103

36 -> 19 -> 58 -> -90

36 -> 19 -> 59 -> -148

36 -> 19 -> 60 -> -64

36 -> 19 -> 61 -> -27

36 -> 19 -> 62 -> -99

36 -> 19 -> 63 -> -32

36 -> 19 -> 64 -> -10

36 -> 19 -> 65 -> -4

36 -> 19 -> 66 -> -136

36 -> 19 -> 67 -> -135

36 -> 19 -> 68 -> -73

36 -> 19 -> 69 -> -77

36 -> 19 -> 70 -> -52

36 -> 19 -> 71 -> -32

36 -> 19 -> 73 -> -32

36 -> 19 -> 74 -> -112

36 -> 19 -> 76 -> -81

36 -> 19 -> 77 -> -73

36 -> 19 -> 78 -> -73

36 -> 19 -> 79 -> -73

36 -> 19 -> 80 -> -32

36 -> 19 -> 81 -> -73

36 -> 19 -> 83 -> -125

36 -> 19 -> 84 -> -73

36 -> 19 -> 85 -> -12

36 -> 19 -> 87 -> -73

36 -> 19 -> 88 -> -83

36 -> 19 -> 90 -> -103

36 -> 19 -> 91 -> -101

36 -> 19 -> 92 -> -133

36 -> 19 -> 93 -> -145

36 -> 19 -> 94 -> -13

36 -> 19 -> 96 -> -73

36 -> 19 -> 97 -> -15

36 -> 19 -> 99 -> -73

36 -> 19 -> 101 -> -64

36 -> 19 -> 102 -> -116

36 -> 19 -> 103 -> -78

36 -> 19 -> 104 -> -2

36 -> 19 -> 106 -> -65

36 -> 19 -> 107 -> -73

36 -> 19 -> 107 -> -99

36 -> 19 -> 108 -> -80

36 -> 19 -> 110 -> -56

36 -> 19 -> 112 -> -54

36 -> 19 -> 113 -> -73

36 -> 19 -> 114 -> -73

36 -> 19 -> 116 -> -73

36 -> 19 -> 117 -> -73

36 -> 19 -> 117 -> -101

36 -> 19 -> 118 -> -73

36 -> 19 -> 120 -> -133

36 -> 19 -> 122 -> -56

36 -> 19 -> 123 -> -73

36 -> 19 -> 125 -> -104

36 -> 19 -> 126 -> -59

36 -> 19 -> 127 -> -93

36 -> 19 -> 129 -> -32

36 -> 19 -> 130 -> -73

36 -> 19 -> 132 -> -32

36 -> 19 -> 133 -> -101

36 -> 19 -> 134 -> -99

36 -> 19 -> 135 -> -21

36 -> 19 -> 135 -> -73

36 -> 19 -> 135 -> -139

36 -> 19 -> 136 -> -20

36 -> 19 -> 139 -> -15

36 -> 19 -> 142 -> -154

36 -> 19 -> 143 -> -50

36 -> 19 -> 145 -> -125

36 -> 19 -> 146 -> -20

36 -> 19 -> 147 -> -32

36 -> 19 -> 148 -> -32

36 -> 19 -> 149 -> -73

36 -> 19 -> 150 -> -91

36 -> 19 -> 151 -> -101

36 -> 19 -> 152 -> -36

36 -> 19 -> 153 -> -2

36 -> 19 -> 155 -> -73

36 -> 19 -> 158 -> -72

36 -> 19 -> 160 -> -129

36 -> 19 -> 161 -> -78

36 -> 19 -> 162 -> -133

36 -> 19 -> 163 -> -8

36 -> 19 -> 164 -> -134

36 -> 19 -> 165 -> -54

36 -> 19 -> 168 -> -73

36 -> 19 -> 168 -> -102

36 -> 19 -> 169 -> -99

36 -> 19 -> 170 -> -138

36 -> 19 -> 171 -> -13

36 -> 19 -> 172 -> -58

36 -> 19 -> 173 -> -154

36 -> 19 -> 174 -> -114

36 -> 19 -> 176 -> -33

36 -> 19 -> 177 -> -106

36 -> 19 -> 178 -> -69

36 -> 19 -> 180 -> -20

36 -> 19 -> 181 -> -84

36 -> 19 -> 182 -> -73

36 -> 19 -> 186 -> -99

36 -> 19 -> 187 -> -53

36 -> 19 -> 188 -> -24

36 -> 19 -> 189 -> -73

36 -> 19 -> -73

36 -> 19 -> -73 -> -1

36 -> 19 -> -73 -> -3

36 -> 19 -> -73 -> -6

36 -> 19 -> -73 -> -17

36 -> 19 -> -73 -> -19

36 -> 19 -> -73 -> -20

36 -> 19 -> -73 -> -22

36 -> 19 -> -73 -> -26

36 -> 19 -> -73 -> -27

36 -> 19 -> -73 -> -28

36 -> 19 -> -73 -> -29

36 -> 19 -> -73 -> -30

36 -> 19 -> -73 -> -32

36 -> 19 -> -73 -> -35

36 -> 19 -> -73 -> -39

36 -> 19 -> -73 -> -43

36 -> 19 -> -73 -> -48

36 -> 19 -> -73 -> -56

36 -> 19 -> -73 -> -61

36 -> 19 -> -73 -> -62

36 -> 19 -> -73 -> -63

36 -> 19 -> -73 -> -64

36 -> 19 -> -73 -> -68

36 -> 19 -> -73 -> -71

36 -> 19 -> -73 -> -78

36 -> 19 -> -73 -> -81

36 -> 19 -> -73 -> -83

36 -> 19 -> -73 -> -86

36 -> 19 -> -73 -> -88

36 -> 19 -> -73 -> -89

36 -> 19 -> -73 -> -92

36 -> 19 -> -73 -> -95

36 -> 19 -> -73 -> -98

36 -> 19 -> -73 -> -99

36 -> 19 -> -73 -> -101

36 -> 19 -> -73 -> -106

36 -> 19 -> -73 -> -107

36 -> 19 -> -73 -> -113

36 -> 19 -> -73 -> -114

36 -> 19 -> -73 -> -117

36 -> 19 -> -73 -> -118

36 -> 19 -> -73 -> -119

36 -> 19 -> -73 -> -121

36 -> 19 -> -73 -> -122

36 -> 19 -> -73 -> -124

36 -> 19 -> -73 -> -133

36 -> 19 -> -73 -> -137

36 -> 19 -> -73 -> -144

36 -> 19 -> -73 -> -145

36 -> 19 -> -73 -> -146

36 -> 19 -> -73 -> -147

36 -> 19 -> -73 -> -150

36 -> 19 -> -73 -> -154

36 -> 19 -> -73 -> -155

36 -> 19 -> -73 -> -156

36 -> 21 -> 3 -> -32

36 -> 21 -> 3 -> -42

36 -> 21 -> 4 -> -101

36 -> 21 -> 6 -> -73

36 -> 21 -> 17 -> -32

36 -> 21 -> 19 -> -73

36 -> 21 -> 27 -> -19

36 -> 21 -> 27 -> -20

36 -> 21 -> 27 -> -32

36 -> 21 -> 27 -> -62

36 -> 21 -> 27 -> -68

36 -> 21 -> 27 -> -73

36 -> 21 -> 27 -> -81

36 -> 21 -> 27 -> -98

36 -> 21 -> 27 -> -99

36 -> 21 -> 27 -> -101

36 -> 21 -> 27 -> -107

36 -> 21 -> 27 -> -124

36 -> 21 -> 27 -> -133

36 -> 21 -> 27 -> -145

36 -> 21 -> 29 -> -133

36 -> 21 -> 30 -> -1

36 -> 21 -> 30 -> -3

36 -> 21 -> 30 -> -32

36 -> 21 -> 30 -> -39

36 -> 21 -> 30 -> -63

36 -> 21 -> 30 -> -73

36 -> 21 -> 30 -> -81

36 -> 21 -> 30 -> -99

36 -> 21 -> 30 -> -101

36 -> 21 -> 30 -> -106

36 -> 21 -> 30 -> -117

36 -> 21 -> 30 -> -133

36 -> 21 -> 30 -> -137

36 -> 21 -> 30 -> -150

36 -> 21 -> 30 -> -156

36 -> 21 -> 38 -> -55

36 -> 21 -> 38 -> -56

36 -> 21 -> 38 -> -105

36 -> 21 -> 39 -> -101

36 -> 21 -> 40 -> -73

36 -> 21 -> 43 -> -73

36 -> 21 -> 43 -> -99

36 -> 21 -> 43 -> -101

36 -> 21 -> 44 -> -29

36 -> 21 -> 44 -> -48

36 -> 21 -> 44 -> -81

36 -> 21 -> 44 -> -89

36 -> 21 -> 44 -> -99

36 -> 21 -> 44 -> -114

36 -> 21 -> 45 -> -73

36 -> 21 -> 60 -> -64

36 -> 21 -> 61 -> -27

36 -> 21 -> 68 -> -73

36 -> 21 -> 75 -> -1

36 -> 21 -> 75 -> -29

36 -> 21 -> 76 -> -81

36 -> 21 -> 77 -> -73

36 -> 21 -> 79 -> -73

36 -> 21 -> 81 -> -73

36 -> 21 -> 82 -> -30

36 -> 21 -> 82 -> -32

36 -> 21 -> 82 -> -35

36 -> 21 -> 82 -> -101

36 -> 21 -> 83 -> -125

36 -> 21 -> 84 -> -73

36 -> 21 -> 87 -> -73

36 -> 21 -> 89 -> -6

36 -> 21 -> 89 -> -73

36 -> 21 -> 89 -> -81

36 -> 21 -> 89 -> -83

36 -> 21 -> 89 -> -86

36 -> 21 -> 89 -> -99

36 -> 21 -> 89 -> -101

36 -> 21 -> 89 -> -114

36 -> 21 -> 89 -> -155

36 -> 21 -> 91 -> -101

36 -> 21 -> 92 -> -133

36 -> 21 -> 103 -> -78

36 -> 21 -> 105 -> -32

36 -> 21 -> 105 -> -146

36 -> 21 -> 107 -> -73

36 -> 21 -> 107 -> -99

36 -> 21 -> 113 -> -73

36 -> 21 -> 114 -> -73

36 -> 21 -> 115 -> -17

36 -> 21 -> 115 -> -32

36 -> 21 -> 115 -> -68

36 -> 21 -> 115 -> -73

36 -> 21 -> 115 -> -92

36 -> 21 -> 116 -> -73

36 -> 21 -> 117 -> -73

36 -> 21 -> 117 -> -101

36 -> 21 -> 118 -> -73

36 -> 21 -> 122 -> -56

36 -> 21 -> 123 -> -73

36 -> 21 -> 128 -> -32

36 -> 21 -> 128 -> -95

36 -> 21 -> 129 -> -32

36 -> 21 -> 130 -> -73

36 -> 21 -> 133 -> -101

36 -> 21 -> 134 -> -99

36 -> 21 -> 136 -> -20

36 -> 21 -> 141 -> -51

36 -> 21 -> 141 -> -100

36 -> 21 -> 141 -> -127

36 -> 21 -> 142 -> -154

36 -> 21 -> 144 -> -7

36 -> 21 -> 144 -> -110

36 -> 21 -> 144 -> -130

36 -> 21 -> 146 -> -20

36 -> 21 -> 149 -> -73

36 -> 21 -> 151 -> -101

36 -> 21 -> 152 -> -36

36 -> 21 -> 161 -> -78

36 -> 21 -> 169 -> -99

36 -> 21 -> 173 -> -154

36 -> 21 -> 174 -> -114

36 -> 21 -> 179 -> -22

36 -> 21 -> 179 -> -147

36 -> 21 -> 180 -> -20

36 -> 21 -> 182 -> -73

36 -> 21 -> 186 -> -99

36 -> 21 -> 189 -> -73

36 -> 21 -> 190 -> -43

36 -> 21 -> 190 -> -73

36 -> 21 -> 190 -> -86

36 -> 21 -> 190 -> -88

36 -> 21 -> 190 -> -144

36 -> 21 -> -73

36 -> 21 -> -73 -> -1

36 -> 21 -> -73 -> -3

36 -> 21 -> -73 -> -6

36 -> 21 -> -73 -> -17

36 -> 21 -> -73 -> -19

36 -> 21 -> -73 -> -20

36 -> 21 -> -73 -> -22

36 -> 21 -> -73 -> -26

36 -> 21 -> -73 -> -27

36 -> 21 -> -73 -> -28

36 -> 21 -> -73 -> -29

36 -> 21 -> -73 -> -30

36 -> 21 -> -73 -> -32

36 -> 21 -> -73 -> -35

36 -> 21 -> -73 -> -39

36 -> 21 -> -73 -> -43

36 -> 21 -> -73 -> -48

36 -> 21 -> -73 -> -56

36 -> 21 -> -73 -> -61

36 -> 21 -> -73 -> -62

36 -> 21 -> -73 -> -63

36 -> 21 -> -73 -> -64

36 -> 21 -> -73 -> -68

36 -> 21 -> -73 -> -71

36 -> 21 -> -73 -> -78

36 -> 21 -> -73 -> -81

36 -> 21 -> -73 -> -83

36 -> 21 -> -73 -> -86

36 -> 21 -> -73 -> -88

36 -> 21 -> -73 -> -89

36 -> 21 -> -73 -> -92

36 -> 21 -> -73 -> -95

36 -> 21 -> -73 -> -98

36 -> 21 -> -73 -> -99

36 -> 21 -> -73 -> -101

36 -> 21 -> -73 -> -106

36 -> 21 -> -73 -> -107

36 -> 21 -> -73 -> -113

36 -> 21 -> -73 -> -114

36 -> 21 -> -73 -> -117

36 -> 21 -> -73 -> -118

36 -> 21 -> -73 -> -119

36 -> 21 -> -73 -> -121

36 -> 21 -> -73 -> -122

36 -> 21 -> -73 -> -124

36 -> 21 -> -73 -> -133

36 -> 21 -> -73 -> -137

36 -> 21 -> -73 -> -144

36 -> 21 -> -73 -> -145

36 -> 21 -> -73 -> -146

36 -> 21 -> -73 -> -147

36 -> 21 -> -73 -> -150

36 -> 21 -> -73 -> -154

36 -> 21 -> -73 -> -155

36 -> 21 -> -73 -> -156

36 -> 21 -> -101

36 -> 21 -> -101 -> -1

36 -> 21 -> -101 -> -3

36 -> 21 -> -101 -> -6

36 -> 21 -> -101 -> -17

36 -> 21 -> -101 -> -19

36 -> 21 -> -101 -> -20

36 -> 21 -> -101 -> -22

36 -> 21 -> -101 -> -26

36 -> 21 -> -101 -> -28

36 -> 21 -> -101 -> -29

36 -> 21 -> -101 -> -30

36 -> 21 -> -101 -> -32

36 -> 21 -> -101 -> -35

36 -> 21 -> -101 -> -36

36 -> 21 -> -101 -> -39

36 -> 21 -> -101 -> -41

36 -> 21 -> -101 -> -43

36 -> 21 -> -101 -> -48

36 -> 21 -> -101 -> -51

36 -> 21 -> -101 -> -56

36 -> 21 -> -101 -> -61

36 -> 21 -> -101 -> -62

36 -> 21 -> -101 -> -63

36 -> 21 -> -101 -> -64

36 -> 21 -> -101 -> -68

36 -> 21 -> -101 -> -73

36 -> 21 -> -101 -> -74

36 -> 21 -> -101 -> -75

36 -> 21 -> -101 -> -78

36 -> 21 -> -101 -> -81

36 -> 21 -> -101 -> -83

36 -> 21 -> -101 -> -86

36 -> 21 -> -101 -> -89

36 -> 21 -> -101 -> -92

36 -> 21 -> -101 -> -95

36 -> 21 -> -101 -> -98

36 -> 21 -> -101 -> -99

36 -> 21 -> -101 -> -100

36 -> 21 -> -101 -> -106

36 -> 21 -> -101 -> -107

36 -> 21 -> -101 -> -109

36 -> 21 -> -101 -> -111

36 -> 21 -> -101 -> -114

36 -> 21 -> -101 -> -117

36 -> 21 -> -101 -> -118

36 -> 21 -> -101 -> -119

36 -> 21 -> -101 -> -121

36 -> 21 -> -101 -> -122

36 -> 21 -> -101 -> -124

36 -> 21 -> -101 -> -125

36 -> 21 -> -101 -> -127

36 -> 21 -> -101 -> -133

36 -> 21 -> -101 -> -137

36 -> 21 -> -101 -> -144

36 -> 21 -> -101 -> -145

36 -> 21 -> -101 -> -146

36 -> 21 -> -101 -> -147

36 -> 21 -> -101 -> -150

36 -> 21 -> -101 -> -151

36 -> 21 -> -101 -> -154

36 -> 21 -> -101 -> -155

36 -> 21 -> -101 -> -156

36 -> 21 -> -154

36 -> 21 -> -154 -> -1

36 -> 21 -> -154 -> -3

36 -> 21 -> -154 -> -6

36 -> 21 -> -154 -> -7

36 -> 21 -> -154 -> -17

36 -> 21 -> -154 -> -19

36 -> 21 -> -154 -> -20

36 -> 21 -> -154 -> -29

36 -> 21 -> -154 -> -30

36 -> 21 -> -154 -> -32

36 -> 21 -> -154 -> -35

36 -> 21 -> -154 -> -36

36 -> 21 -> -154 -> -39

36 -> 21 -> -154 -> -43

36 -> 21 -> -154 -> -48

36 -> 21 -> -154 -> -51

36 -> 21 -> -154 -> -56

36 -> 21 -> -154 -> -62

36 -> 21 -> -154 -> -63

36 -> 21 -> -154 -> -64

36 -> 21 -> -154 -> -73

36 -> 21 -> -154 -> -78

36 -> 21 -> -154 -> -81

36 -> 21 -> -154 -> -83

36 -> 21 -> -154 -> -86

36 -> 21 -> -154 -> -89

36 -> 21 -> -154 -> -92

36 -> 21 -> -154 -> -95

36 -> 21 -> -154 -> -98

36 -> 21 -> -154 -> -99

36 -> 21 -> -154 -> -100

36 -> 21 -> -154 -> -101

36 -> 21 -> -154 -> -106

36 -> 21 -> -154 -> -107

36 -> 21 -> -154 -> -110

36 -> 21 -> -154 -> -114

36 -> 21 -> -154 -> -117

36 -> 21 -> -154 -> -124

36 -> 21 -> -154 -> -127

36 -> 21 -> -154 -> -130

36 -> 21 -> -154 -> -133

36 -> 21 -> -154 -> -137

36 -> 21 -> -154 -> -144

36 -> 21 -> -154 -> -146

36 -> 21 -> -154 -> -150

36 -> 21 -> -154 -> -155

36 -> 21 -> -154 -> -156

36 -> 27 -> 3 -> -32

36 -> 27 -> 3 -> -42

36 -> 27 -> 4 -> -101

36 -> 27 -> 6 -> -73

36 -> 27 -> 16 -> -5

36 -> 27 -> 21 -> -73

36 -> 27 -> 21 -> -101

36 -> 27 -> 21 -> -154

36 -> 27 -> 29 -> -133

36 -> 27 -> 30 -> -1

36 -> 27 -> 30 -> -3

36 -> 27 -> 30 -> -32

36 -> 27 -> 30 -> -39

36 -> 27 -> 30 -> -63

36 -> 27 -> 30 -> -73

36 -> 27 -> 30 -> -81

36 -> 27 -> 30 -> -99

36 -> 27 -> 30 -> -101

36 -> 27 -> 30 -> -106

36 -> 27 -> 30 -> -117

36 -> 27 -> 30 -> -133

36 -> 27 -> 30 -> -137

36 -> 27 -> 30 -> -150

36 -> 27 -> 30 -> -156

36 -> 27 -> 34 -> -32

36 -> 27 -> 34 -> -71

36 -> 27 -> 34 -> -113

36 -> 27 -> 37 -> -73

36 -> 27 -> 38 -> -55

36 -> 27 -> 38 -> -56

36 -> 27 -> 38 -> -105

36 -> 27 -> 39 -> -101

36 -> 27 -> 40 -> -73

36 -> 27 -> 43 -> -73

36 -> 27 -> 43 -> -99

36 -> 27 -> 43 -> -101

36 -> 27 -> 44 -> -29

36 -> 27 -> 44 -> -48

36 -> 27 -> 44 -> -81

36 -> 27 -> 44 -> -89

36 -> 27 -> 44 -> -99

36 -> 27 -> 44 -> -114

36 -> 27 -> 60 -> -64

36 -> 27 -> 61 -> -27

36 -> 27 -> 62 -> -99

36 -> 27 -> 63 -> -32

36 -> 27 -> 75 -> -1

36 -> 27 -> 75 -> -29

36 -> 27 -> 76 -> -81

36 -> 27 -> 77 -> -73

36 -> 27 -> 78 -> -73

36 -> 27 -> 81 -> -73

36 -> 27 -> 82 -> -30

36 -> 27 -> 82 -> -32

36 -> 27 -> 82 -> -35

36 -> 27 -> 82 -> -101

36 -> 27 -> 89 -> -6

36 -> 27 -> 89 -> -73

36 -> 27 -> 89 -> -81

36 -> 27 -> 89 -> -83

36 -> 27 -> 89 -> -86

36 -> 27 -> 89 -> -99

36 -> 27 -> 89 -> -101

36 -> 27 -> 89 -> -114

36 -> 27 -> 89 -> -155

36 -> 27 -> 92 -> -133

36 -> 27 -> 99 -> -73

36 -> 27 -> 102 -> -116

36 -> 27 -> 103 -> -78

36 -> 27 -> 105 -> -32

36 -> 27 -> 105 -> -146

36 -> 27 -> 107 -> -73

36 -> 27 -> 107 -> -99

36 -> 27 -> 109 -> -20

36 -> 27 -> 109 -> -73

36 -> 27 -> 109 -> -99

36 -> 27 -> 109 -> -101

36 -> 27 -> 110 -> -56

36 -> 27 -> 115 -> -17

36 -> 27 -> 115 -> -32

36 -> 27 -> 115 -> -68

36 -> 27 -> 115 -> -73

36 -> 27 -> 115 -> -92

36 -> 27 -> 117 -> -73

36 -> 27 -> 117 -> -101

36 -> 27 -> 122 -> -56

36 -> 27 -> 127 -> -93

36 -> 27 -> 128 -> -32

36 -> 27 -> 128 -> -95

36 -> 27 -> 129 -> -32

36 -> 27 -> 134 -> -99

36 -> 27 -> 136 -> -20

36 -> 27 -> 137 -> -18

36 -> 27 -> 137 -> -97

36 -> 27 -> 137 -> -99

36 -> 27 -> 138 -> -20

36 -> 27 -> 138 -> -61

36 -> 27 -> 138 -> -73

36 -> 27 -> 138 -> -98

36 -> 27 -> 138 -> -133

36 -> 27 -> 140 -> -99

36 -> 27 -> 140 -> -133

36 -> 27 -> 144 -> -7

36 -> 27 -> 144 -> -110

36 -> 27 -> 144 -> -130

36 -> 27 -> 146 -> -20

36 -> 27 -> 151 -> -101

36 -> 27 -> 152 -> -36

36 -> 27 -> 157 -> -26

36 -> 27 -> 157 -> -28

36 -> 27 -> 157 -> -32

36 -> 27 -> 157 -> -68

36 -> 27 -> 157 -> -86

36 -> 27 -> 157 -> -99

36 -> 27 -> 157 -> -101

36 -> 27 -> 157 -> -118

36 -> 27 -> 157 -> -119

36 -> 27 -> 157 -> -120

36 -> 27 -> 157 -> -121

36 -> 27 -> 157 -> -122

36 -> 27 -> 157 -> -133

36 -> 27 -> 161 -> -78

36 -> 27 -> 169 -> -99

36 -> 27 -> 174 -> -114

36 -> 27 -> 179 -> -22

36 -> 27 -> 179 -> -147

36 -> 27 -> 180 -> -20

36 -> 27 -> 182 -> -73

36 -> 27 -> 186 -> -99

36 -> 27 -> -19 -> -1

36 -> 27 -> -19 -> -3

36 -> 27 -> -19 -> -4

36 -> 27 -> -19 -> -5

36 -> 27 -> -19 -> -6

36 -> 27 -> -19 -> -7

36 -> 27 -> -19 -> -8

36 -> 27 -> -19 -> -9

36 -> 27 -> -19 -> -10

36 -> 27 -> -19 -> -17

36 -> 27 -> -19 -> -18

36 -> 27 -> -19 -> -20

36 -> 27 -> -19 -> -22

36 -> 27 -> -19 -> -24

36 -> 27 -> -19 -> -26

36 -> 27 -> -19 -> -27

36 -> 27 -> -19 -> -28

36 -> 27 -> -19 -> -29

36 -> 27 -> -19 -> -30

36 -> 27 -> -19 -> -31

36 -> 27 -> -19 -> -32

36 -> 27 -> -19 -> -33

36 -> 27 -> -19 -> -35

36 -> 27 -> -19 -> -36

36 -> 27 -> -19 -> -39

36 -> 27 -> -19 -> -42

36 -> 27 -> -19 -> -43

36 -> 27 -> -19 -> -44

36 -> 27 -> -19 -> -45

36 -> 27 -> -19 -> -46

36 -> 27 -> -19 -> -47

36 -> 27 -> -19 -> -48

36 -> 27 -> -19 -> -49

36 -> 27 -> -19 -> -55

36 -> 27 -> -19 -> -56

36 -> 27 -> -19 -> -58

36 -> 27 -> -19 -> -60

36 -> 27 -> -19 -> -61

36 -> 27 -> -19 -> -62

36 -> 27 -> -19 -> -63

36 -> 27 -> -19 -> -64

36 -> 27 -> -19 -> -66

36 -> 27 -> -19 -> -68

36 -> 27 -> -19 -> -69

36 -> 27 -> -19 -> -71

36 -> 27 -> -19 -> -73

36 -> 27 -> -19 -> -77

36 -> 27 -> -19 -> -78

36 -> 27 -> -19 -> -81

36 -> 27 -> -19 -> -83

36 -> 27 -> -19 -> -84

36 -> 27 -> -19 -> -86

36 -> 27 -> -19 -> -88

36 -> 27 -> -19 -> -89

36 -> 27 -> -19 -> -91

36 -> 27 -> -19 -> -92

36 -> 27 -> -19 -> -93

36 -> 27 -> -19 -> -95

36 -> 27 -> -19 -> -96

36 -> 27 -> -19 -> -97

36 -> 27 -> -19 -> -98

36 -> 27 -> -19 -> -99

36 -> 27 -> -19 -> -101

36 -> 27 -> -19 -> -105

36 -> 27 -> -19 -> -106

36 -> 27 -> -19 -> -107

36 -> 27 -> -19 -> -110

36 -> 27 -> -19 -> -113

36 -> 27 -> -19 -> -114

36 -> 27 -> -19 -> -116

36 -> 27 -> -19 -> -117

36 -> 27 -> -19 -> -118

36 -> 27 -> -19 -> -119

36 -> 27 -> -19 -> -120

36 -> 27 -> -19 -> -121

36 -> 27 -> -19 -> -122

36 -> 27 -> -19 -> -124

36 -> 27 -> -19 -> -130

36 -> 27 -> -19 -> -131

36 -> 27 -> -19 -> -132

36 -> 27 -> -19 -> -133

36 -> 27 -> -19 -> -135

36 -> 27 -> -19 -> -136

36 -> 27 -> -19 -> -137

36 -> 27 -> -19 -> -141

36 -> 27 -> -19 -> -144

36 -> 27 -> -19 -> -145

36 -> 27 -> -19 -> -146

36 -> 27 -> -19 -> -147

36 -> 27 -> -19 -> -150

36 -> 27 -> -19 -> -154

36 -> 27 -> -19 -> -155

36 -> 27 -> -19 -> -156

36 -> 27 -> -20

36 -> 27 -> -20 -> -1

36 -> 27 -> -20 -> -3

36 -> 27 -> -20 -> -6

36 -> 27 -> -20 -> -16

36 -> 27 -> -20 -> -17

36 -> 27 -> -20 -> -19

36 -> 27 -> -20 -> -22

36 -> 27 -> -20 -> -26

36 -> 27 -> -20 -> -27

36 -> 27 -> -20 -> -28

36 -> 27 -> -20 -> -29

36 -> 27 -> -20 -> -32

36 -> 27 -> -20 -> -39

36 -> 27 -> -20 -> -43

36 -> 27 -> -20 -> -48

36 -> 27 -> -20 -> -56

36 -> 27 -> -20 -> -61

36 -> 27 -> -20 -> -62

36 -> 27 -> -20 -> -63

36 -> 27 -> -20 -> -64

36 -> 27 -> -20 -> -68

36 -> 27 -> -20 -> -70

36 -> 27 -> -20 -> -73

36 -> 27 -> -20 -> -74

36 -> 27 -> -20 -> -75

36 -> 27 -> -20 -> -78

36 -> 27 -> -20 -> -81

36 -> 27 -> -20 -> -82

36 -> 27 -> -20 -> -83

36 -> 27 -> -20 -> -86

36 -> 27 -> -20 -> -88

36 -> 27 -> -20 -> -89

36 -> 27 -> -20 -> -92

36 -> 27 -> -20 -> -95

36 -> 27 -> -20 -> -98

36 -> 27 -> -20 -> -99

36 -> 27 -> -20 -> -101

36 -> 27 -> -20 -> -106

36 -> 27 -> -20 -> -107

36 -> 27 -> -20 -> -114

36 -> 27 -> -20 -> -117

36 -> 27 -> -20 -> -118

36 -> 27 -> -20 -> -119

36 -> 27 -> -20 -> -121

36 -> 27 -> -20 -> -122

36 -> 27 -> -20 -> -124

36 -> 27 -> -20 -> -125

36 -> 27 -> -20 -> -133

36 -> 27 -> -20 -> -137

36 -> 27 -> -20 -> -140

36 -> 27 -> -20 -> -143

36 -> 27 -> -20 -> -144

36 -> 27 -> -20 -> -145

36 -> 27 -> -20 -> -146

36 -> 27 -> -20 -> -147

36 -> 27 -> -20 -> -150

36 -> 27 -> -20 -> -152

36 -> 27 -> -20 -> -154

36 -> 27 -> -20 -> -155

36 -> 27 -> -20 -> -156

36 -> 27 -> -32

36 -> 27 -> -32 -> -1

36 -> 27 -> -32 -> -3

36 -> 27 -> -32 -> -14

36 -> 27 -> -32 -> -17

36 -> 27 -> -32 -> -19

36 -> 27 -> -32 -> -20

36 -> 27 -> -32 -> -22

36 -> 27 -> -32 -> -29

36 -> 27 -> -32 -> -30

36 -> 27 -> -32 -> -34

36 -> 27 -> -32 -> -35

36 -> 27 -> -32 -> -37

36 -> 27 -> -32 -> -38

36 -> 27 -> -32 -> -39

36 -> 27 -> -32 -> -43

36 -> 27 -> -32 -> -55

36 -> 27 -> -32 -> -56

36 -> 27 -> -32 -> -62

36 -> 27 -> -32 -> -63

36 -> 27 -> -32 -> -64

36 -> 27 -> -32 -> -68

36 -> 27 -> -32 -> -71

36 -> 27 -> -32 -> -73

36 -> 27 -> -32 -> -78

36 -> 27 -> -32 -> -81

36 -> 27 -> -32 -> -86

36 -> 27 -> -32 -> -92

36 -> 27 -> -32 -> -94

36 -> 27 -> -32 -> -95

36 -> 27 -> -32 -> -98

36 -> 27 -> -32 -> -99

36 -> 27 -> -32 -> -101

36 -> 27 -> -32 -> -103

36 -> 27 -> -32 -> -105

36 -> 27 -> -32 -> -106

36 -> 27 -> -32 -> -107

36 -> 27 -> -32 -> -113

36 -> 27 -> -32 -> -117

36 -> 27 -> -32 -> -124

36 -> 27 -> -32 -> -125

36 -> 27 -> -32 -> -133

36 -> 27 -> -32 -> -137

36 -> 27 -> -32 -> -144

36 -> 27 -> -32 -> -145

36 -> 27 -> -32 -> -146

36 -> 27 -> -32 -> -147

36 -> 27 -> -32 -> -150

36 -> 27 -> -32 -> -154

36 -> 27 -> -32 -> -156

36 -> 27 -> -62

36 -> 27 -> -62 -> -1

36 -> 27 -> -62 -> -3

36 -> 27 -> -62 -> -4

36 -> 27 -> -62 -> -5

36 -> 27 -> -62 -> -6

36 -> 27 -> -62 -> -7

36 -> 27 -> -62 -> -8

36 -> 27 -> -62 -> -9

36 -> 27 -> -62 -> -10

36 -> 27 -> -62 -> -17

36 -> 27 -> -62 -> -18

36 -> 27 -> -62 -> -19

36 -> 27 -> -62 -> -20

36 -> 27 -> -62 -> -22

36 -> 27 -> -62 -> -24

36 -> 27 -> -62 -> -26

36 -> 27 -> -62 -> -27

36 -> 27 -> -62 -> -28

36 -> 27 -> -62 -> -29

36 -> 27 -> -62 -> -30

36 -> 27 -> -62 -> -31

36 -> 27 -> -62 -> -32

36 -> 27 -> -62 -> -33

36 -> 27 -> -62 -> -35

36 -> 27 -> -62 -> -36

36 -> 27 -> -62 -> -39

36 -> 27 -> -62 -> -42

36 -> 27 -> -62 -> -43

36 -> 27 -> -62 -> -44

36 -> 27 -> -62 -> -45

36 -> 27 -> -62 -> -46

36 -> 27 -> -62 -> -47

36 -> 27 -> -62 -> -48

36 -> 27 -> -62 -> -49

36 -> 27 -> -62 -> -55

36 -> 27 -> -62 -> -56

36 -> 27 -> -62 -> -58

36 -> 27 -> -62 -> -60

36 -> 27 -> -62 -> -61

36 -> 27 -> -62 -> -63

36 -> 27 -> -62 -> -64

36 -> 27 -> -62 -> -66

36 -> 27 -> -62 -> -68

36 -> 27 -> -62 -> -69

36 -> 27 -> -62 -> -71

36 -> 27 -> -62 -> -73

36 -> 27 -> -62 -> -77

36 -> 27 -> -62 -> -78

36 -> 27 -> -62 -> -81

36 -> 27 -> -62 -> -83

36 -> 27 -> -62 -> -84

36 -> 27 -> -62 -> -86

36 -> 27 -> -62 -> -88

36 -> 27 -> -62 -> -89

36 -> 27 -> -62 -> -91

36 -> 27 -> -62 -> -92

36 -> 27 -> -62 -> -93

36 -> 27 -> -62 -> -95

36 -> 27 -> -62 -> -96

36 -> 27 -> -62 -> -97

36 -> 27 -> -62 -> -98

36 -> 27 -> -62 -> -99

36 -> 27 -> -62 -> -101

36 -> 27 -> -62 -> -105

36 -> 27 -> -62 -> -106

36 -> 27 -> -62 -> -107

36 -> 27 -> -62 -> -110

36 -> 27 -> -62 -> -113

36 -> 27 -> -62 -> -114

36 -> 27 -> -62 -> -116

36 -> 27 -> -62 -> -117

36 -> 27 -> -62 -> -118

36 -> 27 -> -62 -> -119

36 -> 27 -> -62 -> -120

36 -> 27 -> -62 -> -121

36 -> 27 -> -62 -> -122

36 -> 27 -> -62 -> -124

36 -> 27 -> -62 -> -130

36 -> 27 -> -62 -> -131

36 -> 27 -> -62 -> -132

36 -> 27 -> -62 -> -133

36 -> 27 -> -62 -> -135

36 -> 27 -> -62 -> -136

36 -> 27 -> -62 -> -137

36 -> 27 -> -62 -> -141

36 -> 27 -> -62 -> -144

36 -> 27 -> -62 -> -145

36 -> 27 -> -62 -> -146

36 -> 27 -> -62 -> -147

36 -> 27 -> -62 -> -150

36 -> 27 -> -62 -> -154

36 -> 27 -> -62 -> -155

36 -> 27 -> -62 -> -156

36 -> 27 -> -68

36 -> 27 -> -68 -> -1

36 -> 27 -> -68 -> -2

36 -> 27 -> -68 -> -3

36 -> 27 -> -68 -> -17

36 -> 27 -> -68 -> -19

36 -> 27 -> -68 -> -20

36 -> 27 -> -68 -> -22

36 -> 27 -> -68 -> -26

36 -> 27 -> -68 -> -27

36 -> 27 -> -68 -> -28

36 -> 27 -> -68 -> -29

36 -> 27 -> -68 -> -32

36 -> 27 -> -68 -> -39

36 -> 27 -> -68 -> -41

36 -> 27 -> -68 -> -43

36 -> 27 -> -68 -> -56

36 -> 27 -> -68 -> -61

36 -> 27 -> -68 -> -62

36 -> 27 -> -68 -> -63

36 -> 27 -> -68 -> -71

36 -> 27 -> -68 -> -73

36 -> 27 -> -68 -> -78

36 -> 27 -> -68 -> -80

36 -> 27 -> -68 -> -81

36 -> 27 -> -68 -> -86

36 -> 27 -> -68 -> -88

36 -> 27 -> -68 -> -92

36 -> 27 -> -68 -> -98

36 -> 27 -> -68 -> -99

36 -> 27 -> -68 -> -101

36 -> 27 -> -68 -> -106

36 -> 27 -> -68 -> -107

36 -> 27 -> -68 -> -109

36 -> 27 -> -68 -> -111

36 -> 27 -> -68 -> -113

36 -> 27 -> -68 -> -117

36 -> 27 -> -68 -> -118

36 -> 27 -> -68 -> -119

36 -> 27 -> -68 -> -121

36 -> 27 -> -68 -> -122

36 -> 27 -> -68 -> -124

36 -> 27 -> -68 -> -128

36 -> 27 -> -68 -> -129

36 -> 27 -> -68 -> -133

36 -> 27 -> -68 -> -137

36 -> 27 -> -68 -> -142

36 -> 27 -> -68 -> -144

36 -> 27 -> -68 -> -145

36 -> 27 -> -68 -> -146

36 -> 27 -> -68 -> -147

36 -> 27 -> -68 -> -150

36 -> 27 -> -68 -> -151

36 -> 27 -> -68 -> -156

36 -> 27 -> -73

36 -> 27 -> -73 -> -1

36 -> 27 -> -73 -> -3

36 -> 27 -> -73 -> -6

36 -> 27 -> -73 -> -17

36 -> 27 -> -73 -> -19

36 -> 27 -> -73 -> -20

36 -> 27 -> -73 -> -22

36 -> 27 -> -73 -> -26

36 -> 27 -> -73 -> -27

36 -> 27 -> -73 -> -28

36 -> 27 -> -73 -> -29

36 -> 27 -> -73 -> -30

36 -> 27 -> -73 -> -32

36 -> 27 -> -73 -> -35

36 -> 27 -> -73 -> -39

36 -> 27 -> -73 -> -43

36 -> 27 -> -73 -> -48

36 -> 27 -> -73 -> -56

36 -> 27 -> -73 -> -61

36 -> 27 -> -73 -> -62

36 -> 27 -> -73 -> -63

36 -> 27 -> -73 -> -64

36 -> 27 -> -73 -> -68

36 -> 27 -> -73 -> -71

36 -> 27 -> -73 -> -78

36 -> 27 -> -73 -> -81

36 -> 27 -> -73 -> -83

36 -> 27 -> -73 -> -86

36 -> 27 -> -73 -> -88

36 -> 27 -> -73 -> -89

36 -> 27 -> -73 -> -92

36 -> 27 -> -73 -> -95

36 -> 27 -> -73 -> -98

36 -> 27 -> -73 -> -99

36 -> 27 -> -73 -> -101

36 -> 27 -> -73 -> -106

36 -> 27 -> -73 -> -107

36 -> 27 -> -73 -> -113

36 -> 27 -> -73 -> -114

36 -> 27 -> -73 -> -117

36 -> 27 -> -73 -> -118

36 -> 27 -> -73 -> -119

36 -> 27 -> -73 -> -121

36 -> 27 -> -73 -> -122

36 -> 27 -> -73 -> -124

36 -> 27 -> -73 -> -133

36 -> 27 -> -73 -> -137

36 -> 27 -> -73 -> -144

36 -> 27 -> -73 -> -145

36 -> 27 -> -73 -> -146

36 -> 27 -> -73 -> -147

36 -> 27 -> -73 -> -150

36 -> 27 -> -73 -> -154

36 -> 27 -> -73 -> -155

36 -> 27 -> -73 -> -156

36 -> 27 -> -81

36 -> 27 -> -81 -> -1

36 -> 27 -> -81 -> -3

36 -> 27 -> -81 -> -6

36 -> 27 -> -81 -> -7

36 -> 27 -> -81 -> -17

36 -> 27 -> -81 -> -18

36 -> 27 -> -81 -> -19

36 -> 27 -> -81 -> -20

36 -> 27 -> -81 -> -22

36 -> 27 -> -81 -> -26

36 -> 27 -> -81 -> -27

36 -> 27 -> -81 -> -28

36 -> 27 -> -81 -> -29

36 -> 27 -> -81 -> -30

36 -> 27 -> -81 -> -32

36 -> 27 -> -81 -> -35

36 -> 27 -> -81 -> -36

36 -> 27 -> -81 -> -39

36 -> 27 -> -81 -> -42

36 -> 27 -> -81 -> -43

36 -> 27 -> -81 -> -48

36 -> 27 -> -81 -> -51

36 -> 27 -> -81 -> -55

36 -> 27 -> -81 -> -56

36 -> 27 -> -81 -> -61

36 -> 27 -> -81 -> -62

36 -> 27 -> -81 -> -63

36 -> 27 -> -81 -> -64

36 -> 27 -> -81 -> -68

36 -> 27 -> -81 -> -71

36 -> 27 -> -81 -> -73

36 -> 27 -> -81 -> -78

36 -> 27 -> -81 -> -83

36 -> 27 -> -81 -> -86

36 -> 27 -> -81 -> -88

36 -> 27 -> -81 -> -89

36 -> 27 -> -81 -> -92

36 -> 27 -> -81 -> -95

36 -> 27 -> -81 -> -97

36 -> 27 -> -81 -> -98

36 -> 27 -> -81 -> -99

36 -> 27 -> -81 -> -100

36 -> 27 -> -81 -> -101

36 -> 27 -> -81 -> -105

36 -> 27 -> -81 -> -106

36 -> 27 -> -81 -> -107

36 -> 27 -> -81 -> -110

36 -> 27 -> -81 -> -113

36 -> 27 -> -81 -> -114

36 -> 27 -> -81 -> -116

36 -> 27 -> -81 -> -117

36 -> 27 -> -81 -> -118

36 -> 27 -> -81 -> -119

36 -> 27 -> -81 -> -121

36 -> 27 -> -81 -> -122

36 -> 27 -> -81 -> -124

36 -> 27 -> -81 -> -127

36 -> 27 -> -81 -> -130

36 -> 27 -> -81 -> -133

36 -> 27 -> -81 -> -134

36 -> 27 -> -81 -> -137

36 -> 27 -> -81 -> -144

36 -> 27 -> -81 -> -145

36 -> 27 -> -81 -> -146

36 -> 27 -> -81 -> -147

36 -> 27 -> -81 -> -150

36 -> 27 -> -81 -> -154

36 -> 27 -> -81 -> -155

36 -> 27 -> -81 -> -156

36 -> 27 -> -98

36 -> 27 -> -98 -> -1

36 -> 27 -> -98 -> -3

36 -> 27 -> -98 -> -4

36 -> 27 -> -98 -> -6

36 -> 27 -> -98 -> -7

36 -> 27 -> -98 -> -8

36 -> 27 -> -98 -> -9

36 -> 27 -> -98 -> -10

36 -> 27 -> -98 -> -17

36 -> 27 -> -98 -> -19

36 -> 27 -> -98 -> -20

36 -> 27 -> -98 -> -22

36 -> 27 -> -98 -> -24

36 -> 27 -> -98 -> -26

36 -> 27 -> -98 -> -27

36 -> 27 -> -98 -> -28

36 -> 27 -> -98 -> -29

36 -> 27 -> -98 -> -30

36 -> 27 -> -98 -> -31

36 -> 27 -> -98 -> -32

36 -> 27 -> -98 -> -33

36 -> 27 -> -98 -> -35

36 -> 27 -> -98 -> -36

36 -> 27 -> -98 -> -39

36 -> 27 -> -98 -> -42

36 -> 27 -> -98 -> -43

36 -> 27 -> -98 -> -44

36 -> 27 -> -98 -> -45

36 -> 27 -> -98 -> -46

36 -> 27 -> -98 -> -47

36 -> 27 -> -98 -> -48

36 -> 27 -> -98 -> -49

36 -> 27 -> -98 -> -51

36 -> 27 -> -98 -> -55

36 -> 27 -> -98 -> -56

36 -> 27 -> -98 -> -58

36 -> 27 -> -98 -> -60

36 -> 27 -> -98 -> -61

36 -> 27 -> -98 -> -62

36 -> 27 -> -98 -> -63

36 -> 27 -> -98 -> -64

36 -> 27 -> -98 -> -66

36 -> 27 -> -98 -> -68

36 -> 27 -> -98 -> -69

36 -> 27 -> -98 -> -71

36 -> 27 -> -98 -> -73

36 -> 27 -> -98 -> -77

36 -> 27 -> -98 -> -78

36 -> 27 -> -98 -> -81

36 -> 27 -> -98 -> -83

36 -> 27 -> -98 -> -84

36 -> 27 -> -98 -> -86

36 -> 27 -> -98 -> -88

36 -> 27 -> -98 -> -89

36 -> 27 -> -98 -> -91

36 -> 27 -> -98 -> -92

36 -> 27 -> -98 -> -95

36 -> 27 -> -98 -> -96

36 -> 27 -> -98 -> -99

36 -> 27 -> -98 -> -100

36 -> 27 -> -98 -> -101

36 -> 27 -> -98 -> -105

36 -> 27 -> -98 -> -106

36 -> 27 -> -98 -> -107

36 -> 27 -> -98 -> -110

36 -> 27 -> -98 -> -113

36 -> 27 -> -98 -> -114

36 -> 27 -> -98 -> -117

36 -> 27 -> -98 -> -118

36 -> 27 -> -98 -> -119

36 -> 27 -> -98 -> -120

36 -> 27 -> -98 -> -121

36 -> 27 -> -98 -> -122

36 -> 27 -> -98 -> -124

36 -> 27 -> -98 -> -127

36 -> 27 -> -98 -> -130

36 -> 27 -> -98 -> -131

36 -> 27 -> -98 -> -132

36 -> 27 -> -98 -> -133

36 -> 27 -> -98 -> -135

36 -> 27 -> -98 -> -136

36 -> 27 -> -98 -> -137

36 -> 27 -> -98 -> -141

36 -> 27 -> -98 -> -144

36 -> 27 -> -98 -> -145

36 -> 27 -> -98 -> -146

36 -> 27 -> -98 -> -147

36 -> 27 -> -98 -> -150

36 -> 27 -> -98 -> -154

36 -> 27 -> -98 -> -155

36 -> 27 -> -98 -> -156

36 -> 27 -> -99

36 -> 27 -> -99 -> -1

36 -> 27 -> -99 -> -3

36 -> 27 -> -99 -> -6

36 -> 27 -> -99 -> -17

36 -> 27 -> -99 -> -18

36 -> 27 -> -99 -> -19

36 -> 27 -> -99 -> -20

36 -> 27 -> -99 -> -22

36 -> 27 -> -99 -> -26

36 -> 27 -> -99 -> -27

36 -> 27 -> -99 -> -28

36 -> 27 -> -99 -> -29

36 -> 27 -> -99 -> -30

36 -> 27 -> -99 -> -32

36 -> 27 -> -99 -> -35

36 -> 27 -> -99 -> -36

36 -> 27 -> -99 -> -39

36 -> 27 -> -99 -> -41

36 -> 27 -> -99 -> -43

36 -> 27 -> -99 -> -48

36 -> 27 -> -99 -> -51

36 -> 27 -> -99 -> -56

36 -> 27 -> -99 -> -61

36 -> 27 -> -99 -> -62

36 -> 27 -> -99 -> -63

36 -> 27 -> -99 -> -64

36 -> 27 -> -99 -> -68

36 -> 27 -> -99 -> -73

36 -> 27 -> -99 -> -78

36 -> 27 -> -99 -> -81

36 -> 27 -> -99 -> -83

36 -> 27 -> -99 -> -86

36 -> 27 -> -99 -> -88

36 -> 27 -> -99 -> -89

36 -> 27 -> -99 -> -92

36 -> 27 -> -99 -> -95

36 -> 27 -> -99 -> -97

36 -> 27 -> -99 -> -98

36 -> 27 -> -99 -> -100

36 -> 27 -> -99 -> -101

36 -> 27 -> -99 -> -106

36 -> 27 -> -99 -> -107

36 -> 27 -> -99 -> -109

36 -> 27 -> -99 -> -111

36 -> 27 -> -99 -> -114

36 -> 27 -> -99 -> -117

36 -> 27 -> -99 -> -118

36 -> 27 -> -99 -> -119

36 -> 27 -> -99 -> -121

36 -> 27 -> -99 -> -122

36 -> 27 -> -99 -> -124

36 -> 27 -> -99 -> -127

36 -> 27 -> -99 -> -133

36 -> 27 -> -99 -> -137

36 -> 27 -> -99 -> -144

36 -> 27 -> -99 -> -145

36 -> 27 -> -99 -> -146

36 -> 27 -> -99 -> -147

36 -> 27 -> -99 -> -150

36 -> 27 -> -99 -> -151

36 -> 27 -> -99 -> -154

36 -> 27 -> -99 -> -155

36 -> 27 -> -99 -> -156

36 -> 27 -> -101

36 -> 27 -> -101 -> -1

36 -> 27 -> -101 -> -3

36 -> 27 -> -101 -> -6

36 -> 27 -> -101 -> -17

36 -> 27 -> -101 -> -19

36 -> 27 -> -101 -> -20

36 -> 27 -> -101 -> -22

36 -> 27 -> -101 -> -26

36 -> 27 -> -101 -> -28

36 -> 27 -> -101 -> -29

36 -> 27 -> -101 -> -30

36 -> 27 -> -101 -> -32

36 -> 27 -> -101 -> -35

36 -> 27 -> -101 -> -36

36 -> 27 -> -101 -> -39

36 -> 27 -> -101 -> -41

36 -> 27 -> -101 -> -43

36 -> 27 -> -101 -> -48

36 -> 27 -> -101 -> -51

36 -> 27 -> -101 -> -56

36 -> 27 -> -101 -> -61

36 -> 27 -> -101 -> -62

36 -> 27 -> -101 -> -63

36 -> 27 -> -101 -> -64

36 -> 27 -> -101 -> -68

36 -> 27 -> -101 -> -73

36 -> 27 -> -101 -> -74

36 -> 27 -> -101 -> -75

36 -> 27 -> -101 -> -78

36 -> 27 -> -101 -> -81

36 -> 27 -> -101 -> -83

36 -> 27 -> -101 -> -86

36 -> 27 -> -101 -> -89

36 -> 27 -> -101 -> -92

36 -> 27 -> -101 -> -95

36 -> 27 -> -101 -> -98

36 -> 27 -> -101 -> -99

36 -> 27 -> -101 -> -100

36 -> 27 -> -101 -> -106

36 -> 27 -> -101 -> -107

36 -> 27 -> -101 -> -109

36 -> 27 -> -101 -> -111

36 -> 27 -> -101 -> -114

36 -> 27 -> -101 -> -117

36 -> 27 -> -101 -> -118

36 -> 27 -> -101 -> -119

36 -> 27 -> -101 -> -121

36 -> 27 -> -101 -> -122

36 -> 27 -> -101 -> -124

36 -> 27 -> -101 -> -125

36 -> 27 -> -101 -> -127

36 -> 27 -> -101 -> -133

36 -> 27 -> -101 -> -137

36 -> 27 -> -101 -> -144

36 -> 27 -> -101 -> -145

36 -> 27 -> -101 -> -146

36 -> 27 -> -101 -> -147

36 -> 27 -> -101 -> -150

36 -> 27 -> -101 -> -151

36 -> 27 -> -101 -> -154

36 -> 27 -> -101 -> -155

36 -> 27 -> -101 -> -156

36 -> 27 -> -107 -> -1

36 -> 27 -> -107 -> -3

36 -> 27 -> -107 -> -4

36 -> 27 -> -107 -> -5

36 -> 27 -> -107 -> -6

36 -> 27 -> -107 -> -7

36 -> 27 -> -107 -> -8

36 -> 27 -> -107 -> -9

36 -> 27 -> -107 -> -10

36 -> 27 -> -107 -> -17

36 -> 27 -> -107 -> -18

36 -> 27 -> -107 -> -19

36 -> 27 -> -107 -> -20

36 -> 27 -> -107 -> -22

36 -> 27 -> -107 -> -24

36 -> 27 -> -107 -> -26

36 -> 27 -> -107 -> -27

36 -> 27 -> -107 -> -28

36 -> 27 -> -107 -> -29

36 -> 27 -> -107 -> -30

36 -> 27 -> -107 -> -31

36 -> 27 -> -107 -> -32

36 -> 27 -> -107 -> -33

36 -> 27 -> -107 -> -35

36 -> 27 -> -107 -> -36

36 -> 27 -> -107 -> -39

36 -> 27 -> -107 -> -42

36 -> 27 -> -107 -> -43

36 -> 27 -> -107 -> -44

36 -> 27 -> -107 -> -45

36 -> 27 -> -107 -> -46

36 -> 27 -> -107 -> -47

36 -> 27 -> -107 -> -48

36 -> 27 -> -107 -> -49

36 -> 27 -> -107 -> -55

36 -> 27 -> -107 -> -56

36 -> 27 -> -107 -> -58

36 -> 27 -> -107 -> -60

36 -> 27 -> -107 -> -61

36 -> 27 -> -107 -> -62

36 -> 27 -> -107 -> -63

36 -> 27 -> -107 -> -64

36 -> 27 -> -107 -> -66

36 -> 27 -> -107 -> -68

36 -> 27 -> -107 -> -69

36 -> 27 -> -107 -> -71

36 -> 27 -> -107 -> -73

36 -> 27 -> -107 -> -77

36 -> 27 -> -107 -> -78

36 -> 27 -> -107 -> -81

36 -> 27 -> -107 -> -83

36 -> 27 -> -107 -> -84

36 -> 27 -> -107 -> -86

36 -> 27 -> -107 -> -88

36 -> 27 -> -107 -> -89

36 -> 27 -> -107 -> -91

36 -> 27 -> -107 -> -92

36 -> 27 -> -107 -> -93

36 -> 27 -> -107 -> -95

36 -> 27 -> -107 -> -96

36 -> 27 -> -107 -> -97

36 -> 27 -> -107 -> -98

36 -> 27 -> -107 -> -99

36 -> 27 -> -107 -> -101

36 -> 27 -> -107 -> -105

36 -> 27 -> -107 -> -106

36 -> 27 -> -107 -> -110

36 -> 27 -> -107 -> -113

36 -> 27 -> -107 -> -114

36 -> 27 -> -107 -> -116

36 -> 27 -> -107 -> -117

36 -> 27 -> -107 -> -118

36 -> 27 -> -107 -> -119

36 -> 27 -> -107 -> -120

36 -> 27 -> -107 -> -121

36 -> 27 -> -107 -> -122

36 -> 27 -> -107 -> -124

36 -> 27 -> -107 -> -130

36 -> 27 -> -107 -> -131

36 -> 27 -> -107 -> -132

36 -> 27 -> -107 -> -133

36 -> 27 -> -107 -> -135

36 -> 27 -> -107 -> -136

36 -> 27 -> -107 -> -137

36 -> 27 -> -107 -> -141

36 -> 27 -> -107 -> -144

36 -> 27 -> -107 -> -145

36 -> 27 -> -107 -> -146

36 -> 27 -> -107 -> -147

36 -> 27 -> -107 -> -150

36 -> 27 -> -107 -> -154

36 -> 27 -> -107 -> -155

36 -> 27 -> -107 -> -156

36 -> 27 -> -124 -> -1

36 -> 27 -> -124 -> -3

36 -> 27 -> -124 -> -4

36 -> 27 -> -124 -> -5

36 -> 27 -> -124 -> -6

36 -> 27 -> -124 -> -7

36 -> 27 -> -124 -> -8

36 -> 27 -> -124 -> -9

36 -> 27 -> -124 -> -10

36 -> 27 -> -124 -> -17

36 -> 27 -> -124 -> -18

36 -> 27 -> -124 -> -19

36 -> 27 -> -124 -> -20

36 -> 27 -> -124 -> -22

36 -> 27 -> -124 -> -24

36 -> 27 -> -124 -> -26

36 -> 27 -> -124 -> -27

36 -> 27 -> -124 -> -28

36 -> 27 -> -124 -> -29

36 -> 27 -> -124 -> -30

36 -> 27 -> -124 -> -31

36 -> 27 -> -124 -> -32

36 -> 27 -> -124 -> -33

36 -> 27 -> -124 -> -35

36 -> 27 -> -124 -> -36

36 -> 27 -> -124 -> -39

36 -> 27 -> -124 -> -42

36 -> 27 -> -124 -> -43

36 -> 27 -> -124 -> -44

36 -> 27 -> -124 -> -45

36 -> 27 -> -124 -> -46

36 -> 27 -> -124 -> -47

36 -> 27 -> -124 -> -48

36 -> 27 -> -124 -> -49

36 -> 27 -> -124 -> -55

36 -> 27 -> -124 -> -56

36 -> 27 -> -124 -> -58

36 -> 27 -> -124 -> -60

36 -> 27 -> -124 -> -61

36 -> 27 -> -124 -> -62

36 -> 27 -> -124 -> -63

36 -> 27 -> -124 -> -64

36 -> 27 -> -124 -> -66

36 -> 27 -> -124 -> -68

36 -> 27 -> -124 -> -69

36 -> 27 -> -124 -> -71

36 -> 27 -> -124 -> -73

36 -> 27 -> -124 -> -77

36 -> 27 -> -124 -> -78

36 -> 27 -> -124 -> -81

36 -> 27 -> -124 -> -83

36 -> 27 -> -124 -> -84

36 -> 27 -> -124 -> -86

36 -> 27 -> -124 -> -88

36 -> 27 -> -124 -> -89

36 -> 27 -> -124 -> -91

36 -> 27 -> -124 -> -92

36 -> 27 -> -124 -> -93

36 -> 27 -> -124 -> -95

36 -> 27 -> -124 -> -96

36 -> 27 -> -124 -> -97

36 -> 27 -> -124 -> -98

36 -> 27 -> -124 -> -99

36 -> 27 -> -124 -> -101

36 -> 27 -> -124 -> -105

36 -> 27 -> -124 -> -106

36 -> 27 -> -124 -> -107

36 -> 27 -> -124 -> -110

36 -> 27 -> -124 -> -113

36 -> 27 -> -124 -> -114

36 -> 27 -> -124 -> -116

36 -> 27 -> -124 -> -117

36 -> 27 -> -124 -> -118

36 -> 27 -> -124 -> -119

36 -> 27 -> -124 -> -120

36 -> 27 -> -124 -> -121

36 -> 27 -> -124 -> -122

36 -> 27 -> -124 -> -130

36 -> 27 -> -124 -> -131

36 -> 27 -> -124 -> -132

36 -> 27 -> -124 -> -133

36 -> 27 -> -124 -> -135

36 -> 27 -> -124 -> -136

36 -> 27 -> -124 -> -137

36 -> 27 -> -124 -> -141

36 -> 27 -> -124 -> -144

36 -> 27 -> -124 -> -145

36 -> 27 -> -124 -> -146

36 -> 27 -> -124 -> -147

36 -> 27 -> -124 -> -150

36 -> 27 -> -124 -> -154

36 -> 27 -> -124 -> -155

36 -> 27 -> -124 -> -156

36 -> 27 -> -133

36 -> 27 -> -133 -> -1

36 -> 27 -> -133 -> -3

36 -> 27 -> -133 -> -19

36 -> 27 -> -133 -> -20

36 -> 27 -> -133 -> -22

36 -> 27 -> -133 -> -26

36 -> 27 -> -133 -> -27

36 -> 27 -> -133 -> -28

36 -> 27 -> -133 -> -29

36 -> 27 -> -133 -> -32

36 -> 27 -> -133 -> -36

36 -> 27 -> -133 -> -39

36 -> 27 -> -133 -> -40

36 -> 27 -> -133 -> -48

36 -> 27 -> -133 -> -56

36 -> 27 -> -133 -> -61

36 -> 27 -> -133 -> -62

36 -> 27 -> -133 -> -63

36 -> 27 -> -133 -> -64

36 -> 27 -> -133 -> -68

36 -> 27 -> -133 -> -73

36 -> 27 -> -133 -> -78

36 -> 27 -> -133 -> -79

36 -> 27 -> -133 -> -81

36 -> 27 -> -133 -> -86

36 -> 27 -> -133 -> -94

36 -> 27 -> -133 -> -95

36 -> 27 -> -133 -> -98

36 -> 27 -> -133 -> -99

36 -> 27 -> -133 -> -101

36 -> 27 -> -133 -> -106

36 -> 27 -> -133 -> -107

36 -> 27 -> -133 -> -114

36 -> 27 -> -133 -> -117

36 -> 27 -> -133 -> -118

36 -> 27 -> -133 -> -119

36 -> 27 -> -133 -> -121

36 -> 27 -> -133 -> -122

36 -> 27 -> -133 -> -123

36 -> 27 -> -133 -> -124

36 -> 27 -> -133 -> -125

36 -> 27 -> -133 -> -137

36 -> 27 -> -133 -> -145

36 -> 27 -> -133 -> -146

36 -> 27 -> -133 -> -147

36 -> 27 -> -133 -> -150

36 -> 27 -> -133 -> -154

36 -> 27 -> -133 -> -156

36 -> 27 -> -145

36 -> 27 -> -145 -> -1

36 -> 27 -> -145 -> -3

36 -> 27 -> -145 -> -12

36 -> 27 -> -145 -> -19

36 -> 27 -> -145 -> -20

36 -> 27 -> -145 -> -22

36 -> 27 -> -145 -> -26

36 -> 27 -> -145 -> -27

36 -> 27 -> -145 -> -28

36 -> 27 -> -145 -> -29

36 -> 27 -> -145 -> -32

36 -> 27 -> -145 -> -37

36 -> 27 -> -145 -> -39

36 -> 27 -> -145 -> -56

36 -> 27 -> -145 -> -61

36 -> 27 -> -145 -> -62

36 -> 27 -> -145 -> -63

36 -> 27 -> -145 -> -68

36 -> 27 -> -145 -> -71

36 -> 27 -> -145 -> -73

36 -> 27 -> -145 -> -78

36 -> 27 -> -145 -> -81

36 -> 27 -> -145 -> -86

36 -> 27 -> -145 -> -98

36 -> 27 -> -145 -> -99

36 -> 27 -> -145 -> -101

36 -> 27 -> -145 -> -103

36 -> 27 -> -145 -> -106

36 -> 27 -> -145 -> -107

36 -> 27 -> -145 -> -113

36 -> 27 -> -145 -> -117

36 -> 27 -> -145 -> -118

36 -> 27 -> -145 -> -119

36 -> 27 -> -145 -> -121

36 -> 27 -> -145 -> -122

36 -> 27 -> -145 -> -124

36 -> 27 -> -145 -> -133

36 -> 27 -> -145 -> -137

36 -> 27 -> -145 -> -147

36 -> 27 -> -145 -> -150

36 -> 27 -> -145 -> -156

36 -> 29 -> 3 -> -32

36 -> 29 -> 3 -> -42

36 -> 29 -> 4 -> -101

36 -> 29 -> 7 -> -54

36 -> 29 -> 9 -> -6

36 -> 29 -> 14 -> -54

36 -> 29 -> 15 -> -68

36 -> 29 -> 19 -> -73

36 -> 29 -> 21 -> -73

36 -> 29 -> 21 -> -101

36 -> 29 -> 21 -> -154

36 -> 29 -> 22 -> -152

36 -> 29 -> 24 -> -49

36 -> 29 -> 25 -> -132

36 -> 29 -> 27 -> -19

36 -> 29 -> 27 -> -20

36 -> 29 -> 27 -> -32

36 -> 29 -> 27 -> -62

36 -> 29 -> 27 -> -68

36 -> 29 -> 27 -> -73

36 -> 29 -> 27 -> -81

36 -> 29 -> 27 -> -98

36 -> 29 -> 27 -> -99

36 -> 29 -> 27 -> -101

36 -> 29 -> 27 -> -107

36 -> 29 -> 27 -> -124

36 -> 29 -> 27 -> -133

36 -> 29 -> 27 -> -145

36 -> 29 -> 28 -> -81

36 -> 29 -> 30 -> -1

36 -> 29 -> 30 -> -3

36 -> 29 -> 30 -> -32

36 -> 29 -> 30 -> -39

36 -> 29 -> 30 -> -63

36 -> 29 -> 30 -> -73

36 -> 29 -> 30 -> -81

36 -> 29 -> 30 -> -99

36 -> 29 -> 30 -> -101

36 -> 29 -> 30 -> -106

36 -> 29 -> 30 -> -117

36 -> 29 -> 30 -> -133

36 -> 29 -> 30 -> -137

36 -> 29 -> 30 -> -150

36 -> 29 -> 30 -> -156

36 -> 29 -> 32 -> -9

36 -> 29 -> 33 -> -145

36 -> 29 -> 38 -> -55

36 -> 29 -> 38 -> -56

36 -> 29 -> 38 -> -105

36 -> 29 -> 39 -> -101

36 -> 29 -> 40 -> -73

36 -> 29 -> 41 -> -6

36 -> 29 -> 42 -> -133

36 -> 29 -> 44 -> -29

36 -> 29 -> 44 -> -48

36 -> 29 -> 44 -> -81

36 -> 29 -> 44 -> -89

36 -> 29 -> 44 -> -99

36 -> 29 -> 44 -> -114

36 -> 29 -> 45 -> -73

36 -> 29 -> 48 -> -99

36 -> 29 -> 49 -> -15

36 -> 29 -> 55 -> -12

36 -> 29 -> 60 -> -64

36 -> 29 -> 64 -> -10

36 -> 29 -> 65 -> -4

36 -> 29 -> 66 -> -136

36 -> 29 -> 67 -> -135

36 -> 29 -> 68 -> -73

36 -> 29 -> 69 -> -77

36 -> 29 -> 73 -> -32

36 -> 29 -> 74 -> -112

36 -> 29 -> 75 -> -1

36 -> 29 -> 75 -> -29

36 -> 29 -> 76 -> -81

36 -> 29 -> 77 -> -73

36 -> 29 -> 79 -> -73

36 -> 29 -> 84 -> -73

36 -> 29 -> 87 -> -73

36 -> 29 -> 88 -> -83

36 -> 29 -> 89 -> -6

36 -> 29 -> 89 -> -73

36 -> 29 -> 89 -> -81

36 -> 29 -> 89 -> -83

36 -> 29 -> 89 -> -86

36 -> 29 -> 89 -> -99

36 -> 29 -> 89 -> -101

36 -> 29 -> 89 -> -114

36 -> 29 -> 89 -> -155

36 -> 29 -> 91 -> -101

36 -> 29 -> 92 -> -133

36 -> 29 -> 97 -> -15

36 -> 29 -> 103 -> -78

36 -> 29 -> 105 -> -32

36 -> 29 -> 105 -> -146

36 -> 29 -> 107 -> -73

36 -> 29 -> 107 -> -99

36 -> 29 -> 108 -> -80

36 -> 29 -> 110 -> -56

36 -> 29 -> 113 -> -73

36 -> 29 -> 116 -> -73

36 -> 29 -> 118 -> -73

36 -> 29 -> 120 -> -133

36 -> 29 -> 122 -> -56

36 -> 29 -> 123 -> -73

36 -> 29 -> 128 -> -32

36 -> 29 -> 128 -> -95

36 -> 29 -> 129 -> -32

36 -> 29 -> 130 -> -73

36 -> 29 -> 134 -> -99

36 -> 29 -> 136 -> -20

36 -> 29 -> 139 -> -15

36 -> 29 -> 142 -> -154

36 -> 29 -> 146 -> -20

36 -> 29 -> 148 -> -32

36 -> 29 -> 149 -> -73

36 -> 29 -> 150 -> -91

36 -> 29 -> 153 -> -2

36 -> 29 -> 160 -> -129

36 -> 29 -> 161 -> -78

36 -> 29 -> 162 -> -133

36 -> 29 -> 163 -> -8

36 -> 29 -> 170 -> -138

36 -> 29 -> 171 -> -13

36 -> 29 -> 172 -> -58

36 -> 29 -> 176 -> -33

36 -> 29 -> 177 -> -106

36 -> 29 -> 179 -> -22

36 -> 29 -> 179 -> -147

36 -> 29 -> 180 -> -20

36 -> 29 -> 181 -> -84

36 -> 29 -> 186 -> -99

36 -> 29 -> 188 -> -24

36 -> 29 -> 189 -> -73

36 -> 29 -> -133

36 -> 29 -> -133 -> -1

36 -> 29 -> -133 -> -3

36 -> 29 -> -133 -> -19

36 -> 29 -> -133 -> -20

36 -> 29 -> -133 -> -22

36 -> 29 -> -133 -> -26

36 -> 29 -> -133 -> -27

36 -> 29 -> -133 -> -28

36 -> 29 -> -133 -> -29

36 -> 29 -> -133 -> -32

36 -> 29 -> -133 -> -36

36 -> 29 -> -133 -> -39

36 -> 29 -> -133 -> -40

36 -> 29 -> -133 -> -48

36 -> 29 -> -133 -> -56

36 -> 29 -> -133 -> -61

36 -> 29 -> -133 -> -62

36 -> 29 -> -133 -> -63

36 -> 29 -> -133 -> -64

36 -> 29 -> -133 -> -68

36 -> 29 -> -133 -> -73

36 -> 29 -> -133 -> -78

36 -> 29 -> -133 -> -79

36 -> 29 -> -133 -> -81

36 -> 29 -> -133 -> -86

36 -> 29 -> -133 -> -94

36 -> 29 -> -133 -> -95

36 -> 29 -> -133 -> -98

36 -> 29 -> -133 -> -99

36 -> 29 -> -133 -> -101

36 -> 29 -> -133 -> -106

36 -> 29 -> -133 -> -107

36 -> 29 -> -133 -> -114

36 -> 29 -> -133 -> -117

36 -> 29 -> -133 -> -118

36 -> 29 -> -133 -> -119

36 -> 29 -> -133 -> -121

36 -> 29 -> -133 -> -122

36 -> 29 -> -133 -> -123

36 -> 29 -> -133 -> -124

36 -> 29 -> -133 -> -125

36 -> 29 -> -133 -> -137

36 -> 29 -> -133 -> -145

36 -> 29 -> -133 -> -146

36 -> 29 -> -133 -> -147

36 -> 29 -> -133 -> -150

36 -> 29 -> -133 -> -154

36 -> 29 -> -133 -> -156

36 -> 30 -> 3 -> -32

36 -> 30 -> 3 -> -42

36 -> 30 -> 4 -> -101

36 -> 30 -> 6 -> -73

36 -> 30 -> 17 -> -32

36 -> 30 -> 21 -> -73

36 -> 30 -> 21 -> -101

36 -> 30 -> 21 -> -154

36 -> 30 -> 27 -> -19

36 -> 30 -> 27 -> -20

36 -> 30 -> 27 -> -32

36 -> 30 -> 27 -> -62

36 -> 30 -> 27 -> -68

36 -> 30 -> 27 -> -73

36 -> 30 -> 27 -> -81

36 -> 30 -> 27 -> -98

36 -> 30 -> 27 -> -99

36 -> 30 -> 27 -> -101

36 -> 30 -> 27 -> -107

36 -> 30 -> 27 -> -124

36 -> 30 -> 27 -> -133

36 -> 30 -> 27 -> -145

36 -> 30 -> 29 -> -133

36 -> 30 -> 37 -> -73

36 -> 30 -> 38 -> -55

36 -> 30 -> 38 -> -56

36 -> 30 -> 38 -> -105

36 -> 30 -> 39 -> -101

36 -> 30 -> 40 -> -73

36 -> 30 -> 43 -> -73

36 -> 30 -> 43 -> -99

36 -> 30 -> 43 -> -101

36 -> 30 -> 44 -> -29

36 -> 30 -> 44 -> -48

36 -> 30 -> 44 -> -81

36 -> 30 -> 44 -> -89

36 -> 30 -> 44 -> -99

36 -> 30 -> 44 -> -114

36 -> 30 -> 51 -> -2

36 -> 30 -> 60 -> -64

36 -> 30 -> 61 -> -27

36 -> 30 -> 62 -> -99

36 -> 30 -> 75 -> -1

36 -> 30 -> 75 -> -29

36 -> 30 -> 76 -> -81

36 -> 30 -> 77 -> -73

36 -> 30 -> 78 -> -73

36 -> 30 -> 81 -> -73

36 -> 30 -> 82 -> -30

36 -> 30 -> 82 -> -32

36 -> 30 -> 82 -> -35

36 -> 30 -> 82 -> -101

36 -> 30 -> 83 -> -125

36 -> 30 -> 89 -> -6

36 -> 30 -> 89 -> -73

36 -> 30 -> 89 -> -81

36 -> 30 -> 89 -> -83

36 -> 30 -> 89 -> -86

36 -> 30 -> 89 -> -99

36 -> 30 -> 89 -> -101

36 -> 30 -> 89 -> -114

36 -> 30 -> 89 -> -155

36 -> 30 -> 92 -> -133

36 -> 30 -> 101 -> -64

36 -> 30 -> 102 -> -116

36 -> 30 -> 103 -> -78

36 -> 30 -> 105 -> -32

36 -> 30 -> 105 -> -146

36 -> 30 -> 107 -> -73

36 -> 30 -> 107 -> -99

36 -> 30 -> 109 -> -20

36 -> 30 -> 109 -> -73

36 -> 30 -> 109 -> -99

36 -> 30 -> 109 -> -101

36 -> 30 -> 110 -> -56

36 -> 30 -> 114 -> -73

36 -> 30 -> 115 -> -17

36 -> 30 -> 115 -> -32

36 -> 30 -> 115 -> -68

36 -> 30 -> 115 -> -73

36 -> 30 -> 115 -> -92

36 -> 30 -> 117 -> -73

36 -> 30 -> 117 -> -101

36 -> 30 -> 122 -> -56

36 -> 30 -> 128 -> -32

36 -> 30 -> 128 -> -95

36 -> 30 -> 129 -> -32

36 -> 30 -> 133 -> -101

36 -> 30 -> 134 -> -99

36 -> 30 -> 136 -> -20

36 -> 30 -> 137 -> -18

36 -> 30 -> 137 -> -97

36 -> 30 -> 137 -> -99

36 -> 30 -> 138 -> -20

36 -> 30 -> 138 -> -61

36 -> 30 -> 138 -> -73

36 -> 30 -> 138 -> -98

36 -> 30 -> 138 -> -133

36 -> 30 -> 140 -> -99

36 -> 30 -> 140 -> -133

36 -> 30 -> 141 -> -51

36 -> 30 -> 141 -> -100

36 -> 30 -> 141 -> -127

36 -> 30 -> 144 -> -7

36 -> 30 -> 144 -> -110

36 -> 30 -> 144 -> -130

36 -> 30 -> 146 -> -20

36 -> 30 -> 151 -> -101

36 -> 30 -> 152 -> -36

36 -> 30 -> 157 -> -26

36 -> 30 -> 157 -> -28

36 -> 30 -> 157 -> -32

36 -> 30 -> 157 -> -68

36 -> 30 -> 157 -> -86

36 -> 30 -> 157 -> -99

36 -> 30 -> 157 -> -101

36 -> 30 -> 157 -> -118

36 -> 30 -> 157 -> -119

36 -> 30 -> 157 -> -120

36 -> 30 -> 157 -> -121

36 -> 30 -> 157 -> -122

36 -> 30 -> 157 -> -133

36 -> 30 -> 161 -> -78

36 -> 30 -> 164 -> -134

36 -> 30 -> 169 -> -99

36 -> 30 -> 173 -> -154

36 -> 30 -> 174 -> -114

36 -> 30 -> 179 -> -22

36 -> 30 -> 179 -> -147

36 -> 30 -> 180 -> -20

36 -> 30 -> 182 -> -73

36 -> 30 -> 186 -> -99

36 -> 30 -> 190 -> -43

36 -> 30 -> 190 -> -73

36 -> 30 -> 190 -> -86

36 -> 30 -> 190 -> -88

36 -> 30 -> 190 -> -144

36 -> 30 -> -1

36 -> 30 -> -1 -> -2

36 -> 30 -> -1 -> -3

36 -> 30 -> -1 -> -6

36 -> 30 -> -1 -> -12

36 -> 30 -> -1 -> -19

36 -> 30 -> -1 -> -20

36 -> 30 -> -1 -> -22

36 -> 30 -> -1 -> -26

36 -> 30 -> -1 -> -27

36 -> 30 -> -1 -> -28

36 -> 30 -> -1 -> -29

36 -> 30 -> -1 -> -30

36 -> 30 -> -1 -> -32

36 -> 30 -> -1 -> -35

36 -> 30 -> -1 -> -36

36 -> 30 -> -1 -> -39

36 -> 30 -> -1 -> -42

36 -> 30 -> -1 -> -48

36 -> 30 -> -1 -> -51

36 -> 30 -> -1 -> -55

36 -> 30 -> -1 -> -56

36 -> 30 -> -1 -> -62

36 -> 30 -> -1 -> -63

36 -> 30 -> -1 -> -64

36 -> 30 -> -1 -> -68

36 -> 30 -> -1 -> -71

36 -> 30 -> -1 -> -73

36 -> 30 -> -1 -> -76

36 -> 30 -> -1 -> -78

36 -> 30 -> -1 -> -80

36 -> 30 -> -1 -> -81

36 -> 30 -> -1 -> -83

36 -> 30 -> -1 -> -86

36 -> 30 -> -1 -> -88

36 -> 30 -> -1 -> -89

36 -> 30 -> -1 -> -95

36 -> 30 -> -1 -> -98

36 -> 30 -> -1 -> -99

36 -> 30 -> -1 -> -100

36 -> 30 -> -1 -> -101

36 -> 30 -> -1 -> -105

36 -> 30 -> -1 -> -106

36 -> 30 -> -1 -> -107

36 -> 30 -> -1 -> -112

36 -> 30 -> -1 -> -113

36 -> 30 -> -1 -> -114

36 -> 30 -> -1 -> -116

36 -> 30 -> -1 -> -117

36 -> 30 -> -1 -> -118

36 -> 30 -> -1 -> -119

36 -> 30 -> -1 -> -121

36 -> 30 -> -1 -> -122

36 -> 30 -> -1 -> -124

36 -> 30 -> -1 -> -127

36 -> 30 -> -1 -> -133

36 -> 30 -> -1 -> -137

36 -> 30 -> -1 -> -145

36 -> 30 -> -1 -> -146

36 -> 30 -> -1 -> -147

36 -> 30 -> -1 -> -150

36 -> 30 -> -1 -> -154

36 -> 30 -> -1 -> -155

36 -> 30 -> -1 -> -156

36 -> 30 -> -3 -> -1

36 -> 30 -> -3 -> -4

36 -> 30 -> -3 -> -6

36 -> 30 -> -3 -> -7

36 -> 30 -> -3 -> -8

36 -> 30 -> -3 -> -9

36 -> 30 -> -3 -> -10

36 -> 30 -> -3 -> -17

36 -> 30 -> -3 -> -18

36 -> 30 -> -3 -> -19

36 -> 30 -> -3 -> -20

36 -> 30 -> -3 -> -22

36 -> 30 -> -3 -> -24

36 -> 30 -> -3 -> -26

36 -> 30 -> -3 -> -27

36 -> 30 -> -3 -> -28

36 -> 30 -> -3 -> -29

36 -> 30 -> -3 -> -30

36 -> 30 -> -3 -> -31

36 -> 30 -> -3 -> -32

36 -> 30 -> -3 -> -33

36 -> 30 -> -3 -> -35

36 -> 30 -> -3 -> -36

36 -> 30 -> -3 -> -39

36 -> 30 -> -3 -> -42

36 -> 30 -> -3 -> -43

36 -> 30 -> -3 -> -44

36 -> 30 -> -3 -> -45

36 -> 30 -> -3 -> -46

36 -> 30 -> -3 -> -47

36 -> 30 -> -3 -> -48

36 -> 30 -> -3 -> -49

36 -> 30 -> -3 -> -51

36 -> 30 -> -3 -> -55

36 -> 30 -> -3 -> -56

36 -> 30 -> -3 -> -58

36 -> 30 -> -3 -> -60

36 -> 30 -> -3 -> -61

36 -> 30 -> -3 -> -62

36 -> 30 -> -3 -> -63

36 -> 30 -> -3 -> -64

36 -> 30 -> -3 -> -66

36 -> 30 -> -3 -> -68

36 -> 30 -> -3 -> -69

36 -> 30 -> -3 -> -73

36 -> 30 -> -3 -> -77

36 -> 30 -> -3 -> -78

36 -> 30 -> -3 -> -81

36 -> 30 -> -3 -> -83

36 -> 30 -> -3 -> -84

36 -> 30 -> -3 -> -86

36 -> 30 -> -3 -> -88

36 -> 30 -> -3 -> -89

36 -> 30 -> -3 -> -91

36 -> 30 -> -3 -> -92

36 -> 30 -> -3 -> -95

36 -> 30 -> -3 -> -96

36 -> 30 -> -3 -> -97

36 -> 30 -> -3 -> -98

36 -> 30 -> -3 -> -99

36 -> 30 -> -3 -> -100

36 -> 30 -> -3 -> -101

36 -> 30 -> -3 -> -105

36 -> 30 -> -3 -> -106

36 -> 30 -> -3 -> -107

36 -> 30 -> -3 -> -110

36 -> 30 -> -3 -> -114

36 -> 30 -> -3 -> -116

36 -> 30 -> -3 -> -117

36 -> 30 -> -3 -> -118

36 -> 30 -> -3 -> -119

36 -> 30 -> -3 -> -120

36 -> 30 -> -3 -> -121

36 -> 30 -> -3 -> -122

36 -> 30 -> -3 -> -124

36 -> 30 -> -3 -> -125

36 -> 30 -> -3 -> -127

36 -> 30 -> -3 -> -130

36 -> 30 -> -3 -> -131

36 -> 30 -> -3 -> -132

36 -> 30 -> -3 -> -133

36 -> 30 -> -3 -> -134

36 -> 30 -> -3 -> -135

36 -> 30 -> -3 -> -136

36 -> 30 -> -3 -> -137

36 -> 30 -> -3 -> -141

36 -> 30 -> -3 -> -144

36 -> 30 -> -3 -> -145

36 -> 30 -> -3 -> -146

36 -> 30 -> -3 -> -147

36 -> 30 -> -3 -> -150

36 -> 30 -> -3 -> -154

36 -> 30 -> -3 -> -155

36 -> 30 -> -3 -> -156

36 -> 30 -> -32

36 -> 30 -> -32 -> -1

36 -> 30 -> -32 -> -3

36 -> 30 -> -32 -> -14

36 -> 30 -> -32 -> -17

36 -> 30 -> -32 -> -19

36 -> 30 -> -32 -> -20

36 -> 30 -> -32 -> -22

36 -> 30 -> -32 -> -29

36 -> 30 -> -32 -> -30

36 -> 30 -> -32 -> -34

36 -> 30 -> -32 -> -35

36 -> 30 -> -32 -> -37

36 -> 30 -> -32 -> -38

36 -> 30 -> -32 -> -39

36 -> 30 -> -32 -> -43

36 -> 30 -> -32 -> -55

36 -> 30 -> -32 -> -56

36 -> 30 -> -32 -> -62

36 -> 30 -> -32 -> -63

36 -> 30 -> -32 -> -64

36 -> 30 -> -32 -> -68

36 -> 30 -> -32 -> -71

36 -> 30 -> -32 -> -73

36 -> 30 -> -32 -> -78

36 -> 30 -> -32 -> -81

36 -> 30 -> -32 -> -86

36 -> 30 -> -32 -> -92

36 -> 30 -> -32 -> -94

36 -> 30 -> -32 -> -95

36 -> 30 -> -32 -> -98

36 -> 30 -> -32 -> -99

36 -> 30 -> -32 -> -101

36 -> 30 -> -32 -> -103

36 -> 30 -> -32 -> -105

36 -> 30 -> -32 -> -106

36 -> 30 -> -32 -> -107

36 -> 30 -> -32 -> -113

36 -> 30 -> -32 -> -117

36 -> 30 -> -32 -> -124

36 -> 30 -> -32 -> -125

36 -> 30 -> -32 -> -133

36 -> 30 -> -32 -> -137

36 -> 30 -> -32 -> -144

36 -> 30 -> -32 -> -145

36 -> 30 -> -32 -> -146

36 -> 30 -> -32 -> -147

36 -> 30 -> -32 -> -150

36 -> 30 -> -32 -> -154

36 -> 30 -> -32 -> -156

36 -> 30 -> -39 -> -1

36 -> 30 -> -39 -> -3

36 -> 30 -> -39 -> -4

36 -> 30 -> -39 -> -6

36 -> 30 -> -39 -> -7

36 -> 30 -> -39 -> -8

36 -> 30 -> -39 -> -9

36 -> 30 -> -39 -> -10

36 -> 30 -> -39 -> -17

36 -> 30 -> -39 -> -18

36 -> 30 -> -39 -> -19

36 -> 30 -> -39 -> -20

36 -> 30 -> -39 -> -22

36 -> 30 -> -39 -> -24

36 -> 30 -> -39 -> -26

36 -> 30 -> -39 -> -27

36 -> 30 -> -39 -> -28

36 -> 30 -> -39 -> -29

36 -> 30 -> -39 -> -30

36 -> 30 -> -39 -> -31

36 -> 30 -> -39 -> -32

36 -> 30 -> -39 -> -33

36 -> 30 -> -39 -> -35

36 -> 30 -> -39 -> -36

36 -> 30 -> -39 -> -42

36 -> 30 -> -39 -> -43

36 -> 30 -> -39 -> -44

36 -> 30 -> -39 -> -45

36 -> 30 -> -39 -> -46

36 -> 30 -> -39 -> -47

36 -> 30 -> -39 -> -48

36 -> 30 -> -39 -> -49

36 -> 30 -> -39 -> -51

36 -> 30 -> -39 -> -55

36 -> 30 -> -39 -> -56

36 -> 30 -> -39 -> -58

36 -> 30 -> -39 -> -60

36 -> 30 -> -39 -> -61

36 -> 30 -> -39 -> -62

36 -> 30 -> -39 -> -63

36 -> 30 -> -39 -> -64

36 -> 30 -> -39 -> -66

36 -> 30 -> -39 -> -68

36 -> 30 -> -39 -> -69

36 -> 30 -> -39 -> -73

36 -> 30 -> -39 -> -77

36 -> 30 -> -39 -> -78

36 -> 30 -> -39 -> -81

36 -> 30 -> -39 -> -83

36 -> 30 -> -39 -> -84

36 -> 30 -> -39 -> -86

36 -> 30 -> -39 -> -88

36 -> 30 -> -39 -> -89

36 -> 30 -> -39 -> -91

36 -> 30 -> -39 -> -92

36 -> 30 -> -39 -> -95

36 -> 30 -> -39 -> -96

36 -> 30 -> -39 -> -97

36 -> 30 -> -39 -> -98

36 -> 30 -> -39 -> -99

36 -> 30 -> -39 -> -100

36 -> 30 -> -39 -> -101

36 -> 30 -> -39 -> -105

36 -> 30 -> -39 -> -106

36 -> 30 -> -39 -> -107

36 -> 30 -> -39 -> -110

36 -> 30 -> -39 -> -114

36 -> 30 -> -39 -> -116

36 -> 30 -> -39 -> -117

36 -> 30 -> -39 -> -118

36 -> 30 -> -39 -> -119

36 -> 30 -> -39 -> -120

36 -> 30 -> -39 -> -121

36 -> 30 -> -39 -> -122

36 -> 30 -> -39 -> -124

36 -> 30 -> -39 -> -125

36 -> 30 -> -39 -> -127

36 -> 30 -> -39 -> -130

36 -> 30 -> -39 -> -131

36 -> 30 -> -39 -> -132

36 -> 30 -> -39 -> -133

36 -> 30 -> -39 -> -134

36 -> 30 -> -39 -> -135

36 -> 30 -> -39 -> -136

36 -> 30 -> -39 -> -137

36 -> 30 -> -39 -> -141

36 -> 30 -> -39 -> -144

36 -> 30 -> -39 -> -145

36 -> 30 -> -39 -> -146

36 -> 30 -> -39 -> -147

36 -> 30 -> -39 -> -150

36 -> 30 -> -39 -> -154

36 -> 30 -> -39 -> -155

36 -> 30 -> -39 -> -156

36 -> 30 -> -63 -> -1

36 -> 30 -> -63 -> -3

36 -> 30 -> -63 -> -4

36 -> 30 -> -63 -> -6

36 -> 30 -> -63 -> -7

36 -> 30 -> -63 -> -8

36 -> 30 -> -63 -> -9

36 -> 30 -> -63 -> -10

36 -> 30 -> -63 -> -17

36 -> 30 -> -63 -> -18

36 -> 30 -> -63 -> -19

36 -> 30 -> -63 -> -20

36 -> 30 -> -63 -> -22

36 -> 30 -> -63 -> -24

36 -> 30 -> -63 -> -26

36 -> 30 -> -63 -> -27

36 -> 30 -> -63 -> -28

36 -> 30 -> -63 -> -29

36 -> 30 -> -63 -> -30

36 -> 30 -> -63 -> -31

36 -> 30 -> -63 -> -32

36 -> 30 -> -63 -> -33

36 -> 30 -> -63 -> -35

36 -> 30 -> -63 -> -36

36 -> 30 -> -63 -> -39

36 -> 30 -> -63 -> -42

36 -> 30 -> -63 -> -43

36 -> 30 -> -63 -> -44

36 -> 30 -> -63 -> -45

36 -> 30 -> -63 -> -46

36 -> 30 -> -63 -> -47

36 -> 30 -> -63 -> -48

36 -> 30 -> -63 -> -49

36 -> 30 -> -63 -> -51

36 -> 30 -> -63 -> -55

36 -> 30 -> -63 -> -56

36 -> 30 -> -63 -> -58

36 -> 30 -> -63 -> -60

36 -> 30 -> -63 -> -61

36 -> 30 -> -63 -> -62

36 -> 30 -> -63 -> -64

36 -> 30 -> -63 -> -66

36 -> 30 -> -63 -> -68

36 -> 30 -> -63 -> -69

36 -> 30 -> -63 -> -73

36 -> 30 -> -63 -> -77

36 -> 30 -> -63 -> -78

36 -> 30 -> -63 -> -81

36 -> 30 -> -63 -> -83

36 -> 30 -> -63 -> -84

36 -> 30 -> -63 -> -86

36 -> 30 -> -63 -> -88

36 -> 30 -> -63 -> -89

36 -> 30 -> -63 -> -91

36 -> 30 -> -63 -> -92

36 -> 30 -> -63 -> -95

36 -> 30 -> -63 -> -96

36 -> 30 -> -63 -> -97

36 -> 30 -> -63 -> -98

36 -> 30 -> -63 -> -99

36 -> 30 -> -63 -> -100

36 -> 30 -> -63 -> -101

36 -> 30 -> -63 -> -105

36 -> 30 -> -63 -> -106

36 -> 30 -> -63 -> -107

36 -> 30 -> -63 -> -110

36 -> 30 -> -63 -> -114

36 -> 30 -> -63 -> -116

36 -> 30 -> -63 -> -117

36 -> 30 -> -63 -> -118

36 -> 30 -> -63 -> -119

36 -> 30 -> -63 -> -120

36 -> 30 -> -63 -> -121

36 -> 30 -> -63 -> -122

36 -> 30 -> -63 -> -124

36 -> 30 -> -63 -> -125

36 -> 30 -> -63 -> -127

36 -> 30 -> -63 -> -130

36 -> 30 -> -63 -> -131

36 -> 30 -> -63 -> -132

36 -> 30 -> -63 -> -133

36 -> 30 -> -63 -> -134

36 -> 30 -> -63 -> -135

36 -> 30 -> -63 -> -136

36 -> 30 -> -63 -> -137

36 -> 30 -> -63 -> -141

36 -> 30 -> -63 -> -144

36 -> 30 -> -63 -> -145

36 -> 30 -> -63 -> -146

36 -> 30 -> -63 -> -147

36 -> 30 -> -63 -> -150

36 -> 30 -> -63 -> -154

36 -> 30 -> -63 -> -155

36 -> 30 -> -63 -> -156

36 -> 30 -> -73

36 -> 30 -> -73 -> -1

36 -> 30 -> -73 -> -3

36 -> 30 -> -73 -> -6

36 -> 30 -> -73 -> -17

36 -> 30 -> -73 -> -19

36 -> 30 -> -73 -> -20

36 -> 30 -> -73 -> -22

36 -> 30 -> -73 -> -26

36 -> 30 -> -73 -> -27

36 -> 30 -> -73 -> -28

36 -> 30 -> -73 -> -29

36 -> 30 -> -73 -> -30

36 -> 30 -> -73 -> -32

36 -> 30 -> -73 -> -35

36 -> 30 -> -73 -> -39

36 -> 30 -> -73 -> -43

36 -> 30 -> -73 -> -48

36 -> 30 -> -73 -> -56

36 -> 30 -> -73 -> -61

36 -> 30 -> -73 -> -62

36 -> 30 -> -73 -> -63

36 -> 30 -> -73 -> -64

36 -> 30 -> -73 -> -68

36 -> 30 -> -73 -> -71

36 -> 30 -> -73 -> -78

36 -> 30 -> -73 -> -81

36 -> 30 -> -73 -> -83

36 -> 30 -> -73 -> -86

36 -> 30 -> -73 -> -88

36 -> 30 -> -73 -> -89

36 -> 30 -> -73 -> -92

36 -> 30 -> -73 -> -95

36 -> 30 -> -73 -> -98

36 -> 30 -> -73 -> -99

36 -> 30 -> -73 -> -101

36 -> 30 -> -73 -> -106

36 -> 30 -> -73 -> -107

36 -> 30 -> -73 -> -113

36 -> 30 -> -73 -> -114

36 -> 30 -> -73 -> -117

36 -> 30 -> -73 -> -118

36 -> 30 -> -73 -> -119

36 -> 30 -> -73 -> -121

36 -> 30 -> -73 -> -122

36 -> 30 -> -73 -> -124

36 -> 30 -> -73 -> -133

36 -> 30 -> -73 -> -137

36 -> 30 -> -73 -> -144

36 -> 30 -> -73 -> -145

36 -> 30 -> -73 -> -146

36 -> 30 -> -73 -> -147

36 -> 30 -> -73 -> -150

36 -> 30 -> -73 -> -154

36 -> 30 -> -73 -> -155

36 -> 30 -> -73 -> -156

36 -> 30 -> -81

36 -> 30 -> -81 -> -1

36 -> 30 -> -81 -> -3

36 -> 30 -> -81 -> -6

36 -> 30 -> -81 -> -7

36 -> 30 -> -81 -> -17

36 -> 30 -> -81 -> -18

36 -> 30 -> -81 -> -19

36 -> 30 -> -81 -> -20

36 -> 30 -> -81 -> -22

36 -> 30 -> -81 -> -26

36 -> 30 -> -81 -> -27

36 -> 30 -> -81 -> -28

36 -> 30 -> -81 -> -29

36 -> 30 -> -81 -> -30

36 -> 30 -> -81 -> -32

36 -> 30 -> -81 -> -35

36 -> 30 -> -81 -> -36

36 -> 30 -> -81 -> -39

36 -> 30 -> -81 -> -42

36 -> 30 -> -81 -> -43

36 -> 30 -> -81 -> -48

36 -> 30 -> -81 -> -51

36 -> 30 -> -81 -> -55

36 -> 30 -> -81 -> -56

36 -> 30 -> -81 -> -61

36 -> 30 -> -81 -> -62

36 -> 30 -> -81 -> -63

36 -> 30 -> -81 -> -64

36 -> 30 -> -81 -> -68

36 -> 30 -> -81 -> -71

36 -> 30 -> -81 -> -73

36 -> 30 -> -81 -> -78

36 -> 30 -> -81 -> -83

36 -> 30 -> -81 -> -86

36 -> 30 -> -81 -> -88

36 -> 30 -> -81 -> -89

36 -> 30 -> -81 -> -92

36 -> 30 -> -81 -> -95

36 -> 30 -> -81 -> -97

36 -> 30 -> -81 -> -98

36 -> 30 -> -81 -> -99

36 -> 30 -> -81 -> -100

36 -> 30 -> -81 -> -101

36 -> 30 -> -81 -> -105

36 -> 30 -> -81 -> -106

36 -> 30 -> -81 -> -107

36 -> 30 -> -81 -> -110

36 -> 30 -> -81 -> -113

36 -> 30 -> -81 -> -114

36 -> 30 -> -81 -> -116

36 -> 30 -> -81 -> -117

36 -> 30 -> -81 -> -118

36 -> 30 -> -81 -> -119

36 -> 30 -> -81 -> -121

36 -> 30 -> -81 -> -122

36 -> 30 -> -81 -> -124

36 -> 30 -> -81 -> -127

36 -> 30 -> -81 -> -130

36 -> 30 -> -81 -> -133

36 -> 30 -> -81 -> -134

36 -> 30 -> -81 -> -137

36 -> 30 -> -81 -> -144

36 -> 30 -> -81 -> -145

36 -> 30 -> -81 -> -146

36 -> 30 -> -81 -> -147

36 -> 30 -> -81 -> -150

36 -> 30 -> -81 -> -154

36 -> 30 -> -81 -> -155

36 -> 30 -> -81 -> -156

36 -> 30 -> -99

36 -> 30 -> -99 -> -1

36 -> 30 -> -99 -> -3

36 -> 30 -> -99 -> -6

36 -> 30 -> -99 -> -17

36 -> 30 -> -99 -> -18

36 -> 30 -> -99 -> -19

36 -> 30 -> -99 -> -20

36 -> 30 -> -99 -> -22

36 -> 30 -> -99 -> -26

36 -> 30 -> -99 -> -27

36 -> 30 -> -99 -> -28

36 -> 30 -> -99 -> -29

36 -> 30 -> -99 -> -30

36 -> 30 -> -99 -> -32

36 -> 30 -> -99 -> -35

36 -> 30 -> -99 -> -36

36 -> 30 -> -99 -> -39

36 -> 30 -> -99 -> -41

36 -> 30 -> -99 -> -43

36 -> 30 -> -99 -> -48

36 -> 30 -> -99 -> -51

36 -> 30 -> -99 -> -56

36 -> 30 -> -99 -> -61

36 -> 30 -> -99 -> -62

36 -> 30 -> -99 -> -63

36 -> 30 -> -99 -> -64

36 -> 30 -> -99 -> -68

36 -> 30 -> -99 -> -73

36 -> 30 -> -99 -> -78

36 -> 30 -> -99 -> -81

36 -> 30 -> -99 -> -83

36 -> 30 -> -99 -> -86

36 -> 30 -> -99 -> -88

36 -> 30 -> -99 -> -89

36 -> 30 -> -99 -> -92

36 -> 30 -> -99 -> -95

36 -> 30 -> -99 -> -97

36 -> 30 -> -99 -> -98

36 -> 30 -> -99 -> -100

36 -> 30 -> -99 -> -101

36 -> 30 -> -99 -> -106

36 -> 30 -> -99 -> -107

36 -> 30 -> -99 -> -109

36 -> 30 -> -99 -> -111

36 -> 30 -> -99 -> -114

36 -> 30 -> -99 -> -117

36 -> 30 -> -99 -> -118

36 -> 30 -> -99 -> -119

36 -> 30 -> -99 -> -121

36 -> 30 -> -99 -> -122

36 -> 30 -> -99 -> -124

36 -> 30 -> -99 -> -127

36 -> 30 -> -99 -> -133

36 -> 30 -> -99 -> -137

36 -> 30 -> -99 -> -144

36 -> 30 -> -99 -> -145

36 -> 30 -> -99 -> -146

36 -> 30 -> -99 -> -147

36 -> 30 -> -99 -> -150

36 -> 30 -> -99 -> -151

36 -> 30 -> -99 -> -154

36 -> 30 -> -99 -> -155

36 -> 30 -> -99 -> -156

36 -> 30 -> -101

36 -> 30 -> -101 -> -1

36 -> 30 -> -101 -> -3

36 -> 30 -> -101 -> -6

36 -> 30 -> -101 -> -17

36 -> 30 -> -101 -> -19

36 -> 30 -> -101 -> -20

36 -> 30 -> -101 -> -22

36 -> 30 -> -101 -> -26

36 -> 30 -> -101 -> -28

36 -> 30 -> -101 -> -29

36 -> 30 -> -101 -> -30

36 -> 30 -> -101 -> -32

36 -> 30 -> -101 -> -35

36 -> 30 -> -101 -> -36

36 -> 30 -> -101 -> -39

36 -> 30 -> -101 -> -41

36 -> 30 -> -101 -> -43

36 -> 30 -> -101 -> -48

36 -> 30 -> -101 -> -51

36 -> 30 -> -101 -> -56

36 -> 30 -> -101 -> -61

36 -> 30 -> -101 -> -62

36 -> 30 -> -101 -> -63

36 -> 30 -> -101 -> -64

36 -> 30 -> -101 -> -68

36 -> 30 -> -101 -> -73

36 -> 30 -> -101 -> -74

36 -> 30 -> -101 -> -75

36 -> 30 -> -101 -> -78

36 -> 30 -> -101 -> -81

36 -> 30 -> -101 -> -83

36 -> 30 -> -101 -> -86

36 -> 30 -> -101 -> -89

36 -> 30 -> -101 -> -92

36 -> 30 -> -101 -> -95

36 -> 30 -> -101 -> -98

36 -> 30 -> -101 -> -99

36 -> 30 -> -101 -> -100

36 -> 30 -> -101 -> -106

36 -> 30 -> -101 -> -107

36 -> 30 -> -101 -> -109

36 -> 30 -> -101 -> -111

36 -> 30 -> -101 -> -114

36 -> 30 -> -101 -> -117

36 -> 30 -> -101 -> -118

36 -> 30 -> -101 -> -119

36 -> 30 -> -101 -> -121

36 -> 30 -> -101 -> -122

36 -> 30 -> -101 -> -124

36 -> 30 -> -101 -> -125

36 -> 30 -> -101 -> -127

36 -> 30 -> -101 -> -133

36 -> 30 -> -101 -> -137

36 -> 30 -> -101 -> -144

36 -> 30 -> -101 -> -145

36 -> 30 -> -101 -> -146

36 -> 30 -> -101 -> -147

36 -> 30 -> -101 -> -150

36 -> 30 -> -101 -> -151

36 -> 30 -> -101 -> -154

36 -> 30 -> -101 -> -155

36 -> 30 -> -101 -> -156

36 -> 30 -> -106

36 -> 30 -> -106 -> -1

36 -> 30 -> -106 -> -3

36 -> 30 -> -106 -> -6

36 -> 30 -> -106 -> -18

36 -> 30 -> -106 -> -19

36 -> 30 -> -106 -> -20

36 -> 30 -> -106 -> -22

36 -> 30 -> -106 -> -27

36 -> 30 -> -106 -> -29

36 -> 30 -> -106 -> -30

36 -> 30 -> -106 -> -32

36 -> 30 -> -106 -> -35

36 -> 30 -> -106 -> -36

36 -> 30 -> -106 -> -39

36 -> 30 -> -106 -> -42

36 -> 30 -> -106 -> -48

36 -> 30 -> -106 -> -51

36 -> 30 -> -106 -> -55

36 -> 30 -> -106 -> -56

36 -> 30 -> -106 -> -62

36 -> 30 -> -106 -> -63

36 -> 30 -> -106 -> -64

36 -> 30 -> -106 -> -68

36 -> 30 -> -106 -> -73

36 -> 30 -> -106 -> -78

36 -> 30 -> -106 -> -81

36 -> 30 -> -106 -> -83

36 -> 30 -> -106 -> -86

36 -> 30 -> -106 -> -88

36 -> 30 -> -106 -> -89

36 -> 30 -> -106 -> -95

36 -> 30 -> -106 -> -97

36 -> 30 -> -106 -> -98

36 -> 30 -> -106 -> -99

36 -> 30 -> -106 -> -100

36 -> 30 -> -106 -> -101

36 -> 30 -> -106 -> -105

36 -> 30 -> -106 -> -107

36 -> 30 -> -106 -> -114

36 -> 30 -> -106 -> -117

36 -> 30 -> -106 -> -124

36 -> 30 -> -106 -> -127

36 -> 30 -> -106 -> -133

36 -> 30 -> -106 -> -134

36 -> 30 -> -106 -> -137

36 -> 30 -> -106 -> -142

36 -> 30 -> -106 -> -145

36 -> 30 -> -106 -> -146

36 -> 30 -> -106 -> -147

36 -> 30 -> -106 -> -150

36 -> 30 -> -106 -> -154

36 -> 30 -> -106 -> -155

36 -> 30 -> -106 -> -156

36 -> 30 -> -117 -> -1

36 -> 30 -> -117 -> -3

36 -> 30 -> -117 -> -4

36 -> 30 -> -117 -> -6

36 -> 30 -> -117 -> -7

36 -> 30 -> -117 -> -8

36 -> 30 -> -117 -> -9

36 -> 30 -> -117 -> -10

36 -> 30 -> -117 -> -17

36 -> 30 -> -117 -> -18

36 -> 30 -> -117 -> -19

36 -> 30 -> -117 -> -20

36 -> 30 -> -117 -> -22

36 -> 30 -> -117 -> -24

36 -> 30 -> -117 -> -26

36 -> 30 -> -117 -> -27

36 -> 30 -> -117 -> -28

36 -> 30 -> -117 -> -29

36 -> 30 -> -117 -> -30

36 -> 30 -> -117 -> -31

36 -> 30 -> -117 -> -32

36 -> 30 -> -117 -> -33

36 -> 30 -> -117 -> -35

36 -> 30 -> -117 -> -36

36 -> 30 -> -117 -> -39

36 -> 30 -> -117 -> -42

36 -> 30 -> -117 -> -43

36 -> 30 -> -117 -> -44

36 -> 30 -> -117 -> -45

36 -> 30 -> -117 -> -46

36 -> 30 -> -117 -> -47

36 -> 30 -> -117 -> -48

36 -> 30 -> -117 -> -49

36 -> 30 -> -117 -> -51

36 -> 30 -> -117 -> -55

36 -> 30 -> -117 -> -56

36 -> 30 -> -117 -> -58

36 -> 30 -> -117 -> -60

36 -> 30 -> -117 -> -61

36 -> 30 -> -117 -> -62

36 -> 30 -> -117 -> -63

36 -> 30 -> -117 -> -64

36 -> 30 -> -117 -> -66

36 -> 30 -> -117 -> -68

36 -> 30 -> -117 -> -69

36 -> 30 -> -117 -> -73

36 -> 30 -> -117 -> -77

36 -> 30 -> -117 -> -78

36 -> 30 -> -117 -> -81

36 -> 30 -> -117 -> -83

36 -> 30 -> -117 -> -84

36 -> 30 -> -117 -> -86

36 -> 30 -> -117 -> -88

36 -> 30 -> -117 -> -89

36 -> 30 -> -117 -> -91

36 -> 30 -> -117 -> -92

36 -> 30 -> -117 -> -95

36 -> 30 -> -117 -> -96

36 -> 30 -> -117 -> -97

36 -> 30 -> -117 -> -98

36 -> 30 -> -117 -> -99

36 -> 30 -> -117 -> -100

36 -> 30 -> -117 -> -101

36 -> 30 -> -117 -> -105

36 -> 30 -> -117 -> -106

36 -> 30 -> -117 -> -107

36 -> 30 -> -117 -> -110

36 -> 30 -> -117 -> -114

36 -> 30 -> -117 -> -116

36 -> 30 -> -117 -> -118

36 -> 30 -> -117 -> -119

36 -> 30 -> -117 -> -120

36 -> 30 -> -117 -> -121

36 -> 30 -> -117 -> -122

36 -> 30 -> -117 -> -124

36 -> 30 -> -117 -> -125

36 -> 30 -> -117 -> -127

36 -> 30 -> -117 -> -130

36 -> 30 -> -117 -> -131

36 -> 30 -> -117 -> -132

36 -> 30 -> -117 -> -133

36 -> 30 -> -117 -> -134

36 -> 30 -> -117 -> -135

36 -> 30 -> -117 -> -136

36 -> 30 -> -117 -> -137

36 -> 30 -> -117 -> -141

36 -> 30 -> -117 -> -144

36 -> 30 -> -117 -> -145

36 -> 30 -> -117 -> -146

36 -> 30 -> -117 -> -147

36 -> 30 -> -117 -> -150

36 -> 30 -> -117 -> -154

36 -> 30 -> -117 -> -155

36 -> 30 -> -117 -> -156

36 -> 30 -> -133

36 -> 30 -> -133 -> -1

36 -> 30 -> -133 -> -3

36 -> 30 -> -133 -> -19

36 -> 30 -> -133 -> -20

36 -> 30 -> -133 -> -22

36 -> 30 -> -133 -> -26

36 -> 30 -> -133 -> -27

36 -> 30 -> -133 -> -28

36 -> 30 -> -133 -> -29

36 -> 30 -> -133 -> -32

36 -> 30 -> -133 -> -36

36 -> 30 -> -133 -> -39

36 -> 30 -> -133 -> -40

36 -> 30 -> -133 -> -48

36 -> 30 -> -133 -> -56

36 -> 30 -> -133 -> -61

36 -> 30 -> -133 -> -62

36 -> 30 -> -133 -> -63

36 -> 30 -> -133 -> -64

36 -> 30 -> -133 -> -68

36 -> 30 -> -133 -> -73

36 -> 30 -> -133 -> -78

36 -> 30 -> -133 -> -79

36 -> 30 -> -133 -> -81

36 -> 30 -> -133 -> -86

36 -> 30 -> -133 -> -94

36 -> 30 -> -133 -> -95

36 -> 30 -> -133 -> -98

36 -> 30 -> -133 -> -99

36 -> 30 -> -133 -> -101

36 -> 30 -> -133 -> -106

36 -> 30 -> -133 -> -107

36 -> 30 -> -133 -> -114

36 -> 30 -> -133 -> -117

36 -> 30 -> -133 -> -118

36 -> 30 -> -133 -> -119

36 -> 30 -> -133 -> -121

36 -> 30 -> -133 -> -122

36 -> 30 -> -133 -> -123

36 -> 30 -> -133 -> -124

36 -> 30 -> -133 -> -125

36 -> 30 -> -133 -> -137

36 -> 30 -> -133 -> -145

36 -> 30 -> -133 -> -146

36 -> 30 -> -133 -> -147

36 -> 30 -> -133 -> -150

36 -> 30 -> -133 -> -154

36 -> 30 -> -133 -> -156

36 -> 30 -> -137 -> -1

36 -> 30 -> -137 -> -3

36 -> 30 -> -137 -> -4

36 -> 30 -> -137 -> -6

36 -> 30 -> -137 -> -7

36 -> 30 -> -137 -> -8

36 -> 30 -> -137 -> -9

36 -> 30 -> -137 -> -10

36 -> 30 -> -137 -> -17

36 -> 30 -> -137 -> -18

36 -> 30 -> -137 -> -19

36 -> 30 -> -137 -> -20

36 -> 30 -> -137 -> -22

36 -> 30 -> -137 -> -24

36 -> 30 -> -137 -> -26

36 -> 30 -> -137 -> -27

36 -> 30 -> -137 -> -28

36 -> 30 -> -137 -> -29

36 -> 30 -> -137 -> -30

36 -> 30 -> -137 -> -31

36 -> 30 -> -137 -> -32

36 -> 30 -> -137 -> -33

36 -> 30 -> -137 -> -35

36 -> 30 -> -137 -> -36

36 -> 30 -> -137 -> -39

36 -> 30 -> -137 -> -42

36 -> 30 -> -137 -> -43

36 -> 30 -> -137 -> -44

36 -> 30 -> -137 -> -45

36 -> 30 -> -137 -> -46

36 -> 30 -> -137 -> -47

36 -> 30 -> -137 -> -48

36 -> 30 -> -137 -> -49

36 -> 30 -> -137 -> -51

36 -> 30 -> -137 -> -55

36 -> 30 -> -137 -> -56

36 -> 30 -> -137 -> -58

36 -> 30 -> -137 -> -60

36 -> 30 -> -137 -> -61

36 -> 30 -> -137 -> -62

36 -> 30 -> -137 -> -63

36 -> 30 -> -137 -> -64

36 -> 30 -> -137 -> -66

36 -> 30 -> -137 -> -68

36 -> 30 -> -137 -> -69

36 -> 30 -> -137 -> -73

36 -> 30 -> -137 -> -77

36 -> 30 -> -137 -> -78

36 -> 30 -> -137 -> -81

36 -> 30 -> -137 -> -83

36 -> 30 -> -137 -> -84

36 -> 30 -> -137 -> -86

36 -> 30 -> -137 -> -88

36 -> 30 -> -137 -> -89

36 -> 30 -> -137 -> -91

36 -> 30 -> -137 -> -92

36 -> 30 -> -137 -> -95

36 -> 30 -> -137 -> -96

36 -> 30 -> -137 -> -97

36 -> 30 -> -137 -> -98

36 -> 30 -> -137 -> -99

36 -> 30 -> -137 -> -100

36 -> 30 -> -137 -> -101

36 -> 30 -> -137 -> -105

36 -> 30 -> -137 -> -106

36 -> 30 -> -137 -> -107

36 -> 30 -> -137 -> -110

36 -> 30 -> -137 -> -114

36 -> 30 -> -137 -> -116

36 -> 30 -> -137 -> -117

36 -> 30 -> -137 -> -118

36 -> 30 -> -137 -> -119

36 -> 30 -> -137 -> -120

36 -> 30 -> -137 -> -121

36 -> 30 -> -137 -> -122

36 -> 30 -> -137 -> -124

36 -> 30 -> -137 -> -125

36 -> 30 -> -137 -> -127

36 -> 30 -> -137 -> -130

36 -> 30 -> -137 -> -131

36 -> 30 -> -137 -> -132

36 -> 30 -> -137 -> -133

36 -> 30 -> -137 -> -134

36 -> 30 -> -137 -> -135

36 -> 30 -> -137 -> -136

36 -> 30 -> -137 -> -141

36 -> 30 -> -137 -> -144

36 -> 30 -> -137 -> -145

36 -> 30 -> -137 -> -146

36 -> 30 -> -137 -> -147

36 -> 30 -> -137 -> -150

36 -> 30 -> -137 -> -154

36 -> 30 -> -137 -> -155

36 -> 30 -> -137 -> -156

36 -> 30 -> -150 -> -1

36 -> 30 -> -150 -> -3

36 -> 30 -> -150 -> -4

36 -> 30 -> -150 -> -6

36 -> 30 -> -150 -> -7

36 -> 30 -> -150 -> -8

36 -> 30 -> -150 -> -9

36 -> 30 -> -150 -> -10

36 -> 30 -> -150 -> -17

36 -> 30 -> -150 -> -18

36 -> 30 -> -150 -> -19

36 -> 30 -> -150 -> -20

36 -> 30 -> -150 -> -22

36 -> 30 -> -150 -> -24

36 -> 30 -> -150 -> -26

36 -> 30 -> -150 -> -27

36 -> 30 -> -150 -> -28

36 -> 30 -> -150 -> -29

36 -> 30 -> -150 -> -30

36 -> 30 -> -150 -> -31

36 -> 30 -> -150 -> -32

36 -> 30 -> -150 -> -33

36 -> 30 -> -150 -> -35

36 -> 30 -> -150 -> -36

36 -> 30 -> -150 -> -39

36 -> 30 -> -150 -> -42

36 -> 30 -> -150 -> -43

36 -> 30 -> -150 -> -44

36 -> 30 -> -150 -> -45

36 -> 30 -> -150 -> -46

36 -> 30 -> -150 -> -47

36 -> 30 -> -150 -> -48

36 -> 30 -> -150 -> -49

36 -> 30 -> -150 -> -51

36 -> 30 -> -150 -> -55

36 -> 30 -> -150 -> -56

36 -> 30 -> -150 -> -58

36 -> 30 -> -150 -> -60

36 -> 30 -> -150 -> -61

36 -> 30 -> -150 -> -62

36 -> 30 -> -150 -> -63

36 -> 30 -> -150 -> -64

36 -> 30 -> -150 -> -66

36 -> 30 -> -150 -> -68

36 -> 30 -> -150 -> -69

36 -> 30 -> -150 -> -73

36 -> 30 -> -150 -> -77

36 -> 30 -> -150 -> -78

36 -> 30 -> -150 -> -81

36 -> 30 -> -150 -> -83

36 -> 30 -> -150 -> -84

36 -> 30 -> -150 -> -86

36 -> 30 -> -150 -> -88

36 -> 30 -> -150 -> -89

36 -> 30 -> -150 -> -91

36 -> 30 -> -150 -> -92

36 -> 30 -> -150 -> -95

36 -> 30 -> -150 -> -96

36 -> 30 -> -150 -> -97

36 -> 30 -> -150 -> -98

36 -> 30 -> -150 -> -99

36 -> 30 -> -150 -> -100

36 -> 30 -> -150 -> -101

36 -> 30 -> -150 -> -105

36 -> 30 -> -150 -> -106

36 -> 30 -> -150 -> -107

36 -> 30 -> -150 -> -110

36 -> 30 -> -150 -> -114

36 -> 30 -> -150 -> -116

36 -> 30 -> -150 -> -117

36 -> 30 -> -150 -> -118

36 -> 30 -> -150 -> -119

36 -> 30 -> -150 -> -120

36 -> 30 -> -150 -> -121

36 -> 30 -> -150 -> -122

36 -> 30 -> -150 -> -124

36 -> 30 -> -150 -> -125

36 -> 30 -> -150 -> -127

36 -> 30 -> -150 -> -130

36 -> 30 -> -150 -> -131

36 -> 30 -> -150 -> -132

36 -> 30 -> -150 -> -133

36 -> 30 -> -150 -> -134

36 -> 30 -> -150 -> -135

36 -> 30 -> -150 -> -136

36 -> 30 -> -150 -> -137

36 -> 30 -> -150 -> -141

36 -> 30 -> -150 -> -144

36 -> 30 -> -150 -> -145

36 -> 30 -> -150 -> -146

36 -> 30 -> -150 -> -147

36 -> 30 -> -150 -> -154

36 -> 30 -> -150 -> -155

36 -> 30 -> -150 -> -156

36 -> 30 -> -156 -> -1

36 -> 30 -> -156 -> -3

36 -> 30 -> -156 -> -4

36 -> 30 -> -156 -> -6

36 -> 30 -> -156 -> -7

36 -> 30 -> -156 -> -8

36 -> 30 -> -156 -> -9

36 -> 30 -> -156 -> -10

36 -> 30 -> -156 -> -17

36 -> 30 -> -156 -> -18

36 -> 30 -> -156 -> -19

36 -> 30 -> -156 -> -20

36 -> 30 -> -156 -> -22

36 -> 30 -> -156 -> -24

36 -> 30 -> -156 -> -26

36 -> 30 -> -156 -> -27

36 -> 30 -> -156 -> -28

36 -> 30 -> -156 -> -29

36 -> 30 -> -156 -> -30

36 -> 30 -> -156 -> -31

36 -> 30 -> -156 -> -32

36 -> 30 -> -156 -> -33

36 -> 30 -> -156 -> -35

36 -> 30 -> -156 -> -36

36 -> 30 -> -156 -> -39

36 -> 30 -> -156 -> -42

36 -> 30 -> -156 -> -43

36 -> 30 -> -156 -> -44

36 -> 30 -> -156 -> -45

36 -> 30 -> -156 -> -46

36 -> 30 -> -156 -> -47

36 -> 30 -> -156 -> -48

36 -> 30 -> -156 -> -49

36 -> 30 -> -156 -> -51

36 -> 30 -> -156 -> -55

36 -> 30 -> -156 -> -56

36 -> 30 -> -156 -> -58

36 -> 30 -> -156 -> -60

36 -> 30 -> -156 -> -61

36 -> 30 -> -156 -> -62

36 -> 30 -> -156 -> -63

36 -> 30 -> -156 -> -64

36 -> 30 -> -156 -> -66

36 -> 30 -> -156 -> -68

36 -> 30 -> -156 -> -69

36 -> 30 -> -156 -> -73

36 -> 30 -> -156 -> -77

36 -> 30 -> -156 -> -78

36 -> 30 -> -156 -> -81

36 -> 30 -> -156 -> -83

36 -> 30 -> -156 -> -84

36 -> 30 -> -156 -> -86

36 -> 30 -> -156 -> -88

36 -> 30 -> -156 -> -89

36 -> 30 -> -156 -> -91

36 -> 30 -> -156 -> -92

36 -> 30 -> -156 -> -95

36 -> 30 -> -156 -> -96

36 -> 30 -> -156 -> -97

36 -> 30 -> -156 -> -98

36 -> 30 -> -156 -> -99

36 -> 30 -> -156 -> -100

36 -> 30 -> -156 -> -101

36 -> 30 -> -156 -> -105

36 -> 30 -> -156 -> -106

36 -> 30 -> -156 -> -107

36 -> 30 -> -156 -> -110

36 -> 30 -> -156 -> -114

36 -> 30 -> -156 -> -116

36 -> 30 -> -156 -> -117

36 -> 30 -> -156 -> -118

36 -> 30 -> -156 -> -119

36 -> 30 -> -156 -> -120

36 -> 30 -> -156 -> -121

36 -> 30 -> -156 -> -122

36 -> 30 -> -156 -> -124

36 -> 30 -> -156 -> -125

36 -> 30 -> -156 -> -127

36 -> 30 -> -156 -> -130

36 -> 30 -> -156 -> -131

36 -> 30 -> -156 -> -132

36 -> 30 -> -156 -> -133

36 -> 30 -> -156 -> -134

36 -> 30 -> -156 -> -135

36 -> 30 -> -156 -> -136

36 -> 30 -> -156 -> -137

36 -> 30 -> -156 -> -141

36 -> 30 -> -156 -> -144

36 -> 30 -> -156 -> -145

36 -> 30 -> -156 -> -146

36 -> 30 -> -156 -> -147

36 -> 30 -> -156 -> -150

36 -> 30 -> -156 -> -154

36 -> 30 -> -156 -> -155

36 -> 34 -> 2 -> -32

36 -> 34 -> 3 -> -32

36 -> 34 -> 3 -> -42

36 -> 34 -> 11 -> -25

36 -> 34 -> 11 -> -153

36 -> 34 -> 12 -> -32

36 -> 34 -> 13 -> -32

36 -> 34 -> 13 -> -73

36 -> 34 -> 16 -> -5

36 -> 34 -> 23 -> -23

36 -> 34 -> 23 -> -126

36 -> 34 -> 26 -> -14

36 -> 34 -> 27 -> -19

36 -> 34 -> 27 -> -20

36 -> 34 -> 27 -> -32

36 -> 34 -> 27 -> -62

36 -> 34 -> 27 -> -68

36 -> 34 -> 27 -> -73

36 -> 34 -> 27 -> -81

36 -> 34 -> 27 -> -98

36 -> 34 -> 27 -> -99

36 -> 34 -> 27 -> -101

36 -> 34 -> 27 -> -107

36 -> 34 -> 27 -> -124

36 -> 34 -> 27 -> -133

36 -> 34 -> 27 -> -145

36 -> 34 -> 31 -> -1

36 -> 34 -> 38 -> -55

36 -> 34 -> 38 -> -56

36 -> 34 -> 38 -> -105

36 -> 34 -> 40 -> -73

36 -> 34 -> 46 -> -32

36 -> 34 -> 47 -> -32

36 -> 34 -> 47 -> -37

36 -> 34 -> 52 -> -32

36 -> 34 -> 53 -> -133

36 -> 34 -> 58 -> -90

36 -> 34 -> 59 -> -148

36 -> 34 -> 61 -> -27

36 -> 34 -> 63 -> -32

36 -> 34 -> 70 -> -52

36 -> 34 -> 71 -> -32

36 -> 34 -> 72 -> -11

36 -> 34 -> 72 -> -67

36 -> 34 -> 73 -> -32

36 -> 34 -> 75 -> -1

36 -> 34 -> 75 -> -29

36 -> 34 -> 76 -> -81

36 -> 34 -> 80 -> -32

36 -> 34 -> 82 -> -30

36 -> 34 -> 82 -> -32

36 -> 34 -> 82 -> -35

36 -> 34 -> 82 -> -101

36 -> 34 -> 85 -> -12

36 -> 34 -> 93 -> -145

36 -> 34 -> 99 -> -73

36 -> 34 -> 102 -> -116

36 -> 34 -> 103 -> -78

36 -> 34 -> 107 -> -73

36 -> 34 -> 107 -> -99

36 -> 34 -> 109 -> -20

36 -> 34 -> 109 -> -73

36 -> 34 -> 109 -> -99

36 -> 34 -> 109 -> -101

36 -> 34 -> 110 -> -56

36 -> 34 -> 117 -> -73

36 -> 34 -> 117 -> -101

36 -> 34 -> 122 -> -56

36 -> 34 -> 124 -> -32

36 -> 34 -> 124 -> -103

36 -> 34 -> 125 -> -104

36 -> 34 -> 127 -> -93

36 -> 34 -> 129 -> -32

36 -> 34 -> 136 -> -20

36 -> 34 -> 147 -> -32

36 -> 34 -> 148 -> -32

36 -> 34 -> 152 -> -36

36 -> 34 -> 155 -> -73

36 -> 34 -> 158 -> -72

36 -> 34 -> 159 -> -80

36 -> 34 -> 159 -> -128

36 -> 34 -> 159 -> -129

36 -> 34 -> 175 -> -32

36 -> 34 -> 175 -> -34

36 -> 34 -> 178 -> -69

36 -> 34 -> 179 -> -22

36 -> 34 -> 179 -> -147

36 -> 34 -> 182 -> -73

36 -> 34 -> 184 -> -86

36 -> 34 -> 184 -> -101

36 -> 34 -> 184 -> -133

36 -> 34 -> 185 -> -32

36 -> 34 -> 185 -> -40

36 -> 34 -> 185 -> -79

36 -> 34 -> 185 -> -94

36 -> 34 -> 185 -> -123

36 -> 34 -> 185 -> -133

36 -> 34 -> 186 -> -99

36 -> 34 -> -32

36 -> 34 -> -32 -> -1

36 -> 34 -> -32 -> -3

36 -> 34 -> -32 -> -14

36 -> 34 -> -32 -> -17

36 -> 34 -> -32 -> -19

36 -> 34 -> -32 -> -20

36 -> 34 -> -32 -> -22

36 -> 34 -> -32 -> -29

36 -> 34 -> -32 -> -30

36 -> 34 -> -32 -> -34

36 -> 34 -> -32 -> -35

36 -> 34 -> -32 -> -37

36 -> 34 -> -32 -> -38

36 -> 34 -> -32 -> -39

36 -> 34 -> -32 -> -43

36 -> 34 -> -32 -> -55

36 -> 34 -> -32 -> -56

36 -> 34 -> -32 -> -62

36 -> 34 -> -32 -> -63

36 -> 34 -> -32 -> -64

36 -> 34 -> -32 -> -68

36 -> 34 -> -32 -> -71

36 -> 34 -> -32 -> -73

36 -> 34 -> -32 -> -78

36 -> 34 -> -32 -> -81

36 -> 34 -> -32 -> -86

36 -> 34 -> -32 -> -92

36 -> 34 -> -32 -> -94

36 -> 34 -> -32 -> -95

36 -> 34 -> -32 -> -98

36 -> 34 -> -32 -> -99

36 -> 34 -> -32 -> -101

36 -> 34 -> -32 -> -103

36 -> 34 -> -32 -> -105

36 -> 34 -> -32 -> -106

36 -> 34 -> -32 -> -107

36 -> 34 -> -32 -> -113

36 -> 34 -> -32 -> -117

36 -> 34 -> -32 -> -124

36 -> 34 -> -32 -> -125

36 -> 34 -> -32 -> -133

36 -> 34 -> -32 -> -137

36 -> 34 -> -32 -> -144

36 -> 34 -> -32 -> -145

36 -> 34 -> -32 -> -146

36 -> 34 -> -32 -> -147

36 -> 34 -> -32 -> -150

36 -> 34 -> -32 -> -154

36 -> 34 -> -32 -> -156

36 -> 34 -> -71 -> -1

36 -> 34 -> -71 -> -4

36 -> 34 -> -71 -> -5

36 -> 34 -> -71 -> -8

36 -> 34 -> -71 -> -9

36 -> 34 -> -71 -> -10

36 -> 34 -> -71 -> -11

36 -> 34 -> -71 -> -12

36 -> 34 -> -71 -> -14

36 -> 34 -> -71 -> -19

36 -> 34 -> -71 -> -22

36 -> 34 -> -71 -> -23

36 -> 34 -> -71 -> -24

36 -> 34 -> -71 -> -27

36 -> 34 -> -71 -> -30

36 -> 34 -> -71 -> -31

36 -> 34 -> -71 -> -32

36 -> 34 -> -71 -> -33

36 -> 34 -> -71 -> -34

36 -> 34 -> -71 -> -35

36 -> 34 -> -71 -> -36

36 -> 34 -> -71 -> -37

36 -> 34 -> -71 -> -38

36 -> 34 -> -71 -> -40

36 -> 34 -> -71 -> -42

36 -> 34 -> -71 -> -44

36 -> 34 -> -71 -> -45

36 -> 34 -> -71 -> -46

36 -> 34 -> -71 -> -47

36 -> 34 -> -71 -> -49

36 -> 34 -> -71 -> -52

36 -> 34 -> -71 -> -55

36 -> 34 -> -71 -> -56

36 -> 34 -> -71 -> -58

36 -> 34 -> -71 -> -60

36 -> 34 -> -71 -> -62

36 -> 34 -> -71 -> -66

36 -> 34 -> -71 -> -67

36 -> 34 -> -71 -> -68

36 -> 34 -> -71 -> -69

36 -> 34 -> -71 -> -72

36 -> 34 -> -71 -> -73

36 -> 34 -> -71 -> -77

36 -> 34 -> -71 -> -79

36 -> 34 -> -71 -> -80

36 -> 34 -> -71 -> -81

36 -> 34 -> -71 -> -84

36 -> 34 -> -71 -> -90

36 -> 34 -> -71 -> -91

36 -> 34 -> -71 -> -93

36 -> 34 -> -71 -> -96

36 -> 34 -> -71 -> -98

36 -> 34 -> -71 -> -103

36 -> 34 -> -71 -> -104

36 -> 34 -> -71 -> -105

36 -> 34 -> -71 -> -107

36 -> 34 -> -71 -> -113

36 -> 34 -> -71 -> -116

36 -> 34 -> -71 -> -120

36 -> 34 -> -71 -> -123

36 -> 34 -> -71 -> -124

36 -> 34 -> -71 -> -126

36 -> 34 -> -71 -> -128

36 -> 34 -> -71 -> -129

36 -> 34 -> -71 -> -131

36 -> 34 -> -71 -> -132

36 -> 34 -> -71 -> -135

36 -> 34 -> -71 -> -136

36 -> 34 -> -71 -> -141

36 -> 34 -> -71 -> -145

36 -> 34 -> -71 -> -147

36 -> 34 -> -71 -> -148

36 -> 34 -> -71 -> -153

36 -> 34 -> -113 -> -1

36 -> 34 -> -113 -> -4

36 -> 34 -> -113 -> -5

36 -> 34 -> -113 -> -8

36 -> 34 -> -113 -> -9

36 -> 34 -> -113 -> -10

36 -> 34 -> -113 -> -11

36 -> 34 -> -113 -> -12

36 -> 34 -> -113 -> -14

36 -> 34 -> -113 -> -19

36 -> 34 -> -113 -> -22

36 -> 34 -> -113 -> -23

36 -> 34 -> -113 -> -24

36 -> 34 -> -113 -> -27

36 -> 34 -> -113 -> -30

36 -> 34 -> -113 -> -31

36 -> 34 -> -113 -> -32

36 -> 34 -> -113 -> -33

36 -> 34 -> -113 -> -34

36 -> 34 -> -113 -> -35

36 -> 34 -> -113 -> -36

36 -> 34 -> -113 -> -37

36 -> 34 -> -113 -> -38

36 -> 34 -> -113 -> -40

36 -> 34 -> -113 -> -42

36 -> 34 -> -113 -> -44

36 -> 34 -> -113 -> -45

36 -> 34 -> -113 -> -46

36 -> 34 -> -113 -> -47

36 -> 34 -> -113 -> -49

36 -> 34 -> -113 -> -52

36 -> 34 -> -113 -> -55

36 -> 34 -> -113 -> -56

36 -> 34 -> -113 -> -58

36 -> 34 -> -113 -> -60

36 -> 34 -> -113 -> -62

36 -> 34 -> -113 -> -66

36 -> 34 -> -113 -> -67

36 -> 34 -> -113 -> -68

36 -> 34 -> -113 -> -69

36 -> 34 -> -113 -> -71

36 -> 34 -> -113 -> -72

36 -> 34 -> -113 -> -73

36 -> 34 -> -113 -> -77

36 -> 34 -> -113 -> -79

36 -> 34 -> -113 -> -80

36 -> 34 -> -113 -> -81

36 -> 34 -> -113 -> -84

36 -> 34 -> -113 -> -90

36 -> 34 -> -113 -> -91

36 -> 34 -> -113 -> -93

36 -> 34 -> -113 -> -96

36 -> 34 -> -113 -> -98

36 -> 34 -> -113 -> -103

36 -> 34 -> -113 -> -104

36 -> 34 -> -113 -> -105

36 -> 34 -> -113 -> -107

36 -> 34 -> -113 -> -116

36 -> 34 -> -113 -> -120

36 -> 34 -> -113 -> -123

36 -> 34 -> -113 -> -124

36 -> 34 -> -113 -> -126

36 -> 34 -> -113 -> -128

36 -> 34 -> -113 -> -129

36 -> 34 -> -113 -> -131

36 -> 34 -> -113 -> -132

36 -> 34 -> -113 -> -135

36 -> 34 -> -113 -> -136

36 -> 34 -> -113 -> -141

36 -> 34 -> -113 -> -145

36 -> 34 -> -113 -> -147

36 -> 34 -> -113 -> -148

36 -> 34 -> -113 -> -153

36 -> 37 -> 6 -> -73

36 -> 37 -> 7 -> -54

36 -> 37 -> 9 -> -6

36 -> 37 -> 14 -> -54

36 -> 37 -> 15 -> -68

36 -> 37 -> 19 -> -73

36 -> 37 -> 22 -> -152

36 -> 37 -> 24 -> -49

36 -> 37 -> 25 -> -132

36 -> 37 -> 27 -> -19

36 -> 37 -> 27 -> -20

36 -> 37 -> 27 -> -32

36 -> 37 -> 27 -> -62

36 -> 37 -> 27 -> -68

36 -> 37 -> 27 -> -73

36 -> 37 -> 27 -> -81

36 -> 37 -> 27 -> -98

36 -> 37 -> 27 -> -99

36 -> 37 -> 27 -> -101

36 -> 37 -> 27 -> -107

36 -> 37 -> 27 -> -124

36 -> 37 -> 27 -> -133

36 -> 37 -> 27 -> -145

36 -> 37 -> 28 -> -81

36 -> 37 -> 30 -> -1

36 -> 37 -> 30 -> -3

36 -> 37 -> 30 -> -32

36 -> 37 -> 30 -> -39

36 -> 37 -> 30 -> -63

36 -> 37 -> 30 -> -73

36 -> 37 -> 30 -> -81

36 -> 37 -> 30 -> -99

36 -> 37 -> 30 -> -101

36 -> 37 -> 30 -> -106

36 -> 37 -> 30 -> -117

36 -> 37 -> 30 -> -133

36 -> 37 -> 30 -> -137

36 -> 37 -> 30 -> -150

36 -> 37 -> 30 -> -156

36 -> 37 -> 32 -> -9

36 -> 37 -> 33 -> -145

36 -> 37 -> 41 -> -6

36 -> 37 -> 42 -> -133

36 -> 37 -> 43 -> -73

36 -> 37 -> 43 -> -99

36 -> 37 -> 43 -> -101

36 -> 37 -> 45 -> -73

36 -> 37 -> 48 -> -99

36 -> 37 -> 49 -> -15

36 -> 37 -> 55 -> -12

36 -> 37 -> 61 -> -27

36 -> 37 -> 62 -> -99

36 -> 37 -> 64 -> -10

36 -> 37 -> 65 -> -4

36 -> 37 -> 66 -> -136

36 -> 37 -> 67 -> -135

36 -> 37 -> 68 -> -73

36 -> 37 -> 69 -> -77

36 -> 37 -> 73 -> -32

36 -> 37 -> 74 -> -112

36 -> 37 -> 77 -> -73

36 -> 37 -> 78 -> -73

36 -> 37 -> 79 -> -73

36 -> 37 -> 84 -> -73

36 -> 37 -> 87 -> -73

36 -> 37 -> 88 -> -83

36 -> 37 -> 89 -> -6

36 -> 37 -> 89 -> -73

36 -> 37 -> 89 -> -81

36 -> 37 -> 89 -> -83

36 -> 37 -> 89 -> -86

36 -> 37 -> 89 -> -99

36 -> 37 -> 89 -> -101

36 -> 37 -> 89 -> -114

36 -> 37 -> 89 -> -155

36 -> 37 -> 91 -> -101

36 -> 37 -> 97 -> -15

36 -> 37 -> 103 -> -78

36 -> 37 -> 108 -> -80

36 -> 37 -> 113 -> -73

36 -> 37 -> 115 -> -17

36 -> 37 -> 115 -> -32

36 -> 37 -> 115 -> -68

36 -> 37 -> 115 -> -73

36 -> 37 -> 115 -> -92

36 -> 37 -> 116 -> -73

36 -> 37 -> 118 -> -73

36 -> 37 -> 120 -> -133

36 -> 37 -> 123 -> -73

36 -> 37 -> 129 -> -32

36 -> 37 -> 130 -> -73

36 -> 37 -> 138 -> -20

36 -> 37 -> 138 -> -61

36 -> 37 -> 138 -> -73

36 -> 37 -> 138 -> -98

36 -> 37 -> 138 -> -133

36 -> 37 -> 139 -> -15

36 -> 37 -> 140 -> -99

36 -> 37 -> 140 -> -133

36 -> 37 -> 141 -> -51

36 -> 37 -> 141 -> -100

36 -> 37 -> 141 -> -127

36 -> 37 -> 142 -> -154

36 -> 37 -> 144 -> -7

36 -> 37 -> 144 -> -110

36 -> 37 -> 144 -> -130

36 -> 37 -> 148 -> -32

36 -> 37 -> 149 -> -73

36 -> 37 -> 150 -> -91

36 -> 37 -> 151 -> -101

36 -> 37 -> 153 -> -2

36 -> 37 -> 157 -> -26

36 -> 37 -> 157 -> -28

36 -> 37 -> 157 -> -32

36 -> 37 -> 157 -> -68

36 -> 37 -> 157 -> -86

36 -> 37 -> 157 -> -99

36 -> 37 -> 157 -> -101

36 -> 37 -> 157 -> -118

36 -> 37 -> 157 -> -119

36 -> 37 -> 157 -> -120

36 -> 37 -> 157 -> -121

36 -> 37 -> 157 -> -122

36 -> 37 -> 157 -> -133

36 -> 37 -> 160 -> -129

36 -> 37 -> 162 -> -133

36 -> 37 -> 163 -> -8

36 -> 37 -> 168 -> -73

36 -> 37 -> 168 -> -102

36 -> 37 -> 169 -> -99

36 -> 37 -> 170 -> -138

36 -> 37 -> 171 -> -13

36 -> 37 -> 172 -> -58

36 -> 37 -> 176 -> -33

36 -> 37 -> 177 -> -106

36 -> 37 -> 179 -> -22

36 -> 37 -> 179 -> -147

36 -> 37 -> 181 -> -84

36 -> 37 -> 182 -> -73

36 -> 37 -> 186 -> -99

36 -> 37 -> 188 -> -24

36 -> 37 -> 189 -> -73

36 -> 37 -> 190 -> -43

36 -> 37 -> 190 -> -73

36 -> 37 -> 190 -> -86

36 -> 37 -> 190 -> -88

36 -> 37 -> 190 -> -144

36 -> 37 -> -73

36 -> 37 -> -73 -> -1

36 -> 37 -> -73 -> -3

36 -> 37 -> -73 -> -6

36 -> 37 -> -73 -> -17

36 -> 37 -> -73 -> -19

36 -> 37 -> -73 -> -20

36 -> 37 -> -73 -> -22

36 -> 37 -> -73 -> -26

36 -> 37 -> -73 -> -27

36 -> 37 -> -73 -> -28

36 -> 37 -> -73 -> -29

36 -> 37 -> -73 -> -30

36 -> 37 -> -73 -> -32

36 -> 37 -> -73 -> -35

36 -> 37 -> -73 -> -39

36 -> 37 -> -73 -> -43

36 -> 37 -> -73 -> -48

36 -> 37 -> -73 -> -56

36 -> 37 -> -73 -> -61

36 -> 37 -> -73 -> -62

36 -> 37 -> -73 -> -63

36 -> 37 -> -73 -> -64

36 -> 37 -> -73 -> -68

36 -> 37 -> -73 -> -71

36 -> 37 -> -73 -> -78

36 -> 37 -> -73 -> -81

36 -> 37 -> -73 -> -83

36 -> 37 -> -73 -> -86

36 -> 37 -> -73 -> -88

36 -> 37 -> -73 -> -89

36 -> 37 -> -73 -> -92

36 -> 37 -> -73 -> -95

36 -> 37 -> -73 -> -98

36 -> 37 -> -73 -> -99

36 -> 37 -> -73 -> -101

36 -> 37 -> -73 -> -106

36 -> 37 -> -73 -> -107

36 -> 37 -> -73 -> -113

36 -> 37 -> -73 -> -114

36 -> 37 -> -73 -> -117

36 -> 37 -> -73 -> -118

36 -> 37 -> -73 -> -119

36 -> 37 -> -73 -> -121

36 -> 37 -> -73 -> -122

36 -> 37 -> -73 -> -124

36 -> 37 -> -73 -> -133

36 -> 37 -> -73 -> -137

36 -> 37 -> -73 -> -144

36 -> 37 -> -73 -> -145

36 -> 37 -> -73 -> -146

36 -> 37 -> -73 -> -147

36 -> 37 -> -73 -> -150

36 -> 37 -> -73 -> -154

36 -> 37 -> -73 -> -155

36 -> 37 -> -73 -> -156

36 -> 39 -> 3 -> -32

36 -> 39 -> 3 -> -42

36 -> 39 -> 4 -> -101

36 -> 39 -> 7 -> -54

36 -> 39 -> 9 -> -6

36 -> 39 -> 14 -> -54

36 -> 39 -> 15 -> -68

36 -> 39 -> 19 -> -73

36 -> 39 -> 21 -> -73

36 -> 39 -> 21 -> -101

36 -> 39 -> 21 -> -154

36 -> 39 -> 22 -> -152

36 -> 39 -> 24 -> -49

36 -> 39 -> 25 -> -132

36 -> 39 -> 27 -> -19

36 -> 39 -> 27 -> -20

36 -> 39 -> 27 -> -32

36 -> 39 -> 27 -> -62

36 -> 39 -> 27 -> -68

36 -> 39 -> 27 -> -73

36 -> 39 -> 27 -> -81

36 -> 39 -> 27 -> -98

36 -> 39 -> 27 -> -99

36 -> 39 -> 27 -> -101

36 -> 39 -> 27 -> -107

36 -> 39 -> 27 -> -124

36 -> 39 -> 27 -> -133

36 -> 39 -> 27 -> -145

36 -> 39 -> 28 -> -81

36 -> 39 -> 29 -> -133

36 -> 39 -> 30 -> -1

36 -> 39 -> 30 -> -3

36 -> 39 -> 30 -> -32

36 -> 39 -> 30 -> -39

36 -> 39 -> 30 -> -63

36 -> 39 -> 30 -> -73

36 -> 39 -> 30 -> -81

36 -> 39 -> 30 -> -99

36 -> 39 -> 30 -> -101

36 -> 39 -> 30 -> -106

36 -> 39 -> 30 -> -117

36 -> 39 -> 30 -> -133

36 -> 39 -> 30 -> -137

36 -> 39 -> 30 -> -150

36 -> 39 -> 30 -> -156

36 -> 39 -> 32 -> -9

36 -> 39 -> 33 -> -145

36 -> 39 -> 38 -> -55

36 -> 39 -> 38 -> -56

36 -> 39 -> 38 -> -105

36 -> 39 -> 40 -> -73

36 -> 39 -> 41 -> -6

36 -> 39 -> 42 -> -133

36 -> 39 -> 44 -> -29

36 -> 39 -> 44 -> -48

36 -> 39 -> 44 -> -81

36 -> 39 -> 44 -> -89

36 -> 39 -> 44 -> -99

36 -> 39 -> 44 -> -114

36 -> 39 -> 45 -> -73

36 -> 39 -> 48 -> -99

36 -> 39 -> 49 -> -15

36 -> 39 -> 55 -> -12

36 -> 39 -> 60 -> -64

36 -> 39 -> 64 -> -10

36 -> 39 -> 65 -> -4

36 -> 39 -> 66 -> -136

36 -> 39 -> 67 -> -135

36 -> 39 -> 68 -> -73

36 -> 39 -> 69 -> -77

36 -> 39 -> 73 -> -32

36 -> 39 -> 74 -> -112

36 -> 39 -> 75 -> -1

36 -> 39 -> 75 -> -29

36 -> 39 -> 76 -> -81

36 -> 39 -> 77 -> -73

36 -> 39 -> 79 -> -73

36 -> 39 -> 84 -> -73

36 -> 39 -> 87 -> -73

36 -> 39 -> 88 -> -83

36 -> 39 -> 89 -> -6

36 -> 39 -> 89 -> -73

36 -> 39 -> 89 -> -81

36 -> 39 -> 89 -> -83

36 -> 39 -> 89 -> -86

36 -> 39 -> 89 -> -99

36 -> 39 -> 89 -> -101

36 -> 39 -> 89 -> -114

36 -> 39 -> 89 -> -155

36 -> 39 -> 91 -> -101

36 -> 39 -> 92 -> -133

36 -> 39 -> 97 -> -15

36 -> 39 -> 103 -> -78

36 -> 39 -> 105 -> -32

36 -> 39 -> 105 -> -146

36 -> 39 -> 107 -> -73

36 -> 39 -> 107 -> -99

36 -> 39 -> 108 -> -80

36 -> 39 -> 110 -> -56

36 -> 39 -> 113 -> -73

36 -> 39 -> 116 -> -73

36 -> 39 -> 118 -> -73

36 -> 39 -> 120 -> -133

36 -> 39 -> 121 -> -101

36 -> 39 -> 121 -> -141

36 -> 39 -> 122 -> -56

36 -> 39 -> 123 -> -73

36 -> 39 -> 128 -> -32

36 -> 39 -> 128 -> -95

36 -> 39 -> 129 -> -32

36 -> 39 -> 130 -> -73

36 -> 39 -> 134 -> -99

36 -> 39 -> 136 -> -20

36 -> 39 -> 139 -> -15

36 -> 39 -> 142 -> -154

36 -> 39 -> 146 -> -20

36 -> 39 -> 148 -> -32

36 -> 39 -> 149 -> -73

36 -> 39 -> 150 -> -91

36 -> 39 -> 153 -> -2

36 -> 39 -> 160 -> -129

36 -> 39 -> 161 -> -78

36 -> 39 -> 162 -> -133

36 -> 39 -> 163 -> -8

36 -> 39 -> 170 -> -138

36 -> 39 -> 171 -> -13

36 -> 39 -> 172 -> -58

36 -> 39 -> 176 -> -33

36 -> 39 -> 177 -> -106

36 -> 39 -> 179 -> -22

36 -> 39 -> 179 -> -147

36 -> 39 -> 180 -> -20

36 -> 39 -> 181 -> -84

36 -> 39 -> 186 -> -99

36 -> 39 -> 188 -> -24

36 -> 39 -> 189 -> -73

36 -> 39 -> -101

36 -> 39 -> -101 -> -1

36 -> 39 -> -101 -> -3

36 -> 39 -> -101 -> -6

36 -> 39 -> -101 -> -17

36 -> 39 -> -101 -> -19

36 -> 39 -> -101 -> -20

36 -> 39 -> -101 -> -22

36 -> 39 -> -101 -> -26

36 -> 39 -> -101 -> -28

36 -> 39 -> -101 -> -29

36 -> 39 -> -101 -> -30

36 -> 39 -> -101 -> -32

36 -> 39 -> -101 -> -35

36 -> 39 -> -101 -> -36

36 -> 39 -> -101 -> -39

36 -> 39 -> -101 -> -41

36 -> 39 -> -101 -> -43

36 -> 39 -> -101 -> -48

36 -> 39 -> -101 -> -51

36 -> 39 -> -101 -> -56

36 -> 39 -> -101 -> -61

36 -> 39 -> -101 -> -62

36 -> 39 -> -101 -> -63

36 -> 39 -> -101 -> -64

36 -> 39 -> -101 -> -68

36 -> 39 -> -101 -> -73

36 -> 39 -> -101 -> -74

36 -> 39 -> -101 -> -75

36 -> 39 -> -101 -> -78

36 -> 39 -> -101 -> -81

36 -> 39 -> -101 -> -83

36 -> 39 -> -101 -> -86

36 -> 39 -> -101 -> -89

36 -> 39 -> -101 -> -92

36 -> 39 -> -101 -> -95

36 -> 39 -> -101 -> -98

36 -> 39 -> -101 -> -99

36 -> 39 -> -101 -> -100

36 -> 39 -> -101 -> -106

36 -> 39 -> -101 -> -107

36 -> 39 -> -101 -> -109

36 -> 39 -> -101 -> -111

36 -> 39 -> -101 -> -114

36 -> 39 -> -101 -> -117

36 -> 39 -> -101 -> -118

36 -> 39 -> -101 -> -119

36 -> 39 -> -101 -> -121

36 -> 39 -> -101 -> -122

36 -> 39 -> -101 -> -124

36 -> 39 -> -101 -> -125

36 -> 39 -> -101 -> -127

36 -> 39 -> -101 -> -133

36 -> 39 -> -101 -> -137

36 -> 39 -> -101 -> -144

36 -> 39 -> -101 -> -145

36 -> 39 -> -101 -> -146

36 -> 39 -> -101 -> -147

36 -> 39 -> -101 -> -150

36 -> 39 -> -101 -> -151

36 -> 39 -> -101 -> -154

36 -> 39 -> -101 -> -155

36 -> 39 -> -101 -> -156

36 -> 43 -> 6 -> -73

36 -> 43 -> 19 -> -73

36 -> 43 -> 21 -> -73

36 -> 43 -> 21 -> -101

36 -> 43 -> 21 -> -154

36 -> 43 -> 27 -> -19

36 -> 43 -> 27 -> -20

36 -> 43 -> 27 -> -32

36 -> 43 -> 27 -> -62

36 -> 43 -> 27 -> -68

36 -> 43 -> 27 -> -73

36 -> 43 -> 27 -> -81

36 -> 43 -> 27 -> -98

36 -> 43 -> 27 -> -99

36 -> 43 -> 27 -> -101

36 -> 43 -> 27 -> -107

36 -> 43 -> 27 -> -124

36 -> 43 -> 27 -> -133

36 -> 43 -> 27 -> -145

36 -> 43 -> 30 -> -1

36 -> 43 -> 30 -> -3

36 -> 43 -> 30 -> -32

36 -> 43 -> 30 -> -39

36 -> 43 -> 30 -> -63

36 -> 43 -> 30 -> -73

36 -> 43 -> 30 -> -81

36 -> 43 -> 30 -> -99

36 -> 43 -> 30 -> -101

36 -> 43 -> 30 -> -106

36 -> 43 -> 30 -> -117

36 -> 43 -> 30 -> -133

36 -> 43 -> 30 -> -137

36 -> 43 -> 30 -> -150

36 -> 43 -> 30 -> -156

36 -> 43 -> 37 -> -73

36 -> 43 -> 44 -> -29

36 -> 43 -> 44 -> -48

36 -> 43 -> 44 -> -81

36 -> 43 -> 44 -> -89

36 -> 43 -> 44 -> -99

36 -> 43 -> 44 -> -114

36 -> 43 -> 45 -> -73

36 -> 43 -> 48 -> -99

36 -> 43 -> 60 -> -64

36 -> 43 -> 68 -> -73

36 -> 43 -> 75 -> -1

36 -> 43 -> 75 -> -29

36 -> 43 -> 76 -> -81

36 -> 43 -> 77 -> -73

36 -> 43 -> 78 -> -73

36 -> 43 -> 79 -> -73

36 -> 43 -> 84 -> -73

36 -> 43 -> 87 -> -73

36 -> 43 -> 89 -> -6

36 -> 43 -> 89 -> -73

36 -> 43 -> 89 -> -81

36 -> 43 -> 89 -> -83

36 -> 43 -> 89 -> -86

36 -> 43 -> 89 -> -99

36 -> 43 -> 89 -> -101

36 -> 43 -> 89 -> -114

36 -> 43 -> 89 -> -155

36 -> 43 -> 91 -> -101

36 -> 43 -> 107 -> -73

36 -> 43 -> 107 -> -99

36 -> 43 -> 113 -> -73

36 -> 43 -> 116 -> -73

36 -> 43 -> 118 -> -73

36 -> 43 -> 123 -> -73

36 -> 43 -> 128 -> -32

36 -> 43 -> 128 -> -95

36 -> 43 -> 129 -> -32

36 -> 43 -> 130 -> -73

36 -> 43 -> 136 -> -20

36 -> 43 -> 138 -> -20

36 -> 43 -> 138 -> -61

36 -> 43 -> 138 -> -73

36 -> 43 -> 138 -> -98

36 -> 43 -> 138 -> -133

36 -> 43 -> 140 -> -99

36 -> 43 -> 140 -> -133

36 -> 43 -> 141 -> -51

36 -> 43 -> 141 -> -100

36 -> 43 -> 141 -> -127

36 -> 43 -> 144 -> -7

36 -> 43 -> 144 -> -110

36 -> 43 -> 144 -> -130

36 -> 43 -> 149 -> -73

36 -> 43 -> 151 -> -101

36 -> 43 -> 157 -> -26

36 -> 43 -> 157 -> -28

36 -> 43 -> 157 -> -32

36 -> 43 -> 157 -> -68

36 -> 43 -> 157 -> -86

36 -> 43 -> 157 -> -99

36 -> 43 -> 157 -> -101

36 -> 43 -> 157 -> -118

36 -> 43 -> 157 -> -119

36 -> 43 -> 157 -> -120

36 -> 43 -> 157 -> -121

36 -> 43 -> 157 -> -122

36 -> 43 -> 157 -> -133

36 -> 43 -> 161 -> -78

36 -> 43 -> 169 -> -99

36 -> 43 -> 179 -> -22

36 -> 43 -> 179 -> -147

36 -> 43 -> 186 -> -99

36 -> 43 -> 189 -> -73

36 -> 43 -> 190 -> -43

36 -> 43 -> 190 -> -73

36 -> 43 -> 190 -> -86

36 -> 43 -> 190 -> -88

36 -> 43 -> 190 -> -144

36 -> 43 -> -73

36 -> 43 -> -73 -> -1

36 -> 43 -> -73 -> -3

36 -> 43 -> -73 -> -6

36 -> 43 -> -73 -> -17

36 -> 43 -> -73 -> -19

36 -> 43 -> -73 -> -20

36 -> 43 -> -73 -> -22

36 -> 43 -> -73 -> -26

36 -> 43 -> -73 -> -27

36 -> 43 -> -73 -> -28

36 -> 43 -> -73 -> -29

36 -> 43 -> -73 -> -30

36 -> 43 -> -73 -> -32

36 -> 43 -> -73 -> -35

36 -> 43 -> -73 -> -39

36 -> 43 -> -73 -> -43

36 -> 43 -> -73 -> -48

36 -> 43 -> -73 -> -56

36 -> 43 -> -73 -> -61

36 -> 43 -> -73 -> -62

36 -> 43 -> -73 -> -63

36 -> 43 -> -73 -> -64

36 -> 43 -> -73 -> -68

36 -> 43 -> -73 -> -71

36 -> 43 -> -73 -> -78

36 -> 43 -> -73 -> -81

36 -> 43 -> -73 -> -83

36 -> 43 -> -73 -> -86

36 -> 43 -> -73 -> -88

36 -> 43 -> -73 -> -89

36 -> 43 -> -73 -> -92

36 -> 43 -> -73 -> -95

36 -> 43 -> -73 -> -98

36 -> 43 -> -73 -> -99

36 -> 43 -> -73 -> -101

36 -> 43 -> -73 -> -106

36 -> 43 -> -73 -> -107

36 -> 43 -> -73 -> -113

36 -> 43 -> -73 -> -114

36 -> 43 -> -73 -> -117

36 -> 43 -> -73 -> -118

36 -> 43 -> -73 -> -119

36 -> 43 -> -73 -> -121

36 -> 43 -> -73 -> -122

36 -> 43 -> -73 -> -124

36 -> 43 -> -73 -> -133

36 -> 43 -> -73 -> -137

36 -> 43 -> -73 -> -144

36 -> 43 -> -73 -> -145

36 -> 43 -> -73 -> -146

36 -> 43 -> -73 -> -147

36 -> 43 -> -73 -> -150

36 -> 43 -> -73 -> -154

36 -> 43 -> -73 -> -155

36 -> 43 -> -73 -> -156

36 -> 43 -> -99

36 -> 43 -> -99 -> -1

36 -> 43 -> -99 -> -3

36 -> 43 -> -99 -> -6

36 -> 43 -> -99 -> -17

36 -> 43 -> -99 -> -18

36 -> 43 -> -99 -> -19

36 -> 43 -> -99 -> -20

36 -> 43 -> -99 -> -22

36 -> 43 -> -99 -> -26

36 -> 43 -> -99 -> -27

36 -> 43 -> -99 -> -28

36 -> 43 -> -99 -> -29

36 -> 43 -> -99 -> -30

36 -> 43 -> -99 -> -32

36 -> 43 -> -99 -> -35

36 -> 43 -> -99 -> -36

36 -> 43 -> -99 -> -39

36 -> 43 -> -99 -> -41

36 -> 43 -> -99 -> -43

36 -> 43 -> -99 -> -48

36 -> 43 -> -99 -> -51

36 -> 43 -> -99 -> -56

36 -> 43 -> -99 -> -61

36 -> 43 -> -99 -> -62

36 -> 43 -> -99 -> -63

36 -> 43 -> -99 -> -64

36 -> 43 -> -99 -> -68

36 -> 43 -> -99 -> -73

36 -> 43 -> -99 -> -78

36 -> 43 -> -99 -> -81

36 -> 43 -> -99 -> -83

36 -> 43 -> -99 -> -86

36 -> 43 -> -99 -> -88

36 -> 43 -> -99 -> -89

36 -> 43 -> -99 -> -92

36 -> 43 -> -99 -> -95

36 -> 43 -> -99 -> -97

36 -> 43 -> -99 -> -98

36 -> 43 -> -99 -> -100

36 -> 43 -> -99 -> -101

36 -> 43 -> -99 -> -106

36 -> 43 -> -99 -> -107

36 -> 43 -> -99 -> -109

36 -> 43 -> -99 -> -111

36 -> 43 -> -99 -> -114

36 -> 43 -> -99 -> -117

36 -> 43 -> -99 -> -118

36 -> 43 -> -99 -> -119

36 -> 43 -> -99 -> -121

36 -> 43 -> -99 -> -122

36 -> 43 -> -99 -> -124

36 -> 43 -> -99 -> -127

36 -> 43 -> -99 -> -133

36 -> 43 -> -99 -> -137

36 -> 43 -> -99 -> -144

36 -> 43 -> -99 -> -145

36 -> 43 -> -99 -> -146

36 -> 43 -> -99 -> -147

36 -> 43 -> -99 -> -150

36 -> 43 -> -99 -> -151

36 -> 43 -> -99 -> -154

36 -> 43 -> -99 -> -155

36 -> 43 -> -99 -> -156

36 -> 43 -> -101

36 -> 43 -> -101 -> -1

36 -> 43 -> -101 -> -3

36 -> 43 -> -101 -> -6

36 -> 43 -> -101 -> -17

36 -> 43 -> -101 -> -19

36 -> 43 -> -101 -> -20

36 -> 43 -> -101 -> -22

36 -> 43 -> -101 -> -26

36 -> 43 -> -101 -> -28

36 -> 43 -> -101 -> -29

36 -> 43 -> -101 -> -30

36 -> 43 -> -101 -> -32

36 -> 43 -> -101 -> -35

36 -> 43 -> -101 -> -36

36 -> 43 -> -101 -> -39

36 -> 43 -> -101 -> -41

36 -> 43 -> -101 -> -43

36 -> 43 -> -101 -> -48

36 -> 43 -> -101 -> -51

36 -> 43 -> -101 -> -56

36 -> 43 -> -101 -> -61

36 -> 43 -> -101 -> -62

36 -> 43 -> -101 -> -63

36 -> 43 -> -101 -> -64

36 -> 43 -> -101 -> -68

36 -> 43 -> -101 -> -73

36 -> 43 -> -101 -> -74

36 -> 43 -> -101 -> -75

36 -> 43 -> -101 -> -78

36 -> 43 -> -101 -> -81

36 -> 43 -> -101 -> -83

36 -> 43 -> -101 -> -86

36 -> 43 -> -101 -> -89

36 -> 43 -> -101 -> -92

36 -> 43 -> -101 -> -95

36 -> 43 -> -101 -> -98

36 -> 43 -> -101 -> -99

36 -> 43 -> -101 -> -100

36 -> 43 -> -101 -> -106

36 -> 43 -> -101 -> -107

36 -> 43 -> -101 -> -109

36 -> 43 -> -101 -> -111

36 -> 43 -> -101 -> -114

36 -> 43 -> -101 -> -117

36 -> 43 -> -101 -> -118

36 -> 43 -> -101 -> -119

36 -> 43 -> -101 -> -121

36 -> 43 -> -101 -> -122

36 -> 43 -> -101 -> -124

36 -> 43 -> -101 -> -125

36 -> 43 -> -101 -> -127

36 -> 43 -> -101 -> -133

36 -> 43 -> -101 -> -137

36 -> 43 -> -101 -> -144

36 -> 43 -> -101 -> -145

36 -> 43 -> -101 -> -146

36 -> 43 -> -101 -> -147

36 -> 43 -> -101 -> -150

36 -> 43 -> -101 -> -151

36 -> 43 -> -101 -> -154

36 -> 43 -> -101 -> -155

36 -> 43 -> -101 -> -156

36 -> 45 -> 2 -> -32

36 -> 45 -> 4 -> -101

36 -> 45 -> 6 -> -73

36 -> 45 -> 7 -> -54

36 -> 45 -> 9 -> -6

36 -> 45 -> 12 -> -32

36 -> 45 -> 13 -> -32

36 -> 45 -> 13 -> -73

36 -> 45 -> 14 -> -54

36 -> 45 -> 15 -> -68

36 -> 45 -> 16 -> -5

36 -> 45 -> 17 -> -32

36 -> 45 -> 18 -> -73

36 -> 45 -> 18 -> -89

36 -> 45 -> 19 -> -73

36 -> 45 -> 21 -> -73

36 -> 45 -> 21 -> -101

36 -> 45 -> 21 -> -154

36 -> 45 -> 22 -> -152

36 -> 45 -> 24 -> -49

36 -> 45 -> 25 -> -132

36 -> 45 -> 26 -> -14

36 -> 45 -> 28 -> -81

36 -> 45 -> 29 -> -133

36 -> 45 -> 31 -> -1

36 -> 45 -> 32 -> -9

36 -> 45 -> 33 -> -145

36 -> 45 -> 37 -> -73

36 -> 45 -> 39 -> -101

36 -> 45 -> 40 -> -73

36 -> 45 -> 41 -> -6

36 -> 45 -> 42 -> -133

36 -> 45 -> 43 -> -73

36 -> 45 -> 43 -> -99

36 -> 45 -> 43 -> -101

36 -> 45 -> 46 -> -32

36 -> 45 -> 48 -> -99

36 -> 45 -> 49 -> -15

36 -> 45 -> 50 -> -54

36 -> 45 -> 51 -> -2

36 -> 45 -> 52 -> -32

36 -> 45 -> 53 -> -133

36 -> 45 -> 54 -> -53

36 -> 45 -> 55 -> -12

36 -> 45 -> 56 -> -115

36 -> 45 -> 57 -> -103

36 -> 45 -> 58 -> -90

36 -> 45 -> 59 -> -148

36 -> 45 -> 60 -> -64

36 -> 45 -> 61 -> -27

36 -> 45 -> 62 -> -99

36 -> 45 -> 63 -> -32

36 -> 45 -> 64 -> -10

36 -> 45 -> 65 -> -4

36 -> 45 -> 66 -> -136

36 -> 45 -> 67 -> -135

36 -> 45 -> 68 -> -73

36 -> 45 -> 69 -> -77

36 -> 45 -> 70 -> -52

36 -> 45 -> 71 -> -32

36 -> 45 -> 73 -> -32

36 -> 45 -> 74 -> -112

36 -> 45 -> 76 -> -81

36 -> 45 -> 77 -> -73

36 -> 45 -> 78 -> -73

36 -> 45 -> 79 -> -73

36 -> 45 -> 80 -> -32

36 -> 45 -> 81 -> -73

36 -> 45 -> 83 -> -125

36 -> 45 -> 84 -> -73

36 -> 45 -> 85 -> -12

36 -> 45 -> 87 -> -73

36 -> 45 -> 88 -> -83

36 -> 45 -> 90 -> -103

36 -> 45 -> 91 -> -101

36 -> 45 -> 92 -> -133

36 -> 45 -> 93 -> -145

36 -> 45 -> 94 -> -13

36 -> 45 -> 96 -> -73

36 -> 45 -> 97 -> -15

36 -> 45 -> 99 -> -73

36 -> 45 -> 101 -> -64

36 -> 45 -> 102 -> -116

36 -> 45 -> 103 -> -78

36 -> 45 -> 104 -> -2

36 -> 45 -> 106 -> -65

36 -> 45 -> 107 -> -73

36 -> 45 -> 107 -> -99

36 -> 45 -> 108 -> -80

36 -> 45 -> 110 -> -56

36 -> 45 -> 112 -> -54

36 -> 45 -> 113 -> -73

36 -> 45 -> 114 -> -73

36 -> 45 -> 116 -> -73

36 -> 45 -> 117 -> -73

36 -> 45 -> 117 -> -101

36 -> 45 -> 118 -> -73

36 -> 45 -> 120 -> -133

36 -> 45 -> 122 -> -56

36 -> 45 -> 123 -> -73

36 -> 45 -> 125 -> -104

36 -> 45 -> 126 -> -59

36 -> 45 -> 127 -> -93

36 -> 45 -> 129 -> -32

36 -> 45 -> 130 -> -73

36 -> 45 -> 132 -> -32

36 -> 45 -> 133 -> -101

36 -> 45 -> 134 -> -99

36 -> 45 -> 135 -> -21

36 -> 45 -> 135 -> -73

36 -> 45 -> 135 -> -139

36 -> 45 -> 136 -> -20

36 -> 45 -> 139 -> -15

36 -> 45 -> 142 -> -154

36 -> 45 -> 143 -> -50

36 -> 45 -> 145 -> -125

36 -> 45 -> 146 -> -20

36 -> 45 -> 147 -> -32

36 -> 45 -> 148 -> -32

36 -> 45 -> 149 -> -73

36 -> 45 -> 150 -> -91

36 -> 45 -> 151 -> -101

36 -> 45 -> 152 -> -36

36 -> 45 -> 153 -> -2

36 -> 45 -> 155 -> -73

36 -> 45 -> 158 -> -72

36 -> 45 -> 160 -> -129

36 -> 45 -> 161 -> -78

36 -> 45 -> 162 -> -133

36 -> 45 -> 163 -> -8

36 -> 45 -> 164 -> -134

36 -> 45 -> 165 -> -54

36 -> 45 -> 168 -> -73

36 -> 45 -> 168 -> -102

36 -> 45 -> 169 -> -99

36 -> 45 -> 170 -> -138

36 -> 45 -> 171 -> -13

36 -> 45 -> 172 -> -58

36 -> 45 -> 173 -> -154

36 -> 45 -> 174 -> -114

36 -> 45 -> 176 -> -33

36 -> 45 -> 177 -> -106

36 -> 45 -> 178 -> -69

36 -> 45 -> 180 -> -20

36 -> 45 -> 181 -> -84

36 -> 45 -> 182 -> -73

36 -> 45 -> 186 -> -99

36 -> 45 -> 187 -> -53

36 -> 45 -> 188 -> -24

36 -> 45 -> 189 -> -73

36 -> 45 -> -73

36 -> 45 -> -73 -> -1

36 -> 45 -> -73 -> -3

36 -> 45 -> -73 -> -6

36 -> 45 -> -73 -> -17

36 -> 45 -> -73 -> -19

36 -> 45 -> -73 -> -20

36 -> 45 -> -73 -> -22

36 -> 45 -> -73 -> -26

36 -> 45 -> -73 -> -27

36 -> 45 -> -73 -> -28

36 -> 45 -> -73 -> -29

36 -> 45 -> -73 -> -30

36 -> 45 -> -73 -> -32

36 -> 45 -> -73 -> -35

36 -> 45 -> -73 -> -39

36 -> 45 -> -73 -> -43

36 -> 45 -> -73 -> -48

36 -> 45 -> -73 -> -56

36 -> 45 -> -73 -> -61

36 -> 45 -> -73 -> -62

36 -> 45 -> -73 -> -63

36 -> 45 -> -73 -> -64

36 -> 45 -> -73 -> -68

36 -> 45 -> -73 -> -71

36 -> 45 -> -73 -> -78

36 -> 45 -> -73 -> -81

36 -> 45 -> -73 -> -83

36 -> 45 -> -73 -> -86

36 -> 45 -> -73 -> -88

36 -> 45 -> -73 -> -89

36 -> 45 -> -73 -> -92

36 -> 45 -> -73 -> -95

36 -> 45 -> -73 -> -98

36 -> 45 -> -73 -> -99

36 -> 45 -> -73 -> -101

36 -> 45 -> -73 -> -106

36 -> 45 -> -73 -> -107

36 -> 45 -> -73 -> -113

36 -> 45 -> -73 -> -114

36 -> 45 -> -73 -> -117

36 -> 45 -> -73 -> -118

36 -> 45 -> -73 -> -119

36 -> 45 -> -73 -> -121

36 -> 45 -> -73 -> -122

36 -> 45 -> -73 -> -124

36 -> 45 -> -73 -> -133

36 -> 45 -> -73 -> -137

36 -> 45 -> -73 -> -144

36 -> 45 -> -73 -> -145

36 -> 45 -> -73 -> -146

36 -> 45 -> -73 -> -147

36 -> 45 -> -73 -> -150

36 -> 45 -> -73 -> -154

36 -> 45 -> -73 -> -155

36 -> 45 -> -73 -> -156

36 -> 48 -> 2 -> -32

36 -> 48 -> 4 -> -101

36 -> 48 -> 6 -> -73

36 -> 48 -> 7 -> -54

36 -> 48 -> 9 -> -6

36 -> 48 -> 12 -> -32

36 -> 48 -> 14 -> -54

36 -> 48 -> 15 -> -68

36 -> 48 -> 16 -> -5

36 -> 48 -> 17 -> -32

36 -> 48 -> 19 -> -73

36 -> 48 -> 22 -> -152

36 -> 48 -> 24 -> -49

36 -> 48 -> 25 -> -132

36 -> 48 -> 26 -> -14

36 -> 48 -> 28 -> -81

36 -> 48 -> 29 -> -133

36 -> 48 -> 31 -> -1

36 -> 48 -> 32 -> -9

36 -> 48 -> 33 -> -145

36 -> 48 -> 37 -> -73

36 -> 48 -> 39 -> -101

36 -> 48 -> 40 -> -73

36 -> 48 -> 41 -> -6

36 -> 48 -> 42 -> -133

36 -> 48 -> 43 -> -73

36 -> 48 -> 43 -> -99

36 -> 48 -> 43 -> -101

36 -> 48 -> 45 -> -73

36 -> 48 -> 46 -> -32

36 -> 48 -> 49 -> -15

36 -> 48 -> 50 -> -54

36 -> 48 -> 51 -> -2

36 -> 48 -> 52 -> -32

36 -> 48 -> 53 -> -133

36 -> 48 -> 54 -> -53

36 -> 48 -> 55 -> -12

36 -> 48 -> 56 -> -115

36 -> 48 -> 57 -> -103

36 -> 48 -> 58 -> -90

36 -> 48 -> 59 -> -148

36 -> 48 -> 60 -> -64

36 -> 48 -> 61 -> -27

36 -> 48 -> 62 -> -99

36 -> 48 -> 63 -> -32

36 -> 48 -> 64 -> -10

36 -> 48 -> 65 -> -4

36 -> 48 -> 66 -> -136

36 -> 48 -> 67 -> -135

36 -> 48 -> 68 -> -73

36 -> 48 -> 69 -> -77

36 -> 48 -> 70 -> -52

36 -> 48 -> 71 -> -32

36 -> 48 -> 73 -> -32

36 -> 48 -> 74 -> -112

36 -> 48 -> 76 -> -81

36 -> 48 -> 77 -> -73

36 -> 48 -> 78 -> -73

36 -> 48 -> 79 -> -73

36 -> 48 -> 80 -> -32

36 -> 48 -> 81 -> -73

36 -> 48 -> 83 -> -125

36 -> 48 -> 84 -> -73

36 -> 48 -> 85 -> -12

36 -> 48 -> 87 -> -73

36 -> 48 -> 88 -> -83

36 -> 48 -> 90 -> -103

36 -> 48 -> 91 -> -101

36 -> 48 -> 92 -> -133

36 -> 48 -> 93 -> -145

36 -> 48 -> 94 -> -13

36 -> 48 -> 96 -> -73

36 -> 48 -> 97 -> -15

36 -> 48 -> 99 -> -73

36 -> 48 -> 101 -> -64

36 -> 48 -> 102 -> -116

36 -> 48 -> 103 -> -78

36 -> 48 -> 104 -> -2

36 -> 48 -> 106 -> -65

36 -> 48 -> 107 -> -73

36 -> 48 -> 107 -> -99

36 -> 48 -> 108 -> -80

36 -> 48 -> 110 -> -56

36 -> 48 -> 112 -> -54

36 -> 48 -> 113 -> -73

36 -> 48 -> 114 -> -73

36 -> 48 -> 116 -> -73

36 -> 48 -> 118 -> -73

36 -> 48 -> 120 -> -133

36 -> 48 -> 122 -> -56

36 -> 48 -> 123 -> -73

36 -> 48 -> 125 -> -104

36 -> 48 -> 126 -> -59

36 -> 48 -> 127 -> -93

36 -> 48 -> 129 -> -32

36 -> 48 -> 130 -> -73

36 -> 48 -> 132 -> -32

36 -> 48 -> 133 -> -101

36 -> 48 -> 134 -> -99

36 -> 48 -> 136 -> -20

36 -> 48 -> 137 -> -18

36 -> 48 -> 137 -> -97

36 -> 48 -> 137 -> -99

36 -> 48 -> 139 -> -15

36 -> 48 -> 140 -> -99

36 -> 48 -> 140 -> -133

36 -> 48 -> 142 -> -154

36 -> 48 -> 143 -> -50

36 -> 48 -> 145 -> -125

36 -> 48 -> 146 -> -20

36 -> 48 -> 147 -> -32

36 -> 48 -> 148 -> -32

36 -> 48 -> 149 -> -73

36 -> 48 -> 150 -> -91

36 -> 48 -> 151 -> -101

36 -> 48 -> 152 -> -36

36 -> 48 -> 153 -> -2

36 -> 48 -> 155 -> -73

36 -> 48 -> 158 -> -72

36 -> 48 -> 160 -> -129

36 -> 48 -> 161 -> -78

36 -> 48 -> 162 -> -133

36 -> 48 -> 163 -> -8

36 -> 48 -> 164 -> -134

36 -> 48 -> 165 -> -54

36 -> 48 -> 169 -> -99

36 -> 48 -> 170 -> -138

36 -> 48 -> 171 -> -13

36 -> 48 -> 172 -> -58

36 -> 48 -> 173 -> -154

36 -> 48 -> 174 -> -114

36 -> 48 -> 176 -> -33

36 -> 48 -> 177 -> -106

36 -> 48 -> 178 -> -69

36 -> 48 -> 180 -> -20

36 -> 48 -> 181 -> -84

36 -> 48 -> 182 -> -73

36 -> 48 -> 186 -> -99

36 -> 48 -> 187 -> -53

36 -> 48 -> 188 -> -24

36 -> 48 -> 189 -> -73

36 -> 48 -> -99

36 -> 48 -> -99 -> -1

36 -> 48 -> -99 -> -3

36 -> 48 -> -99 -> -6

36 -> 48 -> -99 -> -17

36 -> 48 -> -99 -> -18

36 -> 48 -> -99 -> -19

36 -> 48 -> -99 -> -20

36 -> 48 -> -99 -> -22

36 -> 48 -> -99 -> -26

36 -> 48 -> -99 -> -27

36 -> 48 -> -99 -> -28

36 -> 48 -> -99 -> -29

36 -> 48 -> -99 -> -30

36 -> 48 -> -99 -> -32

36 -> 48 -> -99 -> -35

36 -> 48 -> -99 -> -36

36 -> 48 -> -99 -> -39

36 -> 48 -> -99 -> -41

36 -> 48 -> -99 -> -43

36 -> 48 -> -99 -> -48

36 -> 48 -> -99 -> -51

36 -> 48 -> -99 -> -56

36 -> 48 -> -99 -> -61

36 -> 48 -> -99 -> -62

36 -> 48 -> -99 -> -63

36 -> 48 -> -99 -> -64

36 -> 48 -> -99 -> -68

36 -> 48 -> -99 -> -73

36 -> 48 -> -99 -> -78

36 -> 48 -> -99 -> -81

36 -> 48 -> -99 -> -83

36 -> 48 -> -99 -> -86

36 -> 48 -> -99 -> -88

36 -> 48 -> -99 -> -89

36 -> 48 -> -99 -> -92

36 -> 48 -> -99 -> -95

36 -> 48 -> -99 -> -97

36 -> 48 -> -99 -> -98

36 -> 48 -> -99 -> -100

36 -> 48 -> -99 -> -101

36 -> 48 -> -99 -> -106

36 -> 48 -> -99 -> -107

36 -> 48 -> -99 -> -109

36 -> 48 -> -99 -> -111

36 -> 48 -> -99 -> -114

36 -> 48 -> -99 -> -117

36 -> 48 -> -99 -> -118

36 -> 48 -> -99 -> -119

36 -> 48 -> -99 -> -121

36 -> 48 -> -99 -> -122

36 -> 48 -> -99 -> -124

36 -> 48 -> -99 -> -127

36 -> 48 -> -99 -> -133

36 -> 48 -> -99 -> -137

36 -> 48 -> -99 -> -144

36 -> 48 -> -99 -> -145

36 -> 48 -> -99 -> -146

36 -> 48 -> -99 -> -147

36 -> 48 -> -99 -> -150

36 -> 48 -> -99 -> -151

36 -> 48 -> -99 -> -154

36 -> 48 -> -99 -> -155

36 -> 48 -> -99 -> -156

36 -> 60 -> 3 -> -32

36 -> 60 -> 3 -> -42

36 -> 60 -> 4 -> -101

36 -> 60 -> 6 -> -73

36 -> 60 -> 7 -> -54

36 -> 60 -> 9 -> -6

36 -> 60 -> 14 -> -54

36 -> 60 -> 15 -> -68

36 -> 60 -> 17 -> -32

36 -> 60 -> 19 -> -73

36 -> 60 -> 21 -> -73

36 -> 60 -> 21 -> -101

36 -> 60 -> 21 -> -154

36 -> 60 -> 22 -> -152

36 -> 60 -> 24 -> -49

36 -> 60 -> 25 -> -132

36 -> 60 -> 27 -> -19

36 -> 60 -> 27 -> -20

36 -> 60 -> 27 -> -32

36 -> 60 -> 27 -> -62

36 -> 60 -> 27 -> -68

36 -> 60 -> 27 -> -73

36 -> 60 -> 27 -> -81

36 -> 60 -> 27 -> -98

36 -> 60 -> 27 -> -99

36 -> 60 -> 27 -> -101

36 -> 60 -> 27 -> -107

36 -> 60 -> 27 -> -124

36 -> 60 -> 27 -> -133

36 -> 60 -> 27 -> -145

36 -> 60 -> 28 -> -81

36 -> 60 -> 29 -> -133

36 -> 60 -> 30 -> -1

36 -> 60 -> 30 -> -3

36 -> 60 -> 30 -> -32

36 -> 60 -> 30 -> -39

36 -> 60 -> 30 -> -63

36 -> 60 -> 30 -> -73

36 -> 60 -> 30 -> -81

36 -> 60 -> 30 -> -99

36 -> 60 -> 30 -> -101

36 -> 60 -> 30 -> -106

36 -> 60 -> 30 -> -117

36 -> 60 -> 30 -> -133

36 -> 60 -> 30 -> -137

36 -> 60 -> 30 -> -150

36 -> 60 -> 30 -> -156

36 -> 60 -> 32 -> -9

36 -> 60 -> 33 -> -145

36 -> 60 -> 38 -> -55

36 -> 60 -> 38 -> -56

36 -> 60 -> 38 -> -105

36 -> 60 -> 39 -> -101

36 -> 60 -> 40 -> -73

36 -> 60 -> 41 -> -6

36 -> 60 -> 42 -> -133

36 -> 60 -> 43 -> -73

36 -> 60 -> 43 -> -99

36 -> 60 -> 43 -> -101

36 -> 60 -> 44 -> -29

36 -> 60 -> 44 -> -48

36 -> 60 -> 44 -> -81

36 -> 60 -> 44 -> -89

36 -> 60 -> 44 -> -99

36 -> 60 -> 44 -> -114

36 -> 60 -> 45 -> -73

36 -> 60 -> 48 -> -99

36 -> 60 -> 49 -> -15

36 -> 60 -> 55 -> -12

36 -> 60 -> 61 -> -27

36 -> 60 -> 64 -> -10

36 -> 60 -> 65 -> -4

36 -> 60 -> 66 -> -136

36 -> 60 -> 67 -> -135

36 -> 60 -> 68 -> -73

36 -> 60 -> 69 -> -77

36 -> 60 -> 73 -> -32

36 -> 60 -> 74 -> -112

36 -> 60 -> 75 -> -1

36 -> 60 -> 75 -> -29

36 -> 60 -> 76 -> -81

36 -> 60 -> 77 -> -73

36 -> 60 -> 79 -> -73

36 -> 60 -> 81 -> -73

36 -> 60 -> 82 -> -30

36 -> 60 -> 82 -> -32

36 -> 60 -> 82 -> -35

36 -> 60 -> 82 -> -101

36 -> 60 -> 83 -> -125

36 -> 60 -> 84 -> -73

36 -> 60 -> 87 -> -73

36 -> 60 -> 88 -> -83

36 -> 60 -> 89 -> -6

36 -> 60 -> 89 -> -73

36 -> 60 -> 89 -> -81

36 -> 60 -> 89 -> -83

36 -> 60 -> 89 -> -86

36 -> 60 -> 89 -> -99

36 -> 60 -> 89 -> -101

36 -> 60 -> 89 -> -114

36 -> 60 -> 89 -> -155

36 -> 60 -> 91 -> -101

36 -> 60 -> 92 -> -133

36 -> 60 -> 97 -> -15

36 -> 60 -> 103 -> -78

36 -> 60 -> 105 -> -32

36 -> 60 -> 105 -> -146

36 -> 60 -> 107 -> -73

36 -> 60 -> 107 -> -99

36 -> 60 -> 108 -> -80

36 -> 60 -> 113 -> -73

36 -> 60 -> 114 -> -73

36 -> 60 -> 115 -> -17

36 -> 60 -> 115 -> -32

36 -> 60 -> 115 -> -68

36 -> 60 -> 115 -> -73

36 -> 60 -> 115 -> -92

36 -> 60 -> 116 -> -73

36 -> 60 -> 117 -> -73

36 -> 60 -> 117 -> -101

36 -> 60 -> 118 -> -73

36 -> 60 -> 120 -> -133

36 -> 60 -> 122 -> -56

36 -> 60 -> 123 -> -73

36 -> 60 -> 128 -> -32

36 -> 60 -> 128 -> -95

36 -> 60 -> 129 -> -32

36 -> 60 -> 130 -> -73

36 -> 60 -> 133 -> -101

36 -> 60 -> 134 -> -99

36 -> 60 -> 136 -> -20

36 -> 60 -> 139 -> -15

36 -> 60 -> 141 -> -51

36 -> 60 -> 141 -> -100

36 -> 60 -> 141 -> -127

36 -> 60 -> 142 -> -154

36 -> 60 -> 144 -> -7

36 -> 60 -> 144 -> -110

36 -> 60 -> 144 -> -130

36 -> 60 -> 146 -> -20

36 -> 60 -> 148 -> -32

36 -> 60 -> 149 -> -73

36 -> 60 -> 150 -> -91

36 -> 60 -> 151 -> -101

36 -> 60 -> 152 -> -36

36 -> 60 -> 153 -> -2

36 -> 60 -> 160 -> -129

36 -> 60 -> 161 -> -78

36 -> 60 -> 162 -> -133

36 -> 60 -> 163 -> -8

36 -> 60 -> 169 -> -99

36 -> 60 -> 170 -> -138

36 -> 60 -> 171 -> -13

36 -> 60 -> 172 -> -58

36 -> 60 -> 173 -> -154

36 -> 60 -> 174 -> -114

36 -> 60 -> 176 -> -33

36 -> 60 -> 177 -> -106

36 -> 60 -> 179 -> -22

36 -> 60 -> 179 -> -147

36 -> 60 -> 180 -> -20

36 -> 60 -> 181 -> -84

36 -> 60 -> 182 -> -73

36 -> 60 -> 186 -> -99

36 -> 60 -> 188 -> -24

36 -> 60 -> 189 -> -73

36 -> 60 -> 190 -> -43

36 -> 60 -> 190 -> -73

36 -> 60 -> 190 -> -86

36 -> 60 -> 190 -> -88

36 -> 60 -> 190 -> -144

36 -> 60 -> -64

36 -> 60 -> -64 -> -1

36 -> 60 -> -64 -> -3

36 -> 60 -> -64 -> -4

36 -> 60 -> -64 -> -6

36 -> 60 -> -64 -> -8

36 -> 60 -> -64 -> -9

36 -> 60 -> -64 -> -10

36 -> 60 -> -64 -> -17

36 -> 60 -> -64 -> -19

36 -> 60 -> -64 -> -20

36 -> 60 -> -64 -> -22

36 -> 60 -> -64 -> -24

36 -> 60 -> -64 -> -29

36 -> 60 -> -64 -> -30

36 -> 60 -> -64 -> -31

36 -> 60 -> -64 -> -32

36 -> 60 -> -64 -> -33

36 -> 60 -> -64 -> -35

36 -> 60 -> -64 -> -36

36 -> 60 -> -64 -> -39

36 -> 60 -> -64 -> -41

36 -> 60 -> -64 -> -43

36 -> 60 -> -64 -> -44

36 -> 60 -> -64 -> -45

36 -> 60 -> -64 -> -46

36 -> 60 -> -64 -> -47

36 -> 60 -> -64 -> -48

36 -> 60 -> -64 -> -49

36 -> 60 -> -64 -> -51

36 -> 60 -> -64 -> -56

36 -> 60 -> -64 -> -58

36 -> 60 -> -64 -> -60

36 -> 60 -> -64 -> -62

36 -> 60 -> -64 -> -63

36 -> 60 -> -64 -> -66

36 -> 60 -> -64 -> -69

36 -> 60 -> -64 -> -73

36 -> 60 -> -64 -> -77

36 -> 60 -> -64 -> -78

36 -> 60 -> -64 -> -81

36 -> 60 -> -64 -> -83

36 -> 60 -> -64 -> -84

36 -> 60 -> -64 -> -86

36 -> 60 -> -64 -> -89

36 -> 60 -> -64 -> -91

36 -> 60 -> -64 -> -92

36 -> 60 -> -64 -> -95

36 -> 60 -> -64 -> -96

36 -> 60 -> -64 -> -98

36 -> 60 -> -64 -> -99

36 -> 60 -> -64 -> -100

36 -> 60 -> -64 -> -101

36 -> 60 -> -64 -> -106

36 -> 60 -> -64 -> -107

36 -> 60 -> -64 -> -109

36 -> 60 -> -64 -> -111

36 -> 60 -> -64 -> -114

36 -> 60 -> -64 -> -117

36 -> 60 -> -64 -> -120

36 -> 60 -> -64 -> -124

36 -> 60 -> -64 -> -127

36 -> 60 -> -64 -> -131

36 -> 60 -> -64 -> -132

36 -> 60 -> -64 -> -133

36 -> 60 -> -64 -> -135

36 -> 60 -> -64 -> -136

36 -> 60 -> -64 -> -137

36 -> 60 -> -64 -> -141

36 -> 60 -> -64 -> -144

36 -> 60 -> -64 -> -146

36 -> 60 -> -64 -> -147

36 -> 60 -> -64 -> -150

36 -> 60 -> -64 -> -151

36 -> 60 -> -64 -> -154

36 -> 60 -> -64 -> -155

36 -> 60 -> -64 -> -156

36 -> 61 -> 7 -> -54

36 -> 61 -> 9 -> -6

36 -> 61 -> 14 -> -54

36 -> 61 -> 15 -> -68

36 -> 61 -> 19 -> -73

36 -> 61 -> 21 -> -73

36 -> 61 -> 21 -> -101

36 -> 61 -> 21 -> -154

36 -> 61 -> 22 -> -152

36 -> 61 -> 24 -> -49

36 -> 61 -> 25 -> -132

36 -> 61 -> 27 -> -19

36 -> 61 -> 27 -> -20

36 -> 61 -> 27 -> -32

36 -> 61 -> 27 -> -62

36 -> 61 -> 27 -> -68

36 -> 61 -> 27 -> -73

36 -> 61 -> 27 -> -81

36 -> 61 -> 27 -> -98

36 -> 61 -> 27 -> -99

36 -> 61 -> 27 -> -101

36 -> 61 -> 27 -> -107

36 -> 61 -> 27 -> -124

36 -> 61 -> 27 -> -133

36 -> 61 -> 27 -> -145

36 -> 61 -> 28 -> -81

36 -> 61 -> 30 -> -1

36 -> 61 -> 30 -> -3

36 -> 61 -> 30 -> -32

36 -> 61 -> 30 -> -39

36 -> 61 -> 30 -> -63

36 -> 61 -> 30 -> -73

36 -> 61 -> 30 -> -81

36 -> 61 -> 30 -> -99

36 -> 61 -> 30 -> -101

36 -> 61 -> 30 -> -106

36 -> 61 -> 30 -> -117

36 -> 61 -> 30 -> -133

36 -> 61 -> 30 -> -137

36 -> 61 -> 30 -> -150

36 -> 61 -> 30 -> -156

36 -> 61 -> 32 -> -9

36 -> 61 -> 33 -> -145

36 -> 61 -> 34 -> -32

36 -> 61 -> 34 -> -71

36 -> 61 -> 34 -> -113

36 -> 61 -> 37 -> -73

36 -> 61 -> 41 -> -6

36 -> 61 -> 42 -> -133

36 -> 61 -> 45 -> -73

36 -> 61 -> 48 -> -99

36 -> 61 -> 49 -> -15

36 -> 61 -> 55 -> -12

36 -> 61 -> 60 -> -64

36 -> 61 -> 62 -> -99

36 -> 61 -> 63 -> -32

36 -> 61 -> 64 -> -10

36 -> 61 -> 65 -> -4

36 -> 61 -> 66 -> -136

36 -> 61 -> 67 -> -135

36 -> 61 -> 68 -> -73

36 -> 61 -> 69 -> -77

36 -> 61 -> 73 -> -32

36 -> 61 -> 74 -> -112

36 -> 61 -> 75 -> -1

36 -> 61 -> 75 -> -29

36 -> 61 -> 76 -> -81

36 -> 61 -> 77 -> -73

36 -> 61 -> 78 -> -73

36 -> 61 -> 79 -> -73

36 -> 61 -> 84 -> -73

36 -> 61 -> 87 -> -73

36 -> 61 -> 88 -> -83

36 -> 61 -> 91 -> -101

36 -> 61 -> 92 -> -133

36 -> 61 -> 97 -> -15

36 -> 61 -> 99 -> -73

36 -> 61 -> 103 -> -78

36 -> 61 -> 105 -> -32

36 -> 61 -> 105 -> -146

36 -> 61 -> 107 -> -73

36 -> 61 -> 107 -> -99

36 -> 61 -> 108 -> -80

36 -> 61 -> 113 -> -73

36 -> 61 -> 116 -> -73

36 -> 61 -> 118 -> -73

36 -> 61 -> 120 -> -133

36 -> 61 -> 122 -> -56

36 -> 61 -> 123 -> -73

36 -> 61 -> 128 -> -32

36 -> 61 -> 128 -> -95

36 -> 61 -> 129 -> -32

36 -> 61 -> 130 -> -73

36 -> 61 -> 136 -> -20

36 -> 61 -> 138 -> -20

36 -> 61 -> 138 -> -61

36 -> 61 -> 138 -> -73

36 -> 61 -> 138 -> -98

36 -> 61 -> 138 -> -133

36 -> 61 -> 139 -> -15

36 -> 61 -> 140 -> -99

36 -> 61 -> 140 -> -133

36 -> 61 -> 142 -> -154

36 -> 61 -> 148 -> -32

36 -> 61 -> 149 -> -73

36 -> 61 -> 150 -> -91

36 -> 61 -> 153 -> -2

36 -> 61 -> 157 -> -26

36 -> 61 -> 157 -> -28

36 -> 61 -> 157 -> -32

36 -> 61 -> 157 -> -68

36 -> 61 -> 157 -> -86

36 -> 61 -> 157 -> -99

36 -> 61 -> 157 -> -101

36 -> 61 -> 157 -> -118

36 -> 61 -> 157 -> -119

36 -> 61 -> 157 -> -120

36 -> 61 -> 157 -> -121

36 -> 61 -> 157 -> -122

36 -> 61 -> 157 -> -133

36 -> 61 -> 160 -> -129

36 -> 61 -> 161 -> -78

36 -> 61 -> 162 -> -133

36 -> 61 -> 163 -> -8

36 -> 61 -> 170 -> -138

36 -> 61 -> 171 -> -13

36 -> 61 -> 172 -> -58

36 -> 61 -> 176 -> -33

36 -> 61 -> 177 -> -106

36 -> 61 -> 179 -> -22

36 -> 61 -> 179 -> -147

36 -> 61 -> 181 -> -84

36 -> 61 -> 182 -> -73

36 -> 61 -> 186 -> -99

36 -> 61 -> 188 -> -24

36 -> 61 -> 189 -> -73

36 -> 61 -> -27 -> -1

36 -> 61 -> -27 -> -3

36 -> 61 -> -27 -> -4

36 -> 61 -> -27 -> -8

36 -> 61 -> -27 -> -9

36 -> 61 -> -27 -> -10

36 -> 61 -> -27 -> -19

36 -> 61 -> -27 -> -20

36 -> 61 -> -27 -> -22

36 -> 61 -> -27 -> -24

36 -> 61 -> -27 -> -26

36 -> 61 -> -27 -> -28

36 -> 61 -> -27 -> -29

36 -> 61 -> -27 -> -31

36 -> 61 -> -27 -> -33

36 -> 61 -> -27 -> -39

36 -> 61 -> -27 -> -44

36 -> 61 -> -27 -> -45

36 -> 61 -> -27 -> -46

36 -> 61 -> -27 -> -47

36 -> 61 -> -27 -> -49

36 -> 61 -> -27 -> -58

36 -> 61 -> -27 -> -60

36 -> 61 -> -27 -> -61

36 -> 61 -> -27 -> -62

36 -> 61 -> -27 -> -63

36 -> 61 -> -27 -> -66

36 -> 61 -> -27 -> -68

36 -> 61 -> -27 -> -69

36 -> 61 -> -27 -> -71

36 -> 61 -> -27 -> -73

36 -> 61 -> -27 -> -77

36 -> 61 -> -27 -> -78

36 -> 61 -> -27 -> -81

36 -> 61 -> -27 -> -84

36 -> 61 -> -27 -> -86

36 -> 61 -> -27 -> -88

36 -> 61 -> -27 -> -91

36 -> 61 -> -27 -> -95

36 -> 61 -> -27 -> -96

36 -> 61 -> -27 -> -98

36 -> 61 -> -27 -> -99

36 -> 61 -> -27 -> -106

36 -> 61 -> -27 -> -107

36 -> 61 -> -27 -> -113

36 -> 61 -> -27 -> -117

36 -> 61 -> -27 -> -118

36 -> 61 -> -27 -> -119

36 -> 61 -> -27 -> -120

36 -> 61 -> -27 -> -121

36 -> 61 -> -27 -> -122

36 -> 61 -> -27 -> -124

36 -> 61 -> -27 -> -131

36 -> 61 -> -27 -> -132

36 -> 61 -> -27 -> -133

36 -> 61 -> -27 -> -135

36 -> 61 -> -27 -> -136

36 -> 61 -> -27 -> -137

36 -> 61 -> -27 -> -141

36 -> 61 -> -27 -> -145

36 -> 61 -> -27 -> -147

36 -> 61 -> -27 -> -150

36 -> 61 -> -27 -> -156

36 -> 62 -> 7 -> -54

36 -> 62 -> 9 -> -6

36 -> 62 -> 14 -> -54

36 -> 62 -> 15 -> -68

36 -> 62 -> 19 -> -73

36 -> 62 -> 22 -> -152

36 -> 62 -> 24 -> -49

36 -> 62 -> 25 -> -132

36 -> 62 -> 27 -> -19

36 -> 62 -> 27 -> -20

36 -> 62 -> 27 -> -32

36 -> 62 -> 27 -> -62

36 -> 62 -> 27 -> -68

36 -> 62 -> 27 -> -73

36 -> 62 -> 27 -> -81

36 -> 62 -> 27 -> -98

36 -> 62 -> 27 -> -99

36 -> 62 -> 27 -> -101

36 -> 62 -> 27 -> -107

36 -> 62 -> 27 -> -124

36 -> 62 -> 27 -> -133

36 -> 62 -> 27 -> -145

36 -> 62 -> 28 -> -81

36 -> 62 -> 30 -> -1

36 -> 62 -> 30 -> -3

36 -> 62 -> 30 -> -32

36 -> 62 -> 30 -> -39

36 -> 62 -> 30 -> -63

36 -> 62 -> 30 -> -73

36 -> 62 -> 30 -> -81

36 -> 62 -> 30 -> -99

36 -> 62 -> 30 -> -101

36 -> 62 -> 30 -> -106

36 -> 62 -> 30 -> -117

36 -> 62 -> 30 -> -133

36 -> 62 -> 30 -> -137

36 -> 62 -> 30 -> -150

36 -> 62 -> 30 -> -156

36 -> 62 -> 32 -> -9

36 -> 62 -> 33 -> -145

36 -> 62 -> 37 -> -73

36 -> 62 -> 41 -> -6

36 -> 62 -> 42 -> -133

36 -> 62 -> 45 -> -73

36 -> 62 -> 48 -> -99

36 -> 62 -> 49 -> -15

36 -> 62 -> 55 -> -12

36 -> 62 -> 61 -> -27

36 -> 62 -> 64 -> -10

36 -> 62 -> 65 -> -4

36 -> 62 -> 66 -> -136

36 -> 62 -> 67 -> -135

36 -> 62 -> 68 -> -73

36 -> 62 -> 69 -> -77

36 -> 62 -> 73 -> -32

36 -> 62 -> 74 -> -112

36 -> 62 -> 75 -> -1

36 -> 62 -> 75 -> -29

36 -> 62 -> 77 -> -73

36 -> 62 -> 78 -> -73

36 -> 62 -> 79 -> -73

36 -> 62 -> 84 -> -73

36 -> 62 -> 87 -> -73

36 -> 62 -> 88 -> -83

36 -> 62 -> 91 -> -101

36 -> 62 -> 97 -> -15

36 -> 62 -> 102 -> -116

36 -> 62 -> 108 -> -80

36 -> 62 -> 113 -> -73

36 -> 62 -> 115 -> -17

36 -> 62 -> 115 -> -32

36 -> 62 -> 115 -> -68

36 -> 62 -> 115 -> -73

36 -> 62 -> 115 -> -92

36 -> 62 -> 116 -> -73

36 -> 62 -> 117 -> -73

36 -> 62 -> 117 -> -101

36 -> 62 -> 118 -> -73

36 -> 62 -> 120 -> -133

36 -> 62 -> 123 -> -73

36 -> 62 -> 129 -> -32

36 -> 62 -> 130 -> -73

36 -> 62 -> 137 -> -18

36 -> 62 -> 137 -> -97

36 -> 62 -> 137 -> -99

36 -> 62 -> 138 -> -20

36 -> 62 -> 138 -> -61

36 -> 62 -> 138 -> -73

36 -> 62 -> 138 -> -98

36 -> 62 -> 138 -> -133

36 -> 62 -> 139 -> -15

36 -> 62 -> 142 -> -154

36 -> 62 -> 148 -> -32

36 -> 62 -> 149 -> -73

36 -> 62 -> 150 -> -91

36 -> 62 -> 153 -> -2

36 -> 62 -> 157 -> -26

36 -> 62 -> 157 -> -28

36 -> 62 -> 157 -> -32

36 -> 62 -> 157 -> -68

36 -> 62 -> 157 -> -86

36 -> 62 -> 157 -> -99

36 -> 62 -> 157 -> -101

36 -> 62 -> 157 -> -118

36 -> 62 -> 157 -> -119

36 -> 62 -> 157 -> -120

36 -> 62 -> 157 -> -121

36 -> 62 -> 157 -> -122

36 -> 62 -> 157 -> -133

36 -> 62 -> 160 -> -129

36 -> 62 -> 162 -> -133

36 -> 62 -> 163 -> -8

36 -> 62 -> 164 -> -134

36 -> 62 -> 170 -> -138

36 -> 62 -> 171 -> -13

36 -> 62 -> 172 -> -58

36 -> 62 -> 176 -> -33

36 -> 62 -> 177 -> -106

36 -> 62 -> 179 -> -22

36 -> 62 -> 179 -> -147

36 -> 62 -> 181 -> -84

36 -> 62 -> 186 -> -99

36 -> 62 -> 188 -> -24

36 -> 62 -> 189 -> -73

36 -> 62 -> -99

36 -> 62 -> -99 -> -1

36 -> 62 -> -99 -> -3

36 -> 62 -> -99 -> -6

36 -> 62 -> -99 -> -17

36 -> 62 -> -99 -> -18

36 -> 62 -> -99 -> -19

36 -> 62 -> -99 -> -20

36 -> 62 -> -99 -> -22

36 -> 62 -> -99 -> -26

36 -> 62 -> -99 -> -27

36 -> 62 -> -99 -> -28

36 -> 62 -> -99 -> -29

36 -> 62 -> -99 -> -30

36 -> 62 -> -99 -> -32

36 -> 62 -> -99 -> -35

36 -> 62 -> -99 -> -36

36 -> 62 -> -99 -> -39

36 -> 62 -> -99 -> -41

36 -> 62 -> -99 -> -43

36 -> 62 -> -99 -> -48

36 -> 62 -> -99 -> -51

36 -> 62 -> -99 -> -56

36 -> 62 -> -99 -> -61

36 -> 62 -> -99 -> -62

36 -> 62 -> -99 -> -63

36 -> 62 -> -99 -> -64

36 -> 62 -> -99 -> -68

36 -> 62 -> -99 -> -73

36 -> 62 -> -99 -> -78

36 -> 62 -> -99 -> -81

36 -> 62 -> -99 -> -83

36 -> 62 -> -99 -> -86

36 -> 62 -> -99 -> -88

36 -> 62 -> -99 -> -89

36 -> 62 -> -99 -> -92

36 -> 62 -> -99 -> -95

36 -> 62 -> -99 -> -97

36 -> 62 -> -99 -> -98

36 -> 62 -> -99 -> -100

36 -> 62 -> -99 -> -101

36 -> 62 -> -99 -> -106

36 -> 62 -> -99 -> -107

36 -> 62 -> -99 -> -109

36 -> 62 -> -99 -> -111

36 -> 62 -> -99 -> -114

36 -> 62 -> -99 -> -117

36 -> 62 -> -99 -> -118

36 -> 62 -> -99 -> -119

36 -> 62 -> -99 -> -121

36 -> 62 -> -99 -> -122

36 -> 62 -> -99 -> -124

36 -> 62 -> -99 -> -127

36 -> 62 -> -99 -> -133

36 -> 62 -> -99 -> -137

36 -> 62 -> -99 -> -144

36 -> 62 -> -99 -> -145

36 -> 62 -> -99 -> -146

36 -> 62 -> -99 -> -147

36 -> 62 -> -99 -> -150

36 -> 62 -> -99 -> -151

36 -> 62 -> -99 -> -154

36 -> 62 -> -99 -> -155

36 -> 62 -> -99 -> -156

36 -> 63 -> 7 -> -54

36 -> 63 -> 9 -> -6

36 -> 63 -> 14 -> -54

36 -> 63 -> 15 -> -68

36 -> 63 -> 16 -> -5

36 -> 63 -> 19 -> -73

36 -> 63 -> 22 -> -152

36 -> 63 -> 24 -> -49

36 -> 63 -> 25 -> -132

36 -> 63 -> 26 -> -14

36 -> 63 -> 27 -> -19

36 -> 63 -> 27 -> -20

36 -> 63 -> 27 -> -32

36 -> 63 -> 27 -> -62

36 -> 63 -> 27 -> -68

36 -> 63 -> 27 -> -73

36 -> 63 -> 27 -> -81

36 -> 63 -> 27 -> -98

36 -> 63 -> 27 -> -99

36 -> 63 -> 27 -> -101

36 -> 63 -> 27 -> -107

36 -> 63 -> 27 -> -124

36 -> 63 -> 27 -> -133

36 -> 63 -> 27 -> -145

36 -> 63 -> 28 -> -81

36 -> 63 -> 32 -> -9

36 -> 63 -> 33 -> -145

36 -> 63 -> 34 -> -32

36 -> 63 -> 34 -> -71

36 -> 63 -> 34 -> -113

36 -> 63 -> 41 -> -6

36 -> 63 -> 42 -> -133

36 -> 63 -> 45 -> -73

36 -> 63 -> 48 -> -99

36 -> 63 -> 49 -> -15

36 -> 63 -> 55 -> -12

36 -> 63 -> 61 -> -27

36 -> 63 -> 64 -> -10

36 -> 63 -> 65 -> -4

36 -> 63 -> 66 -> -136

36 -> 63 -> 67 -> -135

36 -> 63 -> 68 -> -73

36 -> 63 -> 69 -> -77

36 -> 63 -> 71 -> -32

36 -> 63 -> 73 -> -32

36 -> 63 -> 74 -> -112

36 -> 63 -> 79 -> -73

36 -> 63 -> 84 -> -73

36 -> 63 -> 87 -> -73

36 -> 63 -> 88 -> -83

36 -> 63 -> 91 -> -101

36 -> 63 -> 97 -> -15

36 -> 63 -> 99 -> -73

36 -> 63 -> 100 -> -32

36 -> 63 -> 100 -> -38

36 -> 63 -> 108 -> -80

36 -> 63 -> 113 -> -73

36 -> 63 -> 116 -> -73

36 -> 63 -> 118 -> -73

36 -> 63 -> 120 -> -133

36 -> 63 -> 123 -> -73

36 -> 63 -> 127 -> -93

36 -> 63 -> 129 -> -32

36 -> 63 -> 130 -> -73

36 -> 63 -> 138 -> -20

36 -> 63 -> 138 -> -61

36 -> 63 -> 138 -> -73

36 -> 63 -> 138 -> -98

36 -> 63 -> 138 -> -133

36 -> 63 -> 139 -> -15

36 -> 63 -> 142 -> -154

36 -> 63 -> 148 -> -32

36 -> 63 -> 149 -> -73

36 -> 63 -> 150 -> -91

36 -> 63 -> 153 -> -2

36 -> 63 -> 155 -> -73

36 -> 63 -> 156 -> -32

36 -> 63 -> 156 -> -44

36 -> 63 -> 160 -> -129

36 -> 63 -> 162 -> -133

36 -> 63 -> 163 -> -8

36 -> 63 -> 170 -> -138

36 -> 63 -> 171 -> -13

36 -> 63 -> 172 -> -58

36 -> 63 -> 175 -> -32

36 -> 63 -> 175 -> -34

36 -> 63 -> 176 -> -33

36 -> 63 -> 177 -> -106

36 -> 63 -> 181 -> -84

36 -> 63 -> 182 -> -73

36 -> 63 -> 186 -> -99

36 -> 63 -> 188 -> -24

36 -> 63 -> 189 -> -73

36 -> 63 -> -32

36 -> 63 -> -32 -> -1

36 -> 63 -> -32 -> -3

36 -> 63 -> -32 -> -14

36 -> 63 -> -32 -> -17

36 -> 63 -> -32 -> -19

36 -> 63 -> -32 -> -20

36 -> 63 -> -32 -> -22

36 -> 63 -> -32 -> -29

36 -> 63 -> -32 -> -30

36 -> 63 -> -32 -> -34

36 -> 63 -> -32 -> -35

36 -> 63 -> -32 -> -37

36 -> 63 -> -32 -> -38

36 -> 63 -> -32 -> -39

36 -> 63 -> -32 -> -43

36 -> 63 -> -32 -> -55

36 -> 63 -> -32 -> -56

36 -> 63 -> -32 -> -62

36 -> 63 -> -32 -> -63

36 -> 63 -> -32 -> -64

36 -> 63 -> -32 -> -68

36 -> 63 -> -32 -> -71

36 -> 63 -> -32 -> -73

36 -> 63 -> -32 -> -78

36 -> 63 -> -32 -> -81

36 -> 63 -> -32 -> -86

36 -> 63 -> -32 -> -92

36 -> 63 -> -32 -> -94

36 -> 63 -> -32 -> -95

36 -> 63 -> -32 -> -98

36 -> 63 -> -32 -> -99

36 -> 63 -> -32 -> -101

36 -> 63 -> -32 -> -103

36 -> 63 -> -32 -> -105

36 -> 63 -> -32 -> -106

36 -> 63 -> -32 -> -107

36 -> 63 -> -32 -> -113

36 -> 63 -> -32 -> -117

36 -> 63 -> -32 -> -124

36 -> 63 -> -32 -> -125

36 -> 63 -> -32 -> -133

36 -> 63 -> -32 -> -137

36 -> 63 -> -32 -> -144

36 -> 63 -> -32 -> -145

36 -> 63 -> -32 -> -146

36 -> 63 -> -32 -> -147

36 -> 63 -> -32 -> -150

36 -> 63 -> -32 -> -154

36 -> 63 -> -32 -> -156

36 -> 68 -> 2 -> -32

36 -> 68 -> 4 -> -101

36 -> 68 -> 6 -> -73

36 -> 68 -> 7 -> -54

36 -> 68 -> 9 -> -6

36 -> 68 -> 12 -> -32

36 -> 68 -> 13 -> -32

36 -> 68 -> 13 -> -73

36 -> 68 -> 14 -> -54

36 -> 68 -> 15 -> -68

36 -> 68 -> 16 -> -5

36 -> 68 -> 17 -> -32

36 -> 68 -> 18 -> -73

36 -> 68 -> 18 -> -89

36 -> 68 -> 19 -> -73

36 -> 68 -> 21 -> -73

36 -> 68 -> 21 -> -101

36 -> 68 -> 21 -> -154

36 -> 68 -> 22 -> -152

36 -> 68 -> 24 -> -49

36 -> 68 -> 25 -> -132

36 -> 68 -> 26 -> -14

36 -> 68 -> 28 -> -81

36 -> 68 -> 29 -> -133

36 -> 68 -> 31 -> -1

36 -> 68 -> 32 -> -9

36 -> 68 -> 33 -> -145

36 -> 68 -> 37 -> -73

36 -> 68 -> 39 -> -101

36 -> 68 -> 40 -> -73

36 -> 68 -> 41 -> -6

36 -> 68 -> 42 -> -133

36 -> 68 -> 43 -> -73

36 -> 68 -> 43 -> -99

36 -> 68 -> 43 -> -101

36 -> 68 -> 45 -> -73

36 -> 68 -> 46 -> -32

36 -> 68 -> 48 -> -99

36 -> 68 -> 49 -> -15

36 -> 68 -> 50 -> -54

36 -> 68 -> 51 -> -2

36 -> 68 -> 52 -> -32

36 -> 68 -> 53 -> -133

36 -> 68 -> 54 -> -53

36 -> 68 -> 55 -> -12

36 -> 68 -> 56 -> -115

36 -> 68 -> 57 -> -103

36 -> 68 -> 58 -> -90

36 -> 68 -> 59 -> -148

36 -> 68 -> 60 -> -64

36 -> 68 -> 61 -> -27

36 -> 68 -> 62 -> -99

36 -> 68 -> 63 -> -32

36 -> 68 -> 64 -> -10

36 -> 68 -> 65 -> -4

36 -> 68 -> 66 -> -136

36 -> 68 -> 67 -> -135

36 -> 68 -> 69 -> -77

36 -> 68 -> 70 -> -52

36 -> 68 -> 71 -> -32

36 -> 68 -> 73 -> -32

36 -> 68 -> 74 -> -112

36 -> 68 -> 76 -> -81

36 -> 68 -> 77 -> -73

36 -> 68 -> 78 -> -73

36 -> 68 -> 79 -> -73

36 -> 68 -> 80 -> -32

36 -> 68 -> 81 -> -73

36 -> 68 -> 83 -> -125

36 -> 68 -> 84 -> -73

36 -> 68 -> 85 -> -12

36 -> 68 -> 87 -> -73

36 -> 68 -> 88 -> -83

36 -> 68 -> 90 -> -103

36 -> 68 -> 91 -> -101

36 -> 68 -> 92 -> -133

36 -> 68 -> 93 -> -145

36 -> 68 -> 94 -> -13

36 -> 68 -> 96 -> -73

36 -> 68 -> 97 -> -15

36 -> 68 -> 99 -> -73

36 -> 68 -> 101 -> -64

36 -> 68 -> 102 -> -116

36 -> 68 -> 103 -> -78

36 -> 68 -> 104 -> -2

36 -> 68 -> 106 -> -65

36 -> 68 -> 107 -> -73

36 -> 68 -> 107 -> -99

36 -> 68 -> 108 -> -80

36 -> 68 -> 110 -> -56

36 -> 68 -> 112 -> -54

36 -> 68 -> 113 -> -73

36 -> 68 -> 114 -> -73

36 -> 68 -> 116 -> -73

36 -> 68 -> 117 -> -73

36 -> 68 -> 117 -> -101

36 -> 68 -> 118 -> -73

36 -> 68 -> 120 -> -133

36 -> 68 -> 122 -> -56

36 -> 68 -> 123 -> -73

36 -> 68 -> 125 -> -104

36 -> 68 -> 126 -> -59

36 -> 68 -> 127 -> -93

36 -> 68 -> 129 -> -32

36 -> 68 -> 130 -> -73

36 -> 68 -> 132 -> -32

36 -> 68 -> 133 -> -101

36 -> 68 -> 134 -> -99

36 -> 68 -> 135 -> -21

36 -> 68 -> 135 -> -73

36 -> 68 -> 135 -> -139

36 -> 68 -> 136 -> -20

36 -> 68 -> 139 -> -15

36 -> 68 -> 142 -> -154

36 -> 68 -> 143 -> -50

36 -> 68 -> 145 -> -125

36 -> 68 -> 146 -> -20

36 -> 68 -> 147 -> -32

36 -> 68 -> 148 -> -32

36 -> 68 -> 149 -> -73

36 -> 68 -> 150 -> -91

36 -> 68 -> 151 -> -101

36 -> 68 -> 152 -> -36

36 -> 68 -> 153 -> -2

36 -> 68 -> 155 -> -73

36 -> 68 -> 158 -> -72

36 -> 68 -> 160 -> -129

36 -> 68 -> 161 -> -78

36 -> 68 -> 162 -> -133

36 -> 68 -> 163 -> -8

36 -> 68 -> 164 -> -134

36 -> 68 -> 165 -> -54

36 -> 68 -> 168 -> -73

36 -> 68 -> 168 -> -102

36 -> 68 -> 169 -> -99

36 -> 68 -> 170 -> -138

36 -> 68 -> 171 -> -13

36 -> 68 -> 172 -> -58

36 -> 68 -> 173 -> -154

36 -> 68 -> 174 -> -114

36 -> 68 -> 176 -> -33

36 -> 68 -> 177 -> -106

36 -> 68 -> 178 -> -69

36 -> 68 -> 180 -> -20

36 -> 68 -> 181 -> -84

36 -> 68 -> 182 -> -73

36 -> 68 -> 186 -> -99

36 -> 68 -> 187 -> -53

36 -> 68 -> 188 -> -24

36 -> 68 -> 189 -> -73

36 -> 68 -> -73

36 -> 68 -> -73 -> -1

36 -> 68 -> -73 -> -3

36 -> 68 -> -73 -> -6

36 -> 68 -> -73 -> -17

36 -> 68 -> -73 -> -19

36 -> 68 -> -73 -> -20

36 -> 68 -> -73 -> -22

36 -> 68 -> -73 -> -26

36 -> 68 -> -73 -> -27

36 -> 68 -> -73 -> -28

36 -> 68 -> -73 -> -29

36 -> 68 -> -73 -> -30

36 -> 68 -> -73 -> -32

36 -> 68 -> -73 -> -35

36 -> 68 -> -73 -> -39

36 -> 68 -> -73 -> -43

36 -> 68 -> -73 -> -48

36 -> 68 -> -73 -> -56

36 -> 68 -> -73 -> -61

36 -> 68 -> -73 -> -62

36 -> 68 -> -73 -> -63

36 -> 68 -> -73 -> -64

36 -> 68 -> -73 -> -68

36 -> 68 -> -73 -> -71

36 -> 68 -> -73 -> -78

36 -> 68 -> -73 -> -81

36 -> 68 -> -73 -> -83

36 -> 68 -> -73 -> -86

36 -> 68 -> -73 -> -88

36 -> 68 -> -73 -> -89

36 -> 68 -> -73 -> -92

36 -> 68 -> -73 -> -95

36 -> 68 -> -73 -> -98

36 -> 68 -> -73 -> -99

36 -> 68 -> -73 -> -101

36 -> 68 -> -73 -> -106

36 -> 68 -> -73 -> -107

36 -> 68 -> -73 -> -113

36 -> 68 -> -73 -> -114

36 -> 68 -> -73 -> -117

36 -> 68 -> -73 -> -118

36 -> 68 -> -73 -> -119

36 -> 68 -> -73 -> -121

36 -> 68 -> -73 -> -122

36 -> 68 -> -73 -> -124

36 -> 68 -> -73 -> -133

36 -> 68 -> -73 -> -137

36 -> 68 -> -73 -> -144

36 -> 68 -> -73 -> -145

36 -> 68 -> -73 -> -146

36 -> 68 -> -73 -> -147

36 -> 68 -> -73 -> -150

36 -> 68 -> -73 -> -154

36 -> 68 -> -73 -> -155

36 -> 68 -> -73 -> -156

36 -> 75 -> 3 -> -32

36 -> 75 -> 3 -> -42

36 -> 75 -> 4 -> -101

36 -> 75 -> 6 -> -73

36 -> 75 -> 16 -> -5

36 -> 75 -> 21 -> -73

36 -> 75 -> 21 -> -101

36 -> 75 -> 21 -> -154

36 -> 75 -> 27 -> -19

36 -> 75 -> 27 -> -20

36 -> 75 -> 27 -> -32

36 -> 75 -> 27 -> -62

36 -> 75 -> 27 -> -68

36 -> 75 -> 27 -> -73

36 -> 75 -> 27 -> -81

36 -> 75 -> 27 -> -98

36 -> 75 -> 27 -> -99

36 -> 75 -> 27 -> -101

36 -> 75 -> 27 -> -107

36 -> 75 -> 27 -> -124

36 -> 75 -> 27 -> -133

36 -> 75 -> 27 -> -145

36 -> 75 -> 29 -> -133

36 -> 75 -> 30 -> -1

36 -> 75 -> 30 -> -3

36 -> 75 -> 30 -> -32

36 -> 75 -> 30 -> -39

36 -> 75 -> 30 -> -63

36 -> 75 -> 30 -> -73

36 -> 75 -> 30 -> -81

36 -> 75 -> 30 -> -99

36 -> 75 -> 30 -> -101

36 -> 75 -> 30 -> -106

36 -> 75 -> 30 -> -117

36 -> 75 -> 30 -> -133

36 -> 75 -> 30 -> -137

36 -> 75 -> 30 -> -150

36 -> 75 -> 30 -> -156

36 -> 75 -> 34 -> -32

36 -> 75 -> 34 -> -71

36 -> 75 -> 34 -> -113

36 -> 75 -> 39 -> -101

36 -> 75 -> 43 -> -73

36 -> 75 -> 43 -> -99

36 -> 75 -> 43 -> -101

36 -> 75 -> 60 -> -64

36 -> 75 -> 61 -> -27

36 -> 75 -> 62 -> -99

36 -> 75 -> 76 -> -81

36 -> 75 -> 77 -> -73

36 -> 75 -> 92 -> -133

36 -> 75 -> 103 -> -78

36 -> 75 -> 105 -> -32

36 -> 75 -> 105 -> -146

36 -> 75 -> 107 -> -73

36 -> 75 -> 107 -> -99

36 -> 75 -> 117 -> -73

36 -> 75 -> 117 -> -101

36 -> 75 -> 122 -> -56

36 -> 75 -> 128 -> -32

36 -> 75 -> 128 -> -95

36 -> 75 -> 129 -> -32

36 -> 75 -> 136 -> -20

36 -> 75 -> 138 -> -20

36 -> 75 -> 138 -> -61

36 -> 75 -> 138 -> -73

36 -> 75 -> 138 -> -98

36 -> 75 -> 138 -> -133

36 -> 75 -> 140 -> -99

36 -> 75 -> 140 -> -133

36 -> 75 -> 157 -> -26

36 -> 75 -> 157 -> -28

36 -> 75 -> 157 -> -32

36 -> 75 -> 157 -> -68

36 -> 75 -> 157 -> -86

36 -> 75 -> 157 -> -99

36 -> 75 -> 157 -> -101

36 -> 75 -> 157 -> -118

36 -> 75 -> 157 -> -119

36 -> 75 -> 157 -> -120

36 -> 75 -> 157 -> -121

36 -> 75 -> 157 -> -122

36 -> 75 -> 157 -> -133

36 -> 75 -> 161 -> -78

36 -> 75 -> 179 -> -22

36 -> 75 -> 179 -> -147

36 -> 75 -> 180 -> -20

36 -> 75 -> 186 -> -99

36 -> 75 -> -1

36 -> 75 -> -1 -> -2

36 -> 75 -> -1 -> -3

36 -> 75 -> -1 -> -6

36 -> 75 -> -1 -> -12

36 -> 75 -> -1 -> -19

36 -> 75 -> -1 -> -20

36 -> 75 -> -1 -> -22

36 -> 75 -> -1 -> -26

36 -> 75 -> -1 -> -27

36 -> 75 -> -1 -> -28

36 -> 75 -> -1 -> -29

36 -> 75 -> -1 -> -30

36 -> 75 -> -1 -> -32

36 -> 75 -> -1 -> -35

36 -> 75 -> -1 -> -36

36 -> 75 -> -1 -> -39

36 -> 75 -> -1 -> -42

36 -> 75 -> -1 -> -48

36 -> 75 -> -1 -> -51

36 -> 75 -> -1 -> -55

36 -> 75 -> -1 -> -56

36 -> 75 -> -1 -> -62

36 -> 75 -> -1 -> -63

36 -> 75 -> -1 -> -64

36 -> 75 -> -1 -> -68

36 -> 75 -> -1 -> -71

36 -> 75 -> -1 -> -73

36 -> 75 -> -1 -> -76

36 -> 75 -> -1 -> -78

36 -> 75 -> -1 -> -80

36 -> 75 -> -1 -> -81

36 -> 75 -> -1 -> -83

36 -> 75 -> -1 -> -86

36 -> 75 -> -1 -> -88

36 -> 75 -> -1 -> -89

36 -> 75 -> -1 -> -95

36 -> 75 -> -1 -> -98

36 -> 75 -> -1 -> -99

36 -> 75 -> -1 -> -100

36 -> 75 -> -1 -> -101

36 -> 75 -> -1 -> -105

36 -> 75 -> -1 -> -106

36 -> 75 -> -1 -> -107

36 -> 75 -> -1 -> -112

36 -> 75 -> -1 -> -113

36 -> 75 -> -1 -> -114

36 -> 75 -> -1 -> -116

36 -> 75 -> -1 -> -117

36 -> 75 -> -1 -> -118

36 -> 75 -> -1 -> -119

36 -> 75 -> -1 -> -121

36 -> 75 -> -1 -> -122

36 -> 75 -> -1 -> -124

36 -> 75 -> -1 -> -127

36 -> 75 -> -1 -> -133

36 -> 75 -> -1 -> -137

36 -> 75 -> -1 -> -145

36 -> 75 -> -1 -> -146

36 -> 75 -> -1 -> -147

36 -> 75 -> -1 -> -150

36 -> 75 -> -1 -> -154

36 -> 75 -> -1 -> -155

36 -> 75 -> -1 -> -156

36 -> 75 -> -29

36 -> 75 -> -29 -> -1

36 -> 75 -> -29 -> -3

36 -> 75 -> -29 -> -4

36 -> 75 -> -29 -> -6

36 -> 75 -> -29 -> -8

36 -> 75 -> -29 -> -9

36 -> 75 -> -29 -> -10

36 -> 75 -> -29 -> -19

36 -> 75 -> -29 -> -20

36 -> 75 -> -29 -> -22

36 -> 75 -> -29 -> -24

36 -> 75 -> -29 -> -26

36 -> 75 -> -29 -> -27

36 -> 75 -> -29 -> -28

36 -> 75 -> -29 -> -30

36 -> 75 -> -29 -> -31

36 -> 75 -> -29 -> -32

36 -> 75 -> -29 -> -33

36 -> 75 -> -29 -> -35

36 -> 75 -> -29 -> -39

36 -> 75 -> -29 -> -44

36 -> 75 -> -29 -> -45

36 -> 75 -> -29 -> -46

36 -> 75 -> -29 -> -47

36 -> 75 -> -29 -> -48

36 -> 75 -> -29 -> -49

36 -> 75 -> -29 -> -51

36 -> 75 -> -29 -> -56

36 -> 75 -> -29 -> -58

36 -> 75 -> -29 -> -60

36 -> 75 -> -29 -> -61

36 -> 75 -> -29 -> -62

36 -> 75 -> -29 -> -63

36 -> 75 -> -29 -> -64

36 -> 75 -> -29 -> -66

36 -> 75 -> -29 -> -68

36 -> 75 -> -29 -> -69

36 -> 75 -> -29 -> -73

36 -> 75 -> -29 -> -77

36 -> 75 -> -29 -> -78

36 -> 75 -> -29 -> -81

36 -> 75 -> -29 -> -83

36 -> 75 -> -29 -> -84

36 -> 75 -> -29 -> -86

36 -> 75 -> -29 -> -88

36 -> 75 -> -29 -> -89

36 -> 75 -> -29 -> -91

36 -> 75 -> -29 -> -95

36 -> 75 -> -29 -> -96

36 -> 75 -> -29 -> -98

36 -> 75 -> -29 -> -99

36 -> 75 -> -29 -> -100

36 -> 75 -> -29 -> -101

36 -> 75 -> -29 -> -106

36 -> 75 -> -29 -> -107

36 -> 75 -> -29 -> -114

36 -> 75 -> -29 -> -117

36 -> 75 -> -29 -> -118

36 -> 75 -> -29 -> -119

36 -> 75 -> -29 -> -120

36 -> 75 -> -29 -> -121

36 -> 75 -> -29 -> -122

36 -> 75 -> -29 -> -124

36 -> 75 -> -29 -> -127

36 -> 75 -> -29 -> -131

36 -> 75 -> -29 -> -132

36 -> 75 -> -29 -> -133

36 -> 75 -> -29 -> -135

36 -> 75 -> -29 -> -136

36 -> 75 -> -29 -> -137

36 -> 75 -> -29 -> -141

36 -> 75 -> -29 -> -145

36 -> 75 -> -29 -> -146

36 -> 75 -> -29 -> -147

36 -> 75 -> -29 -> -150

36 -> 75 -> -29 -> -154

36 -> 75 -> -29 -> -155

36 -> 75 -> -29 -> -156

36 -> 76 -> 3 -> -32

36 -> 76 -> 3 -> -42

36 -> 76 -> 4 -> -101

36 -> 76 -> 6 -> -73

36 -> 76 -> 7 -> -54

36 -> 76 -> 9 -> -6

36 -> 76 -> 14 -> -54

36 -> 76 -> 15 -> -68

36 -> 76 -> 16 -> -5

36 -> 76 -> 19 -> -73

36 -> 76 -> 21 -> -73

36 -> 76 -> 21 -> -101

36 -> 76 -> 21 -> -154

36 -> 76 -> 22 -> -152

36 -> 76 -> 24 -> -49

36 -> 76 -> 25 -> -132

36 -> 76 -> 27 -> -19

36 -> 76 -> 27 -> -20

36 -> 76 -> 27 -> -32

36 -> 76 -> 27 -> -62

36 -> 76 -> 27 -> -68

36 -> 76 -> 27 -> -73

36 -> 76 -> 27 -> -81

36 -> 76 -> 27 -> -98

36 -> 76 -> 27 -> -99

36 -> 76 -> 27 -> -101

36 -> 76 -> 27 -> -107

36 -> 76 -> 27 -> -124

36 -> 76 -> 27 -> -133

36 -> 76 -> 27 -> -145

36 -> 76 -> 28 -> -81

36 -> 76 -> 29 -> -133

36 -> 76 -> 30 -> -1

36 -> 76 -> 30 -> -3

36 -> 76 -> 30 -> -32

36 -> 76 -> 30 -> -39

36 -> 76 -> 30 -> -63

36 -> 76 -> 30 -> -73

36 -> 76 -> 30 -> -81

36 -> 76 -> 30 -> -99

36 -> 76 -> 30 -> -101

36 -> 76 -> 30 -> -106

36 -> 76 -> 30 -> -117

36 -> 76 -> 30 -> -133

36 -> 76 -> 30 -> -137

36 -> 76 -> 30 -> -150

36 -> 76 -> 30 -> -156

36 -> 76 -> 32 -> -9

36 -> 76 -> 33 -> -145

36 -> 76 -> 34 -> -32

36 -> 76 -> 34 -> -71

36 -> 76 -> 34 -> -113

36 -> 76 -> 38 -> -55

36 -> 76 -> 38 -> -56

36 -> 76 -> 38 -> -105

36 -> 76 -> 39 -> -101

36 -> 76 -> 40 -> -73

36 -> 76 -> 41 -> -6

36 -> 76 -> 42 -> -133

36 -> 76 -> 43 -> -73

36 -> 76 -> 43 -> -99

36 -> 76 -> 43 -> -101

36 -> 76 -> 44 -> -29

36 -> 76 -> 44 -> -48

36 -> 76 -> 44 -> -81

36 -> 76 -> 44 -> -89

36 -> 76 -> 44 -> -99

36 -> 76 -> 44 -> -114

36 -> 76 -> 45 -> -73

36 -> 76 -> 48 -> -99

36 -> 76 -> 49 -> -15

36 -> 76 -> 52 -> -32

36 -> 76 -> 55 -> -12

36 -> 76 -> 60 -> -64

36 -> 76 -> 61 -> -27

36 -> 76 -> 64 -> -10

36 -> 76 -> 65 -> -4

36 -> 76 -> 66 -> -136

36 -> 76 -> 67 -> -135

36 -> 76 -> 68 -> -73

36 -> 76 -> 69 -> -77

36 -> 76 -> 73 -> -32

36 -> 76 -> 74 -> -112

36 -> 76 -> 75 -> -1

36 -> 76 -> 75 -> -29

36 -> 76 -> 77 -> -73

36 -> 76 -> 79 -> -73

36 -> 76 -> 84 -> -73

36 -> 76 -> 87 -> -73

36 -> 76 -> 88 -> -83

36 -> 76 -> 91 -> -101

36 -> 76 -> 92 -> -133

36 -> 76 -> 97 -> -15

36 -> 76 -> 99 -> -73

36 -> 76 -> 102 -> -116

36 -> 76 -> 103 -> -78

36 -> 76 -> 105 -> -32

36 -> 76 -> 105 -> -146

36 -> 76 -> 107 -> -73

36 -> 76 -> 107 -> -99

36 -> 76 -> 108 -> -80

36 -> 76 -> 109 -> -20

36 -> 76 -> 109 -> -73

36 -> 76 -> 109 -> -99

36 -> 76 -> 109 -> -101

36 -> 76 -> 110 -> -56

36 -> 76 -> 113 -> -73

36 -> 76 -> 115 -> -17

36 -> 76 -> 115 -> -32

36 -> 76 -> 115 -> -68

36 -> 76 -> 115 -> -73

36 -> 76 -> 115 -> -92

36 -> 76 -> 116 -> -73

36 -> 76 -> 117 -> -73

36 -> 76 -> 117 -> -101

36 -> 76 -> 118 -> -73

36 -> 76 -> 120 -> -133

36 -> 76 -> 122 -> -56

36 -> 76 -> 123 -> -73

36 -> 76 -> 128 -> -32

36 -> 76 -> 128 -> -95

36 -> 76 -> 129 -> -32

36 -> 76 -> 130 -> -73

36 -> 76 -> 134 -> -99

36 -> 76 -> 136 -> -20

36 -> 76 -> 138 -> -20

36 -> 76 -> 138 -> -61

36 -> 76 -> 138 -> -73

36 -> 76 -> 138 -> -98

36 -> 76 -> 138 -> -133

36 -> 76 -> 139 -> -15

36 -> 76 -> 140 -> -99

36 -> 76 -> 140 -> -133

36 -> 76 -> 142 -> -154

36 -> 76 -> 146 -> -20

36 -> 76 -> 148 -> -32

36 -> 76 -> 149 -> -73

36 -> 76 -> 150 -> -91

36 -> 76 -> 152 -> -36

36 -> 76 -> 153 -> -2

36 -> 76 -> 157 -> -26

36 -> 76 -> 157 -> -28

36 -> 76 -> 157 -> -32

36 -> 76 -> 157 -> -68

36 -> 76 -> 157 -> -86

36 -> 76 -> 157 -> -99

36 -> 76 -> 157 -> -101

36 -> 76 -> 157 -> -118

36 -> 76 -> 157 -> -119

36 -> 76 -> 157 -> -120

36 -> 76 -> 157 -> -121

36 -> 76 -> 157 -> -122

36 -> 76 -> 157 -> -133

36 -> 76 -> 160 -> -129

36 -> 76 -> 161 -> -78

36 -> 76 -> 162 -> -133

36 -> 76 -> 163 -> -8

36 -> 76 -> 170 -> -138

36 -> 76 -> 171 -> -13

36 -> 76 -> 172 -> -58

36 -> 76 -> 176 -> -33

36 -> 76 -> 177 -> -106

36 -> 76 -> 179 -> -22

36 -> 76 -> 179 -> -147

36 -> 76 -> 180 -> -20

36 -> 76 -> 181 -> -84

36 -> 76 -> 182 -> -73

36 -> 76 -> 184 -> -86

36 -> 76 -> 184 -> -101

36 -> 76 -> 184 -> -133

36 -> 76 -> 186 -> -99

36 -> 76 -> 188 -> -24

36 -> 76 -> 189 -> -73

36 -> 76 -> -81

36 -> 76 -> -81 -> -1

36 -> 76 -> -81 -> -3

36 -> 76 -> -81 -> -6

36 -> 76 -> -81 -> -7

36 -> 76 -> -81 -> -17

36 -> 76 -> -81 -> -18

36 -> 76 -> -81 -> -19

36 -> 76 -> -81 -> -20

36 -> 76 -> -81 -> -22

36 -> 76 -> -81 -> -26

36 -> 76 -> -81 -> -27

36 -> 76 -> -81 -> -28

36 -> 76 -> -81 -> -29

36 -> 76 -> -81 -> -30

36 -> 76 -> -81 -> -32

36 -> 76 -> -81 -> -35

36 -> 76 -> -81 -> -36

36 -> 76 -> -81 -> -39

36 -> 76 -> -81 -> -42

36 -> 76 -> -81 -> -43

36 -> 76 -> -81 -> -48

36 -> 76 -> -81 -> -51

36 -> 76 -> -81 -> -55

36 -> 76 -> -81 -> -56

36 -> 76 -> -81 -> -61

36 -> 76 -> -81 -> -62

36 -> 76 -> -81 -> -63

36 -> 76 -> -81 -> -64

36 -> 76 -> -81 -> -68

36 -> 76 -> -81 -> -71

36 -> 76 -> -81 -> -73

36 -> 76 -> -81 -> -78

36 -> 76 -> -81 -> -83

36 -> 76 -> -81 -> -86

36 -> 76 -> -81 -> -88

36 -> 76 -> -81 -> -89

36 -> 76 -> -81 -> -92

36 -> 76 -> -81 -> -95

36 -> 76 -> -81 -> -97

36 -> 76 -> -81 -> -98

36 -> 76 -> -81 -> -99

36 -> 76 -> -81 -> -100

36 -> 76 -> -81 -> -101

36 -> 76 -> -81 -> -105

36 -> 76 -> -81 -> -106

36 -> 76 -> -81 -> -107

36 -> 76 -> -81 -> -110

36 -> 76 -> -81 -> -113

36 -> 76 -> -81 -> -114

36 -> 76 -> -81 -> -116

36 -> 76 -> -81 -> -117

36 -> 76 -> -81 -> -118

36 -> 76 -> -81 -> -119

36 -> 76 -> -81 -> -121

36 -> 76 -> -81 -> -122

36 -> 76 -> -81 -> -124

36 -> 76 -> -81 -> -127

36 -> 76 -> -81 -> -130

36 -> 76 -> -81 -> -133

36 -> 76 -> -81 -> -134

36 -> 76 -> -81 -> -137

36 -> 76 -> -81 -> -144

36 -> 76 -> -81 -> -145

36 -> 76 -> -81 -> -146

36 -> 76 -> -81 -> -147

36 -> 76 -> -81 -> -150

36 -> 76 -> -81 -> -154

36 -> 76 -> -81 -> -155

36 -> 76 -> -81 -> -156

36 -> 77 -> 3 -> -32

36 -> 77 -> 3 -> -42

36 -> 77 -> 4 -> -101

36 -> 77 -> 6 -> -73

36 -> 77 -> 7 -> -54

36 -> 77 -> 9 -> -6

36 -> 77 -> 14 -> -54

36 -> 77 -> 15 -> -68

36 -> 77 -> 17 -> -32

36 -> 77 -> 19 -> -73

36 -> 77 -> 21 -> -73

36 -> 77 -> 21 -> -101

36 -> 77 -> 21 -> -154

36 -> 77 -> 22 -> -152

36 -> 77 -> 24 -> -49

36 -> 77 -> 25 -> -132

36 -> 77 -> 27 -> -19

36 -> 77 -> 27 -> -20

36 -> 77 -> 27 -> -32

36 -> 77 -> 27 -> -62

36 -> 77 -> 27 -> -68

36 -> 77 -> 27 -> -73

36 -> 77 -> 27 -> -81

36 -> 77 -> 27 -> -98

36 -> 77 -> 27 -> -99

36 -> 77 -> 27 -> -101

36 -> 77 -> 27 -> -107

36 -> 77 -> 27 -> -124

36 -> 77 -> 27 -> -133

36 -> 77 -> 27 -> -145

36 -> 77 -> 28 -> -81

36 -> 77 -> 29 -> -133

36 -> 77 -> 30 -> -1

36 -> 77 -> 30 -> -3

36 -> 77 -> 30 -> -32

36 -> 77 -> 30 -> -39

36 -> 77 -> 30 -> -63

36 -> 77 -> 30 -> -73

36 -> 77 -> 30 -> -81

36 -> 77 -> 30 -> -99

36 -> 77 -> 30 -> -101

36 -> 77 -> 30 -> -106

36 -> 77 -> 30 -> -117

36 -> 77 -> 30 -> -133

36 -> 77 -> 30 -> -137

36 -> 77 -> 30 -> -150

36 -> 77 -> 30 -> -156

36 -> 77 -> 32 -> -9

36 -> 77 -> 33 -> -145

36 -> 77 -> 37 -> -73

36 -> 77 -> 38 -> -55

36 -> 77 -> 38 -> -56

36 -> 77 -> 38 -> -105

36 -> 77 -> 39 -> -101

36 -> 77 -> 40 -> -73

36 -> 77 -> 41 -> -6

36 -> 77 -> 42 -> -133

36 -> 77 -> 43 -> -73

36 -> 77 -> 43 -> -99

36 -> 77 -> 43 -> -101

36 -> 77 -> 44 -> -29

36 -> 77 -> 44 -> -48

36 -> 77 -> 44 -> -81

36 -> 77 -> 44 -> -89

36 -> 77 -> 44 -> -99

36 -> 77 -> 44 -> -114

36 -> 77 -> 45 -> -73

36 -> 77 -> 48 -> -99

36 -> 77 -> 49 -> -15

36 -> 77 -> 55 -> -12

36 -> 77 -> 60 -> -64

36 -> 77 -> 61 -> -27

36 -> 77 -> 62 -> -99

36 -> 77 -> 64 -> -10

36 -> 77 -> 65 -> -4

36 -> 77 -> 66 -> -136

36 -> 77 -> 67 -> -135

36 -> 77 -> 68 -> -73

36 -> 77 -> 69 -> -77

36 -> 77 -> 73 -> -32

36 -> 77 -> 74 -> -112

36 -> 77 -> 75 -> -1

36 -> 77 -> 75 -> -29

36 -> 77 -> 76 -> -81

36 -> 77 -> 78 -> -73

36 -> 77 -> 79 -> -73

36 -> 77 -> 81 -> -73

36 -> 77 -> 82 -> -30

36 -> 77 -> 82 -> -32

36 -> 77 -> 82 -> -35

36 -> 77 -> 82 -> -101

36 -> 77 -> 83 -> -125

36 -> 77 -> 84 -> -73

36 -> 77 -> 87 -> -73

36 -> 77 -> 88 -> -83

36 -> 77 -> 89 -> -6

36 -> 77 -> 89 -> -73

36 -> 77 -> 89 -> -81

36 -> 77 -> 89 -> -83

36 -> 77 -> 89 -> -86

36 -> 77 -> 89 -> -99

36 -> 77 -> 89 -> -101

36 -> 77 -> 89 -> -114

36 -> 77 -> 89 -> -155

36 -> 77 -> 91 -> -101

36 -> 77 -> 92 -> -133

36 -> 77 -> 97 -> -15

36 -> 77 -> 101 -> -64

36 -> 77 -> 102 -> -116

36 -> 77 -> 103 -> -78

36 -> 77 -> 105 -> -32

36 -> 77 -> 105 -> -146

36 -> 77 -> 107 -> -73

36 -> 77 -> 107 -> -99

36 -> 77 -> 108 -> -80

36 -> 77 -> 109 -> -20

36 -> 77 -> 109 -> -73

36 -> 77 -> 109 -> -99

36 -> 77 -> 109 -> -101

36 -> 77 -> 110 -> -56

36 -> 77 -> 113 -> -73

36 -> 77 -> 114 -> -73

36 -> 77 -> 115 -> -17

36 -> 77 -> 115 -> -32

36 -> 77 -> 115 -> -68

36 -> 77 -> 115 -> -73

36 -> 77 -> 115 -> -92

36 -> 77 -> 116 -> -73

36 -> 77 -> 117 -> -73

36 -> 77 -> 117 -> -101

36 -> 77 -> 118 -> -73

36 -> 77 -> 120 -> -133

36 -> 77 -> 122 -> -56

36 -> 77 -> 123 -> -73

36 -> 77 -> 128 -> -32

36 -> 77 -> 128 -> -95

36 -> 77 -> 129 -> -32

36 -> 77 -> 130 -> -73

36 -> 77 -> 131 -> -41

36 -> 77 -> 131 -> -99

36 -> 77 -> 131 -> -101

36 -> 77 -> 131 -> -109

36 -> 77 -> 131 -> -111

36 -> 77 -> 131 -> -151

36 -> 77 -> 133 -> -101

36 -> 77 -> 134 -> -99

36 -> 77 -> 136 -> -20

36 -> 77 -> 137 -> -18

36 -> 77 -> 137 -> -97

36 -> 77 -> 137 -> -99

36 -> 77 -> 138 -> -20

36 -> 77 -> 138 -> -61

36 -> 77 -> 138 -> -73

36 -> 77 -> 138 -> -98

36 -> 77 -> 138 -> -133

36 -> 77 -> 139 -> -15

36 -> 77 -> 140 -> -99

36 -> 77 -> 140 -> -133

36 -> 77 -> 141 -> -51

36 -> 77 -> 141 -> -100

36 -> 77 -> 141 -> -127

36 -> 77 -> 142 -> -154

36 -> 77 -> 144 -> -7

36 -> 77 -> 144 -> -110

36 -> 77 -> 144 -> -130

36 -> 77 -> 146 -> -20

36 -> 77 -> 148 -> -32

36 -> 77 -> 149 -> -73

36 -> 77 -> 150 -> -91

36 -> 77 -> 151 -> -101

36 -> 77 -> 152 -> -36

36 -> 77 -> 153 -> -2

36 -> 77 -> 157 -> -26

36 -> 77 -> 157 -> -28

36 -> 77 -> 157 -> -32

36 -> 77 -> 157 -> -68

36 -> 77 -> 157 -> -86

36 -> 77 -> 157 -> -99

36 -> 77 -> 157 -> -101

36 -> 77 -> 157 -> -118

36 -> 77 -> 157 -> -119

36 -> 77 -> 157 -> -120

36 -> 77 -> 157 -> -121

36 -> 77 -> 157 -> -122

36 -> 77 -> 157 -> -133

36 -> 77 -> 160 -> -129

36 -> 77 -> 161 -> -78

36 -> 77 -> 162 -> -133

36 -> 77 -> 163 -> -8

36 -> 77 -> 164 -> -134

36 -> 77 -> 168 -> -73

36 -> 77 -> 168 -> -102

36 -> 77 -> 169 -> -99

36 -> 77 -> 170 -> -138

36 -> 77 -> 171 -> -13

36 -> 77 -> 172 -> -58

36 -> 77 -> 173 -> -154

36 -> 77 -> 174 -> -114

36 -> 77 -> 176 -> -33

36 -> 77 -> 177 -> -106

36 -> 77 -> 179 -> -22

36 -> 77 -> 179 -> -147

36 -> 77 -> 180 -> -20

36 -> 77 -> 181 -> -84

36 -> 77 -> 182 -> -73

36 -> 77 -> 186 -> -99

36 -> 77 -> 188 -> -24

36 -> 77 -> 189 -> -73

36 -> 77 -> 190 -> -43

36 -> 77 -> 190 -> -73

36 -> 77 -> 190 -> -86

36 -> 77 -> 190 -> -88

36 -> 77 -> 190 -> -144

36 -> 77 -> -73

36 -> 77 -> -73 -> -1

36 -> 77 -> -73 -> -3

36 -> 77 -> -73 -> -6

36 -> 77 -> -73 -> -17

36 -> 77 -> -73 -> -19

36 -> 77 -> -73 -> -20

36 -> 77 -> -73 -> -22

36 -> 77 -> -73 -> -26

36 -> 77 -> -73 -> -27

36 -> 77 -> -73 -> -28

36 -> 77 -> -73 -> -29

36 -> 77 -> -73 -> -30

36 -> 77 -> -73 -> -32

36 -> 77 -> -73 -> -35

36 -> 77 -> -73 -> -39

36 -> 77 -> -73 -> -43

36 -> 77 -> -73 -> -48

36 -> 77 -> -73 -> -56

36 -> 77 -> -73 -> -61

36 -> 77 -> -73 -> -62

36 -> 77 -> -73 -> -63

36 -> 77 -> -73 -> -64

36 -> 77 -> -73 -> -68

36 -> 77 -> -73 -> -71

36 -> 77 -> -73 -> -78

36 -> 77 -> -73 -> -81

36 -> 77 -> -73 -> -83

36 -> 77 -> -73 -> -86

36 -> 77 -> -73 -> -88

36 -> 77 -> -73 -> -89

36 -> 77 -> -73 -> -92

36 -> 77 -> -73 -> -95

36 -> 77 -> -73 -> -98

36 -> 77 -> -73 -> -99

36 -> 77 -> -73 -> -101

36 -> 77 -> -73 -> -106

36 -> 77 -> -73 -> -107

36 -> 77 -> -73 -> -113

36 -> 77 -> -73 -> -114

36 -> 77 -> -73 -> -117

36 -> 77 -> -73 -> -118

36 -> 77 -> -73 -> -119

36 -> 77 -> -73 -> -121

36 -> 77 -> -73 -> -122

36 -> 77 -> -73 -> -124

36 -> 77 -> -73 -> -133

36 -> 77 -> -73 -> -137

36 -> 77 -> -73 -> -144

36 -> 77 -> -73 -> -145

36 -> 77 -> -73 -> -146

36 -> 77 -> -73 -> -147

36 -> 77 -> -73 -> -150

36 -> 77 -> -73 -> -154

36 -> 77 -> -73 -> -155

36 -> 77 -> -73 -> -156

36 -> 78 -> 6 -> -73

36 -> 78 -> 7 -> -54

36 -> 78 -> 9 -> -6

36 -> 78 -> 14 -> -54

36 -> 78 -> 15 -> -68

36 -> 78 -> 19 -> -73

36 -> 78 -> 22 -> -152

36 -> 78 -> 24 -> -49

36 -> 78 -> 25 -> -132

36 -> 78 -> 27 -> -19

36 -> 78 -> 27 -> -20

36 -> 78 -> 27 -> -32

36 -> 78 -> 27 -> -62

36 -> 78 -> 27 -> -68

36 -> 78 -> 27 -> -73

36 -> 78 -> 27 -> -81

36 -> 78 -> 27 -> -98

36 -> 78 -> 27 -> -99

36 -> 78 -> 27 -> -101

36 -> 78 -> 27 -> -107

36 -> 78 -> 27 -> -124

36 -> 78 -> 27 -> -133

36 -> 78 -> 27 -> -145

36 -> 78 -> 28 -> -81

36 -> 78 -> 30 -> -1

36 -> 78 -> 30 -> -3

36 -> 78 -> 30 -> -32

36 -> 78 -> 30 -> -39

36 -> 78 -> 30 -> -63

36 -> 78 -> 30 -> -73

36 -> 78 -> 30 -> -81

36 -> 78 -> 30 -> -99

36 -> 78 -> 30 -> -101

36 -> 78 -> 30 -> -106

36 -> 78 -> 30 -> -117

36 -> 78 -> 30 -> -133

36 -> 78 -> 30 -> -137

36 -> 78 -> 30 -> -150

36 -> 78 -> 30 -> -156

36 -> 78 -> 32 -> -9

36 -> 78 -> 33 -> -145

36 -> 78 -> 37 -> -73

36 -> 78 -> 41 -> -6

36 -> 78 -> 42 -> -133

36 -> 78 -> 43 -> -73

36 -> 78 -> 43 -> -99

36 -> 78 -> 43 -> -101

36 -> 78 -> 45 -> -73

36 -> 78 -> 48 -> -99

36 -> 78 -> 49 -> -15

36 -> 78 -> 55 -> -12

36 -> 78 -> 61 -> -27

36 -> 78 -> 62 -> -99

36 -> 78 -> 64 -> -10

36 -> 78 -> 65 -> -4

36 -> 78 -> 66 -> -136

36 -> 78 -> 67 -> -135

36 -> 78 -> 68 -> -73

36 -> 78 -> 69 -> -77

36 -> 78 -> 73 -> -32

36 -> 78 -> 74 -> -112

36 -> 78 -> 77 -> -73

36 -> 78 -> 79 -> -73

36 -> 78 -> 84 -> -73

36 -> 78 -> 87 -> -73

36 -> 78 -> 88 -> -83

36 -> 78 -> 89 -> -6

36 -> 78 -> 89 -> -73

36 -> 78 -> 89 -> -81

36 -> 78 -> 89 -> -83

36 -> 78 -> 89 -> -86

36 -> 78 -> 89 -> -99

36 -> 78 -> 89 -> -101

36 -> 78 -> 89 -> -114

36 -> 78 -> 89 -> -155

36 -> 78 -> 91 -> -101

36 -> 78 -> 97 -> -15

36 -> 78 -> 108 -> -80

36 -> 78 -> 113 -> -73

36 -> 78 -> 115 -> -17

36 -> 78 -> 115 -> -32

36 -> 78 -> 115 -> -68

36 -> 78 -> 115 -> -73

36 -> 78 -> 115 -> -92

36 -> 78 -> 116 -> -73

36 -> 78 -> 118 -> -73

36 -> 78 -> 120 -> -133

36 -> 78 -> 123 -> -73

36 -> 78 -> 129 -> -32

36 -> 78 -> 130 -> -73

36 -> 78 -> 138 -> -20

36 -> 78 -> 138 -> -61

36 -> 78 -> 138 -> -73

36 -> 78 -> 138 -> -98

36 -> 78 -> 138 -> -133

36 -> 78 -> 139 -> -15

36 -> 78 -> 140 -> -99

36 -> 78 -> 140 -> -133

36 -> 78 -> 141 -> -51

36 -> 78 -> 141 -> -100

36 -> 78 -> 141 -> -127

36 -> 78 -> 142 -> -154

36 -> 78 -> 144 -> -7

36 -> 78 -> 144 -> -110

36 -> 78 -> 144 -> -130

36 -> 78 -> 148 -> -32

36 -> 78 -> 149 -> -73

36 -> 78 -> 150 -> -91

36 -> 78 -> 153 -> -2

36 -> 78 -> 157 -> -26

36 -> 78 -> 157 -> -28

36 -> 78 -> 157 -> -32

36 -> 78 -> 157 -> -68

36 -> 78 -> 157 -> -86

36 -> 78 -> 157 -> -99

36 -> 78 -> 157 -> -101

36 -> 78 -> 157 -> -118

36 -> 78 -> 157 -> -119

36 -> 78 -> 157 -> -120

36 -> 78 -> 157 -> -121

36 -> 78 -> 157 -> -122

36 -> 78 -> 157 -> -133

36 -> 78 -> 160 -> -129

36 -> 78 -> 162 -> -133

36 -> 78 -> 163 -> -8

36 -> 78 -> 168 -> -73

36 -> 78 -> 168 -> -102

36 -> 78 -> 169 -> -99

36 -> 78 -> 170 -> -138

36 -> 78 -> 171 -> -13

36 -> 78 -> 172 -> -58

36 -> 78 -> 176 -> -33

36 -> 78 -> 177 -> -106

36 -> 78 -> 179 -> -22

36 -> 78 -> 179 -> -147

36 -> 78 -> 181 -> -84

36 -> 78 -> 182 -> -73

36 -> 78 -> 186 -> -99

36 -> 78 -> 188 -> -24

36 -> 78 -> 189 -> -73

36 -> 78 -> 190 -> -43

36 -> 78 -> 190 -> -73

36 -> 78 -> 190 -> -86

36 -> 78 -> 190 -> -88

36 -> 78 -> 190 -> -144

36 -> 78 -> -73

36 -> 78 -> -73 -> -1

36 -> 78 -> -73 -> -3

36 -> 78 -> -73 -> -6

36 -> 78 -> -73 -> -17

36 -> 78 -> -73 -> -19

36 -> 78 -> -73 -> -20

36 -> 78 -> -73 -> -22

36 -> 78 -> -73 -> -26

36 -> 78 -> -73 -> -27

36 -> 78 -> -73 -> -28

36 -> 78 -> -73 -> -29

36 -> 78 -> -73 -> -30

36 -> 78 -> -73 -> -32

36 -> 78 -> -73 -> -35

36 -> 78 -> -73 -> -39

36 -> 78 -> -73 -> -43

36 -> 78 -> -73 -> -48

36 -> 78 -> -73 -> -56

36 -> 78 -> -73 -> -61

36 -> 78 -> -73 -> -62

36 -> 78 -> -73 -> -63

36 -> 78 -> -73 -> -64

36 -> 78 -> -73 -> -68

36 -> 78 -> -73 -> -71

36 -> 78 -> -73 -> -78

36 -> 78 -> -73 -> -81

36 -> 78 -> -73 -> -83

36 -> 78 -> -73 -> -86

36 -> 78 -> -73 -> -88

36 -> 78 -> -73 -> -89

36 -> 78 -> -73 -> -92

36 -> 78 -> -73 -> -95

36 -> 78 -> -73 -> -98

36 -> 78 -> -73 -> -99

36 -> 78 -> -73 -> -101

36 -> 78 -> -73 -> -106

36 -> 78 -> -73 -> -107

36 -> 78 -> -73 -> -113

36 -> 78 -> -73 -> -114

36 -> 78 -> -73 -> -117

36 -> 78 -> -73 -> -118

36 -> 78 -> -73 -> -119

36 -> 78 -> -73 -> -121

36 -> 78 -> -73 -> -122

36 -> 78 -> -73 -> -124

36 -> 78 -> -73 -> -133

36 -> 78 -> -73 -> -137

36 -> 78 -> -73 -> -144

36 -> 78 -> -73 -> -145

36 -> 78 -> -73 -> -146

36 -> 78 -> -73 -> -147

36 -> 78 -> -73 -> -150

36 -> 78 -> -73 -> -154

36 -> 78 -> -73 -> -155

36 -> 78 -> -73 -> -156

36 -> 79 -> 2 -> -32

36 -> 79 -> 4 -> -101

36 -> 79 -> 6 -> -73

36 -> 79 -> 7 -> -54

36 -> 79 -> 9 -> -6

36 -> 79 -> 12 -> -32

36 -> 79 -> 13 -> -32

36 -> 79 -> 13 -> -73

36 -> 79 -> 14 -> -54

36 -> 79 -> 15 -> -68

36 -> 79 -> 16 -> -5

36 -> 79 -> 17 -> -32

36 -> 79 -> 18 -> -73

36 -> 79 -> 18 -> -89

36 -> 79 -> 19 -> -73

36 -> 79 -> 21 -> -73

36 -> 79 -> 21 -> -101

36 -> 79 -> 21 -> -154

36 -> 79 -> 22 -> -152

36 -> 79 -> 24 -> -49

36 -> 79 -> 25 -> -132

36 -> 79 -> 26 -> -14

36 -> 79 -> 28 -> -81

36 -> 79 -> 29 -> -133

36 -> 79 -> 31 -> -1

36 -> 79 -> 32 -> -9

36 -> 79 -> 33 -> -145

36 -> 79 -> 37 -> -73

36 -> 79 -> 39 -> -101

36 -> 79 -> 40 -> -73

36 -> 79 -> 41 -> -6

36 -> 79 -> 42 -> -133

36 -> 79 -> 43 -> -73

36 -> 79 -> 43 -> -99

36 -> 79 -> 43 -> -101

36 -> 79 -> 45 -> -73

36 -> 79 -> 46 -> -32

36 -> 79 -> 48 -> -99

36 -> 79 -> 49 -> -15

36 -> 79 -> 50 -> -54

36 -> 79 -> 51 -> -2

36 -> 79 -> 52 -> -32

36 -> 79 -> 53 -> -133

36 -> 79 -> 54 -> -53

36 -> 79 -> 55 -> -12

36 -> 79 -> 56 -> -115

36 -> 79 -> 57 -> -103

36 -> 79 -> 58 -> -90

36 -> 79 -> 59 -> -148

36 -> 79 -> 60 -> -64

36 -> 79 -> 61 -> -27

36 -> 79 -> 62 -> -99

36 -> 79 -> 63 -> -32

36 -> 79 -> 64 -> -10

36 -> 79 -> 65 -> -4

36 -> 79 -> 66 -> -136

36 -> 79 -> 67 -> -135

36 -> 79 -> 68 -> -73

36 -> 79 -> 69 -> -77

36 -> 79 -> 70 -> -52

36 -> 79 -> 71 -> -32

36 -> 79 -> 73 -> -32

36 -> 79 -> 74 -> -112

36 -> 79 -> 76 -> -81

36 -> 79 -> 77 -> -73

36 -> 79 -> 78 -> -73

36 -> 79 -> 80 -> -32

36 -> 79 -> 81 -> -73

36 -> 79 -> 83 -> -125

36 -> 79 -> 84 -> -73

36 -> 79 -> 85 -> -12

36 -> 79 -> 87 -> -73

36 -> 79 -> 88 -> -83

36 -> 79 -> 90 -> -103

36 -> 79 -> 91 -> -101

36 -> 79 -> 92 -> -133

36 -> 79 -> 93 -> -145

36 -> 79 -> 94 -> -13

36 -> 79 -> 96 -> -73

36 -> 79 -> 97 -> -15

36 -> 79 -> 99 -> -73

36 -> 79 -> 101 -> -64

36 -> 79 -> 102 -> -116

36 -> 79 -> 103 -> -78

36 -> 79 -> 104 -> -2

36 -> 79 -> 106 -> -65

36 -> 79 -> 107 -> -73

36 -> 79 -> 107 -> -99

36 -> 79 -> 108 -> -80

36 -> 79 -> 110 -> -56

36 -> 79 -> 112 -> -54

36 -> 79 -> 113 -> -73

36 -> 79 -> 114 -> -73

36 -> 79 -> 116 -> -73

36 -> 79 -> 117 -> -73

36 -> 79 -> 117 -> -101

36 -> 79 -> 118 -> -73

36 -> 79 -> 120 -> -133

36 -> 79 -> 122 -> -56

36 -> 79 -> 123 -> -73

36 -> 79 -> 125 -> -104

36 -> 79 -> 126 -> -59

36 -> 79 -> 127 -> -93

36 -> 79 -> 129 -> -32

36 -> 79 -> 130 -> -73

36 -> 79 -> 132 -> -32

36 -> 79 -> 133 -> -101

36 -> 79 -> 134 -> -99

36 -> 79 -> 135 -> -21

36 -> 79 -> 135 -> -73

36 -> 79 -> 135 -> -139

36 -> 79 -> 136 -> -20

36 -> 79 -> 139 -> -15

36 -> 79 -> 142 -> -154

36 -> 79 -> 143 -> -50

36 -> 79 -> 145 -> -125

36 -> 79 -> 146 -> -20

36 -> 79 -> 147 -> -32

36 -> 79 -> 148 -> -32

36 -> 79 -> 149 -> -73

36 -> 79 -> 150 -> -91

36 -> 79 -> 151 -> -101

36 -> 79 -> 152 -> -36

36 -> 79 -> 153 -> -2

36 -> 79 -> 155 -> -73

36 -> 79 -> 158 -> -72

36 -> 79 -> 160 -> -129

36 -> 79 -> 161 -> -78

36 -> 79 -> 162 -> -133

36 -> 79 -> 163 -> -8

36 -> 79 -> 164 -> -134

36 -> 79 -> 165 -> -54

36 -> 79 -> 168 -> -73

36 -> 79 -> 168 -> -102

36 -> 79 -> 169 -> -99

36 -> 79 -> 170 -> -138

36 -> 79 -> 171 -> -13

36 -> 79 -> 172 -> -58

36 -> 79 -> 173 -> -154

36 -> 79 -> 174 -> -114

36 -> 79 -> 176 -> -33

36 -> 79 -> 177 -> -106

36 -> 79 -> 178 -> -69

36 -> 79 -> 180 -> -20

36 -> 79 -> 181 -> -84

36 -> 79 -> 182 -> -73

36 -> 79 -> 186 -> -99

36 -> 79 -> 187 -> -53

36 -> 79 -> 188 -> -24

36 -> 79 -> 189 -> -73

36 -> 79 -> -73

36 -> 79 -> -73 -> -1

36 -> 79 -> -73 -> -3

36 -> 79 -> -73 -> -6

36 -> 79 -> -73 -> -17

36 -> 79 -> -73 -> -19

36 -> 79 -> -73 -> -20

36 -> 79 -> -73 -> -22

36 -> 79 -> -73 -> -26

36 -> 79 -> -73 -> -27

36 -> 79 -> -73 -> -28

36 -> 79 -> -73 -> -29

36 -> 79 -> -73 -> -30

36 -> 79 -> -73 -> -32

36 -> 79 -> -73 -> -35

36 -> 79 -> -73 -> -39

36 -> 79 -> -73 -> -43

36 -> 79 -> -73 -> -48

36 -> 79 -> -73 -> -56

36 -> 79 -> -73 -> -61

36 -> 79 -> -73 -> -62

36 -> 79 -> -73 -> -63

36 -> 79 -> -73 -> -64

36 -> 79 -> -73 -> -68

36 -> 79 -> -73 -> -71

36 -> 79 -> -73 -> -78

36 -> 79 -> -73 -> -81

36 -> 79 -> -73 -> -83

36 -> 79 -> -73 -> -86

36 -> 79 -> -73 -> -88

36 -> 79 -> -73 -> -89

36 -> 79 -> -73 -> -92

36 -> 79 -> -73 -> -95

36 -> 79 -> -73 -> -98

36 -> 79 -> -73 -> -99

36 -> 79 -> -73 -> -101

36 -> 79 -> -73 -> -106

36 -> 79 -> -73 -> -107

36 -> 79 -> -73 -> -113

36 -> 79 -> -73 -> -114

36 -> 79 -> -73 -> -117

36 -> 79 -> -73 -> -118

36 -> 79 -> -73 -> -119

36 -> 79 -> -73 -> -121

36 -> 79 -> -73 -> -122

36 -> 79 -> -73 -> -124

36 -> 79 -> -73 -> -133

36 -> 79 -> -73 -> -137

36 -> 79 -> -73 -> -144

36 -> 79 -> -73 -> -145

36 -> 79 -> -73 -> -146

36 -> 79 -> -73 -> -147

36 -> 79 -> -73 -> -150

36 -> 79 -> -73 -> -154

36 -> 79 -> -73 -> -155

36 -> 79 -> -73 -> -156

36 -> 84 -> 2 -> -32

36 -> 84 -> 4 -> -101

36 -> 84 -> 6 -> -73

36 -> 84 -> 7 -> -54

36 -> 84 -> 9 -> -6

36 -> 84 -> 12 -> -32

36 -> 84 -> 13 -> -32

36 -> 84 -> 13 -> -73

36 -> 84 -> 14 -> -54

36 -> 84 -> 15 -> -68

36 -> 84 -> 16 -> -5

36 -> 84 -> 17 -> -32

36 -> 84 -> 18 -> -73

36 -> 84 -> 18 -> -89

36 -> 84 -> 19 -> -73

36 -> 84 -> 21 -> -73

36 -> 84 -> 21 -> -101

36 -> 84 -> 21 -> -154

36 -> 84 -> 22 -> -152

36 -> 84 -> 24 -> -49

36 -> 84 -> 25 -> -132

36 -> 84 -> 26 -> -14

36 -> 84 -> 28 -> -81

36 -> 84 -> 29 -> -133

36 -> 84 -> 31 -> -1

36 -> 84 -> 32 -> -9

36 -> 84 -> 33 -> -145

36 -> 84 -> 37 -> -73

36 -> 84 -> 39 -> -101

36 -> 84 -> 40 -> -73

36 -> 84 -> 41 -> -6

36 -> 84 -> 42 -> -133

36 -> 84 -> 43 -> -73

36 -> 84 -> 43 -> -99

36 -> 84 -> 43 -> -101

36 -> 84 -> 45 -> -73

36 -> 84 -> 46 -> -32

36 -> 84 -> 48 -> -99

36 -> 84 -> 49 -> -15

36 -> 84 -> 50 -> -54

36 -> 84 -> 51 -> -2

36 -> 84 -> 52 -> -32

36 -> 84 -> 53 -> -133

36 -> 84 -> 54 -> -53

36 -> 84 -> 55 -> -12

36 -> 84 -> 56 -> -115

36 -> 84 -> 57 -> -103

36 -> 84 -> 58 -> -90

36 -> 84 -> 59 -> -148

36 -> 84 -> 60 -> -64

36 -> 84 -> 61 -> -27

36 -> 84 -> 62 -> -99

36 -> 84 -> 63 -> -32

36 -> 84 -> 64 -> -10

36 -> 84 -> 65 -> -4

36 -> 84 -> 66 -> -136

36 -> 84 -> 67 -> -135

36 -> 84 -> 68 -> -73

36 -> 84 -> 69 -> -77

36 -> 84 -> 70 -> -52

36 -> 84 -> 71 -> -32

36 -> 84 -> 73 -> -32

36 -> 84 -> 74 -> -112

36 -> 84 -> 76 -> -81

36 -> 84 -> 77 -> -73

36 -> 84 -> 78 -> -73

36 -> 84 -> 79 -> -73

36 -> 84 -> 80 -> -32

36 -> 84 -> 81 -> -73

36 -> 84 -> 83 -> -125

36 -> 84 -> 85 -> -12

36 -> 84 -> 87 -> -73

36 -> 84 -> 88 -> -83

36 -> 84 -> 90 -> -103

36 -> 84 -> 91 -> -101

36 -> 84 -> 92 -> -133

36 -> 84 -> 93 -> -145

36 -> 84 -> 94 -> -13

36 -> 84 -> 96 -> -73

36 -> 84 -> 97 -> -15

36 -> 84 -> 99 -> -73

36 -> 84 -> 101 -> -64

36 -> 84 -> 102 -> -116

36 -> 84 -> 103 -> -78

36 -> 84 -> 104 -> -2

36 -> 84 -> 106 -> -65

36 -> 84 -> 107 -> -73

36 -> 84 -> 107 -> -99

36 -> 84 -> 108 -> -80

36 -> 84 -> 110 -> -56

36 -> 84 -> 112 -> -54

36 -> 84 -> 113 -> -73

36 -> 84 -> 114 -> -73

36 -> 84 -> 116 -> -73

36 -> 84 -> 117 -> -73

36 -> 84 -> 117 -> -101

36 -> 84 -> 118 -> -73

36 -> 84 -> 120 -> -133

36 -> 84 -> 122 -> -56

36 -> 84 -> 123 -> -73

36 -> 84 -> 125 -> -104

36 -> 84 -> 126 -> -59

36 -> 84 -> 127 -> -93

36 -> 84 -> 129 -> -32

36 -> 84 -> 130 -> -73

36 -> 84 -> 132 -> -32

36 -> 84 -> 133 -> -101

36 -> 84 -> 134 -> -99

36 -> 84 -> 135 -> -21

36 -> 84 -> 135 -> -73

36 -> 84 -> 135 -> -139

36 -> 84 -> 136 -> -20

36 -> 84 -> 139 -> -15

36 -> 84 -> 142 -> -154

36 -> 84 -> 143 -> -50

36 -> 84 -> 145 -> -125

36 -> 84 -> 146 -> -20

36 -> 84 -> 147 -> -32

36 -> 84 -> 148 -> -32

36 -> 84 -> 149 -> -73

36 -> 84 -> 150 -> -91

36 -> 84 -> 151 -> -101

36 -> 84 -> 152 -> -36

36 -> 84 -> 153 -> -2

36 -> 84 -> 155 -> -73

36 -> 84 -> 158 -> -72

36 -> 84 -> 160 -> -129

36 -> 84 -> 161 -> -78

36 -> 84 -> 162 -> -133

36 -> 84 -> 163 -> -8

36 -> 84 -> 164 -> -134

36 -> 84 -> 165 -> -54

36 -> 84 -> 168 -> -73

36 -> 84 -> 168 -> -102

36 -> 84 -> 169 -> -99

36 -> 84 -> 170 -> -138

36 -> 84 -> 171 -> -13

36 -> 84 -> 172 -> -58

36 -> 84 -> 173 -> -154

36 -> 84 -> 174 -> -114

36 -> 84 -> 176 -> -33

36 -> 84 -> 177 -> -106

36 -> 84 -> 178 -> -69

36 -> 84 -> 180 -> -20

36 -> 84 -> 181 -> -84

36 -> 84 -> 182 -> -73

36 -> 84 -> 186 -> -99

36 -> 84 -> 187 -> -53

36 -> 84 -> 188 -> -24

36 -> 84 -> 189 -> -73

36 -> 84 -> -73

36 -> 84 -> -73 -> -1

36 -> 84 -> -73 -> -3

36 -> 84 -> -73 -> -6

36 -> 84 -> -73 -> -17

36 -> 84 -> -73 -> -19

36 -> 84 -> -73 -> -20

36 -> 84 -> -73 -> -22

36 -> 84 -> -73 -> -26

36 -> 84 -> -73 -> -27

36 -> 84 -> -73 -> -28

36 -> 84 -> -73 -> -29

36 -> 84 -> -73 -> -30

36 -> 84 -> -73 -> -32

36 -> 84 -> -73 -> -35

36 -> 84 -> -73 -> -39

36 -> 84 -> -73 -> -43

36 -> 84 -> -73 -> -48

36 -> 84 -> -73 -> -56

36 -> 84 -> -73 -> -61

36 -> 84 -> -73 -> -62

36 -> 84 -> -73 -> -63

36 -> 84 -> -73 -> -64

36 -> 84 -> -73 -> -68

36 -> 84 -> -73 -> -71

36 -> 84 -> -73 -> -78

36 -> 84 -> -73 -> -81

36 -> 84 -> -73 -> -83

36 -> 84 -> -73 -> -86

36 -> 84 -> -73 -> -88

36 -> 84 -> -73 -> -89

36 -> 84 -> -73 -> -92

36 -> 84 -> -73 -> -95

36 -> 84 -> -73 -> -98

36 -> 84 -> -73 -> -99

36 -> 84 -> -73 -> -101

36 -> 84 -> -73 -> -106

36 -> 84 -> -73 -> -107

36 -> 84 -> -73 -> -113

36 -> 84 -> -73 -> -114

36 -> 84 -> -73 -> -117

36 -> 84 -> -73 -> -118

36 -> 84 -> -73 -> -119

36 -> 84 -> -73 -> -121

36 -> 84 -> -73 -> -122

36 -> 84 -> -73 -> -124

36 -> 84 -> -73 -> -133

36 -> 84 -> -73 -> -137

36 -> 84 -> -73 -> -144

36 -> 84 -> -73 -> -145

36 -> 84 -> -73 -> -146

36 -> 84 -> -73 -> -147

36 -> 84 -> -73 -> -150

36 -> 84 -> -73 -> -154

36 -> 84 -> -73 -> -155

36 -> 84 -> -73 -> -156

36 -> 87 -> 2 -> -32

36 -> 87 -> 4 -> -101

36 -> 87 -> 6 -> -73

36 -> 87 -> 7 -> -54

36 -> 87 -> 9 -> -6

36 -> 87 -> 12 -> -32

36 -> 87 -> 13 -> -32

36 -> 87 -> 13 -> -73

36 -> 87 -> 14 -> -54

36 -> 87 -> 15 -> -68

36 -> 87 -> 16 -> -5

36 -> 87 -> 17 -> -32

36 -> 87 -> 18 -> -73

36 -> 87 -> 18 -> -89

36 -> 87 -> 19 -> -73

36 -> 87 -> 21 -> -73

36 -> 87 -> 21 -> -101

36 -> 87 -> 21 -> -154

36 -> 87 -> 22 -> -152

36 -> 87 -> 24 -> -49

36 -> 87 -> 25 -> -132

36 -> 87 -> 26 -> -14

36 -> 87 -> 28 -> -81

36 -> 87 -> 29 -> -133

36 -> 87 -> 31 -> -1

36 -> 87 -> 32 -> -9

36 -> 87 -> 33 -> -145

36 -> 87 -> 37 -> -73

36 -> 87 -> 39 -> -101

36 -> 87 -> 40 -> -73

36 -> 87 -> 41 -> -6

36 -> 87 -> 42 -> -133

36 -> 87 -> 43 -> -73

36 -> 87 -> 43 -> -99

36 -> 87 -> 43 -> -101

36 -> 87 -> 45 -> -73

36 -> 87 -> 46 -> -32

36 -> 87 -> 48 -> -99

36 -> 87 -> 49 -> -15

36 -> 87 -> 50 -> -54

36 -> 87 -> 51 -> -2

36 -> 87 -> 52 -> -32

36 -> 87 -> 53 -> -133

36 -> 87 -> 54 -> -53

36 -> 87 -> 55 -> -12

36 -> 87 -> 56 -> -115

36 -> 87 -> 57 -> -103

36 -> 87 -> 58 -> -90

36 -> 87 -> 59 -> -148

36 -> 87 -> 60 -> -64

36 -> 87 -> 61 -> -27

36 -> 87 -> 62 -> -99

36 -> 87 -> 63 -> -32

36 -> 87 -> 64 -> -10

36 -> 87 -> 65 -> -4

36 -> 87 -> 66 -> -136

36 -> 87 -> 67 -> -135

36 -> 87 -> 68 -> -73

36 -> 87 -> 69 -> -77

36 -> 87 -> 70 -> -52

36 -> 87 -> 71 -> -32

36 -> 87 -> 73 -> -32

36 -> 87 -> 74 -> -112

36 -> 87 -> 76 -> -81

36 -> 87 -> 77 -> -73

36 -> 87 -> 78 -> -73

36 -> 87 -> 79 -> -73

36 -> 87 -> 80 -> -32

36 -> 87 -> 81 -> -73

36 -> 87 -> 83 -> -125

36 -> 87 -> 84 -> -73

36 -> 87 -> 85 -> -12

36 -> 87 -> 88 -> -83

36 -> 87 -> 90 -> -103

36 -> 87 -> 91 -> -101

36 -> 87 -> 92 -> -133

36 -> 87 -> 93 -> -145

36 -> 87 -> 94 -> -13

36 -> 87 -> 96 -> -73

36 -> 87 -> 97 -> -15

36 -> 87 -> 99 -> -73

36 -> 87 -> 101 -> -64

36 -> 87 -> 102 -> -116

36 -> 87 -> 103 -> -78

36 -> 87 -> 104 -> -2

36 -> 87 -> 106 -> -65

36 -> 87 -> 107 -> -73

36 -> 87 -> 107 -> -99

36 -> 87 -> 108 -> -80

36 -> 87 -> 110 -> -56

36 -> 87 -> 112 -> -54

36 -> 87 -> 113 -> -73

36 -> 87 -> 114 -> -73

36 -> 87 -> 116 -> -73

36 -> 87 -> 117 -> -73

36 -> 87 -> 117 -> -101

36 -> 87 -> 118 -> -73

36 -> 87 -> 120 -> -133

36 -> 87 -> 122 -> -56

36 -> 87 -> 123 -> -73

36 -> 87 -> 125 -> -104

36 -> 87 -> 126 -> -59

36 -> 87 -> 127 -> -93

36 -> 87 -> 129 -> -32

36 -> 87 -> 130 -> -73

36 -> 87 -> 132 -> -32

36 -> 87 -> 133 -> -101

36 -> 87 -> 134 -> -99

36 -> 87 -> 135 -> -21

36 -> 87 -> 135 -> -73

36 -> 87 -> 135 -> -139

36 -> 87 -> 136 -> -20

36 -> 87 -> 139 -> -15

36 -> 87 -> 142 -> -154

36 -> 87 -> 143 -> -50

36 -> 87 -> 145 -> -125

36 -> 87 -> 146 -> -20

36 -> 87 -> 147 -> -32

36 -> 87 -> 148 -> -32

36 -> 87 -> 149 -> -73

36 -> 87 -> 150 -> -91

36 -> 87 -> 151 -> -101

36 -> 87 -> 152 -> -36

36 -> 87 -> 153 -> -2

36 -> 87 -> 155 -> -73

36 -> 87 -> 158 -> -72

36 -> 87 -> 160 -> -129

36 -> 87 -> 161 -> -78

36 -> 87 -> 162 -> -133

36 -> 87 -> 163 -> -8

36 -> 87 -> 164 -> -134

36 -> 87 -> 165 -> -54

36 -> 87 -> 168 -> -73

36 -> 87 -> 168 -> -102

36 -> 87 -> 169 -> -99

36 -> 87 -> 170 -> -138

36 -> 87 -> 171 -> -13

36 -> 87 -> 172 -> -58

36 -> 87 -> 173 -> -154

36 -> 87 -> 174 -> -114

36 -> 87 -> 176 -> -33

36 -> 87 -> 177 -> -106

36 -> 87 -> 178 -> -69

36 -> 87 -> 180 -> -20

36 -> 87 -> 181 -> -84

36 -> 87 -> 182 -> -73

36 -> 87 -> 186 -> -99

36 -> 87 -> 187 -> -53

36 -> 87 -> 188 -> -24

36 -> 87 -> 189 -> -73

36 -> 87 -> -73

36 -> 87 -> -73 -> -1

36 -> 87 -> -73 -> -3

36 -> 87 -> -73 -> -6

36 -> 87 -> -73 -> -17

36 -> 87 -> -73 -> -19

36 -> 87 -> -73 -> -20

36 -> 87 -> -73 -> -22

36 -> 87 -> -73 -> -26

36 -> 87 -> -73 -> -27

36 -> 87 -> -73 -> -28

36 -> 87 -> -73 -> -29

36 -> 87 -> -73 -> -30

36 -> 87 -> -73 -> -32

36 -> 87 -> -73 -> -35

36 -> 87 -> -73 -> -39

36 -> 87 -> -73 -> -43

36 -> 87 -> -73 -> -48

36 -> 87 -> -73 -> -56

36 -> 87 -> -73 -> -61

36 -> 87 -> -73 -> -62

36 -> 87 -> -73 -> -63

36 -> 87 -> -73 -> -64

36 -> 87 -> -73 -> -68

36 -> 87 -> -73 -> -71

36 -> 87 -> -73 -> -78

36 -> 87 -> -73 -> -81

36 -> 87 -> -73 -> -83

36 -> 87 -> -73 -> -86

36 -> 87 -> -73 -> -88

36 -> 87 -> -73 -> -89

36 -> 87 -> -73 -> -92

36 -> 87 -> -73 -> -95

36 -> 87 -> -73 -> -98

36 -> 87 -> -73 -> -99

36 -> 87 -> -73 -> -101

36 -> 87 -> -73 -> -106

36 -> 87 -> -73 -> -107

36 -> 87 -> -73 -> -113

36 -> 87 -> -73 -> -114

36 -> 87 -> -73 -> -117

36 -> 87 -> -73 -> -118

36 -> 87 -> -73 -> -119

36 -> 87 -> -73 -> -121

36 -> 87 -> -73 -> -122

36 -> 87 -> -73 -> -124

36 -> 87 -> -73 -> -133

36 -> 87 -> -73 -> -137

36 -> 87 -> -73 -> -144

36 -> 87 -> -73 -> -145

36 -> 87 -> -73 -> -146

36 -> 87 -> -73 -> -147

36 -> 87 -> -73 -> -150

36 -> 87 -> -73 -> -154

36 -> 87 -> -73 -> -155

36 -> 87 -> -73 -> -156

36 -> 92 -> 3 -> -32

36 -> 92 -> 3 -> -42

36 -> 92 -> 4 -> -101

36 -> 92 -> 7 -> -54

36 -> 92 -> 9 -> -6

36 -> 92 -> 14 -> -54

36 -> 92 -> 15 -> -68

36 -> 92 -> 17 -> -32

36 -> 92 -> 19 -> -73

36 -> 92 -> 21 -> -73

36 -> 92 -> 21 -> -101

36 -> 92 -> 21 -> -154

36 -> 92 -> 22 -> -152

36 -> 92 -> 24 -> -49

36 -> 92 -> 25 -> -132

36 -> 92 -> 27 -> -19

36 -> 92 -> 27 -> -20

36 -> 92 -> 27 -> -32

36 -> 92 -> 27 -> -62

36 -> 92 -> 27 -> -68

36 -> 92 -> 27 -> -73

36 -> 92 -> 27 -> -81

36 -> 92 -> 27 -> -98

36 -> 92 -> 27 -> -99

36 -> 92 -> 27 -> -101

36 -> 92 -> 27 -> -107

36 -> 92 -> 27 -> -124

36 -> 92 -> 27 -> -133

36 -> 92 -> 27 -> -145

36 -> 92 -> 28 -> -81

36 -> 92 -> 29 -> -133

36 -> 92 -> 30 -> -1

36 -> 92 -> 30 -> -3

36 -> 92 -> 30 -> -32

36 -> 92 -> 30 -> -39

36 -> 92 -> 30 -> -63

36 -> 92 -> 30 -> -73

36 -> 92 -> 30 -> -81

36 -> 92 -> 30 -> -99

36 -> 92 -> 30 -> -101

36 -> 92 -> 30 -> -106

36 -> 92 -> 30 -> -117

36 -> 92 -> 30 -> -133

36 -> 92 -> 30 -> -137

36 -> 92 -> 30 -> -150

36 -> 92 -> 30 -> -156

36 -> 92 -> 32 -> -9

36 -> 92 -> 33 -> -145

36 -> 92 -> 38 -> -55

36 -> 92 -> 38 -> -56

36 -> 92 -> 38 -> -105

36 -> 92 -> 39 -> -101

36 -> 92 -> 40 -> -73

36 -> 92 -> 41 -> -6

36 -> 92 -> 42 -> -133

36 -> 92 -> 44 -> -29

36 -> 92 -> 44 -> -48

36 -> 92 -> 44 -> -81

36 -> 92 -> 44 -> -89

36 -> 92 -> 44 -> -99

36 -> 92 -> 44 -> -114

36 -> 92 -> 45 -> -73

36 -> 92 -> 48 -> -99

36 -> 92 -> 49 -> -15

36 -> 92 -> 55 -> -12

36 -> 92 -> 60 -> -64

36 -> 92 -> 61 -> -27

36 -> 92 -> 64 -> -10

36 -> 92 -> 65 -> -4

36 -> 92 -> 66 -> -136

36 -> 92 -> 67 -> -135

36 -> 92 -> 68 -> -73

36 -> 92 -> 69 -> -77

36 -> 92 -> 73 -> -32

36 -> 92 -> 74 -> -112

36 -> 92 -> 75 -> -1

36 -> 92 -> 75 -> -29

36 -> 92 -> 76 -> -81

36 -> 92 -> 77 -> -73

36 -> 92 -> 79 -> -73

36 -> 92 -> 84 -> -73

36 -> 92 -> 87 -> -73

36 -> 92 -> 88 -> -83

36 -> 92 -> 89 -> -6

36 -> 92 -> 89 -> -73

36 -> 92 -> 89 -> -81

36 -> 92 -> 89 -> -83

36 -> 92 -> 89 -> -86

36 -> 92 -> 89 -> -99

36 -> 92 -> 89 -> -101

36 -> 92 -> 89 -> -114

36 -> 92 -> 89 -> -155

36 -> 92 -> 91 -> -101

36 -> 92 -> 97 -> -15

36 -> 92 -> 103 -> -78

36 -> 92 -> 105 -> -32

36 -> 92 -> 105 -> -146

36 -> 92 -> 107 -> -73

36 -> 92 -> 107 -> -99

36 -> 92 -> 108 -> -80

36 -> 92 -> 109 -> -20

36 -> 92 -> 109 -> -73

36 -> 92 -> 109 -> -99

36 -> 92 -> 109 -> -101

36 -> 92 -> 110 -> -56

36 -> 92 -> 113 -> -73

36 -> 92 -> 116 -> -73

36 -> 92 -> 117 -> -73

36 -> 92 -> 117 -> -101

36 -> 92 -> 118 -> -73

36 -> 92 -> 120 -> -133

36 -> 92 -> 122 -> -56

36 -> 92 -> 123 -> -73

36 -> 92 -> 128 -> -32

36 -> 92 -> 128 -> -95

36 -> 92 -> 129 -> -32

36 -> 92 -> 130 -> -73

36 -> 92 -> 133 -> -101

36 -> 92 -> 134 -> -99

36 -> 92 -> 136 -> -20

36 -> 92 -> 138 -> -20

36 -> 92 -> 138 -> -61

36 -> 92 -> 138 -> -73

36 -> 92 -> 138 -> -98

36 -> 92 -> 138 -> -133

36 -> 92 -> 139 -> -15

36 -> 92 -> 140 -> -99

36 -> 92 -> 140 -> -133

36 -> 92 -> 141 -> -51

36 -> 92 -> 141 -> -100

36 -> 92 -> 141 -> -127

36 -> 92 -> 142 -> -154

36 -> 92 -> 146 -> -20

36 -> 92 -> 148 -> -32

36 -> 92 -> 149 -> -73

36 -> 92 -> 150 -> -91

36 -> 92 -> 151 -> -101

36 -> 92 -> 153 -> -2

36 -> 92 -> 157 -> -26

36 -> 92 -> 157 -> -28

36 -> 92 -> 157 -> -32

36 -> 92 -> 157 -> -68

36 -> 92 -> 157 -> -86

36 -> 92 -> 157 -> -99

36 -> 92 -> 157 -> -101

36 -> 92 -> 157 -> -118

36 -> 92 -> 157 -> -119

36 -> 92 -> 157 -> -120

36 -> 92 -> 157 -> -121

36 -> 92 -> 157 -> -122

36 -> 92 -> 157 -> -133

36 -> 92 -> 160 -> -129

36 -> 92 -> 161 -> -78

36 -> 92 -> 162 -> -133

36 -> 92 -> 163 -> -8

36 -> 92 -> 170 -> -138

36 -> 92 -> 171 -> -13

36 -> 92 -> 172 -> -58

36 -> 92 -> 173 -> -154

36 -> 92 -> 176 -> -33

36 -> 92 -> 177 -> -106

36 -> 92 -> 179 -> -22

36 -> 92 -> 179 -> -147

36 -> 92 -> 180 -> -20

36 -> 92 -> 181 -> -84

36 -> 92 -> 182 -> -73

36 -> 92 -> 186 -> -99

36 -> 92 -> 188 -> -24

36 -> 92 -> 189 -> -73

36 -> 92 -> -133

36 -> 92 -> -133 -> -1

36 -> 92 -> -133 -> -3

36 -> 92 -> -133 -> -19

36 -> 92 -> -133 -> -20

36 -> 92 -> -133 -> -22

36 -> 92 -> -133 -> -26

36 -> 92 -> -133 -> -27

36 -> 92 -> -133 -> -28

36 -> 92 -> -133 -> -29

36 -> 92 -> -133 -> -32

36 -> 92 -> -133 -> -36

36 -> 92 -> -133 -> -39

36 -> 92 -> -133 -> -40

36 -> 92 -> -133 -> -48

36 -> 92 -> -133 -> -56

36 -> 92 -> -133 -> -61

36 -> 92 -> -133 -> -62

36 -> 92 -> -133 -> -63

36 -> 92 -> -133 -> -64

36 -> 92 -> -133 -> -68

36 -> 92 -> -133 -> -73

36 -> 92 -> -133 -> -78

36 -> 92 -> -133 -> -79

36 -> 92 -> -133 -> -81

36 -> 92 -> -133 -> -86

36 -> 92 -> -133 -> -94

36 -> 92 -> -133 -> -95

36 -> 92 -> -133 -> -98

36 -> 92 -> -133 -> -99

36 -> 92 -> -133 -> -101

36 -> 92 -> -133 -> -106

36 -> 92 -> -133 -> -107

36 -> 92 -> -133 -> -114

36 -> 92 -> -133 -> -117

36 -> 92 -> -133 -> -118

36 -> 92 -> -133 -> -119

36 -> 92 -> -133 -> -121

36 -> 92 -> -133 -> -122

36 -> 92 -> -133 -> -123

36 -> 92 -> -133 -> -124

36 -> 92 -> -133 -> -125

36 -> 92 -> -133 -> -137

36 -> 92 -> -133 -> -145

36 -> 92 -> -133 -> -146

36 -> 92 -> -133 -> -147

36 -> 92 -> -133 -> -150

36 -> 92 -> -133 -> -154

36 -> 92 -> -133 -> -156

36 -> 99 -> 7 -> -54

36 -> 99 -> 9 -> -6

36 -> 99 -> 12 -> -32

36 -> 99 -> 14 -> -54

36 -> 99 -> 15 -> -68

36 -> 99 -> 16 -> -5

36 -> 99 -> 19 -> -73

36 -> 99 -> 22 -> -152

36 -> 99 -> 23 -> -23

36 -> 99 -> 23 -> -126

36 -> 99 -> 24 -> -49

36 -> 99 -> 25 -> -132

36 -> 99 -> 26 -> -14

36 -> 99 -> 27 -> -19

36 -> 99 -> 27 -> -20

36 -> 99 -> 27 -> -32

36 -> 99 -> 27 -> -62

36 -> 99 -> 27 -> -68

36 -> 99 -> 27 -> -73

36 -> 99 -> 27 -> -81

36 -> 99 -> 27 -> -98

36 -> 99 -> 27 -> -99

36 -> 99 -> 27 -> -101

36 -> 99 -> 27 -> -107

36 -> 99 -> 27 -> -124

36 -> 99 -> 27 -> -133

36 -> 99 -> 27 -> -145

36 -> 99 -> 28 -> -81

36 -> 99 -> 31 -> -1

36 -> 99 -> 32 -> -9

36 -> 99 -> 33 -> -145

36 -> 99 -> 34 -> -32

36 -> 99 -> 34 -> -71

36 -> 99 -> 34 -> -113

36 -> 99 -> 41 -> -6

36 -> 99 -> 42 -> -133

36 -> 99 -> 45 -> -73

36 -> 99 -> 47 -> -32

36 -> 99 -> 47 -> -37

36 -> 99 -> 48 -> -99

36 -> 99 -> 49 -> -15

36 -> 99 -> 52 -> -32

36 -> 99 -> 53 -> -133

36 -> 99 -> 55 -> -12

36 -> 99 -> 59 -> -148

36 -> 99 -> 61 -> -27

36 -> 99 -> 63 -> -32

36 -> 99 -> 64 -> -10

36 -> 99 -> 65 -> -4

36 -> 99 -> 66 -> -136

36 -> 99 -> 67 -> -135

36 -> 99 -> 68 -> -73

36 -> 99 -> 69 -> -77

36 -> 99 -> 71 -> -32

36 -> 99 -> 73 -> -32

36 -> 99 -> 74 -> -112

36 -> 99 -> 76 -> -81

36 -> 99 -> 79 -> -73

36 -> 99 -> 84 -> -73

36 -> 99 -> 87 -> -73

36 -> 99 -> 88 -> -83

36 -> 99 -> 91 -> -101

36 -> 99 -> 93 -> -145

36 -> 99 -> 97 -> -15

36 -> 99 -> 107 -> -73

36 -> 99 -> 107 -> -99

36 -> 99 -> 108 -> -80

36 -> 99 -> 113 -> -73

36 -> 99 -> 116 -> -73

36 -> 99 -> 117 -> -73

36 -> 99 -> 117 -> -101

36 -> 99 -> 118 -> -73

36 -> 99 -> 120 -> -133

36 -> 99 -> 122 -> -56

36 -> 99 -> 123 -> -73

36 -> 99 -> 124 -> -32

36 -> 99 -> 124 -> -103

36 -> 99 -> 127 -> -93

36 -> 99 -> 129 -> -32

36 -> 99 -> 130 -> -73

36 -> 99 -> 136 -> -20

36 -> 99 -> 138 -> -20

36 -> 99 -> 138 -> -61

36 -> 99 -> 138 -> -73

36 -> 99 -> 138 -> -98

36 -> 99 -> 138 -> -133

36 -> 99 -> 139 -> -15

36 -> 99 -> 142 -> -154

36 -> 99 -> 148 -> -32

36 -> 99 -> 149 -> -73

36 -> 99 -> 150 -> -91

36 -> 99 -> 153 -> -2

36 -> 99 -> 155 -> -73

36 -> 99 -> 157 -> -26

36 -> 99 -> 157 -> -28

36 -> 99 -> 157 -> -32

36 -> 99 -> 157 -> -68

36 -> 99 -> 157 -> -86

36 -> 99 -> 157 -> -99

36 -> 99 -> 157 -> -101

36 -> 99 -> 157 -> -118

36 -> 99 -> 157 -> -119

36 -> 99 -> 157 -> -120

36 -> 99 -> 157 -> -121

36 -> 99 -> 157 -> -122

36 -> 99 -> 157 -> -133

36 -> 99 -> 158 -> -72

36 -> 99 -> 159 -> -80

36 -> 99 -> 159 -> -128

36 -> 99 -> 159 -> -129

36 -> 99 -> 160 -> -129

36 -> 99 -> 162 -> -133

36 -> 99 -> 163 -> -8

36 -> 99 -> 168 -> -73

36 -> 99 -> 168 -> -102

36 -> 99 -> 170 -> -138

36 -> 99 -> 171 -> -13

36 -> 99 -> 172 -> -58

36 -> 99 -> 175 -> -32

36 -> 99 -> 175 -> -34

36 -> 99 -> 176 -> -33

36 -> 99 -> 177 -> -106

36 -> 99 -> 178 -> -69

36 -> 99 -> 179 -> -22

36 -> 99 -> 179 -> -147

36 -> 99 -> 181 -> -84

36 -> 99 -> 182 -> -73

36 -> 99 -> 184 -> -86

36 -> 99 -> 184 -> -101

36 -> 99 -> 184 -> -133

36 -> 99 -> 186 -> -99

36 -> 99 -> 188 -> -24

36 -> 99 -> 189 -> -73

36 -> 99 -> -73

36 -> 99 -> -73 -> -1

36 -> 99 -> -73 -> -3

36 -> 99 -> -73 -> -6

36 -> 99 -> -73 -> -17

36 -> 99 -> -73 -> -19

36 -> 99 -> -73 -> -20

36 -> 99 -> -73 -> -22

36 -> 99 -> -73 -> -26

36 -> 99 -> -73 -> -27

36 -> 99 -> -73 -> -28

36 -> 99 -> -73 -> -29

36 -> 99 -> -73 -> -30

36 -> 99 -> -73 -> -32

36 -> 99 -> -73 -> -35

36 -> 99 -> -73 -> -39

36 -> 99 -> -73 -> -43

36 -> 99 -> -73 -> -48

36 -> 99 -> -73 -> -56

36 -> 99 -> -73 -> -61

36 -> 99 -> -73 -> -62

36 -> 99 -> -73 -> -63

36 -> 99 -> -73 -> -64

36 -> 99 -> -73 -> -68

36 -> 99 -> -73 -> -71

36 -> 99 -> -73 -> -78

36 -> 99 -> -73 -> -81

36 -> 99 -> -73 -> -83

36 -> 99 -> -73 -> -86

36 -> 99 -> -73 -> -88

36 -> 99 -> -73 -> -89

36 -> 99 -> -73 -> -92

36 -> 99 -> -73 -> -95

36 -> 99 -> -73 -> -98

36 -> 99 -> -73 -> -99

36 -> 99 -> -73 -> -101

36 -> 99 -> -73 -> -106

36 -> 99 -> -73 -> -107

36 -> 99 -> -73 -> -113

36 -> 99 -> -73 -> -114

36 -> 99 -> -73 -> -117

36 -> 99 -> -73 -> -118

36 -> 99 -> -73 -> -119

36 -> 99 -> -73 -> -121

36 -> 99 -> -73 -> -122

36 -> 99 -> -73 -> -124

36 -> 99 -> -73 -> -133

36 -> 99 -> -73 -> -137

36 -> 99 -> -73 -> -144

36 -> 99 -> -73 -> -145

36 -> 99 -> -73 -> -146

36 -> 99 -> -73 -> -147

36 -> 99 -> -73 -> -150

36 -> 99 -> -73 -> -154

36 -> 99 -> -73 -> -155

36 -> 99 -> -73 -> -156

36 -> 103 -> 3 -> -32

36 -> 103 -> 3 -> -42

36 -> 103 -> 4 -> -101

36 -> 103 -> 7 -> -54

36 -> 103 -> 9 -> -6

36 -> 103 -> 14 -> -54

36 -> 103 -> 15 -> -68

36 -> 103 -> 19 -> -73

36 -> 103 -> 21 -> -73

36 -> 103 -> 21 -> -101

36 -> 103 -> 21 -> -154

36 -> 103 -> 22 -> -152

36 -> 103 -> 24 -> -49

36 -> 103 -> 25 -> -132

36 -> 103 -> 27 -> -19

36 -> 103 -> 27 -> -20

36 -> 103 -> 27 -> -32

36 -> 103 -> 27 -> -62

36 -> 103 -> 27 -> -68

36 -> 103 -> 27 -> -73

36 -> 103 -> 27 -> -81

36 -> 103 -> 27 -> -98

36 -> 103 -> 27 -> -99

36 -> 103 -> 27 -> -101

36 -> 103 -> 27 -> -107

36 -> 103 -> 27 -> -124

36 -> 103 -> 27 -> -133

36 -> 103 -> 27 -> -145

36 -> 103 -> 28 -> -81

36 -> 103 -> 29 -> -133

36 -> 103 -> 30 -> -1

36 -> 103 -> 30 -> -3

36 -> 103 -> 30 -> -32

36 -> 103 -> 30 -> -39

36 -> 103 -> 30 -> -63

36 -> 103 -> 30 -> -73

36 -> 103 -> 30 -> -81

36 -> 103 -> 30 -> -99

36 -> 103 -> 30 -> -101

36 -> 103 -> 30 -> -106

36 -> 103 -> 30 -> -117

36 -> 103 -> 30 -> -133

36 -> 103 -> 30 -> -137

36 -> 103 -> 30 -> -150

36 -> 103 -> 30 -> -156

36 -> 103 -> 32 -> -9

36 -> 103 -> 33 -> -145

36 -> 103 -> 34 -> -32

36 -> 103 -> 34 -> -71

36 -> 103 -> 34 -> -113

36 -> 103 -> 37 -> -73

36 -> 103 -> 39 -> -101

36 -> 103 -> 41 -> -6

36 -> 103 -> 42 -> -133

36 -> 103 -> 45 -> -73

36 -> 103 -> 48 -> -99

36 -> 103 -> 49 -> -15

36 -> 103 -> 55 -> -12

36 -> 103 -> 60 -> -64

36 -> 103 -> 61 -> -27

36 -> 103 -> 64 -> -10

36 -> 103 -> 65 -> -4

36 -> 103 -> 66 -> -136

36 -> 103 -> 67 -> -135

36 -> 103 -> 68 -> -73

36 -> 103 -> 69 -> -77

36 -> 103 -> 73 -> -32

36 -> 103 -> 74 -> -112

36 -> 103 -> 75 -> -1

36 -> 103 -> 75 -> -29

36 -> 103 -> 76 -> -81

36 -> 103 -> 77 -> -73

36 -> 103 -> 79 -> -73

36 -> 103 -> 84 -> -73

36 -> 103 -> 87 -> -73

36 -> 103 -> 88 -> -83

36 -> 103 -> 91 -> -101

36 -> 103 -> 92 -> -133

36 -> 103 -> 97 -> -15

36 -> 103 -> 105 -> -32

36 -> 103 -> 105 -> -146

36 -> 103 -> 107 -> -73

36 -> 103 -> 107 -> -99

36 -> 103 -> 108 -> -80

36 -> 103 -> 113 -> -73

36 -> 103 -> 116 -> -73

36 -> 103 -> 117 -> -73

36 -> 103 -> 117 -> -101

36 -> 103 -> 118 -> -73

36 -> 103 -> 120 -> -133

36 -> 103 -> 122 -> -56

36 -> 103 -> 123 -> -73

36 -> 103 -> 128 -> -32

36 -> 103 -> 128 -> -95

36 -> 103 -> 129 -> -32

36 -> 103 -> 130 -> -73

36 -> 103 -> 136 -> -20

36 -> 103 -> 138 -> -20

36 -> 103 -> 138 -> -61

36 -> 103 -> 138 -> -73

36 -> 103 -> 138 -> -98

36 -> 103 -> 138 -> -133

36 -> 103 -> 139 -> -15

36 -> 103 -> 142 -> -154

36 -> 103 -> 148 -> -32

36 -> 103 -> 149 -> -73

36 -> 103 -> 150 -> -91

36 -> 103 -> 153 -> -2

36 -> 103 -> 157 -> -26

36 -> 103 -> 157 -> -28

36 -> 103 -> 157 -> -32

36 -> 103 -> 157 -> -68

36 -> 103 -> 157 -> -86

36 -> 103 -> 157 -> -99

36 -> 103 -> 157 -> -101

36 -> 103 -> 157 -> -118

36 -> 103 -> 157 -> -119

36 -> 103 -> 157 -> -120

36 -> 103 -> 157 -> -121

36 -> 103 -> 157 -> -122

36 -> 103 -> 157 -> -133

36 -> 103 -> 160 -> -129

36 -> 103 -> 161 -> -78

36 -> 103 -> 162 -> -133

36 -> 103 -> 163 -> -8

36 -> 103 -> 170 -> -138

36 -> 103 -> 171 -> -13

36 -> 103 -> 172 -> -58

36 -> 103 -> 176 -> -33

36 -> 103 -> 177 -> -106

36 -> 103 -> 179 -> -22

36 -> 103 -> 179 -> -147

36 -> 103 -> 180 -> -20

36 -> 103 -> 181 -> -84

36 -> 103 -> 186 -> -99

36 -> 103 -> 188 -> -24

36 -> 103 -> 189 -> -73

36 -> 103 -> -78

36 -> 103 -> -78 -> -1

36 -> 103 -> -78 -> -3

36 -> 103 -> -78 -> -4

36 -> 103 -> -78 -> -6

36 -> 103 -> -78 -> -8

36 -> 103 -> -78 -> -9

36 -> 103 -> -78 -> -10

36 -> 103 -> -78 -> -17

36 -> 103 -> -78 -> -19

36 -> 103 -> -78 -> -20

36 -> 103 -> -78 -> -22

36 -> 103 -> -78 -> -24

36 -> 103 -> -78 -> -26

36 -> 103 -> -78 -> -27

36 -> 103 -> -78 -> -28

36 -> 103 -> -78 -> -29

36 -> 103 -> -78 -> -30

36 -> 103 -> -78 -> -31

36 -> 103 -> -78 -> -32

36 -> 103 -> -78 -> -33

36 -> 103 -> -78 -> -35

36 -> 103 -> -78 -> -36

36 -> 103 -> -78 -> -39

36 -> 103 -> -78 -> -43

36 -> 103 -> -78 -> -44

36 -> 103 -> -78 -> -45

36 -> 103 -> -78 -> -46

36 -> 103 -> -78 -> -47

36 -> 103 -> -78 -> -48

36 -> 103 -> -78 -> -49

36 -> 103 -> -78 -> -51

36 -> 103 -> -78 -> -56

36 -> 103 -> -78 -> -58

36 -> 103 -> -78 -> -60

36 -> 103 -> -78 -> -61

36 -> 103 -> -78 -> -62

36 -> 103 -> -78 -> -63

36 -> 103 -> -78 -> -64

36 -> 103 -> -78 -> -66

36 -> 103 -> -78 -> -68

36 -> 103 -> -78 -> -69

36 -> 103 -> -78 -> -73

36 -> 103 -> -78 -> -77

36 -> 103 -> -78 -> -81

36 -> 103 -> -78 -> -83

36 -> 103 -> -78 -> -84

36 -> 103 -> -78 -> -86

36 -> 103 -> -78 -> -88

36 -> 103 -> -78 -> -89

36 -> 103 -> -78 -> -91

36 -> 103 -> -78 -> -92

36 -> 103 -> -78 -> -95

36 -> 103 -> -78 -> -96

36 -> 103 -> -78 -> -98

36 -> 103 -> -78 -> -99

36 -> 103 -> -78 -> -100

36 -> 103 -> -78 -> -101

36 -> 103 -> -78 -> -106

36 -> 103 -> -78 -> -107

36 -> 103 -> -78 -> -114

36 -> 103 -> -78 -> -117

36 -> 103 -> -78 -> -118

36 -> 103 -> -78 -> -119

36 -> 103 -> -78 -> -120

36 -> 103 -> -78 -> -121

36 -> 103 -> -78 -> -122

36 -> 103 -> -78 -> -124

36 -> 103 -> -78 -> -127

36 -> 103 -> -78 -> -131

36 -> 103 -> -78 -> -132

36 -> 103 -> -78 -> -133

36 -> 103 -> -78 -> -135

36 -> 103 -> -78 -> -136

36 -> 103 -> -78 -> -137

36 -> 103 -> -78 -> -141

36 -> 103 -> -78 -> -144

36 -> 103 -> -78 -> -145

36 -> 103 -> -78 -> -146

36 -> 103 -> -78 -> -147

36 -> 103 -> -78 -> -150

36 -> 103 -> -78 -> -154

36 -> 103 -> -78 -> -155

36 -> 103 -> -78 -> -156

36 -> 105 -> 3 -> -32

36 -> 105 -> 3 -> -42

36 -> 105 -> 4 -> -101

36 -> 105 -> 17 -> -32

36 -> 105 -> 21 -> -73

36 -> 105 -> 21 -> -101

36 -> 105 -> 21 -> -154

36 -> 105 -> 27 -> -19

36 -> 105 -> 27 -> -20

36 -> 105 -> 27 -> -32

36 -> 105 -> 27 -> -62

36 -> 105 -> 27 -> -68

36 -> 105 -> 27 -> -73

36 -> 105 -> 27 -> -81

36 -> 105 -> 27 -> -98

36 -> 105 -> 27 -> -99

36 -> 105 -> 27 -> -101

36 -> 105 -> 27 -> -107

36 -> 105 -> 27 -> -124

36 -> 105 -> 27 -> -133

36 -> 105 -> 27 -> -145

36 -> 105 -> 29 -> -133

36 -> 105 -> 30 -> -1

36 -> 105 -> 30 -> -3

36 -> 105 -> 30 -> -32

36 -> 105 -> 30 -> -39

36 -> 105 -> 30 -> -63

36 -> 105 -> 30 -> -73

36 -> 105 -> 30 -> -81

36 -> 105 -> 30 -> -99

36 -> 105 -> 30 -> -101

36 -> 105 -> 30 -> -106

36 -> 105 -> 30 -> -117

36 -> 105 -> 30 -> -133

36 -> 105 -> 30 -> -137

36 -> 105 -> 30 -> -150

36 -> 105 -> 30 -> -156

36 -> 105 -> 38 -> -55

36 -> 105 -> 38 -> -56

36 -> 105 -> 38 -> -105

36 -> 105 -> 39 -> -101

36 -> 105 -> 40 -> -73

36 -> 105 -> 44 -> -29

36 -> 105 -> 44 -> -48

36 -> 105 -> 44 -> -81

36 -> 105 -> 44 -> -89

36 -> 105 -> 44 -> -99

36 -> 105 -> 44 -> -114

36 -> 105 -> 60 -> -64

36 -> 105 -> 61 -> -27

36 -> 105 -> 73 -> -32

36 -> 105 -> 75 -> -1

36 -> 105 -> 75 -> -29

36 -> 105 -> 76 -> -81

36 -> 105 -> 77 -> -73

36 -> 105 -> 89 -> -6

36 -> 105 -> 89 -> -73

36 -> 105 -> 89 -> -81

36 -> 105 -> 89 -> -83

36 -> 105 -> 89 -> -86

36 -> 105 -> 89 -> -99

36 -> 105 -> 89 -> -101

36 -> 105 -> 89 -> -114

36 -> 105 -> 89 -> -155

36 -> 105 -> 92 -> -133

36 -> 105 -> 100 -> -32

36 -> 105 -> 100 -> -38

36 -> 105 -> 103 -> -78

36 -> 105 -> 107 -> -73

36 -> 105 -> 107 -> -99

36 -> 105 -> 109 -> -20

36 -> 105 -> 109 -> -73

36 -> 105 -> 109 -> -99

36 -> 105 -> 109 -> -101

36 -> 105 -> 110 -> -56

36 -> 105 -> 117 -> -73

36 -> 105 -> 117 -> -101

36 -> 105 -> 122 -> -56

36 -> 105 -> 128 -> -32

36 -> 105 -> 128 -> -95

36 -> 105 -> 129 -> -32

36 -> 105 -> 133 -> -101

36 -> 105 -> 134 -> -99

36 -> 105 -> 136 -> -20

36 -> 105 -> 138 -> -20

36 -> 105 -> 138 -> -61

36 -> 105 -> 138 -> -73

36 -> 105 -> 138 -> -98

36 -> 105 -> 138 -> -133

36 -> 105 -> 140 -> -99

36 -> 105 -> 140 -> -133

36 -> 105 -> 141 -> -51

36 -> 105 -> 141 -> -100

36 -> 105 -> 141 -> -127

36 -> 105 -> 146 -> -20

36 -> 105 -> 148 -> -32

36 -> 105 -> 151 -> -101

36 -> 105 -> 156 -> -32

36 -> 105 -> 156 -> -44

36 -> 105 -> 157 -> -26

36 -> 105 -> 157 -> -28

36 -> 105 -> 157 -> -32

36 -> 105 -> 157 -> -68

36 -> 105 -> 157 -> -86

36 -> 105 -> 157 -> -99

36 -> 105 -> 157 -> -101

36 -> 105 -> 157 -> -118

36 -> 105 -> 157 -> -119

36 -> 105 -> 157 -> -120

36 -> 105 -> 157 -> -121

36 -> 105 -> 157 -> -122

36 -> 105 -> 157 -> -133

36 -> 105 -> 161 -> -78

36 -> 105 -> 173 -> -154

36 -> 105 -> 179 -> -22

36 -> 105 -> 179 -> -147

36 -> 105 -> 180 -> -20

36 -> 105 -> 182 -> -73

36 -> 105 -> 186 -> -99

36 -> 105 -> -32

36 -> 105 -> -32 -> -1

36 -> 105 -> -32 -> -3

36 -> 105 -> -32 -> -14

36 -> 105 -> -32 -> -17

36 -> 105 -> -32 -> -19

36 -> 105 -> -32 -> -20

36 -> 105 -> -32 -> -22

36 -> 105 -> -32 -> -29

36 -> 105 -> -32 -> -30

36 -> 105 -> -32 -> -34

36 -> 105 -> -32 -> -35

36 -> 105 -> -32 -> -37

36 -> 105 -> -32 -> -38

36 -> 105 -> -32 -> -39

36 -> 105 -> -32 -> -43

36 -> 105 -> -32 -> -55

36 -> 105 -> -32 -> -56

36 -> 105 -> -32 -> -62

36 -> 105 -> -32 -> -63

36 -> 105 -> -32 -> -64

36 -> 105 -> -32 -> -68

36 -> 105 -> -32 -> -71

36 -> 105 -> -32 -> -73

36 -> 105 -> -32 -> -78

36 -> 105 -> -32 -> -81

36 -> 105 -> -32 -> -86

36 -> 105 -> -32 -> -92

36 -> 105 -> -32 -> -94

36 -> 105 -> -32 -> -95

36 -> 105 -> -32 -> -98

36 -> 105 -> -32 -> -99

36 -> 105 -> -32 -> -101

36 -> 105 -> -32 -> -103

36 -> 105 -> -32 -> -105

36 -> 105 -> -32 -> -106

36 -> 105 -> -32 -> -107

36 -> 105 -> -32 -> -113

36 -> 105 -> -32 -> -117

36 -> 105 -> -32 -> -124

36 -> 105 -> -32 -> -125

36 -> 105 -> -32 -> -133

36 -> 105 -> -32 -> -137

36 -> 105 -> -32 -> -144

36 -> 105 -> -32 -> -145

36 -> 105 -> -32 -> -146

36 -> 105 -> -32 -> -147

36 -> 105 -> -32 -> -150

36 -> 105 -> -32 -> -154

36 -> 105 -> -32 -> -156

36 -> 105 -> -146

36 -> 105 -> -146 -> -1

36 -> 105 -> -146 -> -3

36 -> 105 -> -146 -> -4

36 -> 105 -> -146 -> -8

36 -> 105 -> -146 -> -9

36 -> 105 -> -146 -> -10

36 -> 105 -> -146 -> -19

36 -> 105 -> -146 -> -20

36 -> 105 -> -146 -> -24

36 -> 105 -> -146 -> -29

36 -> 105 -> -146 -> -31

36 -> 105 -> -146 -> -32

36 -> 105 -> -146 -> -33

36 -> 105 -> -146 -> -39

36 -> 105 -> -146 -> -42

36 -> 105 -> -146 -> -44

36 -> 105 -> -146 -> -45

36 -> 105 -> -146 -> -46

36 -> 105 -> -146 -> -47

36 -> 105 -> -146 -> -48

36 -> 105 -> -146 -> -49

36 -> 105 -> -146 -> -55

36 -> 105 -> -146 -> -56

36 -> 105 -> -146 -> -58

36 -> 105 -> -146 -> -60

36 -> 105 -> -146 -> -62

36 -> 105 -> -146 -> -63

36 -> 105 -> -146 -> -64

36 -> 105 -> -146 -> -66

36 -> 105 -> -146 -> -68

36 -> 105 -> -146 -> -69

36 -> 105 -> -146 -> -73

36 -> 105 -> -146 -> -77

36 -> 105 -> -146 -> -78

36 -> 105 -> -146 -> -81

36 -> 105 -> -146 -> -84

36 -> 105 -> -146 -> -91

36 -> 105 -> -146 -> -95

36 -> 105 -> -146 -> -96

36 -> 105 -> -146 -> -98

36 -> 105 -> -146 -> -99

36 -> 105 -> -146 -> -101

36 -> 105 -> -146 -> -105

36 -> 105 -> -146 -> -106

36 -> 105 -> -146 -> -107

36 -> 105 -> -146 -> -114

36 -> 105 -> -146 -> -117

36 -> 105 -> -146 -> -120

36 -> 105 -> -146 -> -124

36 -> 105 -> -146 -> -131

36 -> 105 -> -146 -> -132

36 -> 105 -> -146 -> -133

36 -> 105 -> -146 -> -135

36 -> 105 -> -146 -> -136

36 -> 105 -> -146 -> -137

36 -> 105 -> -146 -> -141

36 -> 105 -> -146 -> -142

36 -> 105 -> -146 -> -150

36 -> 105 -> -146 -> -154

36 -> 105 -> -146 -> -156

36 -> 107 -> 3 -> -32

36 -> 107 -> 3 -> -42

36 -> 107 -> 4 -> -101

36 -> 107 -> 6 -> -73

36 -> 107 -> 16 -> -5

36 -> 107 -> 19 -> -73

36 -> 107 -> 21 -> -73

36 -> 107 -> 21 -> -101

36 -> 107 -> 21 -> -154

36 -> 107 -> 27 -> -19

36 -> 107 -> 27 -> -20

36 -> 107 -> 27 -> -32

36 -> 107 -> 27 -> -62

36 -> 107 -> 27 -> -68

36 -> 107 -> 27 -> -73

36 -> 107 -> 27 -> -81

36 -> 107 -> 27 -> -98

36 -> 107 -> 27 -> -99

36 -> 107 -> 27 -> -101

36 -> 107 -> 27 -> -107

36 -> 107 -> 27 -> -124

36 -> 107 -> 27 -> -133

36 -> 107 -> 27 -> -145

36 -> 107 -> 29 -> -133

36 -> 107 -> 30 -> -1

36 -> 107 -> 30 -> -3

36 -> 107 -> 30 -> -32

36 -> 107 -> 30 -> -39

36 -> 107 -> 30 -> -63

36 -> 107 -> 30 -> -73

36 -> 107 -> 30 -> -81

36 -> 107 -> 30 -> -99

36 -> 107 -> 30 -> -101

36 -> 107 -> 30 -> -106

36 -> 107 -> 30 -> -117

36 -> 107 -> 30 -> -133

36 -> 107 -> 30 -> -137

36 -> 107 -> 30 -> -150

36 -> 107 -> 30 -> -156

36 -> 107 -> 34 -> -32

36 -> 107 -> 34 -> -71

36 -> 107 -> 34 -> -113

36 -> 107 -> 38 -> -55

36 -> 107 -> 38 -> -56

36 -> 107 -> 38 -> -105

36 -> 107 -> 39 -> -101

36 -> 107 -> 40 -> -73

36 -> 107 -> 43 -> -73

36 -> 107 -> 43 -> -99

36 -> 107 -> 43 -> -101

36 -> 107 -> 44 -> -29

36 -> 107 -> 44 -> -48

36 -> 107 -> 44 -> -81

36 -> 107 -> 44 -> -89

36 -> 107 -> 44 -> -99

36 -> 107 -> 44 -> -114

36 -> 107 -> 45 -> -73

36 -> 107 -> 48 -> -99

36 -> 107 -> 52 -> -32

36 -> 107 -> 60 -> -64

36 -> 107 -> 61 -> -27

36 -> 107 -> 68 -> -73

36 -> 107 -> 75 -> -1

36 -> 107 -> 75 -> -29

36 -> 107 -> 76 -> -81

36 -> 107 -> 77 -> -73

36 -> 107 -> 79 -> -73

36 -> 107 -> 81 -> -73

36 -> 107 -> 84 -> -73

36 -> 107 -> 85 -> -12

36 -> 107 -> 87 -> -73

36 -> 107 -> 92 -> -133

36 -> 107 -> 99 -> -73

36 -> 107 -> 102 -> -116

36 -> 107 -> 103 -> -78

36 -> 107 -> 105 -> -32

36 -> 107 -> 105 -> -146

36 -> 107 -> 109 -> -20

36 -> 107 -> 109 -> -73

36 -> 107 -> 109 -> -99

36 -> 107 -> 109 -> -101

36 -> 107 -> 110 -> -56

36 -> 107 -> 113 -> -73

36 -> 107 -> 115 -> -17

36 -> 107 -> 115 -> -32

36 -> 107 -> 115 -> -68

36 -> 107 -> 115 -> -73

36 -> 107 -> 115 -> -92

36 -> 107 -> 116 -> -73

36 -> 107 -> 117 -> -73

36 -> 107 -> 117 -> -101

36 -> 107 -> 118 -> -73

36 -> 107 -> 122 -> -56

36 -> 107 -> 123 -> -73

36 -> 107 -> 128 -> -32

36 -> 107 -> 128 -> -95

36 -> 107 -> 129 -> -32

36 -> 107 -> 130 -> -73

36 -> 107 -> 134 -> -99

36 -> 107 -> 136 -> -20

36 -> 107 -> 138 -> -20

36 -> 107 -> 138 -> -61

36 -> 107 -> 138 -> -73

36 -> 107 -> 138 -> -98

36 -> 107 -> 138 -> -133

36 -> 107 -> 140 -> -99

36 -> 107 -> 140 -> -133

36 -> 107 -> 146 -> -20

36 -> 107 -> 149 -> -73

36 -> 107 -> 152 -> -36

36 -> 107 -> 157 -> -26

36 -> 107 -> 157 -> -28

36 -> 107 -> 157 -> -32

36 -> 107 -> 157 -> -68

36 -> 107 -> 157 -> -86

36 -> 107 -> 157 -> -99

36 -> 107 -> 157 -> -101

36 -> 107 -> 157 -> -118

36 -> 107 -> 157 -> -119

36 -> 107 -> 157 -> -120

36 -> 107 -> 157 -> -121

36 -> 107 -> 157 -> -122

36 -> 107 -> 157 -> -133

36 -> 107 -> 161 -> -78

36 -> 107 -> 168 -> -73

36 -> 107 -> 168 -> -102

36 -> 107 -> 169 -> -99

36 -> 107 -> 174 -> -114

36 -> 107 -> 179 -> -22

36 -> 107 -> 179 -> -147

36 -> 107 -> 180 -> -20

36 -> 107 -> 182 -> -73

36 -> 107 -> 184 -> -86

36 -> 107 -> 184 -> -101

36 -> 107 -> 184 -> -133

36 -> 107 -> 186 -> -99

36 -> 107 -> 189 -> -73

36 -> 107 -> -73

36 -> 107 -> -73 -> -1

36 -> 107 -> -73 -> -3

36 -> 107 -> -73 -> -6

36 -> 107 -> -73 -> -17

36 -> 107 -> -73 -> -19

36 -> 107 -> -73 -> -20

36 -> 107 -> -73 -> -22

36 -> 107 -> -73 -> -26

36 -> 107 -> -73 -> -27

36 -> 107 -> -73 -> -28

36 -> 107 -> -73 -> -29

36 -> 107 -> -73 -> -30

36 -> 107 -> -73 -> -32

36 -> 107 -> -73 -> -35

36 -> 107 -> -73 -> -39

36 -> 107 -> -73 -> -43

36 -> 107 -> -73 -> -48

36 -> 107 -> -73 -> -56

36 -> 107 -> -73 -> -61

36 -> 107 -> -73 -> -62

36 -> 107 -> -73 -> -63

36 -> 107 -> -73 -> -64

36 -> 107 -> -73 -> -68

36 -> 107 -> -73 -> -71

36 -> 107 -> -73 -> -78

36 -> 107 -> -73 -> -81

36 -> 107 -> -73 -> -83

36 -> 107 -> -73 -> -86

36 -> 107 -> -73 -> -88

36 -> 107 -> -73 -> -89

36 -> 107 -> -73 -> -92

36 -> 107 -> -73 -> -95

36 -> 107 -> -73 -> -98

36 -> 107 -> -73 -> -99

36 -> 107 -> -73 -> -101

36 -> 107 -> -73 -> -106

36 -> 107 -> -73 -> -107

36 -> 107 -> -73 -> -113

36 -> 107 -> -73 -> -114

36 -> 107 -> -73 -> -117

36 -> 107 -> -73 -> -118

36 -> 107 -> -73 -> -119

36 -> 107 -> -73 -> -121

36 -> 107 -> -73 -> -122

36 -> 107 -> -73 -> -124

36 -> 107 -> -73 -> -133

36 -> 107 -> -73 -> -137

36 -> 107 -> -73 -> -144

36 -> 107 -> -73 -> -145

36 -> 107 -> -73 -> -146

36 -> 107 -> -73 -> -147

36 -> 107 -> -73 -> -150

36 -> 107 -> -73 -> -154

36 -> 107 -> -73 -> -155

36 -> 107 -> -73 -> -156

36 -> 107 -> -99

36 -> 107 -> -99 -> -1

36 -> 107 -> -99 -> -3

36 -> 107 -> -99 -> -6

36 -> 107 -> -99 -> -17

36 -> 107 -> -99 -> -18

36 -> 107 -> -99 -> -19

36 -> 107 -> -99 -> -20

36 -> 107 -> -99 -> -22

36 -> 107 -> -99 -> -26

36 -> 107 -> -99 -> -27

36 -> 107 -> -99 -> -28

36 -> 107 -> -99 -> -29

36 -> 107 -> -99 -> -30

36 -> 107 -> -99 -> -32

36 -> 107 -> -99 -> -35

36 -> 107 -> -99 -> -36

36 -> 107 -> -99 -> -39

36 -> 107 -> -99 -> -41

36 -> 107 -> -99 -> -43

36 -> 107 -> -99 -> -48

36 -> 107 -> -99 -> -51

36 -> 107 -> -99 -> -56

36 -> 107 -> -99 -> -61

36 -> 107 -> -99 -> -62

36 -> 107 -> -99 -> -63

36 -> 107 -> -99 -> -64

36 -> 107 -> -99 -> -68

36 -> 107 -> -99 -> -73

36 -> 107 -> -99 -> -78

36 -> 107 -> -99 -> -81

36 -> 107 -> -99 -> -83

36 -> 107 -> -99 -> -86

36 -> 107 -> -99 -> -88

36 -> 107 -> -99 -> -89

36 -> 107 -> -99 -> -92

36 -> 107 -> -99 -> -95

36 -> 107 -> -99 -> -97

36 -> 107 -> -99 -> -98

36 -> 107 -> -99 -> -100

36 -> 107 -> -99 -> -101

36 -> 107 -> -99 -> -106

36 -> 107 -> -99 -> -107

36 -> 107 -> -99 -> -109

36 -> 107 -> -99 -> -111

36 -> 107 -> -99 -> -114

36 -> 107 -> -99 -> -117

36 -> 107 -> -99 -> -118

36 -> 107 -> -99 -> -119

36 -> 107 -> -99 -> -121

36 -> 107 -> -99 -> -122

36 -> 107 -> -99 -> -124

36 -> 107 -> -99 -> -127

36 -> 107 -> -99 -> -133

36 -> 107 -> -99 -> -137

36 -> 107 -> -99 -> -144

36 -> 107 -> -99 -> -145

36 -> 107 -> -99 -> -146

36 -> 107 -> -99 -> -147

36 -> 107 -> -99 -> -150

36 -> 107 -> -99 -> -151

36 -> 107 -> -99 -> -154

36 -> 107 -> -99 -> -155

36 -> 107 -> -99 -> -156

36 -> 113 -> 2 -> -32

36 -> 113 -> 4 -> -101

36 -> 113 -> 6 -> -73

36 -> 113 -> 7 -> -54

36 -> 113 -> 9 -> -6

36 -> 113 -> 12 -> -32

36 -> 113 -> 13 -> -32

36 -> 113 -> 13 -> -73

36 -> 113 -> 14 -> -54

36 -> 113 -> 15 -> -68

36 -> 113 -> 16 -> -5

36 -> 113 -> 17 -> -32

36 -> 113 -> 18 -> -73

36 -> 113 -> 18 -> -89

36 -> 113 -> 19 -> -73

36 -> 113 -> 21 -> -73

36 -> 113 -> 21 -> -101

36 -> 113 -> 21 -> -154

36 -> 113 -> 22 -> -152

36 -> 113 -> 24 -> -49

36 -> 113 -> 25 -> -132

36 -> 113 -> 26 -> -14

36 -> 113 -> 28 -> -81

36 -> 113 -> 29 -> -133

36 -> 113 -> 31 -> -1

36 -> 113 -> 32 -> -9

36 -> 113 -> 33 -> -145

36 -> 113 -> 37 -> -73

36 -> 113 -> 39 -> -101

36 -> 113 -> 40 -> -73

36 -> 113 -> 41 -> -6

36 -> 113 -> 42 -> -133

36 -> 113 -> 43 -> -73

36 -> 113 -> 43 -> -99

36 -> 113 -> 43 -> -101

36 -> 113 -> 45 -> -73

36 -> 113 -> 46 -> -32

36 -> 113 -> 48 -> -99

36 -> 113 -> 49 -> -15

36 -> 113 -> 50 -> -54

36 -> 113 -> 51 -> -2

36 -> 113 -> 52 -> -32

36 -> 113 -> 53 -> -133

36 -> 113 -> 54 -> -53

36 -> 113 -> 55 -> -12

36 -> 113 -> 56 -> -115

36 -> 113 -> 57 -> -103

36 -> 113 -> 58 -> -90

36 -> 113 -> 59 -> -148

36 -> 113 -> 60 -> -64

36 -> 113 -> 61 -> -27

36 -> 113 -> 62 -> -99

36 -> 113 -> 63 -> -32

36 -> 113 -> 64 -> -10

36 -> 113 -> 65 -> -4

36 -> 113 -> 66 -> -136

36 -> 113 -> 67 -> -135

36 -> 113 -> 68 -> -73

36 -> 113 -> 69 -> -77

36 -> 113 -> 70 -> -52

36 -> 113 -> 71 -> -32

36 -> 113 -> 73 -> -32

36 -> 113 -> 74 -> -112

36 -> 113 -> 76 -> -81

36 -> 113 -> 77 -> -73

36 -> 113 -> 78 -> -73

36 -> 113 -> 79 -> -73

36 -> 113 -> 80 -> -32

36 -> 113 -> 81 -> -73

36 -> 113 -> 83 -> -125

36 -> 113 -> 84 -> -73

36 -> 113 -> 85 -> -12

36 -> 113 -> 87 -> -73

36 -> 113 -> 88 -> -83

36 -> 113 -> 90 -> -103

36 -> 113 -> 91 -> -101

36 -> 113 -> 92 -> -133

36 -> 113 -> 93 -> -145

36 -> 113 -> 94 -> -13

36 -> 113 -> 96 -> -73

36 -> 113 -> 97 -> -15

36 -> 113 -> 99 -> -73

36 -> 113 -> 101 -> -64

36 -> 113 -> 102 -> -116

36 -> 113 -> 103 -> -78

36 -> 113 -> 104 -> -2

36 -> 113 -> 106 -> -65

36 -> 113 -> 107 -> -73

36 -> 113 -> 107 -> -99

36 -> 113 -> 108 -> -80

36 -> 113 -> 110 -> -56

36 -> 113 -> 112 -> -54

36 -> 113 -> 114 -> -73

36 -> 113 -> 116 -> -73

36 -> 113 -> 117 -> -73

36 -> 113 -> 117 -> -101

36 -> 113 -> 118 -> -73

36 -> 113 -> 120 -> -133

36 -> 113 -> 122 -> -56

36 -> 113 -> 123 -> -73

36 -> 113 -> 125 -> -104

36 -> 113 -> 126 -> -59

36 -> 113 -> 127 -> -93

36 -> 113 -> 129 -> -32

36 -> 113 -> 130 -> -73

36 -> 113 -> 132 -> -32

36 -> 113 -> 133 -> -101

36 -> 113 -> 134 -> -99

36 -> 113 -> 135 -> -21

36 -> 113 -> 135 -> -73

36 -> 113 -> 135 -> -139

36 -> 113 -> 136 -> -20

36 -> 113 -> 139 -> -15

36 -> 113 -> 142 -> -154

36 -> 113 -> 143 -> -50

36 -> 113 -> 145 -> -125

36 -> 113 -> 146 -> -20

36 -> 113 -> 147 -> -32

36 -> 113 -> 148 -> -32

36 -> 113 -> 149 -> -73

36 -> 113 -> 150 -> -91

36 -> 113 -> 151 -> -101

36 -> 113 -> 152 -> -36

36 -> 113 -> 153 -> -2

36 -> 113 -> 155 -> -73

36 -> 113 -> 158 -> -72

36 -> 113 -> 160 -> -129

36 -> 113 -> 161 -> -78

36 -> 113 -> 162 -> -133

36 -> 113 -> 163 -> -8

36 -> 113 -> 164 -> -134

36 -> 113 -> 165 -> -54

36 -> 113 -> 168 -> -73

36 -> 113 -> 168 -> -102

36 -> 113 -> 169 -> -99

36 -> 113 -> 170 -> -138

36 -> 113 -> 171 -> -13

36 -> 113 -> 172 -> -58

36 -> 113 -> 173 -> -154

36 -> 113 -> 174 -> -114

36 -> 113 -> 176 -> -33

36 -> 113 -> 177 -> -106

36 -> 113 -> 178 -> -69

36 -> 113 -> 180 -> -20

36 -> 113 -> 181 -> -84

36 -> 113 -> 182 -> -73

36 -> 113 -> 186 -> -99

36 -> 113 -> 187 -> -53

36 -> 113 -> 188 -> -24

36 -> 113 -> 189 -> -73

36 -> 113 -> -73

36 -> 113 -> -73 -> -1

36 -> 113 -> -73 -> -3

36 -> 113 -> -73 -> -6

36 -> 113 -> -73 -> -17

36 -> 113 -> -73 -> -19

36 -> 113 -> -73 -> -20

36 -> 113 -> -73 -> -22

36 -> 113 -> -73 -> -26

36 -> 113 -> -73 -> -27

36 -> 113 -> -73 -> -28

36 -> 113 -> -73 -> -29

36 -> 113 -> -73 -> -30

36 -> 113 -> -73 -> -32

36 -> 113 -> -73 -> -35

36 -> 113 -> -73 -> -39

36 -> 113 -> -73 -> -43

36 -> 113 -> -73 -> -48

36 -> 113 -> -73 -> -56

36 -> 113 -> -73 -> -61

36 -> 113 -> -73 -> -62

36 -> 113 -> -73 -> -63

36 -> 113 -> -73 -> -64

36 -> 113 -> -73 -> -68

36 -> 113 -> -73 -> -71

36 -> 113 -> -73 -> -78

36 -> 113 -> -73 -> -81

36 -> 113 -> -73 -> -83

36 -> 113 -> -73 -> -86

36 -> 113 -> -73 -> -88

36 -> 113 -> -73 -> -89

36 -> 113 -> -73 -> -92

36 -> 113 -> -73 -> -95

36 -> 113 -> -73 -> -98

36 -> 113 -> -73 -> -99

36 -> 113 -> -73 -> -101

36 -> 113 -> -73 -> -106

36 -> 113 -> -73 -> -107

36 -> 113 -> -73 -> -113

36 -> 113 -> -73 -> -114

36 -> 113 -> -73 -> -117

36 -> 113 -> -73 -> -118

36 -> 113 -> -73 -> -119

36 -> 113 -> -73 -> -121

36 -> 113 -> -73 -> -122

36 -> 113 -> -73 -> -124

36 -> 113 -> -73 -> -133

36 -> 113 -> -73 -> -137

36 -> 113 -> -73 -> -144

36 -> 113 -> -73 -> -145

36 -> 113 -> -73 -> -146

36 -> 113 -> -73 -> -147

36 -> 113 -> -73 -> -150

36 -> 113 -> -73 -> -154

36 -> 113 -> -73 -> -155

36 -> 113 -> -73 -> -156

36 -> 116 -> 2 -> -32

36 -> 116 -> 4 -> -101

36 -> 116 -> 6 -> -73

36 -> 116 -> 7 -> -54

36 -> 116 -> 9 -> -6

36 -> 116 -> 12 -> -32

36 -> 116 -> 13 -> -32

36 -> 116 -> 13 -> -73

36 -> 116 -> 14 -> -54

36 -> 116 -> 15 -> -68

36 -> 116 -> 16 -> -5

36 -> 116 -> 17 -> -32

36 -> 116 -> 18 -> -73

36 -> 116 -> 18 -> -89

36 -> 116 -> 19 -> -73

36 -> 116 -> 21 -> -73

36 -> 116 -> 21 -> -101

36 -> 116 -> 21 -> -154

36 -> 116 -> 22 -> -152

36 -> 116 -> 24 -> -49

36 -> 116 -> 25 -> -132

36 -> 116 -> 26 -> -14

36 -> 116 -> 28 -> -81

36 -> 116 -> 29 -> -133

36 -> 116 -> 31 -> -1

36 -> 116 -> 32 -> -9

36 -> 116 -> 33 -> -145

36 -> 116 -> 37 -> -73

36 -> 116 -> 39 -> -101

36 -> 116 -> 40 -> -73

36 -> 116 -> 41 -> -6

36 -> 116 -> 42 -> -133

36 -> 116 -> 43 -> -73

36 -> 116 -> 43 -> -99

36 -> 116 -> 43 -> -101

36 -> 116 -> 45 -> -73

36 -> 116 -> 46 -> -32

36 -> 116 -> 48 -> -99

36 -> 116 -> 49 -> -15

36 -> 116 -> 50 -> -54

36 -> 116 -> 51 -> -2

36 -> 116 -> 52 -> -32

36 -> 116 -> 53 -> -133

36 -> 116 -> 54 -> -53

36 -> 116 -> 55 -> -12

36 -> 116 -> 56 -> -115

36 -> 116 -> 57 -> -103

36 -> 116 -> 58 -> -90

36 -> 116 -> 59 -> -148

36 -> 116 -> 60 -> -64

36 -> 116 -> 61 -> -27

36 -> 116 -> 62 -> -99

36 -> 116 -> 63 -> -32

36 -> 116 -> 64 -> -10

36 -> 116 -> 65 -> -4

36 -> 116 -> 66 -> -136

36 -> 116 -> 67 -> -135

36 -> 116 -> 68 -> -73

36 -> 116 -> 69 -> -77

36 -> 116 -> 70 -> -52

36 -> 116 -> 71 -> -32

36 -> 116 -> 73 -> -32

36 -> 116 -> 74 -> -112

36 -> 116 -> 76 -> -81

36 -> 116 -> 77 -> -73

36 -> 116 -> 78 -> -73

36 -> 116 -> 79 -> -73

36 -> 116 -> 80 -> -32

36 -> 116 -> 81 -> -73

36 -> 116 -> 83 -> -125

36 -> 116 -> 84 -> -73

36 -> 116 -> 85 -> -12

36 -> 116 -> 87 -> -73

36 -> 116 -> 88 -> -83

36 -> 116 -> 90 -> -103

36 -> 116 -> 91 -> -101

36 -> 116 -> 92 -> -133

36 -> 116 -> 93 -> -145

36 -> 116 -> 94 -> -13

36 -> 116 -> 96 -> -73

36 -> 116 -> 97 -> -15

36 -> 116 -> 99 -> -73

36 -> 116 -> 101 -> -64

36 -> 116 -> 102 -> -116

36 -> 116 -> 103 -> -78

36 -> 116 -> 104 -> -2

36 -> 116 -> 106 -> -65

36 -> 116 -> 107 -> -73

36 -> 116 -> 107 -> -99

36 -> 116 -> 108 -> -80

36 -> 116 -> 110 -> -56

36 -> 116 -> 112 -> -54

36 -> 116 -> 113 -> -73

36 -> 116 -> 114 -> -73

36 -> 116 -> 117 -> -73

36 -> 116 -> 117 -> -101

36 -> 116 -> 118 -> -73

36 -> 116 -> 120 -> -133

36 -> 116 -> 122 -> -56

36 -> 116 -> 123 -> -73

36 -> 116 -> 125 -> -104

36 -> 116 -> 126 -> -59

36 -> 116 -> 127 -> -93

36 -> 116 -> 129 -> -32

36 -> 116 -> 130 -> -73

36 -> 116 -> 132 -> -32

36 -> 116 -> 133 -> -101

36 -> 116 -> 134 -> -99

36 -> 116 -> 135 -> -21

36 -> 116 -> 135 -> -73

36 -> 116 -> 135 -> -139

36 -> 116 -> 136 -> -20

36 -> 116 -> 139 -> -15

36 -> 116 -> 142 -> -154

36 -> 116 -> 143 -> -50

36 -> 116 -> 145 -> -125

36 -> 116 -> 146 -> -20

36 -> 116 -> 147 -> -32

36 -> 116 -> 148 -> -32

36 -> 116 -> 149 -> -73

36 -> 116 -> 150 -> -91

36 -> 116 -> 151 -> -101

36 -> 116 -> 152 -> -36

36 -> 116 -> 153 -> -2

36 -> 116 -> 155 -> -73

36 -> 116 -> 158 -> -72

36 -> 116 -> 160 -> -129

36 -> 116 -> 161 -> -78

36 -> 116 -> 162 -> -133

36 -> 116 -> 163 -> -8

36 -> 116 -> 164 -> -134

36 -> 116 -> 165 -> -54

36 -> 116 -> 168 -> -73

36 -> 116 -> 168 -> -102

36 -> 116 -> 169 -> -99

36 -> 116 -> 170 -> -138

36 -> 116 -> 171 -> -13

36 -> 116 -> 172 -> -58

36 -> 116 -> 173 -> -154

36 -> 116 -> 174 -> -114

36 -> 116 -> 176 -> -33

36 -> 116 -> 177 -> -106

36 -> 116 -> 178 -> -69

36 -> 116 -> 180 -> -20

36 -> 116 -> 181 -> -84

36 -> 116 -> 182 -> -73

36 -> 116 -> 186 -> -99

36 -> 116 -> 187 -> -53

36 -> 116 -> 188 -> -24

36 -> 116 -> 189 -> -73

36 -> 116 -> -73

36 -> 116 -> -73 -> -1

36 -> 116 -> -73 -> -3

36 -> 116 -> -73 -> -6

36 -> 116 -> -73 -> -17

36 -> 116 -> -73 -> -19

36 -> 116 -> -73 -> -20

36 -> 116 -> -73 -> -22

36 -> 116 -> -73 -> -26

36 -> 116 -> -73 -> -27

36 -> 116 -> -73 -> -28

36 -> 116 -> -73 -> -29

36 -> 116 -> -73 -> -30

36 -> 116 -> -73 -> -32

36 -> 116 -> -73 -> -35

36 -> 116 -> -73 -> -39

36 -> 116 -> -73 -> -43

36 -> 116 -> -73 -> -48

36 -> 116 -> -73 -> -56

36 -> 116 -> -73 -> -61

36 -> 116 -> -73 -> -62

36 -> 116 -> -73 -> -63

36 -> 116 -> -73 -> -64

36 -> 116 -> -73 -> -68

36 -> 116 -> -73 -> -71

36 -> 116 -> -73 -> -78

36 -> 116 -> -73 -> -81

36 -> 116 -> -73 -> -83

36 -> 116 -> -73 -> -86

36 -> 116 -> -73 -> -88

36 -> 116 -> -73 -> -89

36 -> 116 -> -73 -> -92

36 -> 116 -> -73 -> -95

36 -> 116 -> -73 -> -98

36 -> 116 -> -73 -> -99

36 -> 116 -> -73 -> -101

36 -> 116 -> -73 -> -106

36 -> 116 -> -73 -> -107

36 -> 116 -> -73 -> -113

36 -> 116 -> -73 -> -114

36 -> 116 -> -73 -> -117

36 -> 116 -> -73 -> -118

36 -> 116 -> -73 -> -119

36 -> 116 -> -73 -> -121

36 -> 116 -> -73 -> -122

36 -> 116 -> -73 -> -124

36 -> 116 -> -73 -> -133

36 -> 116 -> -73 -> -137

36 -> 116 -> -73 -> -144

36 -> 116 -> -73 -> -145

36 -> 116 -> -73 -> -146

36 -> 116 -> -73 -> -147

36 -> 116 -> -73 -> -150

36 -> 116 -> -73 -> -154

36 -> 116 -> -73 -> -155

36 -> 116 -> -73 -> -156

36 -> 118 -> 2 -> -32

36 -> 118 -> 4 -> -101

36 -> 118 -> 6 -> -73

36 -> 118 -> 7 -> -54

36 -> 118 -> 9 -> -6

36 -> 118 -> 12 -> -32

36 -> 118 -> 13 -> -32

36 -> 118 -> 13 -> -73

36 -> 118 -> 14 -> -54

36 -> 118 -> 15 -> -68

36 -> 118 -> 16 -> -5

36 -> 118 -> 17 -> -32

36 -> 118 -> 18 -> -73

36 -> 118 -> 18 -> -89

36 -> 118 -> 19 -> -73

36 -> 118 -> 21 -> -73

36 -> 118 -> 21 -> -101

36 -> 118 -> 21 -> -154

36 -> 118 -> 22 -> -152

36 -> 118 -> 24 -> -49

36 -> 118 -> 25 -> -132

36 -> 118 -> 26 -> -14

36 -> 118 -> 28 -> -81

36 -> 118 -> 29 -> -133

36 -> 118 -> 31 -> -1

36 -> 118 -> 32 -> -9

36 -> 118 -> 33 -> -145

36 -> 118 -> 37 -> -73

36 -> 118 -> 39 -> -101

36 -> 118 -> 40 -> -73

36 -> 118 -> 41 -> -6

36 -> 118 -> 42 -> -133

36 -> 118 -> 43 -> -73

36 -> 118 -> 43 -> -99

36 -> 118 -> 43 -> -101

36 -> 118 -> 45 -> -73

36 -> 118 -> 46 -> -32

36 -> 118 -> 48 -> -99

36 -> 118 -> 49 -> -15

36 -> 118 -> 50 -> -54

36 -> 118 -> 51 -> -2

36 -> 118 -> 52 -> -32

36 -> 118 -> 53 -> -133

36 -> 118 -> 54 -> -53

36 -> 118 -> 55 -> -12

36 -> 118 -> 56 -> -115

36 -> 118 -> 57 -> -103

36 -> 118 -> 58 -> -90

36 -> 118 -> 59 -> -148

36 -> 118 -> 60 -> -64

36 -> 118 -> 61 -> -27

36 -> 118 -> 62 -> -99

36 -> 118 -> 63 -> -32

36 -> 118 -> 64 -> -10

36 -> 118 -> 65 -> -4

36 -> 118 -> 66 -> -136

36 -> 118 -> 67 -> -135

36 -> 118 -> 68 -> -73

36 -> 118 -> 69 -> -77

36 -> 118 -> 70 -> -52

36 -> 118 -> 71 -> -32

36 -> 118 -> 73 -> -32

36 -> 118 -> 74 -> -112

36 -> 118 -> 76 -> -81

36 -> 118 -> 77 -> -73

36 -> 118 -> 78 -> -73

36 -> 118 -> 79 -> -73

36 -> 118 -> 80 -> -32

36 -> 118 -> 81 -> -73

36 -> 118 -> 83 -> -125

36 -> 118 -> 84 -> -73

36 -> 118 -> 85 -> -12

36 -> 118 -> 87 -> -73

36 -> 118 -> 88 -> -83

36 -> 118 -> 90 -> -103

36 -> 118 -> 91 -> -101

36 -> 118 -> 92 -> -133

36 -> 118 -> 93 -> -145

36 -> 118 -> 94 -> -13

36 -> 118 -> 96 -> -73

36 -> 118 -> 97 -> -15

36 -> 118 -> 99 -> -73

36 -> 118 -> 101 -> -64

36 -> 118 -> 102 -> -116

36 -> 118 -> 103 -> -78

36 -> 118 -> 104 -> -2

36 -> 118 -> 106 -> -65

36 -> 118 -> 107 -> -73

36 -> 118 -> 107 -> -99

36 -> 118 -> 108 -> -80

36 -> 118 -> 110 -> -56

36 -> 118 -> 112 -> -54

36 -> 118 -> 113 -> -73

36 -> 118 -> 114 -> -73

36 -> 118 -> 116 -> -73

36 -> 118 -> 117 -> -73

36 -> 118 -> 117 -> -101

36 -> 118 -> 120 -> -133

36 -> 118 -> 122 -> -56

36 -> 118 -> 123 -> -73

36 -> 118 -> 125 -> -104

36 -> 118 -> 126 -> -59

36 -> 118 -> 127 -> -93

36 -> 118 -> 129 -> -32

36 -> 118 -> 130 -> -73

36 -> 118 -> 132 -> -32

36 -> 118 -> 133 -> -101

36 -> 118 -> 134 -> -99

36 -> 118 -> 135 -> -21

36 -> 118 -> 135 -> -73

36 -> 118 -> 135 -> -139

36 -> 118 -> 136 -> -20

36 -> 118 -> 139 -> -15

36 -> 118 -> 142 -> -154

36 -> 118 -> 143 -> -50

36 -> 118 -> 145 -> -125

36 -> 118 -> 146 -> -20

36 -> 118 -> 147 -> -32

36 -> 118 -> 148 -> -32

36 -> 118 -> 149 -> -73

36 -> 118 -> 150 -> -91

36 -> 118 -> 151 -> -101

36 -> 118 -> 152 -> -36

36 -> 118 -> 153 -> -2

36 -> 118 -> 155 -> -73

36 -> 118 -> 158 -> -72

36 -> 118 -> 160 -> -129

36 -> 118 -> 161 -> -78

36 -> 118 -> 162 -> -133

36 -> 118 -> 163 -> -8

36 -> 118 -> 164 -> -134

36 -> 118 -> 165 -> -54

36 -> 118 -> 168 -> -73

36 -> 118 -> 168 -> -102

36 -> 118 -> 169 -> -99

36 -> 118 -> 170 -> -138

36 -> 118 -> 171 -> -13

36 -> 118 -> 172 -> -58

36 -> 118 -> 173 -> -154

36 -> 118 -> 174 -> -114

36 -> 118 -> 176 -> -33

36 -> 118 -> 177 -> -106

36 -> 118 -> 178 -> -69

36 -> 118 -> 180 -> -20

36 -> 118 -> 181 -> -84

36 -> 118 -> 182 -> -73

36 -> 118 -> 186 -> -99

36 -> 118 -> 187 -> -53

36 -> 118 -> 188 -> -24

36 -> 118 -> 189 -> -73

36 -> 118 -> -73

36 -> 118 -> -73 -> -1

36 -> 118 -> -73 -> -3

36 -> 118 -> -73 -> -6

36 -> 118 -> -73 -> -17

36 -> 118 -> -73 -> -19

36 -> 118 -> -73 -> -20

36 -> 118 -> -73 -> -22

36 -> 118 -> -73 -> -26

36 -> 118 -> -73 -> -27

36 -> 118 -> -73 -> -28

36 -> 118 -> -73 -> -29

36 -> 118 -> -73 -> -30

36 -> 118 -> -73 -> -32

36 -> 118 -> -73 -> -35

36 -> 118 -> -73 -> -39

36 -> 118 -> -73 -> -43

36 -> 118 -> -73 -> -48

36 -> 118 -> -73 -> -56

36 -> 118 -> -73 -> -61

36 -> 118 -> -73 -> -62

36 -> 118 -> -73 -> -63

36 -> 118 -> -73 -> -64

36 -> 118 -> -73 -> -68

36 -> 118 -> -73 -> -71

36 -> 118 -> -73 -> -78

36 -> 118 -> -73 -> -81

36 -> 118 -> -73 -> -83

36 -> 118 -> -73 -> -86

36 -> 118 -> -73 -> -88

36 -> 118 -> -73 -> -89

36 -> 118 -> -73 -> -92

36 -> 118 -> -73 -> -95

36 -> 118 -> -73 -> -98

36 -> 118 -> -73 -> -99

36 -> 118 -> -73 -> -101

36 -> 118 -> -73 -> -106

36 -> 118 -> -73 -> -107

36 -> 118 -> -73 -> -113

36 -> 118 -> -73 -> -114

36 -> 118 -> -73 -> -117

36 -> 118 -> -73 -> -118

36 -> 118 -> -73 -> -119

36 -> 118 -> -73 -> -121

36 -> 118 -> -73 -> -122

36 -> 118 -> -73 -> -124

36 -> 118 -> -73 -> -133

36 -> 118 -> -73 -> -137

36 -> 118 -> -73 -> -144

36 -> 118 -> -73 -> -145

36 -> 118 -> -73 -> -146

36 -> 118 -> -73 -> -147

36 -> 118 -> -73 -> -150

36 -> 118 -> -73 -> -154

36 -> 118 -> -73 -> -155

36 -> 118 -> -73 -> -156

36 -> 122 -> 3 -> -32

36 -> 122 -> 3 -> -42

36 -> 122 -> 4 -> -101

36 -> 122 -> 6 -> -73

36 -> 122 -> 7 -> -54

36 -> 122 -> 9 -> -6

36 -> 122 -> 14 -> -54

36 -> 122 -> 15 -> -68

36 -> 122 -> 17 -> -32

36 -> 122 -> 19 -> -73

36 -> 122 -> 21 -> -73

36 -> 122 -> 21 -> -101

36 -> 122 -> 21 -> -154

36 -> 122 -> 22 -> -152

36 -> 122 -> 24 -> -49

36 -> 122 -> 25 -> -132

36 -> 122 -> 27 -> -19

36 -> 122 -> 27 -> -20

36 -> 122 -> 27 -> -32

36 -> 122 -> 27 -> -62

36 -> 122 -> 27 -> -68

36 -> 122 -> 27 -> -73

36 -> 122 -> 27 -> -81

36 -> 122 -> 27 -> -98

36 -> 122 -> 27 -> -99

36 -> 122 -> 27 -> -101

36 -> 122 -> 27 -> -107

36 -> 122 -> 27 -> -124

36 -> 122 -> 27 -> -133

36 -> 122 -> 27 -> -145

36 -> 122 -> 28 -> -81

36 -> 122 -> 29 -> -133

36 -> 122 -> 30 -> -1

36 -> 122 -> 30 -> -3

36 -> 122 -> 30 -> -32

36 -> 122 -> 30 -> -39

36 -> 122 -> 30 -> -63

36 -> 122 -> 30 -> -73

36 -> 122 -> 30 -> -81

36 -> 122 -> 30 -> -99

36 -> 122 -> 30 -> -101

36 -> 122 -> 30 -> -106

36 -> 122 -> 30 -> -117

36 -> 122 -> 30 -> -133

36 -> 122 -> 30 -> -137

36 -> 122 -> 30 -> -150

36 -> 122 -> 30 -> -156

36 -> 122 -> 31 -> -1

36 -> 122 -> 32 -> -9

36 -> 122 -> 33 -> -145

36 -> 122 -> 34 -> -32

36 -> 122 -> 34 -> -71

36 -> 122 -> 34 -> -113

36 -> 122 -> 38 -> -55

36 -> 122 -> 38 -> -56

36 -> 122 -> 38 -> -105

36 -> 122 -> 39 -> -101

36 -> 122 -> 40 -> -73

36 -> 122 -> 41 -> -6

36 -> 122 -> 42 -> -133

36 -> 122 -> 44 -> -29

36 -> 122 -> 44 -> -48

36 -> 122 -> 44 -> -81

36 -> 122 -> 44 -> -89

36 -> 122 -> 44 -> -99

36 -> 122 -> 44 -> -114

36 -> 122 -> 45 -> -73

36 -> 122 -> 48 -> -99

36 -> 122 -> 49 -> -15

36 -> 122 -> 51 -> -2

36 -> 122 -> 55 -> -12

36 -> 122 -> 60 -> -64

36 -> 122 -> 61 -> -27

36 -> 122 -> 64 -> -10

36 -> 122 -> 65 -> -4

36 -> 122 -> 66 -> -136

36 -> 122 -> 67 -> -135

36 -> 122 -> 68 -> -73

36 -> 122 -> 69 -> -77

36 -> 122 -> 73 -> -32

36 -> 122 -> 74 -> -112

36 -> 122 -> 75 -> -1

36 -> 122 -> 75 -> -29

36 -> 122 -> 76 -> -81

36 -> 122 -> 77 -> -73

36 -> 122 -> 79 -> -73

36 -> 122 -> 82 -> -30

36 -> 122 -> 82 -> -32

36 -> 122 -> 82 -> -35

36 -> 122 -> 82 -> -101

36 -> 122 -> 84 -> -73

36 -> 122 -> 87 -> -73

36 -> 122 -> 88 -> -83

36 -> 122 -> 89 -> -6

36 -> 122 -> 89 -> -73

36 -> 122 -> 89 -> -81

36 -> 122 -> 89 -> -83

36 -> 122 -> 89 -> -86

36 -> 122 -> 89 -> -99

36 -> 122 -> 89 -> -101

36 -> 122 -> 89 -> -114

36 -> 122 -> 89 -> -155

36 -> 122 -> 91 -> -101

36 -> 122 -> 92 -> -133

36 -> 122 -> 97 -> -15

36 -> 122 -> 99 -> -73

36 -> 122 -> 102 -> -116

36 -> 122 -> 103 -> -78

36 -> 122 -> 105 -> -32

36 -> 122 -> 105 -> -146

36 -> 122 -> 107 -> -73

36 -> 122 -> 107 -> -99

36 -> 122 -> 108 -> -80

36 -> 122 -> 109 -> -20

36 -> 122 -> 109 -> -73

36 -> 122 -> 109 -> -99

36 -> 122 -> 109 -> -101

36 -> 122 -> 110 -> -56

36 -> 122 -> 113 -> -73

36 -> 122 -> 115 -> -17

36 -> 122 -> 115 -> -32

36 -> 122 -> 115 -> -68

36 -> 122 -> 115 -> -73

36 -> 122 -> 115 -> -92

36 -> 122 -> 116 -> -73

36 -> 122 -> 117 -> -73

36 -> 122 -> 117 -> -101

36 -> 122 -> 118 -> -73

36 -> 122 -> 120 -> -133

36 -> 122 -> 123 -> -73

36 -> 122 -> 128 -> -32

36 -> 122 -> 128 -> -95

36 -> 122 -> 129 -> -32

36 -> 122 -> 130 -> -73

36 -> 122 -> 133 -> -101

36 -> 122 -> 134 -> -99

36 -> 122 -> 136 -> -20

36 -> 122 -> 137 -> -18

36 -> 122 -> 137 -> -97

36 -> 122 -> 137 -> -99

36 -> 122 -> 138 -> -20

36 -> 122 -> 138 -> -61

36 -> 122 -> 138 -> -73

36 -> 122 -> 138 -> -98

36 -> 122 -> 138 -> -133

36 -> 122 -> 139 -> -15

36 -> 122 -> 140 -> -99

36 -> 122 -> 140 -> -133

36 -> 122 -> 141 -> -51

36 -> 122 -> 141 -> -100

36 -> 122 -> 141 -> -127

36 -> 122 -> 142 -> -154

36 -> 122 -> 146 -> -20

36 -> 122 -> 148 -> -32

36 -> 122 -> 149 -> -73

36 -> 122 -> 150 -> -91

36 -> 122 -> 151 -> -101

36 -> 122 -> 152 -> -36

36 -> 122 -> 153 -> -2

36 -> 122 -> 157 -> -26

36 -> 122 -> 157 -> -28

36 -> 122 -> 157 -> -32

36 -> 122 -> 157 -> -68

36 -> 122 -> 157 -> -86

36 -> 122 -> 157 -> -99

36 -> 122 -> 157 -> -101

36 -> 122 -> 157 -> -118

36 -> 122 -> 157 -> -119

36 -> 122 -> 157 -> -120

36 -> 122 -> 157 -> -121

36 -> 122 -> 157 -> -122

36 -> 122 -> 157 -> -133

36 -> 122 -> 160 -> -129

36 -> 122 -> 161 -> -78

36 -> 122 -> 162 -> -133

36 -> 122 -> 163 -> -8

36 -> 122 -> 170 -> -138

36 -> 122 -> 171 -> -13

36 -> 122 -> 172 -> -58

36 -> 122 -> 173 -> -154

36 -> 122 -> 176 -> -33

36 -> 122 -> 177 -> -106

36 -> 122 -> 179 -> -22

36 -> 122 -> 179 -> -147

36 -> 122 -> 180 -> -20

36 -> 122 -> 181 -> -84

36 -> 122 -> 182 -> -73

36 -> 122 -> 186 -> -99

36 -> 122 -> 188 -> -24

36 -> 122 -> 189 -> -73

36 -> 122 -> 190 -> -43

36 -> 122 -> 190 -> -73

36 -> 122 -> 190 -> -86

36 -> 122 -> 190 -> -88

36 -> 122 -> 190 -> -144

36 -> 122 -> -56

36 -> 122 -> -56 -> -1

36 -> 122 -> -56 -> -3

36 -> 122 -> -56 -> -19

36 -> 122 -> -56 -> -20

36 -> 122 -> -56 -> -22

36 -> 122 -> -56 -> -29

36 -> 122 -> -56 -> -32

36 -> 122 -> -56 -> -39

36 -> 122 -> -56 -> -42

36 -> 122 -> -56 -> -55

36 -> 122 -> -56 -> -62

36 -> 122 -> -56 -> -63

36 -> 122 -> -56 -> -64

36 -> 122 -> -56 -> -68

36 -> 122 -> -56 -> -71

36 -> 122 -> -56 -> -73

36 -> 122 -> -56 -> -78

36 -> 122 -> -56 -> -81

36 -> 122 -> -56 -> -95

36 -> 122 -> -56 -> -98

36 -> 122 -> -56 -> -99

36 -> 122 -> -56 -> -101

36 -> 122 -> -56 -> -105

36 -> 122 -> -56 -> -106

36 -> 122 -> -56 -> -107

36 -> 122 -> -56 -> -113

36 -> 122 -> -56 -> -117

36 -> 122 -> -56 -> -124

36 -> 122 -> -56 -> -133

36 -> 122 -> -56 -> -137

36 -> 122 -> -56 -> -142

36 -> 122 -> -56 -> -145

36 -> 122 -> -56 -> -146

36 -> 122 -> -56 -> -147

36 -> 122 -> -56 -> -150

36 -> 122 -> -56 -> -154

36 -> 122 -> -56 -> -156

36 -> 123 -> 2 -> -32

36 -> 123 -> 4 -> -101

36 -> 123 -> 6 -> -73

36 -> 123 -> 7 -> -54

36 -> 123 -> 9 -> -6

36 -> 123 -> 12 -> -32

36 -> 123 -> 13 -> -32

36 -> 123 -> 13 -> -73

36 -> 123 -> 14 -> -54

36 -> 123 -> 15 -> -68

36 -> 123 -> 16 -> -5

36 -> 123 -> 17 -> -32

36 -> 123 -> 18 -> -73

36 -> 123 -> 18 -> -89

36 -> 123 -> 19 -> -73

36 -> 123 -> 21 -> -73

36 -> 123 -> 21 -> -101

36 -> 123 -> 21 -> -154

36 -> 123 -> 22 -> -152

36 -> 123 -> 24 -> -49

36 -> 123 -> 25 -> -132

36 -> 123 -> 26 -> -14

36 -> 123 -> 28 -> -81

36 -> 123 -> 29 -> -133

36 -> 123 -> 31 -> -1

36 -> 123 -> 32 -> -9

36 -> 123 -> 33 -> -145

36 -> 123 -> 37 -> -73

36 -> 123 -> 39 -> -101

36 -> 123 -> 40 -> -73

36 -> 123 -> 41 -> -6

36 -> 123 -> 42 -> -133

36 -> 123 -> 43 -> -73

36 -> 123 -> 43 -> -99

36 -> 123 -> 43 -> -101

36 -> 123 -> 45 -> -73

36 -> 123 -> 46 -> -32

36 -> 123 -> 48 -> -99

36 -> 123 -> 49 -> -15

36 -> 123 -> 50 -> -54

36 -> 123 -> 51 -> -2

36 -> 123 -> 52 -> -32

36 -> 123 -> 53 -> -133

36 -> 123 -> 54 -> -53

36 -> 123 -> 55 -> -12

36 -> 123 -> 56 -> -115

36 -> 123 -> 57 -> -103

36 -> 123 -> 58 -> -90

36 -> 123 -> 59 -> -148

36 -> 123 -> 60 -> -64

36 -> 123 -> 61 -> -27

36 -> 123 -> 62 -> -99

36 -> 123 -> 63 -> -32

36 -> 123 -> 64 -> -10

36 -> 123 -> 65 -> -4

36 -> 123 -> 66 -> -136

36 -> 123 -> 67 -> -135

36 -> 123 -> 68 -> -73

36 -> 123 -> 69 -> -77

36 -> 123 -> 70 -> -52

36 -> 123 -> 71 -> -32

36 -> 123 -> 73 -> -32

36 -> 123 -> 74 -> -112

36 -> 123 -> 76 -> -81

36 -> 123 -> 77 -> -73

36 -> 123 -> 78 -> -73

36 -> 123 -> 79 -> -73

36 -> 123 -> 80 -> -32

36 -> 123 -> 81 -> -73

36 -> 123 -> 83 -> -125

36 -> 123 -> 84 -> -73

36 -> 123 -> 85 -> -12

36 -> 123 -> 87 -> -73

36 -> 123 -> 88 -> -83

36 -> 123 -> 90 -> -103

36 -> 123 -> 91 -> -101

36 -> 123 -> 92 -> -133

36 -> 123 -> 93 -> -145

36 -> 123 -> 94 -> -13

36 -> 123 -> 96 -> -73

36 -> 123 -> 97 -> -15

36 -> 123 -> 99 -> -73

36 -> 123 -> 101 -> -64

36 -> 123 -> 102 -> -116

36 -> 123 -> 103 -> -78

36 -> 123 -> 104 -> -2

36 -> 123 -> 106 -> -65

36 -> 123 -> 107 -> -73

36 -> 123 -> 107 -> -99

36 -> 123 -> 108 -> -80

36 -> 123 -> 110 -> -56

36 -> 123 -> 112 -> -54

36 -> 123 -> 113 -> -73

36 -> 123 -> 114 -> -73

36 -> 123 -> 116 -> -73

36 -> 123 -> 117 -> -73

36 -> 123 -> 117 -> -101

36 -> 123 -> 118 -> -73

36 -> 123 -> 120 -> -133

36 -> 123 -> 122 -> -56

36 -> 123 -> 125 -> -104

36 -> 123 -> 126 -> -59

36 -> 123 -> 127 -> -93

36 -> 123 -> 129 -> -32

36 -> 123 -> 130 -> -73

36 -> 123 -> 132 -> -32

36 -> 123 -> 133 -> -101

36 -> 123 -> 134 -> -99

36 -> 123 -> 135 -> -21

36 -> 123 -> 135 -> -73

36 -> 123 -> 135 -> -139

36 -> 123 -> 136 -> -20

36 -> 123 -> 139 -> -15

36 -> 123 -> 142 -> -154

36 -> 123 -> 143 -> -50

36 -> 123 -> 145 -> -125

36 -> 123 -> 146 -> -20

36 -> 123 -> 147 -> -32

36 -> 123 -> 148 -> -32

36 -> 123 -> 149 -> -73

36 -> 123 -> 150 -> -91

36 -> 123 -> 151 -> -101

36 -> 123 -> 152 -> -36

36 -> 123 -> 153 -> -2

36 -> 123 -> 155 -> -73

36 -> 123 -> 158 -> -72

36 -> 123 -> 160 -> -129

36 -> 123 -> 161 -> -78

36 -> 123 -> 162 -> -133

36 -> 123 -> 163 -> -8

36 -> 123 -> 164 -> -134

36 -> 123 -> 165 -> -54

36 -> 123 -> 168 -> -73

36 -> 123 -> 168 -> -102

36 -> 123 -> 169 -> -99

36 -> 123 -> 170 -> -138

36 -> 123 -> 171 -> -13

36 -> 123 -> 172 -> -58

36 -> 123 -> 173 -> -154

36 -> 123 -> 174 -> -114

36 -> 123 -> 176 -> -33

36 -> 123 -> 177 -> -106

36 -> 123 -> 178 -> -69

36 -> 123 -> 180 -> -20

36 -> 123 -> 181 -> -84

36 -> 123 -> 182 -> -73

36 -> 123 -> 186 -> -99

36 -> 123 -> 187 -> -53

36 -> 123 -> 188 -> -24

36 -> 123 -> 189 -> -73

36 -> 123 -> -73

36 -> 123 -> -73 -> -1

36 -> 123 -> -73 -> -3

36 -> 123 -> -73 -> -6

36 -> 123 -> -73 -> -17

36 -> 123 -> -73 -> -19

36 -> 123 -> -73 -> -20

36 -> 123 -> -73 -> -22

36 -> 123 -> -73 -> -26

36 -> 123 -> -73 -> -27

36 -> 123 -> -73 -> -28

36 -> 123 -> -73 -> -29

36 -> 123 -> -73 -> -30

36 -> 123 -> -73 -> -32

36 -> 123 -> -73 -> -35

36 -> 123 -> -73 -> -39

36 -> 123 -> -73 -> -43

36 -> 123 -> -73 -> -48

36 -> 123 -> -73 -> -56

36 -> 123 -> -73 -> -61

36 -> 123 -> -73 -> -62

36 -> 123 -> -73 -> -63

36 -> 123 -> -73 -> -64

36 -> 123 -> -73 -> -68

36 -> 123 -> -73 -> -71

36 -> 123 -> -73 -> -78

36 -> 123 -> -73 -> -81

36 -> 123 -> -73 -> -83

36 -> 123 -> -73 -> -86

36 -> 123 -> -73 -> -88

36 -> 123 -> -73 -> -89

36 -> 123 -> -73 -> -92

36 -> 123 -> -73 -> -95

36 -> 123 -> -73 -> -98

36 -> 123 -> -73 -> -99

36 -> 123 -> -73 -> -101

36 -> 123 -> -73 -> -106

36 -> 123 -> -73 -> -107

36 -> 123 -> -73 -> -113

36 -> 123 -> -73 -> -114

36 -> 123 -> -73 -> -117

36 -> 123 -> -73 -> -118

36 -> 123 -> -73 -> -119

36 -> 123 -> -73 -> -121

36 -> 123 -> -73 -> -122

36 -> 123 -> -73 -> -124

36 -> 123 -> -73 -> -133

36 -> 123 -> -73 -> -137

36 -> 123 -> -73 -> -144

36 -> 123 -> -73 -> -145

36 -> 123 -> -73 -> -146

36 -> 123 -> -73 -> -147

36 -> 123 -> -73 -> -150

36 -> 123 -> -73 -> -154

36 -> 123 -> -73 -> -155

36 -> 123 -> -73 -> -156

36 -> 128 -> 3 -> -32

36 -> 128 -> 3 -> -42

36 -> 128 -> 4 -> -101

36 -> 128 -> 6 -> -73

36 -> 128 -> 17 -> -32

36 -> 128 -> 21 -> -73

36 -> 128 -> 21 -> -101

36 -> 128 -> 21 -> -154

36 -> 128 -> 27 -> -19

36 -> 128 -> 27 -> -20

36 -> 128 -> 27 -> -32

36 -> 128 -> 27 -> -62

36 -> 128 -> 27 -> -68

36 -> 128 -> 27 -> -73

36 -> 128 -> 27 -> -81

36 -> 128 -> 27 -> -98

36 -> 128 -> 27 -> -99

36 -> 128 -> 27 -> -101

36 -> 128 -> 27 -> -107

36 -> 128 -> 27 -> -124

36 -> 128 -> 27 -> -133

36 -> 128 -> 27 -> -145

36 -> 128 -> 29 -> -133

36 -> 128 -> 30 -> -1

36 -> 128 -> 30 -> -3

36 -> 128 -> 30 -> -32

36 -> 128 -> 30 -> -39

36 -> 128 -> 30 -> -63

36 -> 128 -> 30 -> -73

36 -> 128 -> 30 -> -81

36 -> 128 -> 30 -> -99

36 -> 128 -> 30 -> -101

36 -> 128 -> 30 -> -106

36 -> 128 -> 30 -> -117

36 -> 128 -> 30 -> -133

36 -> 128 -> 30 -> -137

36 -> 128 -> 30 -> -150

36 -> 128 -> 30 -> -156

36 -> 128 -> 38 -> -55

36 -> 128 -> 38 -> -56

36 -> 128 -> 38 -> -105

36 -> 128 -> 39 -> -101

36 -> 128 -> 40 -> -73

36 -> 128 -> 43 -> -73

36 -> 128 -> 43 -> -99

36 -> 128 -> 43 -> -101

36 -> 128 -> 44 -> -29

36 -> 128 -> 44 -> -48

36 -> 128 -> 44 -> -81

36 -> 128 -> 44 -> -89

36 -> 128 -> 44 -> -99

36 -> 128 -> 44 -> -114

36 -> 128 -> 60 -> -64

36 -> 128 -> 61 -> -27

36 -> 128 -> 73 -> -32

36 -> 128 -> 75 -> -1

36 -> 128 -> 75 -> -29

36 -> 128 -> 76 -> -81

36 -> 128 -> 77 -> -73

36 -> 128 -> 81 -> -73

36 -> 128 -> 82 -> -30

36 -> 128 -> 82 -> -32

36 -> 128 -> 82 -> -35

36 -> 128 -> 82 -> -101

36 -> 128 -> 83 -> -125

36 -> 128 -> 89 -> -6

36 -> 128 -> 89 -> -73

36 -> 128 -> 89 -> -81

36 -> 128 -> 89 -> -83

36 -> 128 -> 89 -> -86

36 -> 128 -> 89 -> -99

36 -> 128 -> 89 -> -101

36 -> 128 -> 89 -> -114

36 -> 128 -> 89 -> -155

36 -> 128 -> 92 -> -133

36 -> 128 -> 100 -> -32

36 -> 128 -> 100 -> -38

36 -> 128 -> 103 -> -78

36 -> 128 -> 105 -> -32

36 -> 128 -> 105 -> -146

36 -> 128 -> 107 -> -73

36 -> 128 -> 107 -> -99

36 -> 128 -> 114 -> -73

36 -> 128 -> 115 -> -17

36 -> 128 -> 115 -> -32

36 -> 128 -> 115 -> -68

36 -> 128 -> 115 -> -73

36 -> 128 -> 115 -> -92

36 -> 128 -> 117 -> -73

36 -> 128 -> 117 -> -101

36 -> 128 -> 122 -> -56

36 -> 128 -> 129 -> -32

36 -> 128 -> 133 -> -101

36 -> 128 -> 134 -> -99

36 -> 128 -> 136 -> -20

36 -> 128 -> 141 -> -51

36 -> 128 -> 141 -> -100

36 -> 128 -> 141 -> -127

36 -> 128 -> 144 -> -7

36 -> 128 -> 144 -> -110

36 -> 128 -> 144 -> -130

36 -> 128 -> 146 -> -20

36 -> 128 -> 148 -> -32

36 -> 128 -> 151 -> -101

36 -> 128 -> 152 -> -36

36 -> 128 -> 156 -> -32

36 -> 128 -> 156 -> -44

36 -> 128 -> 161 -> -78

36 -> 128 -> 169 -> -99

36 -> 128 -> 173 -> -154

36 -> 128 -> 174 -> -114

36 -> 128 -> 179 -> -22

36 -> 128 -> 179 -> -147

36 -> 128 -> 180 -> -20

36 -> 128 -> 182 -> -73

36 -> 128 -> 186 -> -99

36 -> 128 -> 190 -> -43

36 -> 128 -> 190 -> -73

36 -> 128 -> 190 -> -86

36 -> 128 -> 190 -> -88

36 -> 128 -> 190 -> -144

36 -> 128 -> -32

36 -> 128 -> -32 -> -1

36 -> 128 -> -32 -> -3

36 -> 128 -> -32 -> -14

36 -> 128 -> -32 -> -17

36 -> 128 -> -32 -> -19

36 -> 128 -> -32 -> -20

36 -> 128 -> -32 -> -22

36 -> 128 -> -32 -> -29

36 -> 128 -> -32 -> -30

36 -> 128 -> -32 -> -34

36 -> 128 -> -32 -> -35

36 -> 128 -> -32 -> -37

36 -> 128 -> -32 -> -38

36 -> 128 -> -32 -> -39

36 -> 128 -> -32 -> -43

36 -> 128 -> -32 -> -55

36 -> 128 -> -32 -> -56

36 -> 128 -> -32 -> -62

36 -> 128 -> -32 -> -63

36 -> 128 -> -32 -> -64

36 -> 128 -> -32 -> -68

36 -> 128 -> -32 -> -71

36 -> 128 -> -32 -> -73

36 -> 128 -> -32 -> -78

36 -> 128 -> -32 -> -81

36 -> 128 -> -32 -> -86

36 -> 128 -> -32 -> -92

36 -> 128 -> -32 -> -94

36 -> 128 -> -32 -> -95

36 -> 128 -> -32 -> -98

36 -> 128 -> -32 -> -99

36 -> 128 -> -32 -> -101

36 -> 128 -> -32 -> -103

36 -> 128 -> -32 -> -105

36 -> 128 -> -32 -> -106

36 -> 128 -> -32 -> -107

36 -> 128 -> -32 -> -113

36 -> 128 -> -32 -> -117

36 -> 128 -> -32 -> -124

36 -> 128 -> -32 -> -125

36 -> 128 -> -32 -> -133

36 -> 128 -> -32 -> -137

36 -> 128 -> -32 -> -144

36 -> 128 -> -32 -> -145

36 -> 128 -> -32 -> -146

36 -> 128 -> -32 -> -147

36 -> 128 -> -32 -> -150

36 -> 128 -> -32 -> -154

36 -> 128 -> -32 -> -156

36 -> 128 -> -95 -> -1

36 -> 128 -> -95 -> -3

36 -> 128 -> -95 -> -4

36 -> 128 -> -95 -> -6

36 -> 128 -> -95 -> -7

36 -> 128 -> -95 -> -8

36 -> 128 -> -95 -> -9

36 -> 128 -> -95 -> -10

36 -> 128 -> -95 -> -17

36 -> 128 -> -95 -> -19

36 -> 128 -> -95 -> -20

36 -> 128 -> -95 -> -22

36 -> 128 -> -95 -> -24

36 -> 128 -> -95 -> -27

36 -> 128 -> -95 -> -29

36 -> 128 -> -95 -> -30

36 -> 128 -> -95 -> -31

36 -> 128 -> -95 -> -32

36 -> 128 -> -95 -> -33

36 -> 128 -> -95 -> -35

36 -> 128 -> -95 -> -36

36 -> 128 -> -95 -> -39

36 -> 128 -> -95 -> -43

36 -> 128 -> -95 -> -44

36 -> 128 -> -95 -> -45

36 -> 128 -> -95 -> -46

36 -> 128 -> -95 -> -47

36 -> 128 -> -95 -> -48

36 -> 128 -> -95 -> -49

36 -> 128 -> -95 -> -51

36 -> 128 -> -95 -> -55

36 -> 128 -> -95 -> -56

36 -> 128 -> -95 -> -58

36 -> 128 -> -95 -> -60

36 -> 128 -> -95 -> -62

36 -> 128 -> -95 -> -63

36 -> 128 -> -95 -> -64

36 -> 128 -> -95 -> -66

36 -> 128 -> -95 -> -69

36 -> 128 -> -95 -> -73

36 -> 128 -> -95 -> -77

36 -> 128 -> -95 -> -78

36 -> 128 -> -95 -> -81

36 -> 128 -> -95 -> -83

36 -> 128 -> -95 -> -84

36 -> 128 -> -95 -> -86

36 -> 128 -> -95 -> -89

36 -> 128 -> -95 -> -91

36 -> 128 -> -95 -> -92

36 -> 128 -> -95 -> -96

36 -> 128 -> -95 -> -98

36 -> 128 -> -95 -> -99

36 -> 128 -> -95 -> -100

36 -> 128 -> -95 -> -101

36 -> 128 -> -95 -> -105

36 -> 128 -> -95 -> -106

36 -> 128 -> -95 -> -107

36 -> 128 -> -95 -> -110

36 -> 128 -> -95 -> -114

36 -> 128 -> -95 -> -117

36 -> 128 -> -95 -> -120

36 -> 128 -> -95 -> -124

36 -> 128 -> -95 -> -127

36 -> 128 -> -95 -> -130

36 -> 128 -> -95 -> -131

36 -> 128 -> -95 -> -132

36 -> 128 -> -95 -> -133

36 -> 128 -> -95 -> -135

36 -> 128 -> -95 -> -136

36 -> 128 -> -95 -> -137

36 -> 128 -> -95 -> -141

36 -> 128 -> -95 -> -144

36 -> 128 -> -95 -> -146

36 -> 128 -> -95 -> -147

36 -> 128 -> -95 -> -150

36 -> 128 -> -95 -> -154

36 -> 128 -> -95 -> -155

36 -> 128 -> -95 -> -156

36 -> 129 -> 3 -> -32

36 -> 129 -> 3 -> -42

36 -> 129 -> 4 -> -101

36 -> 129 -> 6 -> -73

36 -> 129 -> 7 -> -54

36 -> 129 -> 9 -> -6

36 -> 129 -> 10 -> -16

36 -> 129 -> 10 -> -20

36 -> 129 -> 10 -> -32

36 -> 129 -> 10 -> -70

36 -> 129 -> 10 -> -74

36 -> 129 -> 10 -> -75

36 -> 129 -> 10 -> -140

36 -> 129 -> 10 -> -143

36 -> 129 -> 11 -> -25

36 -> 129 -> 11 -> -153

36 -> 129 -> 14 -> -54

36 -> 129 -> 15 -> -68

36 -> 129 -> 16 -> -5

36 -> 129 -> 17 -> -32

36 -> 129 -> 19 -> -73

36 -> 129 -> 21 -> -73

36 -> 129 -> 21 -> -101

36 -> 129 -> 21 -> -154

36 -> 129 -> 22 -> -152

36 -> 129 -> 24 -> -49

36 -> 129 -> 25 -> -132

36 -> 129 -> 26 -> -14

36 -> 129 -> 27 -> -19

36 -> 129 -> 27 -> -20

36 -> 129 -> 27 -> -32

36 -> 129 -> 27 -> -62

36 -> 129 -> 27 -> -68

36 -> 129 -> 27 -> -73

36 -> 129 -> 27 -> -81

36 -> 129 -> 27 -> -98

36 -> 129 -> 27 -> -99

36 -> 129 -> 27 -> -101

36 -> 129 -> 27 -> -107

36 -> 129 -> 27 -> -124

36 -> 129 -> 27 -> -133

36 -> 129 -> 27 -> -145

36 -> 129 -> 28 -> -81

36 -> 129 -> 29 -> -133

36 -> 129 -> 30 -> -1

36 -> 129 -> 30 -> -3

36 -> 129 -> 30 -> -32

36 -> 129 -> 30 -> -39

36 -> 129 -> 30 -> -63

36 -> 129 -> 30 -> -73

36 -> 129 -> 30 -> -81

36 -> 129 -> 30 -> -99

36 -> 129 -> 30 -> -101

36 -> 129 -> 30 -> -106

36 -> 129 -> 30 -> -117

36 -> 129 -> 30 -> -133

36 -> 129 -> 30 -> -137

36 -> 129 -> 30 -> -150

36 -> 129 -> 30 -> -156

36 -> 129 -> 32 -> -9

36 -> 129 -> 33 -> -145

36 -> 129 -> 34 -> -32

36 -> 129 -> 34 -> -71

36 -> 129 -> 34 -> -113

36 -> 129 -> 37 -> -73

36 -> 129 -> 38 -> -55

36 -> 129 -> 38 -> -56

36 -> 129 -> 38 -> -105

36 -> 129 -> 39 -> -101

36 -> 129 -> 40 -> -73

36 -> 129 -> 41 -> -6

36 -> 129 -> 42 -> -133

36 -> 129 -> 43 -> -73

36 -> 129 -> 43 -> -99

36 -> 129 -> 43 -> -101

36 -> 129 -> 44 -> -29

36 -> 129 -> 44 -> -48

36 -> 129 -> 44 -> -81

36 -> 129 -> 44 -> -89

36 -> 129 -> 44 -> -99

36 -> 129 -> 44 -> -114

36 -> 129 -> 45 -> -73

36 -> 129 -> 48 -> -99

36 -> 129 -> 49 -> -15

36 -> 129 -> 51 -> -2

36 -> 129 -> 52 -> -32

36 -> 129 -> 53 -> -133

36 -> 129 -> 55 -> -12

36 -> 129 -> 58 -> -90

36 -> 129 -> 60 -> -64

36 -> 129 -> 61 -> -27

36 -> 129 -> 62 -> -99

36 -> 129 -> 63 -> -32

36 -> 129 -> 64 -> -10

36 -> 129 -> 65 -> -4

36 -> 129 -> 66 -> -136

36 -> 129 -> 67 -> -135

36 -> 129 -> 68 -> -73

36 -> 129 -> 69 -> -77

36 -> 129 -> 70 -> -52

36 -> 129 -> 71 -> -32

36 -> 129 -> 72 -> -11

36 -> 129 -> 72 -> -67

36 -> 129 -> 73 -> -32

36 -> 129 -> 74 -> -112

36 -> 129 -> 75 -> -1

36 -> 129 -> 75 -> -29

36 -> 129 -> 76 -> -81

36 -> 129 -> 77 -> -73

36 -> 129 -> 78 -> -73

36 -> 129 -> 79 -> -73

36 -> 129 -> 80 -> -32

36 -> 129 -> 81 -> -73

36 -> 129 -> 82 -> -30

36 -> 129 -> 82 -> -32

36 -> 129 -> 82 -> -35

36 -> 129 -> 82 -> -101

36 -> 129 -> 84 -> -73

36 -> 129 -> 87 -> -73

36 -> 129 -> 88 -> -83

36 -> 129 -> 89 -> -6

36 -> 129 -> 89 -> -73

36 -> 129 -> 89 -> -81

36 -> 129 -> 89 -> -83

36 -> 129 -> 89 -> -86

36 -> 129 -> 89 -> -99

36 -> 129 -> 89 -> -101

36 -> 129 -> 89 -> -114

36 -> 129 -> 89 -> -155

36 -> 129 -> 91 -> -101

36 -> 129 -> 92 -> -133

36 -> 129 -> 94 -> -13

36 -> 129 -> 97 -> -15

36 -> 129 -> 99 -> -73

36 -> 129 -> 100 -> -32

36 -> 129 -> 100 -> -38

36 -> 129 -> 101 -> -64

36 -> 129 -> 102 -> -116

36 -> 129 -> 103 -> -78

36 -> 129 -> 105 -> -32

36 -> 129 -> 105 -> -146

36 -> 129 -> 107 -> -73

36 -> 129 -> 107 -> -99

36 -> 129 -> 108 -> -80

36 -> 129 -> 109 -> -20

36 -> 129 -> 109 -> -73

36 -> 129 -> 109 -> -99

36 -> 129 -> 109 -> -101

36 -> 129 -> 110 -> -56

36 -> 129 -> 113 -> -73

36 -> 129 -> 114 -> -73

36 -> 129 -> 115 -> -17

36 -> 129 -> 115 -> -32

36 -> 129 -> 115 -> -68

36 -> 129 -> 115 -> -73

36 -> 129 -> 115 -> -92

36 -> 129 -> 116 -> -73

36 -> 129 -> 117 -> -73

36 -> 129 -> 117 -> -101

36 -> 129 -> 118 -> -73

36 -> 129 -> 119 -> -80

36 -> 129 -> 119 -> -85

36 -> 129 -> 120 -> -133

36 -> 129 -> 122 -> -56

36 -> 129 -> 123 -> -73

36 -> 129 -> 125 -> -104

36 -> 129 -> 126 -> -59

36 -> 129 -> 127 -> -93

36 -> 129 -> 128 -> -32

36 -> 129 -> 128 -> -95

36 -> 129 -> 130 -> -73

36 -> 129 -> 131 -> -41

36 -> 129 -> 131 -> -99

36 -> 129 -> 131 -> -101

36 -> 129 -> 131 -> -109

36 -> 129 -> 131 -> -111

36 -> 129 -> 131 -> -151

36 -> 129 -> 133 -> -101

36 -> 129 -> 134 -> -99

36 -> 129 -> 136 -> -20

36 -> 129 -> 137 -> -18

36 -> 129 -> 137 -> -97

36 -> 129 -> 137 -> -99

36 -> 129 -> 138 -> -20

36 -> 129 -> 138 -> -61

36 -> 129 -> 138 -> -73

36 -> 129 -> 138 -> -98

36 -> 129 -> 138 -> -133

36 -> 129 -> 139 -> -15

36 -> 129 -> 140 -> -99

36 -> 129 -> 140 -> -133

36 -> 129 -> 141 -> -51

36 -> 129 -> 141 -> -100

36 -> 129 -> 141 -> -127

36 -> 129 -> 142 -> -154

36 -> 129 -> 144 -> -7

36 -> 129 -> 144 -> -110

36 -> 129 -> 144 -> -130

36 -> 129 -> 145 -> -125

36 -> 129 -> 146 -> -20

36 -> 129 -> 148 -> -32

36 -> 129 -> 149 -> -73

36 -> 129 -> 150 -> -91

36 -> 129 -> 151 -> -101

36 -> 129 -> 152 -> -36

36 -> 129 -> 153 -> -2

36 -> 129 -> 156 -> -32

36 -> 129 -> 156 -> -44

36 -> 129 -> 157 -> -26

36 -> 129 -> 157 -> -28

36 -> 129 -> 157 -> -32

36 -> 129 -> 157 -> -68

36 -> 129 -> 157 -> -86

36 -> 129 -> 157 -> -99

36 -> 129 -> 157 -> -101

36 -> 129 -> 157 -> -118

36 -> 129 -> 157 -> -119

36 -> 129 -> 157 -> -120

36 -> 129 -> 157 -> -121

36 -> 129 -> 157 -> -122

36 -> 129 -> 157 -> -133

36 -> 129 -> 158 -> -72

36 -> 129 -> 159 -> -80

36 -> 129 -> 159 -> -128

36 -> 129 -> 159 -> -129

36 -> 129 -> 160 -> -129

36 -> 129 -> 161 -> -78

36 -> 129 -> 162 -> -133

36 -> 129 -> 163 -> -8

36 -> 129 -> 164 -> -134

36 -> 129 -> 169 -> -99

36 -> 129 -> 170 -> -138

36 -> 129 -> 171 -> -13

36 -> 129 -> 172 -> -58

36 -> 129 -> 173 -> -154

36 -> 129 -> 174 -> -114

36 -> 129 -> 175 -> -32

36 -> 129 -> 175 -> -34

36 -> 129 -> 176 -> -33

36 -> 129 -> 177 -> -106

36 -> 129 -> 179 -> -22

36 -> 129 -> 179 -> -147

36 -> 129 -> 180 -> -20

36 -> 129 -> 181 -> -84

36 -> 129 -> 182 -> -73

36 -> 129 -> 184 -> -86

36 -> 129 -> 184 -> -101

36 -> 129 -> 184 -> -133

36 -> 129 -> 185 -> -32

36 -> 129 -> 185 -> -40

36 -> 129 -> 185 -> -79

36 -> 129 -> 185 -> -94

36 -> 129 -> 185 -> -123

36 -> 129 -> 185 -> -133

36 -> 129 -> 186 -> -99

36 -> 129 -> 188 -> -24

36 -> 129 -> 189 -> -73

36 -> 129 -> 190 -> -43

36 -> 129 -> 190 -> -73

36 -> 129 -> 190 -> -86

36 -> 129 -> 190 -> -88

36 -> 129 -> 190 -> -144

36 -> 129 -> -32

36 -> 129 -> -32 -> -1

36 -> 129 -> -32 -> -3

36 -> 129 -> -32 -> -14

36 -> 129 -> -32 -> -17

36 -> 129 -> -32 -> -19

36 -> 129 -> -32 -> -20

36 -> 129 -> -32 -> -22

36 -> 129 -> -32 -> -29

36 -> 129 -> -32 -> -30

36 -> 129 -> -32 -> -34

36 -> 129 -> -32 -> -35

36 -> 129 -> -32 -> -37

36 -> 129 -> -32 -> -38

36 -> 129 -> -32 -> -39

36 -> 129 -> -32 -> -43

36 -> 129 -> -32 -> -55

36 -> 129 -> -32 -> -56

36 -> 129 -> -32 -> -62

36 -> 129 -> -32 -> -63

36 -> 129 -> -32 -> -64

36 -> 129 -> -32 -> -68

36 -> 129 -> -32 -> -71

36 -> 129 -> -32 -> -73

36 -> 129 -> -32 -> -78

36 -> 129 -> -32 -> -81

36 -> 129 -> -32 -> -86

36 -> 129 -> -32 -> -92

36 -> 129 -> -32 -> -94

36 -> 129 -> -32 -> -95

36 -> 129 -> -32 -> -98

36 -> 129 -> -32 -> -99

36 -> 129 -> -32 -> -101

36 -> 129 -> -32 -> -103

36 -> 129 -> -32 -> -105

36 -> 129 -> -32 -> -106

36 -> 129 -> -32 -> -107

36 -> 129 -> -32 -> -113

36 -> 129 -> -32 -> -117

36 -> 129 -> -32 -> -124

36 -> 129 -> -32 -> -125

36 -> 129 -> -32 -> -133

36 -> 129 -> -32 -> -137

36 -> 129 -> -32 -> -144

36 -> 129 -> -32 -> -145

36 -> 129 -> -32 -> -146

36 -> 129 -> -32 -> -147

36 -> 129 -> -32 -> -150

36 -> 129 -> -32 -> -154

36 -> 129 -> -32 -> -156

36 -> 130 -> 2 -> -32

36 -> 130 -> 4 -> -101

36 -> 130 -> 6 -> -73

36 -> 130 -> 7 -> -54

36 -> 130 -> 9 -> -6

36 -> 130 -> 12 -> -32

36 -> 130 -> 13 -> -32

36 -> 130 -> 13 -> -73

36 -> 130 -> 14 -> -54

36 -> 130 -> 15 -> -68

36 -> 130 -> 16 -> -5

36 -> 130 -> 17 -> -32

36 -> 130 -> 18 -> -73

36 -> 130 -> 18 -> -89

36 -> 130 -> 19 -> -73

36 -> 130 -> 21 -> -73

36 -> 130 -> 21 -> -101

36 -> 130 -> 21 -> -154

36 -> 130 -> 22 -> -152

36 -> 130 -> 24 -> -49

36 -> 130 -> 25 -> -132

36 -> 130 -> 26 -> -14

36 -> 130 -> 28 -> -81

36 -> 130 -> 29 -> -133

36 -> 130 -> 31 -> -1

36 -> 130 -> 32 -> -9

36 -> 130 -> 33 -> -145

36 -> 130 -> 37 -> -73

36 -> 130 -> 39 -> -101

36 -> 130 -> 40 -> -73

36 -> 130 -> 41 -> -6

36 -> 130 -> 42 -> -133

36 -> 130 -> 43 -> -73

36 -> 130 -> 43 -> -99

36 -> 130 -> 43 -> -101

36 -> 130 -> 45 -> -73

36 -> 130 -> 46 -> -32

36 -> 130 -> 48 -> -99

36 -> 130 -> 49 -> -15

36 -> 130 -> 50 -> -54

36 -> 130 -> 51 -> -2

36 -> 130 -> 52 -> -32

36 -> 130 -> 53 -> -133

36 -> 130 -> 54 -> -53

36 -> 130 -> 55 -> -12

36 -> 130 -> 56 -> -115

36 -> 130 -> 57 -> -103

36 -> 130 -> 58 -> -90

36 -> 130 -> 59 -> -148

36 -> 130 -> 60 -> -64

36 -> 130 -> 61 -> -27

36 -> 130 -> 62 -> -99

36 -> 130 -> 63 -> -32

36 -> 130 -> 64 -> -10

36 -> 130 -> 65 -> -4

36 -> 130 -> 66 -> -136

36 -> 130 -> 67 -> -135

36 -> 130 -> 68 -> -73

36 -> 130 -> 69 -> -77

36 -> 130 -> 70 -> -52

36 -> 130 -> 71 -> -32

36 -> 130 -> 73 -> -32

36 -> 130 -> 74 -> -112

36 -> 130 -> 76 -> -81

36 -> 130 -> 77 -> -73

36 -> 130 -> 78 -> -73

36 -> 130 -> 79 -> -73

36 -> 130 -> 80 -> -32

36 -> 130 -> 81 -> -73

36 -> 130 -> 83 -> -125

36 -> 130 -> 84 -> -73

36 -> 130 -> 85 -> -12

36 -> 130 -> 87 -> -73

36 -> 130 -> 88 -> -83

36 -> 130 -> 90 -> -103

36 -> 130 -> 91 -> -101

36 -> 130 -> 92 -> -133

36 -> 130 -> 93 -> -145

36 -> 130 -> 94 -> -13

36 -> 130 -> 96 -> -73

36 -> 130 -> 97 -> -15

36 -> 130 -> 99 -> -73

36 -> 130 -> 101 -> -64

36 -> 130 -> 102 -> -116

36 -> 130 -> 103 -> -78

36 -> 130 -> 104 -> -2

36 -> 130 -> 106 -> -65

36 -> 130 -> 107 -> -73

36 -> 130 -> 107 -> -99

36 -> 130 -> 108 -> -80

36 -> 130 -> 110 -> -56

36 -> 130 -> 112 -> -54

36 -> 130 -> 113 -> -73

36 -> 130 -> 114 -> -73

36 -> 130 -> 116 -> -73

36 -> 130 -> 117 -> -73

36 -> 130 -> 117 -> -101

36 -> 130 -> 118 -> -73

36 -> 130 -> 120 -> -133

36 -> 130 -> 122 -> -56

36 -> 130 -> 123 -> -73

36 -> 130 -> 125 -> -104

36 -> 130 -> 126 -> -59

36 -> 130 -> 127 -> -93

36 -> 130 -> 129 -> -32

36 -> 130 -> 132 -> -32

36 -> 130 -> 133 -> -101

36 -> 130 -> 134 -> -99

36 -> 130 -> 135 -> -21

36 -> 130 -> 135 -> -73

36 -> 130 -> 135 -> -139

36 -> 130 -> 136 -> -20

36 -> 130 -> 139 -> -15

36 -> 130 -> 142 -> -154

36 -> 130 -> 143 -> -50

36 -> 130 -> 145 -> -125

36 -> 130 -> 146 -> -20

36 -> 130 -> 147 -> -32

36 -> 130 -> 148 -> -32

36 -> 130 -> 149 -> -73

36 -> 130 -> 150 -> -91

36 -> 130 -> 151 -> -101

36 -> 130 -> 152 -> -36

36 -> 130 -> 153 -> -2

36 -> 130 -> 155 -> -73

36 -> 130 -> 158 -> -72

36 -> 130 -> 160 -> -129

36 -> 130 -> 161 -> -78

36 -> 130 -> 162 -> -133

36 -> 130 -> 163 -> -8

36 -> 130 -> 164 -> -134

36 -> 130 -> 165 -> -54

36 -> 130 -> 168 -> -73

36 -> 130 -> 168 -> -102

36 -> 130 -> 169 -> -99

36 -> 130 -> 170 -> -138

36 -> 130 -> 171 -> -13

36 -> 130 -> 172 -> -58

36 -> 130 -> 173 -> -154

36 -> 130 -> 174 -> -114

36 -> 130 -> 176 -> -33

36 -> 130 -> 177 -> -106

36 -> 130 -> 178 -> -69

36 -> 130 -> 180 -> -20

36 -> 130 -> 181 -> -84

36 -> 130 -> 182 -> -73

36 -> 130 -> 186 -> -99

36 -> 130 -> 187 -> -53

36 -> 130 -> 188 -> -24

36 -> 130 -> 189 -> -73

36 -> 130 -> -73

36 -> 130 -> -73 -> -1

36 -> 130 -> -73 -> -3

36 -> 130 -> -73 -> -6

36 -> 130 -> -73 -> -17

36 -> 130 -> -73 -> -19

36 -> 130 -> -73 -> -20

36 -> 130 -> -73 -> -22

36 -> 130 -> -73 -> -26

36 -> 130 -> -73 -> -27

36 -> 130 -> -73 -> -28

36 -> 130 -> -73 -> -29

36 -> 130 -> -73 -> -30

36 -> 130 -> -73 -> -32

36 -> 130 -> -73 -> -35

36 -> 130 -> -73 -> -39

36 -> 130 -> -73 -> -43

36 -> 130 -> -73 -> -48

36 -> 130 -> -73 -> -56

36 -> 130 -> -73 -> -61

36 -> 130 -> -73 -> -62

36 -> 130 -> -73 -> -63

36 -> 130 -> -73 -> -64

36 -> 130 -> -73 -> -68

36 -> 130 -> -73 -> -71

36 -> 130 -> -73 -> -78

36 -> 130 -> -73 -> -81

36 -> 130 -> -73 -> -83

36 -> 130 -> -73 -> -86

36 -> 130 -> -73 -> -88

36 -> 130 -> -73 -> -89

36 -> 130 -> -73 -> -92

36 -> 130 -> -73 -> -95

36 -> 130 -> -73 -> -98

36 -> 130 -> -73 -> -99

36 -> 130 -> -73 -> -101

36 -> 130 -> -73 -> -106

36 -> 130 -> -73 -> -107

36 -> 130 -> -73 -> -113

36 -> 130 -> -73 -> -114

36 -> 130 -> -73 -> -117

36 -> 130 -> -73 -> -118

36 -> 130 -> -73 -> -119

36 -> 130 -> -73 -> -121

36 -> 130 -> -73 -> -122

36 -> 130 -> -73 -> -124

36 -> 130 -> -73 -> -133

36 -> 130 -> -73 -> -137

36 -> 130 -> -73 -> -144

36 -> 130 -> -73 -> -145

36 -> 130 -> -73 -> -146

36 -> 130 -> -73 -> -147

36 -> 130 -> -73 -> -150

36 -> 130 -> -73 -> -154

36 -> 130 -> -73 -> -155

36 -> 130 -> -73 -> -156

36 -> 136 -> 3 -> -32

36 -> 136 -> 3 -> -42

36 -> 136 -> 4 -> -101

36 -> 136 -> 6 -> -73

36 -> 136 -> 7 -> -54

36 -> 136 -> 8 -> -20

36 -> 136 -> 8 -> -88

36 -> 136 -> 9 -> -6

36 -> 136 -> 14 -> -54

36 -> 136 -> 15 -> -68

36 -> 136 -> 16 -> -5

36 -> 136 -> 19 -> -73

36 -> 136 -> 21 -> -73

36 -> 136 -> 21 -> -101

36 -> 136 -> 21 -> -154

36 -> 136 -> 22 -> -152

36 -> 136 -> 24 -> -49

36 -> 136 -> 25 -> -132

36 -> 136 -> 27 -> -19

36 -> 136 -> 27 -> -20

36 -> 136 -> 27 -> -32

36 -> 136 -> 27 -> -62

36 -> 136 -> 27 -> -68

36 -> 136 -> 27 -> -73

36 -> 136 -> 27 -> -81

36 -> 136 -> 27 -> -98

36 -> 136 -> 27 -> -99

36 -> 136 -> 27 -> -101

36 -> 136 -> 27 -> -107

36 -> 136 -> 27 -> -124

36 -> 136 -> 27 -> -133

36 -> 136 -> 27 -> -145

36 -> 136 -> 28 -> -81

36 -> 136 -> 29 -> -133

36 -> 136 -> 30 -> -1

36 -> 136 -> 30 -> -3

36 -> 136 -> 30 -> -32

36 -> 136 -> 30 -> -39

36 -> 136 -> 30 -> -63

36 -> 136 -> 30 -> -73

36 -> 136 -> 30 -> -81

36 -> 136 -> 30 -> -99

36 -> 136 -> 30 -> -101

36 -> 136 -> 30 -> -106

36 -> 136 -> 30 -> -117

36 -> 136 -> 30 -> -133

36 -> 136 -> 30 -> -137

36 -> 136 -> 30 -> -150

36 -> 136 -> 30 -> -156

36 -> 136 -> 32 -> -9

36 -> 136 -> 33 -> -145

36 -> 136 -> 34 -> -32

36 -> 136 -> 34 -> -71

36 -> 136 -> 34 -> -113

36 -> 136 -> 38 -> -55

36 -> 136 -> 38 -> -56

36 -> 136 -> 38 -> -105

36 -> 136 -> 39 -> -101

36 -> 136 -> 40 -> -73

36 -> 136 -> 41 -> -6

36 -> 136 -> 42 -> -133

36 -> 136 -> 43 -> -73

36 -> 136 -> 43 -> -99

36 -> 136 -> 43 -> -101

36 -> 136 -> 45 -> -73

36 -> 136 -> 48 -> -99

36 -> 136 -> 49 -> -15

36 -> 136 -> 52 -> -32

36 -> 136 -> 55 -> -12

36 -> 136 -> 59 -> -148

36 -> 136 -> 60 -> -64

36 -> 136 -> 61 -> -27

36 -> 136 -> 64 -> -10

36 -> 136 -> 65 -> -4

36 -> 136 -> 66 -> -136

36 -> 136 -> 67 -> -135

36 -> 136 -> 68 -> -73

36 -> 136 -> 69 -> -77

36 -> 136 -> 73 -> -32

36 -> 136 -> 74 -> -112

36 -> 136 -> 75 -> -1

36 -> 136 -> 75 -> -29

36 -> 136 -> 76 -> -81

36 -> 136 -> 77 -> -73

36 -> 136 -> 79 -> -73

36 -> 136 -> 84 -> -73

36 -> 136 -> 87 -> -73

36 -> 136 -> 88 -> -83

36 -> 136 -> 91 -> -101

36 -> 136 -> 92 -> -133

36 -> 136 -> 97 -> -15

36 -> 136 -> 99 -> -73

36 -> 136 -> 103 -> -78

36 -> 136 -> 105 -> -32

36 -> 136 -> 105 -> -146

36 -> 136 -> 107 -> -73

36 -> 136 -> 107 -> -99

36 -> 136 -> 108 -> -80

36 -> 136 -> 109 -> -20

36 -> 136 -> 109 -> -73

36 -> 136 -> 109 -> -99

36 -> 136 -> 109 -> -101

36 -> 136 -> 110 -> -56

36 -> 136 -> 113 -> -73

36 -> 136 -> 115 -> -17

36 -> 136 -> 115 -> -32

36 -> 136 -> 115 -> -68

36 -> 136 -> 115 -> -73

36 -> 136 -> 115 -> -92

36 -> 136 -> 116 -> -73

36 -> 136 -> 117 -> -73

36 -> 136 -> 117 -> -101

36 -> 136 -> 118 -> -73

36 -> 136 -> 120 -> -133

36 -> 136 -> 122 -> -56

36 -> 136 -> 123 -> -73

36 -> 136 -> 128 -> -32

36 -> 136 -> 128 -> -95

36 -> 136 -> 129 -> -32

36 -> 136 -> 130 -> -73

36 -> 136 -> 132 -> -32

36 -> 136 -> 138 -> -20

36 -> 136 -> 138 -> -61

36 -> 136 -> 138 -> -73

36 -> 136 -> 138 -> -98

36 -> 136 -> 138 -> -133

36 -> 136 -> 139 -> -15

36 -> 136 -> 140 -> -99

36 -> 136 -> 140 -> -133

36 -> 136 -> 142 -> -154

36 -> 136 -> 144 -> -7

36 -> 136 -> 144 -> -110

36 -> 136 -> 144 -> -130

36 -> 136 -> 146 -> -20

36 -> 136 -> 148 -> -32

36 -> 136 -> 149 -> -73

36 -> 136 -> 150 -> -91

36 -> 136 -> 152 -> -36

36 -> 136 -> 153 -> -2

36 -> 136 -> 157 -> -26

36 -> 136 -> 157 -> -28

36 -> 136 -> 157 -> -32

36 -> 136 -> 157 -> -68

36 -> 136 -> 157 -> -86

36 -> 136 -> 157 -> -99

36 -> 136 -> 157 -> -101

36 -> 136 -> 157 -> -118

36 -> 136 -> 157 -> -119

36 -> 136 -> 157 -> -120

36 -> 136 -> 157 -> -121

36 -> 136 -> 157 -> -122

36 -> 136 -> 157 -> -133

36 -> 136 -> 160 -> -129

36 -> 136 -> 161 -> -78

36 -> 136 -> 162 -> -133

36 -> 136 -> 163 -> -8

36 -> 136 -> 170 -> -138

36 -> 136 -> 171 -> -13

36 -> 136 -> 172 -> -58

36 -> 136 -> 176 -> -33

36 -> 136 -> 177 -> -106

36 -> 136 -> 179 -> -22

36 -> 136 -> 179 -> -147

36 -> 136 -> 180 -> -20

36 -> 136 -> 181 -> -84

36 -> 136 -> 182 -> -73

36 -> 136 -> 184 -> -86

36 -> 136 -> 184 -> -101

36 -> 136 -> 184 -> -133

36 -> 136 -> 186 -> -99

36 -> 136 -> 188 -> -24

36 -> 136 -> 189 -> -73

36 -> 136 -> -20

36 -> 136 -> -20 -> -1

36 -> 136 -> -20 -> -3

36 -> 136 -> -20 -> -6

36 -> 136 -> -20 -> -16

36 -> 136 -> -20 -> -17

36 -> 136 -> -20 -> -19

36 -> 136 -> -20 -> -22

36 -> 136 -> -20 -> -26

36 -> 136 -> -20 -> -27

36 -> 136 -> -20 -> -28

36 -> 136 -> -20 -> -29

36 -> 136 -> -20 -> -32

36 -> 136 -> -20 -> -39

36 -> 136 -> -20 -> -43

36 -> 136 -> -20 -> -48

36 -> 136 -> -20 -> -56

36 -> 136 -> -20 -> -61

36 -> 136 -> -20 -> -62

36 -> 136 -> -20 -> -63

36 -> 136 -> -20 -> -64

36 -> 136 -> -20 -> -68

36 -> 136 -> -20 -> -70

36 -> 136 -> -20 -> -73

36 -> 136 -> -20 -> -74

36 -> 136 -> -20 -> -75

36 -> 136 -> -20 -> -78

36 -> 136 -> -20 -> -81

36 -> 136 -> -20 -> -82

36 -> 136 -> -20 -> -83

36 -> 136 -> -20 -> -86

36 -> 136 -> -20 -> -88

36 -> 136 -> -20 -> -89

36 -> 136 -> -20 -> -92

36 -> 136 -> -20 -> -95

36 -> 136 -> -20 -> -98

36 -> 136 -> -20 -> -99

36 -> 136 -> -20 -> -101

36 -> 136 -> -20 -> -106

36 -> 136 -> -20 -> -107

36 -> 136 -> -20 -> -114

36 -> 136 -> -20 -> -117

36 -> 136 -> -20 -> -118

36 -> 136 -> -20 -> -119

36 -> 136 -> -20 -> -121

36 -> 136 -> -20 -> -122

36 -> 136 -> -20 -> -124

36 -> 136 -> -20 -> -125

36 -> 136 -> -20 -> -133

36 -> 136 -> -20 -> -137

36 -> 136 -> -20 -> -140

36 -> 136 -> -20 -> -143

36 -> 136 -> -20 -> -144

36 -> 136 -> -20 -> -145

36 -> 136 -> -20 -> -146

36 -> 136 -> -20 -> -147

36 -> 136 -> -20 -> -150

36 -> 136 -> -20 -> -152

36 -> 136 -> -20 -> -154

36 -> 136 -> -20 -> -155

36 -> 136 -> -20 -> -156

36 -> 138 -> 3 -> -32

36 -> 138 -> 3 -> -42

36 -> 138 -> 6 -> -73

36 -> 138 -> 27 -> -19

36 -> 138 -> 27 -> -20

36 -> 138 -> 27 -> -32

36 -> 138 -> 27 -> -62

36 -> 138 -> 27 -> -68

36 -> 138 -> 27 -> -73

36 -> 138 -> 27 -> -81

36 -> 138 -> 27 -> -98

36 -> 138 -> 27 -> -99

36 -> 138 -> 27 -> -101

36 -> 138 -> 27 -> -107

36 -> 138 -> 27 -> -124

36 -> 138 -> 27 -> -133

36 -> 138 -> 27 -> -145

36 -> 138 -> 30 -> -1

36 -> 138 -> 30 -> -3

36 -> 138 -> 30 -> -32

36 -> 138 -> 30 -> -39

36 -> 138 -> 30 -> -63

36 -> 138 -> 30 -> -73

36 -> 138 -> 30 -> -81

36 -> 138 -> 30 -> -99

36 -> 138 -> 30 -> -101

36 -> 138 -> 30 -> -106

36 -> 138 -> 30 -> -117

36 -> 138 -> 30 -> -133

36 -> 138 -> 30 -> -137

36 -> 138 -> 30 -> -150

36 -> 138 -> 30 -> -156

36 -> 138 -> 37 -> -73

36 -> 138 -> 43 -> -73

36 -> 138 -> 43 -> -99

36 -> 138 -> 43 -> -101

36 -> 138 -> 61 -> -27

36 -> 138 -> 62 -> -99

36 -> 138 -> 63 -> -32

36 -> 138 -> 75 -> -1

36 -> 138 -> 75 -> -29

36 -> 138 -> 76 -> -81

36 -> 138 -> 77 -> -73

36 -> 138 -> 78 -> -73

36 -> 138 -> 92 -> -133

36 -> 138 -> 99 -> -73

36 -> 138 -> 103 -> -78

36 -> 138 -> 105 -> -32

36 -> 138 -> 105 -> -146

36 -> 138 -> 107 -> -73

36 -> 138 -> 107 -> -99

36 -> 138 -> 115 -> -17

36 -> 138 -> 115 -> -32

36 -> 138 -> 115 -> -68

36 -> 138 -> 115 -> -73

36 -> 138 -> 115 -> -92

36 -> 138 -> 122 -> -56

36 -> 138 -> 129 -> -32

36 -> 138 -> 136 -> -20

36 -> 138 -> 140 -> -99

36 -> 138 -> 140 -> -133

36 -> 138 -> 144 -> -7

36 -> 138 -> 144 -> -110

36 -> 138 -> 144 -> -130

36 -> 138 -> 157 -> -26

36 -> 138 -> 157 -> -28

36 -> 138 -> 157 -> -32

36 -> 138 -> 157 -> -68

36 -> 138 -> 157 -> -86

36 -> 138 -> 157 -> -99

36 -> 138 -> 157 -> -101

36 -> 138 -> 157 -> -118

36 -> 138 -> 157 -> -119

36 -> 138 -> 157 -> -120

36 -> 138 -> 157 -> -121

36 -> 138 -> 157 -> -122

36 -> 138 -> 157 -> -133

36 -> 138 -> 169 -> -99

36 -> 138 -> 179 -> -22

36 -> 138 -> 179 -> -147

36 -> 138 -> 182 -> -73

36 -> 138 -> 186 -> -99

36 -> 138 -> -20

36 -> 138 -> -20 -> -1

36 -> 138 -> -20 -> -3

36 -> 138 -> -20 -> -6

36 -> 138 -> -20 -> -16

36 -> 138 -> -20 -> -17

36 -> 138 -> -20 -> -19

36 -> 138 -> -20 -> -22

36 -> 138 -> -20 -> -26

36 -> 138 -> -20 -> -27

36 -> 138 -> -20 -> -28

36 -> 138 -> -20 -> -29

36 -> 138 -> -20 -> -32

36 -> 138 -> -20 -> -39

36 -> 138 -> -20 -> -43

36 -> 138 -> -20 -> -48

36 -> 138 -> -20 -> -56

36 -> 138 -> -20 -> -61

36 -> 138 -> -20 -> -62

36 -> 138 -> -20 -> -63

36 -> 138 -> -20 -> -64

36 -> 138 -> -20 -> -68

36 -> 138 -> -20 -> -70

36 -> 138 -> -20 -> -73

36 -> 138 -> -20 -> -74

36 -> 138 -> -20 -> -75

36 -> 138 -> -20 -> -78

36 -> 138 -> -20 -> -81

36 -> 138 -> -20 -> -82

36 -> 138 -> -20 -> -83

36 -> 138 -> -20 -> -86

36 -> 138 -> -20 -> -88

36 -> 138 -> -20 -> -89

36 -> 138 -> -20 -> -92

36 -> 138 -> -20 -> -95

36 -> 138 -> -20 -> -98

36 -> 138 -> -20 -> -99

36 -> 138 -> -20 -> -101

36 -> 138 -> -20 -> -106

36 -> 138 -> -20 -> -107

36 -> 138 -> -20 -> -114

36 -> 138 -> -20 -> -117

36 -> 138 -> -20 -> -118

36 -> 138 -> -20 -> -119

36 -> 138 -> -20 -> -121

36 -> 138 -> -20 -> -122

36 -> 138 -> -20 -> -124

36 -> 138 -> -20 -> -125

36 -> 138 -> -20 -> -133

36 -> 138 -> -20 -> -137

36 -> 138 -> -20 -> -140

36 -> 138 -> -20 -> -143

36 -> 138 -> -20 -> -144

36 -> 138 -> -20 -> -145

36 -> 138 -> -20 -> -146

36 -> 138 -> -20 -> -147

36 -> 138 -> -20 -> -150

36 -> 138 -> -20 -> -152

36 -> 138 -> -20 -> -154

36 -> 138 -> -20 -> -155

36 -> 138 -> -20 -> -156

36 -> 138 -> -61 -> -3

36 -> 138 -> -61 -> -4

36 -> 138 -> -61 -> -7

36 -> 138 -> -61 -> -8

36 -> 138 -> -61 -> -9

36 -> 138 -> -61 -> -10

36 -> 138 -> -61 -> -17

36 -> 138 -> -61 -> -19

36 -> 138 -> -61 -> -20

36 -> 138 -> -61 -> -22

36 -> 138 -> -61 -> -24

36 -> 138 -> -61 -> -26

36 -> 138 -> -61 -> -27

36 -> 138 -> -61 -> -28

36 -> 138 -> -61 -> -29

36 -> 138 -> -61 -> -31

36 -> 138 -> -61 -> -33

36 -> 138 -> -61 -> -39

36 -> 138 -> -61 -> -43

36 -> 138 -> -61 -> -44

36 -> 138 -> -61 -> -45

36 -> 138 -> -61 -> -46

36 -> 138 -> -61 -> -47

36 -> 138 -> -61 -> -49

36 -> 138 -> -61 -> -58

36 -> 138 -> -61 -> -60

36 -> 138 -> -61 -> -62

36 -> 138 -> -61 -> -63

36 -> 138 -> -61 -> -66

36 -> 138 -> -61 -> -68

36 -> 138 -> -61 -> -69

36 -> 138 -> -61 -> -73

36 -> 138 -> -61 -> -77

36 -> 138 -> -61 -> -78

36 -> 138 -> -61 -> -81

36 -> 138 -> -61 -> -84

36 -> 138 -> -61 -> -86

36 -> 138 -> -61 -> -88

36 -> 138 -> -61 -> -91

36 -> 138 -> -61 -> -92

36 -> 138 -> -61 -> -96

36 -> 138 -> -61 -> -98

36 -> 138 -> -61 -> -99

36 -> 138 -> -61 -> -101

36 -> 138 -> -61 -> -107

36 -> 138 -> -61 -> -110

36 -> 138 -> -61 -> -117

36 -> 138 -> -61 -> -118

36 -> 138 -> -61 -> -119

36 -> 138 -> -61 -> -120

36 -> 138 -> -61 -> -121

36 -> 138 -> -61 -> -122

36 -> 138 -> -61 -> -124

36 -> 138 -> -61 -> -130

36 -> 138 -> -61 -> -131

36 -> 138 -> -61 -> -132

36 -> 138 -> -61 -> -133

36 -> 138 -> -61 -> -135

36 -> 138 -> -61 -> -136

36 -> 138 -> -61 -> -137

36 -> 138 -> -61 -> -141

36 -> 138 -> -61 -> -144

36 -> 138 -> -61 -> -145

36 -> 138 -> -61 -> -147

36 -> 138 -> -61 -> -150

36 -> 138 -> -61 -> -156

36 -> 138 -> -73

36 -> 138 -> -73 -> -1

36 -> 138 -> -73 -> -3

36 -> 138 -> -73 -> -6

36 -> 138 -> -73 -> -17

36 -> 138 -> -73 -> -19

36 -> 138 -> -73 -> -20

36 -> 138 -> -73 -> -22

36 -> 138 -> -73 -> -26

36 -> 138 -> -73 -> -27

36 -> 138 -> -73 -> -28

36 -> 138 -> -73 -> -29

36 -> 138 -> -73 -> -30

36 -> 138 -> -73 -> -32

36 -> 138 -> -73 -> -35

36 -> 138 -> -73 -> -39

36 -> 138 -> -73 -> -43

36 -> 138 -> -73 -> -48

36 -> 138 -> -73 -> -56

36 -> 138 -> -73 -> -61

36 -> 138 -> -73 -> -62

36 -> 138 -> -73 -> -63

36 -> 138 -> -73 -> -64

36 -> 138 -> -73 -> -68

36 -> 138 -> -73 -> -71

36 -> 138 -> -73 -> -78

36 -> 138 -> -73 -> -81

36 -> 138 -> -73 -> -83

36 -> 138 -> -73 -> -86

36 -> 138 -> -73 -> -88

36 -> 138 -> -73 -> -89

36 -> 138 -> -73 -> -92

36 -> 138 -> -73 -> -95

36 -> 138 -> -73 -> -98

36 -> 138 -> -73 -> -99

36 -> 138 -> -73 -> -101

36 -> 138 -> -73 -> -106

36 -> 138 -> -73 -> -107

36 -> 138 -> -73 -> -113

36 -> 138 -> -73 -> -114

36 -> 138 -> -73 -> -117

36 -> 138 -> -73 -> -118

36 -> 138 -> -73 -> -119

36 -> 138 -> -73 -> -121

36 -> 138 -> -73 -> -122

36 -> 138 -> -73 -> -124

36 -> 138 -> -73 -> -133

36 -> 138 -> -73 -> -137

36 -> 138 -> -73 -> -144

36 -> 138 -> -73 -> -145

36 -> 138 -> -73 -> -146

36 -> 138 -> -73 -> -147

36 -> 138 -> -73 -> -150

36 -> 138 -> -73 -> -154

36 -> 138 -> -73 -> -155

36 -> 138 -> -73 -> -156

36 -> 138 -> -98

36 -> 138 -> -98 -> -1

36 -> 138 -> -98 -> -3

36 -> 138 -> -98 -> -4

36 -> 138 -> -98 -> -6

36 -> 138 -> -98 -> -7

36 -> 138 -> -98 -> -8

36 -> 138 -> -98 -> -9

36 -> 138 -> -98 -> -10

36 -> 138 -> -98 -> -17

36 -> 138 -> -98 -> -19

36 -> 138 -> -98 -> -20

36 -> 138 -> -98 -> -22

36 -> 138 -> -98 -> -24

36 -> 138 -> -98 -> -26

36 -> 138 -> -98 -> -27

36 -> 138 -> -98 -> -28

36 -> 138 -> -98 -> -29

36 -> 138 -> -98 -> -30

36 -> 138 -> -98 -> -31

36 -> 138 -> -98 -> -32

36 -> 138 -> -98 -> -33

36 -> 138 -> -98 -> -35

36 -> 138 -> -98 -> -36

36 -> 138 -> -98 -> -39

36 -> 138 -> -98 -> -42

36 -> 138 -> -98 -> -43

36 -> 138 -> -98 -> -44

36 -> 138 -> -98 -> -45

36 -> 138 -> -98 -> -46

36 -> 138 -> -98 -> -47

36 -> 138 -> -98 -> -48

36 -> 138 -> -98 -> -49

36 -> 138 -> -98 -> -51

36 -> 138 -> -98 -> -55

36 -> 138 -> -98 -> -56

36 -> 138 -> -98 -> -58

36 -> 138 -> -98 -> -60

36 -> 138 -> -98 -> -61

36 -> 138 -> -98 -> -62

36 -> 138 -> -98 -> -63

36 -> 138 -> -98 -> -64

36 -> 138 -> -98 -> -66

36 -> 138 -> -98 -> -68

36 -> 138 -> -98 -> -69

36 -> 138 -> -98 -> -71

36 -> 138 -> -98 -> -73

36 -> 138 -> -98 -> -77

36 -> 138 -> -98 -> -78

36 -> 138 -> -98 -> -81

36 -> 138 -> -98 -> -83

36 -> 138 -> -98 -> -84

36 -> 138 -> -98 -> -86

36 -> 138 -> -98 -> -88

36 -> 138 -> -98 -> -89

36 -> 138 -> -98 -> -91

36 -> 138 -> -98 -> -92

36 -> 138 -> -98 -> -95

36 -> 138 -> -98 -> -96

36 -> 138 -> -98 -> -99

36 -> 138 -> -98 -> -100

36 -> 138 -> -98 -> -101

36 -> 138 -> -98 -> -105

36 -> 138 -> -98 -> -106

36 -> 138 -> -98 -> -107

36 -> 138 -> -98 -> -110

36 -> 138 -> -98 -> -113

36 -> 138 -> -98 -> -114

36 -> 138 -> -98 -> -117

36 -> 138 -> -98 -> -118

36 -> 138 -> -98 -> -119

36 -> 138 -> -98 -> -120

36 -> 138 -> -98 -> -121

36 -> 138 -> -98 -> -122

36 -> 138 -> -98 -> -124

36 -> 138 -> -98 -> -127

36 -> 138 -> -98 -> -130

36 -> 138 -> -98 -> -131

36 -> 138 -> -98 -> -132

36 -> 138 -> -98 -> -133

36 -> 138 -> -98 -> -135

36 -> 138 -> -98 -> -136

36 -> 138 -> -98 -> -137

36 -> 138 -> -98 -> -141

36 -> 138 -> -98 -> -144

36 -> 138 -> -98 -> -145

36 -> 138 -> -98 -> -146

36 -> 138 -> -98 -> -147

36 -> 138 -> -98 -> -150

36 -> 138 -> -98 -> -154

36 -> 138 -> -98 -> -155

36 -> 138 -> -98 -> -156

36 -> 138 -> -133

36 -> 138 -> -133 -> -1

36 -> 138 -> -133 -> -3

36 -> 138 -> -133 -> -19

36 -> 138 -> -133 -> -20

36 -> 138 -> -133 -> -22

36 -> 138 -> -133 -> -26

36 -> 138 -> -133 -> -27

36 -> 138 -> -133 -> -28

36 -> 138 -> -133 -> -29

36 -> 138 -> -133 -> -32

36 -> 138 -> -133 -> -36

36 -> 138 -> -133 -> -39

36 -> 138 -> -133 -> -40

36 -> 138 -> -133 -> -48

36 -> 138 -> -133 -> -56

36 -> 138 -> -133 -> -61

36 -> 138 -> -133 -> -62

36 -> 138 -> -133 -> -63

36 -> 138 -> -133 -> -64

36 -> 138 -> -133 -> -68

36 -> 138 -> -133 -> -73

36 -> 138 -> -133 -> -78

36 -> 138 -> -133 -> -79

36 -> 138 -> -133 -> -81

36 -> 138 -> -133 -> -86

36 -> 138 -> -133 -> -94

36 -> 138 -> -133 -> -95

36 -> 138 -> -133 -> -98

36 -> 138 -> -133 -> -99

36 -> 138 -> -133 -> -101

36 -> 138 -> -133 -> -106

36 -> 138 -> -133 -> -107

36 -> 138 -> -133 -> -114

36 -> 138 -> -133 -> -117

36 -> 138 -> -133 -> -118

36 -> 138 -> -133 -> -119

36 -> 138 -> -133 -> -121

36 -> 138 -> -133 -> -122

36 -> 138 -> -133 -> -123

36 -> 138 -> -133 -> -124

36 -> 138 -> -133 -> -125

36 -> 138 -> -133 -> -137

36 -> 138 -> -133 -> -145

36 -> 138 -> -133 -> -146

36 -> 138 -> -133 -> -147

36 -> 138 -> -133 -> -150

36 -> 138 -> -133 -> -154

36 -> 138 -> -133 -> -156

36 -> 140 -> 6 -> -73

36 -> 140 -> 27 -> -19

36 -> 140 -> 27 -> -20

36 -> 140 -> 27 -> -32

36 -> 140 -> 27 -> -62

36 -> 140 -> 27 -> -68

36 -> 140 -> 27 -> -73

36 -> 140 -> 27 -> -81

36 -> 140 -> 27 -> -98

36 -> 140 -> 27 -> -99

36 -> 140 -> 27 -> -101

36 -> 140 -> 27 -> -107

36 -> 140 -> 27 -> -124

36 -> 140 -> 27 -> -133

36 -> 140 -> 27 -> -145

36 -> 140 -> 30 -> -1

36 -> 140 -> 30 -> -3

36 -> 140 -> 30 -> -32

36 -> 140 -> 30 -> -39

36 -> 140 -> 30 -> -63

36 -> 140 -> 30 -> -73

36 -> 140 -> 30 -> -81

36 -> 140 -> 30 -> -99

36 -> 140 -> 30 -> -101

36 -> 140 -> 30 -> -106

36 -> 140 -> 30 -> -117

36 -> 140 -> 30 -> -133

36 -> 140 -> 30 -> -137

36 -> 140 -> 30 -> -150

36 -> 140 -> 30 -> -156

36 -> 140 -> 37 -> -73

36 -> 140 -> 42 -> -133

36 -> 140 -> 43 -> -73

36 -> 140 -> 43 -> -99

36 -> 140 -> 43 -> -101

36 -> 140 -> 48 -> -99

36 -> 140 -> 52 -> -32

36 -> 140 -> 61 -> -27

36 -> 140 -> 75 -> -1

36 -> 140 -> 75 -> -29

36 -> 140 -> 76 -> -81

36 -> 140 -> 77 -> -73

36 -> 140 -> 78 -> -73

36 -> 140 -> 92 -> -133

36 -> 140 -> 105 -> -32

36 -> 140 -> 105 -> -146

36 -> 140 -> 107 -> -73

36 -> 140 -> 107 -> -99

36 -> 140 -> 115 -> -17

36 -> 140 -> 115 -> -32

36 -> 140 -> 115 -> -68

36 -> 140 -> 115 -> -73

36 -> 140 -> 115 -> -92

36 -> 140 -> 120 -> -133

36 -> 140 -> 122 -> -56

36 -> 140 -> 129 -> -32

36 -> 140 -> 136 -> -20

36 -> 140 -> 138 -> -20

36 -> 140 -> 138 -> -61

36 -> 140 -> 138 -> -73

36 -> 140 -> 138 -> -98

36 -> 140 -> 138 -> -133

36 -> 140 -> 157 -> -26

36 -> 140 -> 157 -> -28

36 -> 140 -> 157 -> -32

36 -> 140 -> 157 -> -68

36 -> 140 -> 157 -> -86

36 -> 140 -> 157 -> -99

36 -> 140 -> 157 -> -101

36 -> 140 -> 157 -> -118

36 -> 140 -> 157 -> -119

36 -> 140 -> 157 -> -120

36 -> 140 -> 157 -> -121

36 -> 140 -> 157 -> -122

36 -> 140 -> 157 -> -133

36 -> 140 -> 162 -> -133

36 -> 140 -> 169 -> -99

36 -> 140 -> 179 -> -22

36 -> 140 -> 179 -> -147

36 -> 140 -> 184 -> -86

36 -> 140 -> 184 -> -101

36 -> 140 -> 184 -> -133

36 -> 140 -> 186 -> -99

36 -> 140 -> -99

36 -> 140 -> -99 -> -1

36 -> 140 -> -99 -> -3

36 -> 140 -> -99 -> -6

36 -> 140 -> -99 -> -17

36 -> 140 -> -99 -> -18

36 -> 140 -> -99 -> -19

36 -> 140 -> -99 -> -20

36 -> 140 -> -99 -> -22

36 -> 140 -> -99 -> -26

36 -> 140 -> -99 -> -27

36 -> 140 -> -99 -> -28

36 -> 140 -> -99 -> -29

36 -> 140 -> -99 -> -30

36 -> 140 -> -99 -> -32

36 -> 140 -> -99 -> -35

36 -> 140 -> -99 -> -36

36 -> 140 -> -99 -> -39

36 -> 140 -> -99 -> -41

36 -> 140 -> -99 -> -43

36 -> 140 -> -99 -> -48

36 -> 140 -> -99 -> -51

36 -> 140 -> -99 -> -56

36 -> 140 -> -99 -> -61

36 -> 140 -> -99 -> -62

36 -> 140 -> -99 -> -63

36 -> 140 -> -99 -> -64

36 -> 140 -> -99 -> -68

36 -> 140 -> -99 -> -73

36 -> 140 -> -99 -> -78

36 -> 140 -> -99 -> -81

36 -> 140 -> -99 -> -83

36 -> 140 -> -99 -> -86

36 -> 140 -> -99 -> -88

36 -> 140 -> -99 -> -89

36 -> 140 -> -99 -> -92

36 -> 140 -> -99 -> -95

36 -> 140 -> -99 -> -97

36 -> 140 -> -99 -> -98

36 -> 140 -> -99 -> -100

36 -> 140 -> -99 -> -101

36 -> 140 -> -99 -> -106

36 -> 140 -> -99 -> -107

36 -> 140 -> -99 -> -109

36 -> 140 -> -99 -> -111

36 -> 140 -> -99 -> -114

36 -> 140 -> -99 -> -117

36 -> 140 -> -99 -> -118

36 -> 140 -> -99 -> -119

36 -> 140 -> -99 -> -121

36 -> 140 -> -99 -> -122

36 -> 140 -> -99 -> -124

36 -> 140 -> -99 -> -127

36 -> 140 -> -99 -> -133

36 -> 140 -> -99 -> -137

36 -> 140 -> -99 -> -144

36 -> 140 -> -99 -> -145

36 -> 140 -> -99 -> -146

36 -> 140 -> -99 -> -147

36 -> 140 -> -99 -> -150

36 -> 140 -> -99 -> -151

36 -> 140 -> -99 -> -154

36 -> 140 -> -99 -> -155

36 -> 140 -> -99 -> -156

36 -> 140 -> -133

36 -> 140 -> -133 -> -1

36 -> 140 -> -133 -> -3

36 -> 140 -> -133 -> -19

36 -> 140 -> -133 -> -20

36 -> 140 -> -133 -> -22

36 -> 140 -> -133 -> -26

36 -> 140 -> -133 -> -27

36 -> 140 -> -133 -> -28

36 -> 140 -> -133 -> -29

36 -> 140 -> -133 -> -32

36 -> 140 -> -133 -> -36

36 -> 140 -> -133 -> -39

36 -> 140 -> -133 -> -40

36 -> 140 -> -133 -> -48

36 -> 140 -> -133 -> -56

36 -> 140 -> -133 -> -61

36 -> 140 -> -133 -> -62

36 -> 140 -> -133 -> -63

36 -> 140 -> -133 -> -64

36 -> 140 -> -133 -> -68

36 -> 140 -> -133 -> -73

36 -> 140 -> -133 -> -78

36 -> 140 -> -133 -> -79

36 -> 140 -> -133 -> -81

36 -> 140 -> -133 -> -86

36 -> 140 -> -133 -> -94

36 -> 140 -> -133 -> -95

36 -> 140 -> -133 -> -98

36 -> 140 -> -133 -> -99

36 -> 140 -> -133 -> -101

36 -> 140 -> -133 -> -106

36 -> 140 -> -133 -> -107

36 -> 140 -> -133 -> -114

36 -> 140 -> -133 -> -117

36 -> 140 -> -133 -> -118

36 -> 140 -> -133 -> -119

36 -> 140 -> -133 -> -121

36 -> 140 -> -133 -> -122

36 -> 140 -> -133 -> -123

36 -> 140 -> -133 -> -124

36 -> 140 -> -133 -> -125

36 -> 140 -> -133 -> -137

36 -> 140 -> -133 -> -145

36 -> 140 -> -133 -> -146

36 -> 140 -> -133 -> -147

36 -> 140 -> -133 -> -150

36 -> 140 -> -133 -> -154

36 -> 140 -> -133 -> -156

36 -> 149 -> 2 -> -32

36 -> 149 -> 4 -> -101

36 -> 149 -> 6 -> -73

36 -> 149 -> 7 -> -54

36 -> 149 -> 9 -> -6

36 -> 149 -> 12 -> -32

36 -> 149 -> 13 -> -32

36 -> 149 -> 13 -> -73

36 -> 149 -> 14 -> -54

36 -> 149 -> 15 -> -68

36 -> 149 -> 16 -> -5

36 -> 149 -> 17 -> -32

36 -> 149 -> 18 -> -73

36 -> 149 -> 18 -> -89

36 -> 149 -> 19 -> -73

36 -> 149 -> 21 -> -73

36 -> 149 -> 21 -> -101

36 -> 149 -> 21 -> -154

36 -> 149 -> 22 -> -152

36 -> 149 -> 24 -> -49

36 -> 149 -> 25 -> -132

36 -> 149 -> 26 -> -14

36 -> 149 -> 28 -> -81

36 -> 149 -> 29 -> -133

36 -> 149 -> 31 -> -1

36 -> 149 -> 32 -> -9

36 -> 149 -> 33 -> -145

36 -> 149 -> 37 -> -73

36 -> 149 -> 39 -> -101

36 -> 149 -> 40 -> -73

36 -> 149 -> 41 -> -6

36 -> 149 -> 42 -> -133

36 -> 149 -> 43 -> -73

36 -> 149 -> 43 -> -99

36 -> 149 -> 43 -> -101

36 -> 149 -> 45 -> -73

36 -> 149 -> 46 -> -32

36 -> 149 -> 48 -> -99

36 -> 149 -> 49 -> -15

36 -> 149 -> 50 -> -54

36 -> 149 -> 51 -> -2

36 -> 149 -> 52 -> -32

36 -> 149 -> 53 -> -133

36 -> 149 -> 54 -> -53

36 -> 149 -> 55 -> -12

36 -> 149 -> 56 -> -115

36 -> 149 -> 57 -> -103

36 -> 149 -> 58 -> -90

36 -> 149 -> 59 -> -148

36 -> 149 -> 60 -> -64

36 -> 149 -> 61 -> -27

36 -> 149 -> 62 -> -99

36 -> 149 -> 63 -> -32

36 -> 149 -> 64 -> -10

36 -> 149 -> 65 -> -4

36 -> 149 -> 66 -> -136

36 -> 149 -> 67 -> -135

36 -> 149 -> 68 -> -73

36 -> 149 -> 69 -> -77

36 -> 149 -> 70 -> -52

36 -> 149 -> 71 -> -32

36 -> 149 -> 73 -> -32

36 -> 149 -> 74 -> -112

36 -> 149 -> 76 -> -81

36 -> 149 -> 77 -> -73

36 -> 149 -> 78 -> -73

36 -> 149 -> 79 -> -73

36 -> 149 -> 80 -> -32

36 -> 149 -> 81 -> -73

36 -> 149 -> 83 -> -125

36 -> 149 -> 84 -> -73

36 -> 149 -> 85 -> -12

36 -> 149 -> 87 -> -73

36 -> 149 -> 88 -> -83

36 -> 149 -> 90 -> -103

36 -> 149 -> 91 -> -101

36 -> 149 -> 92 -> -133

36 -> 149 -> 93 -> -145

36 -> 149 -> 94 -> -13

36 -> 149 -> 96 -> -73

36 -> 149 -> 97 -> -15

36 -> 149 -> 99 -> -73

36 -> 149 -> 101 -> -64

36 -> 149 -> 102 -> -116

36 -> 149 -> 103 -> -78

36 -> 149 -> 104 -> -2

36 -> 149 -> 106 -> -65

36 -> 149 -> 107 -> -73

36 -> 149 -> 107 -> -99

36 -> 149 -> 108 -> -80

36 -> 149 -> 110 -> -56

36 -> 149 -> 112 -> -54

36 -> 149 -> 113 -> -73

36 -> 149 -> 114 -> -73

36 -> 149 -> 116 -> -73

36 -> 149 -> 117 -> -73

36 -> 149 -> 117 -> -101

36 -> 149 -> 118 -> -73

36 -> 149 -> 120 -> -133

36 -> 149 -> 122 -> -56

36 -> 149 -> 123 -> -73

36 -> 149 -> 125 -> -104

36 -> 149 -> 126 -> -59

36 -> 149 -> 127 -> -93

36 -> 149 -> 129 -> -32

36 -> 149 -> 130 -> -73

36 -> 149 -> 132 -> -32

36 -> 149 -> 133 -> -101

36 -> 149 -> 134 -> -99

36 -> 149 -> 135 -> -21

36 -> 149 -> 135 -> -73

36 -> 149 -> 135 -> -139

36 -> 149 -> 136 -> -20

36 -> 149 -> 139 -> -15

36 -> 149 -> 142 -> -154

36 -> 149 -> 143 -> -50

36 -> 149 -> 145 -> -125

36 -> 149 -> 146 -> -20

36 -> 149 -> 147 -> -32

36 -> 149 -> 148 -> -32

36 -> 149 -> 150 -> -91

36 -> 149 -> 151 -> -101

36 -> 149 -> 152 -> -36

36 -> 149 -> 153 -> -2

36 -> 149 -> 155 -> -73

36 -> 149 -> 158 -> -72

36 -> 149 -> 160 -> -129

36 -> 149 -> 161 -> -78

36 -> 149 -> 162 -> -133

36 -> 149 -> 163 -> -8

36 -> 149 -> 164 -> -134

36 -> 149 -> 165 -> -54

36 -> 149 -> 168 -> -73

36 -> 149 -> 168 -> -102

36 -> 149 -> 169 -> -99

36 -> 149 -> 170 -> -138

36 -> 149 -> 171 -> -13

36 -> 149 -> 172 -> -58

36 -> 149 -> 173 -> -154

36 -> 149 -> 174 -> -114

36 -> 149 -> 176 -> -33

36 -> 149 -> 177 -> -106

36 -> 149 -> 178 -> -69

36 -> 149 -> 180 -> -20

36 -> 149 -> 181 -> -84

36 -> 149 -> 182 -> -73

36 -> 149 -> 186 -> -99

36 -> 149 -> 187 -> -53

36 -> 149 -> 188 -> -24

36 -> 149 -> 189 -> -73

36 -> 149 -> -73

36 -> 149 -> -73 -> -1

36 -> 149 -> -73 -> -3

36 -> 149 -> -73 -> -6

36 -> 149 -> -73 -> -17

36 -> 149 -> -73 -> -19

36 -> 149 -> -73 -> -20

36 -> 149 -> -73 -> -22

36 -> 149 -> -73 -> -26

36 -> 149 -> -73 -> -27

36 -> 149 -> -73 -> -28

36 -> 149 -> -73 -> -29

36 -> 149 -> -73 -> -30

36 -> 149 -> -73 -> -32

36 -> 149 -> -73 -> -35

36 -> 149 -> -73 -> -39

36 -> 149 -> -73 -> -43

36 -> 149 -> -73 -> -48

36 -> 149 -> -73 -> -56

36 -> 149 -> -73 -> -61

36 -> 149 -> -73 -> -62

36 -> 149 -> -73 -> -63

36 -> 149 -> -73 -> -64

36 -> 149 -> -73 -> -68

36 -> 149 -> -73 -> -71

36 -> 149 -> -73 -> -78

36 -> 149 -> -73 -> -81

36 -> 149 -> -73 -> -83

36 -> 149 -> -73 -> -86

36 -> 149 -> -73 -> -88

36 -> 149 -> -73 -> -89

36 -> 149 -> -73 -> -92

36 -> 149 -> -73 -> -95

36 -> 149 -> -73 -> -98

36 -> 149 -> -73 -> -99

36 -> 149 -> -73 -> -101

36 -> 149 -> -73 -> -106

36 -> 149 -> -73 -> -107

36 -> 149 -> -73 -> -113

36 -> 149 -> -73 -> -114

36 -> 149 -> -73 -> -117

36 -> 149 -> -73 -> -118

36 -> 149 -> -73 -> -119

36 -> 149 -> -73 -> -121

36 -> 149 -> -73 -> -122

36 -> 149 -> -73 -> -124

36 -> 149 -> -73 -> -133

36 -> 149 -> -73 -> -137

36 -> 149 -> -73 -> -144

36 -> 149 -> -73 -> -145

36 -> 149 -> -73 -> -146

36 -> 149 -> -73 -> -147

36 -> 149 -> -73 -> -150

36 -> 149 -> -73 -> -154

36 -> 149 -> -73 -> -155

36 -> 149 -> -73 -> -156

36 -> 157 -> 6 -> -73

36 -> 157 -> 27 -> -19

36 -> 157 -> 27 -> -20

36 -> 157 -> 27 -> -32

36 -> 157 -> 27 -> -62

36 -> 157 -> 27 -> -68

36 -> 157 -> 27 -> -73

36 -> 157 -> 27 -> -81

36 -> 157 -> 27 -> -98

36 -> 157 -> 27 -> -99

36 -> 157 -> 27 -> -101

36 -> 157 -> 27 -> -107

36 -> 157 -> 27 -> -124

36 -> 157 -> 27 -> -133

36 -> 157 -> 27 -> -145

36 -> 157 -> 30 -> -1

36 -> 157 -> 30 -> -3

36 -> 157 -> 30 -> -32

36 -> 157 -> 30 -> -39

36 -> 157 -> 30 -> -63

36 -> 157 -> 30 -> -73

36 -> 157 -> 30 -> -81

36 -> 157 -> 30 -> -99

36 -> 157 -> 30 -> -101

36 -> 157 -> 30 -> -106

36 -> 157 -> 30 -> -117

36 -> 157 -> 30 -> -133

36 -> 157 -> 30 -> -137

36 -> 157 -> 30 -> -150

36 -> 157 -> 30 -> -156

36 -> 157 -> 37 -> -73

36 -> 157 -> 43 -> -73

36 -> 157 -> 43 -> -99

36 -> 157 -> 43 -> -101

36 -> 157 -> 61 -> -27

36 -> 157 -> 62 -> -99

36 -> 157 -> 75 -> -1

36 -> 157 -> 75 -> -29

36 -> 157 -> 76 -> -81

36 -> 157 -> 77 -> -73

36 -> 157 -> 78 -> -73

36 -> 157 -> 92 -> -133

36 -> 157 -> 99 -> -73

36 -> 157 -> 103 -> -78

36 -> 157 -> 105 -> -32

36 -> 157 -> 105 -> -146

36 -> 157 -> 107 -> -73

36 -> 157 -> 107 -> -99

36 -> 157 -> 122 -> -56

36 -> 157 -> 129 -> -32

36 -> 157 -> 136 -> -20

36 -> 157 -> 138 -> -20

36 -> 157 -> 138 -> -61

36 -> 157 -> 138 -> -73

36 -> 157 -> 138 -> -98

36 -> 157 -> 138 -> -133

36 -> 157 -> 140 -> -99

36 -> 157 -> 140 -> -133

36 -> 157 -> 179 -> -22

36 -> 157 -> 179 -> -147

36 -> 157 -> 186 -> -99

36 -> 157 -> -26 -> -1

36 -> 157 -> -26 -> -3

36 -> 157 -> -26 -> -4

36 -> 157 -> -26 -> -8

36 -> 157 -> -26 -> -9

36 -> 157 -> -26 -> -10

36 -> 157 -> -26 -> -19

36 -> 157 -> -26 -> -20

36 -> 157 -> -26 -> -22

36 -> 157 -> -26 -> -24

36 -> 157 -> -26 -> -27

36 -> 157 -> -26 -> -28

36 -> 157 -> -26 -> -29

36 -> 157 -> -26 -> -31

36 -> 157 -> -26 -> -33

36 -> 157 -> -26 -> -39

36 -> 157 -> -26 -> -44

36 -> 157 -> -26 -> -45

36 -> 157 -> -26 -> -46

36 -> 157 -> -26 -> -47

36 -> 157 -> -26 -> -49

36 -> 157 -> -26 -> -58

36 -> 157 -> -26 -> -60

36 -> 157 -> -26 -> -61

36 -> 157 -> -26 -> -62

36 -> 157 -> -26 -> -63

36 -> 157 -> -26 -> -66

36 -> 157 -> -26 -> -68

36 -> 157 -> -26 -> -69

36 -> 157 -> -26 -> -73

36 -> 157 -> -26 -> -77

36 -> 157 -> -26 -> -78

36 -> 157 -> -26 -> -81

36 -> 157 -> -26 -> -84

36 -> 157 -> -26 -> -86

36 -> 157 -> -26 -> -88

36 -> 157 -> -26 -> -91

36 -> 157 -> -26 -> -96

36 -> 157 -> -26 -> -98

36 -> 157 -> -26 -> -99

36 -> 157 -> -26 -> -101

36 -> 157 -> -26 -> -107

36 -> 157 -> -26 -> -117

36 -> 157 -> -26 -> -118

36 -> 157 -> -26 -> -119

36 -> 157 -> -26 -> -120

36 -> 157 -> -26 -> -121

36 -> 157 -> -26 -> -122

36 -> 157 -> -26 -> -124

36 -> 157 -> -26 -> -131

36 -> 157 -> -26 -> -132

36 -> 157 -> -26 -> -133

36 -> 157 -> -26 -> -135

36 -> 157 -> -26 -> -136

36 -> 157 -> -26 -> -137

36 -> 157 -> -26 -> -141

36 -> 157 -> -26 -> -145

36 -> 157 -> -26 -> -147

36 -> 157 -> -26 -> -150

36 -> 157 -> -26 -> -156

36 -> 157 -> -28 -> -1

36 -> 157 -> -28 -> -3

36 -> 157 -> -28 -> -4

36 -> 157 -> -28 -> -8

36 -> 157 -> -28 -> -9

36 -> 157 -> -28 -> -10

36 -> 157 -> -28 -> -19

36 -> 157 -> -28 -> -20

36 -> 157 -> -28 -> -22

36 -> 157 -> -28 -> -24

36 -> 157 -> -28 -> -26

36 -> 157 -> -28 -> -27

36 -> 157 -> -28 -> -29

36 -> 157 -> -28 -> -31

36 -> 157 -> -28 -> -33

36 -> 157 -> -28 -> -39

36 -> 157 -> -28 -> -44

36 -> 157 -> -28 -> -45

36 -> 157 -> -28 -> -46

36 -> 157 -> -28 -> -47

36 -> 157 -> -28 -> -49

36 -> 157 -> -28 -> -58

36 -> 157 -> -28 -> -60

36 -> 157 -> -28 -> -61

36 -> 157 -> -28 -> -62

36 -> 157 -> -28 -> -63

36 -> 157 -> -28 -> -66

36 -> 157 -> -28 -> -68

36 -> 157 -> -28 -> -69

36 -> 157 -> -28 -> -73

36 -> 157 -> -28 -> -77

36 -> 157 -> -28 -> -78

36 -> 157 -> -28 -> -81

36 -> 157 -> -28 -> -84

36 -> 157 -> -28 -> -86

36 -> 157 -> -28 -> -88

36 -> 157 -> -28 -> -91

36 -> 157 -> -28 -> -96

36 -> 157 -> -28 -> -98

36 -> 157 -> -28 -> -99

36 -> 157 -> -28 -> -101

36 -> 157 -> -28 -> -107

36 -> 157 -> -28 -> -117

36 -> 157 -> -28 -> -118

36 -> 157 -> -28 -> -119

36 -> 157 -> -28 -> -120

36 -> 157 -> -28 -> -121

36 -> 157 -> -28 -> -122

36 -> 157 -> -28 -> -124

36 -> 157 -> -28 -> -131

36 -> 157 -> -28 -> -132

36 -> 157 -> -28 -> -133

36 -> 157 -> -28 -> -135

36 -> 157 -> -28 -> -136

36 -> 157 -> -28 -> -137

36 -> 157 -> -28 -> -141

36 -> 157 -> -28 -> -145

36 -> 157 -> -28 -> -147

36 -> 157 -> -28 -> -150

36 -> 157 -> -28 -> -156

36 -> 157 -> -32

36 -> 157 -> -32 -> -1

36 -> 157 -> -32 -> -3

36 -> 157 -> -32 -> -14

36 -> 157 -> -32 -> -17

36 -> 157 -> -32 -> -19

36 -> 157 -> -32 -> -20

36 -> 157 -> -32 -> -22

36 -> 157 -> -32 -> -29

36 -> 157 -> -32 -> -30

36 -> 157 -> -32 -> -34

36 -> 157 -> -32 -> -35

36 -> 157 -> -32 -> -37

36 -> 157 -> -32 -> -38

36 -> 157 -> -32 -> -39

36 -> 157 -> -32 -> -43

36 -> 157 -> -32 -> -55

36 -> 157 -> -32 -> -56

36 -> 157 -> -32 -> -62

36 -> 157 -> -32 -> -63

36 -> 157 -> -32 -> -64

36 -> 157 -> -32 -> -68

36 -> 157 -> -32 -> -71

36 -> 157 -> -32 -> -73

36 -> 157 -> -32 -> -78

36 -> 157 -> -32 -> -81

36 -> 157 -> -32 -> -86

36 -> 157 -> -32 -> -92

36 -> 157 -> -32 -> -94

36 -> 157 -> -32 -> -95

36 -> 157 -> -32 -> -98

36 -> 157 -> -32 -> -99

36 -> 157 -> -32 -> -101

36 -> 157 -> -32 -> -103

36 -> 157 -> -32 -> -105

36 -> 157 -> -32 -> -106

36 -> 157 -> -32 -> -107

36 -> 157 -> -32 -> -113

36 -> 157 -> -32 -> -117

36 -> 157 -> -32 -> -124

36 -> 157 -> -32 -> -125

36 -> 157 -> -32 -> -133

36 -> 157 -> -32 -> -137

36 -> 157 -> -32 -> -144

36 -> 157 -> -32 -> -145

36 -> 157 -> -32 -> -146

36 -> 157 -> -32 -> -147

36 -> 157 -> -32 -> -150

36 -> 157 -> -32 -> -154

36 -> 157 -> -32 -> -156

36 -> 157 -> -68

36 -> 157 -> -68 -> -1

36 -> 157 -> -68 -> -2

36 -> 157 -> -68 -> -3

36 -> 157 -> -68 -> -17

36 -> 157 -> -68 -> -19

36 -> 157 -> -68 -> -20

36 -> 157 -> -68 -> -22

36 -> 157 -> -68 -> -26

36 -> 157 -> -68 -> -27

36 -> 157 -> -68 -> -28

36 -> 157 -> -68 -> -29

36 -> 157 -> -68 -> -32

36 -> 157 -> -68 -> -39

36 -> 157 -> -68 -> -41

36 -> 157 -> -68 -> -43

36 -> 157 -> -68 -> -56

36 -> 157 -> -68 -> -61

36 -> 157 -> -68 -> -62

36 -> 157 -> -68 -> -63

36 -> 157 -> -68 -> -71

36 -> 157 -> -68 -> -73

36 -> 157 -> -68 -> -78

36 -> 157 -> -68 -> -80

36 -> 157 -> -68 -> -81

36 -> 157 -> -68 -> -86

36 -> 157 -> -68 -> -88

36 -> 157 -> -68 -> -92

36 -> 157 -> -68 -> -98

36 -> 157 -> -68 -> -99

36 -> 157 -> -68 -> -101

36 -> 157 -> -68 -> -106

36 -> 157 -> -68 -> -107

36 -> 157 -> -68 -> -109

36 -> 157 -> -68 -> -111

36 -> 157 -> -68 -> -113

36 -> 157 -> -68 -> -117

36 -> 157 -> -68 -> -118

36 -> 157 -> -68 -> -119

36 -> 157 -> -68 -> -121

36 -> 157 -> -68 -> -122

36 -> 157 -> -68 -> -124

36 -> 157 -> -68 -> -128

36 -> 157 -> -68 -> -129

36 -> 157 -> -68 -> -133

36 -> 157 -> -68 -> -137

36 -> 157 -> -68 -> -142

36 -> 157 -> -68 -> -144

36 -> 157 -> -68 -> -145

36 -> 157 -> -68 -> -146

36 -> 157 -> -68 -> -147

36 -> 157 -> -68 -> -150

36 -> 157 -> -68 -> -151

36 -> 157 -> -68 -> -156

36 -> 157 -> -86

36 -> 157 -> -86 -> -1

36 -> 157 -> -86 -> -3

36 -> 157 -> -86 -> -6

36 -> 157 -> -86 -> -7

36 -> 157 -> -86 -> -19

36 -> 157 -> -86 -> -20

36 -> 157 -> -86 -> -22

36 -> 157 -> -86 -> -26

36 -> 157 -> -86 -> -27

36 -> 157 -> -86 -> -28

36 -> 157 -> -86 -> -29

36 -> 157 -> -86 -> -32

36 -> 157 -> -86 -> -39

36 -> 157 -> -86 -> -40

36 -> 157 -> -86 -> -48

36 -> 157 -> -86 -> -51

36 -> 157 -> -86 -> -61

36 -> 157 -> -86 -> -62

36 -> 157 -> -86 -> -63

36 -> 157 -> -86 -> -64

36 -> 157 -> -86 -> -68

36 -> 157 -> -86 -> -73

36 -> 157 -> -86 -> -78

36 -> 157 -> -86 -> -79

36 -> 157 -> -86 -> -81

36 -> 157 -> -86 -> -83

36 -> 157 -> -86 -> -88

36 -> 157 -> -86 -> -89

36 -> 157 -> -86 -> -94

36 -> 157 -> -86 -> -95

36 -> 157 -> -86 -> -98

36 -> 157 -> -86 -> -99

36 -> 157 -> -86 -> -100

36 -> 157 -> -86 -> -101

36 -> 157 -> -86 -> -106

36 -> 157 -> -86 -> -107

36 -> 157 -> -86 -> -110

36 -> 157 -> -86 -> -114

36 -> 157 -> -86 -> -117

36 -> 157 -> -86 -> -118

36 -> 157 -> -86 -> -119

36 -> 157 -> -86 -> -120

36 -> 157 -> -86 -> -121

36 -> 157 -> -86 -> -122

36 -> 157 -> -86 -> -123

36 -> 157 -> -86 -> -124

36 -> 157 -> -86 -> -125

36 -> 157 -> -86 -> -127

36 -> 157 -> -86 -> -130

36 -> 157 -> -86 -> -133

36 -> 157 -> -86 -> -137

36 -> 157 -> -86 -> -145

36 -> 157 -> -86 -> -147

36 -> 157 -> -86 -> -150

36 -> 157 -> -86 -> -154

36 -> 157 -> -86 -> -155

36 -> 157 -> -86 -> -156

36 -> 157 -> -99

36 -> 157 -> -99 -> -1

36 -> 157 -> -99 -> -3

36 -> 157 -> -99 -> -6

36 -> 157 -> -99 -> -17

36 -> 157 -> -99 -> -18

36 -> 157 -> -99 -> -19

36 -> 157 -> -99 -> -20

36 -> 157 -> -99 -> -22

36 -> 157 -> -99 -> -26

36 -> 157 -> -99 -> -27

36 -> 157 -> -99 -> -28

36 -> 157 -> -99 -> -29

36 -> 157 -> -99 -> -30

36 -> 157 -> -99 -> -32

36 -> 157 -> -99 -> -35

36 -> 157 -> -99 -> -36

36 -> 157 -> -99 -> -39

36 -> 157 -> -99 -> -41

36 -> 157 -> -99 -> -43

36 -> 157 -> -99 -> -48

36 -> 157 -> -99 -> -51

36 -> 157 -> -99 -> -56

36 -> 157 -> -99 -> -61

36 -> 157 -> -99 -> -62

36 -> 157 -> -99 -> -63

36 -> 157 -> -99 -> -64

36 -> 157 -> -99 -> -68

36 -> 157 -> -99 -> -73

36 -> 157 -> -99 -> -78

36 -> 157 -> -99 -> -81

36 -> 157 -> -99 -> -83

36 -> 157 -> -99 -> -86

36 -> 157 -> -99 -> -88

36 -> 157 -> -99 -> -89

36 -> 157 -> -99 -> -92

36 -> 157 -> -99 -> -95

36 -> 157 -> -99 -> -97

36 -> 157 -> -99 -> -98

36 -> 157 -> -99 -> -100

36 -> 157 -> -99 -> -101

36 -> 157 -> -99 -> -106

36 -> 157 -> -99 -> -107

36 -> 157 -> -99 -> -109

36 -> 157 -> -99 -> -111

36 -> 157 -> -99 -> -114

36 -> 157 -> -99 -> -117

36 -> 157 -> -99 -> -118

36 -> 157 -> -99 -> -119

36 -> 157 -> -99 -> -121

36 -> 157 -> -99 -> -122

36 -> 157 -> -99 -> -124

36 -> 157 -> -99 -> -127

36 -> 157 -> -99 -> -133

36 -> 157 -> -99 -> -137

36 -> 157 -> -99 -> -144

36 -> 157 -> -99 -> -145

36 -> 157 -> -99 -> -146

36 -> 157 -> -99 -> -147

36 -> 157 -> -99 -> -150

36 -> 157 -> -99 -> -151

36 -> 157 -> -99 -> -154

36 -> 157 -> -99 -> -155

36 -> 157 -> -99 -> -156

36 -> 157 -> -101

36 -> 157 -> -101 -> -1

36 -> 157 -> -101 -> -3

36 -> 157 -> -101 -> -6

36 -> 157 -> -101 -> -17

36 -> 157 -> -101 -> -19

36 -> 157 -> -101 -> -20

36 -> 157 -> -101 -> -22

36 -> 157 -> -101 -> -26

36 -> 157 -> -101 -> -28

36 -> 157 -> -101 -> -29

36 -> 157 -> -101 -> -30

36 -> 157 -> -101 -> -32

36 -> 157 -> -101 -> -35

36 -> 157 -> -101 -> -36

36 -> 157 -> -101 -> -39

36 -> 157 -> -101 -> -41

36 -> 157 -> -101 -> -43

36 -> 157 -> -101 -> -48

36 -> 157 -> -101 -> -51

36 -> 157 -> -101 -> -56

36 -> 157 -> -101 -> -61

36 -> 157 -> -101 -> -62

36 -> 157 -> -101 -> -63

36 -> 157 -> -101 -> -64

36 -> 157 -> -101 -> -68

36 -> 157 -> -101 -> -73

36 -> 157 -> -101 -> -74

36 -> 157 -> -101 -> -75

36 -> 157 -> -101 -> -78

36 -> 157 -> -101 -> -81

36 -> 157 -> -101 -> -83

36 -> 157 -> -101 -> -86

36 -> 157 -> -101 -> -89

36 -> 157 -> -101 -> -92

36 -> 157 -> -101 -> -95

36 -> 157 -> -101 -> -98

36 -> 157 -> -101 -> -99

36 -> 157 -> -101 -> -100

36 -> 157 -> -101 -> -106

36 -> 157 -> -101 -> -107

36 -> 157 -> -101 -> -109

36 -> 157 -> -101 -> -111

36 -> 157 -> -101 -> -114

36 -> 157 -> -101 -> -117

36 -> 157 -> -101 -> -118

36 -> 157 -> -101 -> -119

36 -> 157 -> -101 -> -121

36 -> 157 -> -101 -> -122

36 -> 157 -> -101 -> -124

36 -> 157 -> -101 -> -125

36 -> 157 -> -101 -> -127

36 -> 157 -> -101 -> -133

36 -> 157 -> -101 -> -137

36 -> 157 -> -101 -> -144

36 -> 157 -> -101 -> -145

36 -> 157 -> -101 -> -146

36 -> 157 -> -101 -> -147

36 -> 157 -> -101 -> -150

36 -> 157 -> -101 -> -151

36 -> 157 -> -101 -> -154

36 -> 157 -> -101 -> -155

36 -> 157 -> -101 -> -156

36 -> 157 -> -118 -> -1

36 -> 157 -> -118 -> -3

36 -> 157 -> -118 -> -4

36 -> 157 -> -118 -> -8

36 -> 157 -> -118 -> -9

36 -> 157 -> -118 -> -10

36 -> 157 -> -118 -> -19

36 -> 157 -> -118 -> -20

36 -> 157 -> -118 -> -22

36 -> 157 -> -118 -> -24

36 -> 157 -> -118 -> -26

36 -> 157 -> -118 -> -27

36 -> 157 -> -118 -> -28

36 -> 157 -> -118 -> -29

36 -> 157 -> -118 -> -31

36 -> 157 -> -118 -> -33

36 -> 157 -> -118 -> -39

36 -> 157 -> -118 -> -44

36 -> 157 -> -118 -> -45

36 -> 157 -> -118 -> -46

36 -> 157 -> -118 -> -47

36 -> 157 -> -118 -> -49

36 -> 157 -> -118 -> -58

36 -> 157 -> -118 -> -60

36 -> 157 -> -118 -> -61

36 -> 157 -> -118 -> -62

36 -> 157 -> -118 -> -63

36 -> 157 -> -118 -> -66

36 -> 157 -> -118 -> -68

36 -> 157 -> -118 -> -69

36 -> 157 -> -118 -> -73

36 -> 157 -> -118 -> -77

36 -> 157 -> -118 -> -78

36 -> 157 -> -118 -> -81

36 -> 157 -> -118 -> -84

36 -> 157 -> -118 -> -86

36 -> 157 -> -118 -> -88

36 -> 157 -> -118 -> -91

36 -> 157 -> -118 -> -96

36 -> 157 -> -118 -> -98

36 -> 157 -> -118 -> -99

36 -> 157 -> -118 -> -101

36 -> 157 -> -118 -> -107

36 -> 157 -> -118 -> -117

36 -> 157 -> -118 -> -119

36 -> 157 -> -118 -> -120

36 -> 157 -> -118 -> -121

36 -> 157 -> -118 -> -122

36 -> 157 -> -118 -> -124

36 -> 157 -> -118 -> -131

36 -> 157 -> -118 -> -132

36 -> 157 -> -118 -> -133

36 -> 157 -> -118 -> -135

36 -> 157 -> -118 -> -136

36 -> 157 -> -118 -> -137

36 -> 157 -> -118 -> -141

36 -> 157 -> -118 -> -145

36 -> 157 -> -118 -> -147

36 -> 157 -> -118 -> -150

36 -> 157 -> -118 -> -156

36 -> 157 -> -119 -> -1

36 -> 157 -> -119 -> -3

36 -> 157 -> -119 -> -4

36 -> 157 -> -119 -> -8

36 -> 157 -> -119 -> -9

36 -> 157 -> -119 -> -10

36 -> 157 -> -119 -> -19

36 -> 157 -> -119 -> -20

36 -> 157 -> -119 -> -22

36 -> 157 -> -119 -> -24

36 -> 157 -> -119 -> -26

36 -> 157 -> -119 -> -27

36 -> 157 -> -119 -> -28

36 -> 157 -> -119 -> -29

36 -> 157 -> -119 -> -31

36 -> 157 -> -119 -> -33

36 -> 157 -> -119 -> -39

36 -> 157 -> -119 -> -44

36 -> 157 -> -119 -> -45

36 -> 157 -> -119 -> -46

36 -> 157 -> -119 -> -47

36 -> 157 -> -119 -> -49

36 -> 157 -> -119 -> -58

36 -> 157 -> -119 -> -60

36 -> 157 -> -119 -> -61

36 -> 157 -> -119 -> -62

36 -> 157 -> -119 -> -63

36 -> 157 -> -119 -> -66

36 -> 157 -> -119 -> -68

36 -> 157 -> -119 -> -69

36 -> 157 -> -119 -> -73

36 -> 157 -> -119 -> -77

36 -> 157 -> -119 -> -78

36 -> 157 -> -119 -> -81

36 -> 157 -> -119 -> -84

36 -> 157 -> -119 -> -86

36 -> 157 -> -119 -> -88

36 -> 157 -> -119 -> -91

36 -> 157 -> -119 -> -96

36 -> 157 -> -119 -> -98

36 -> 157 -> -119 -> -99

36 -> 157 -> -119 -> -101

36 -> 157 -> -119 -> -107

36 -> 157 -> -119 -> -117

36 -> 157 -> -119 -> -118

36 -> 157 -> -119 -> -120

36 -> 157 -> -119 -> -121

36 -> 157 -> -119 -> -122

36 -> 157 -> -119 -> -124

36 -> 157 -> -119 -> -131

36 -> 157 -> -119 -> -132

36 -> 157 -> -119 -> -133

36 -> 157 -> -119 -> -135

36 -> 157 -> -119 -> -136

36 -> 157 -> -119 -> -137

36 -> 157 -> -119 -> -141

36 -> 157 -> -119 -> -145

36 -> 157 -> -119 -> -147

36 -> 157 -> -119 -> -150

36 -> 157 -> -119 -> -156

36 -> 157 -> -120 -> -3

36 -> 157 -> -120 -> -4

36 -> 157 -> -120 -> -5

36 -> 157 -> -120 -> -7

36 -> 157 -> -120 -> -8

36 -> 157 -> -120 -> -9

36 -> 157 -> -120 -> -10

36 -> 157 -> -120 -> -11

36 -> 157 -> -120 -> -12

36 -> 157 -> -120 -> -13

36 -> 157 -> -120 -> -14

36 -> 157 -> -120 -> -16

36 -> 157 -> -120 -> -17

36 -> 157 -> -120 -> -18

36 -> 157 -> -120 -> -19

36 -> 157 -> -120 -> -21

36 -> 157 -> -120 -> -22

36 -> 157 -> -120 -> -23

36 -> 157 -> -120 -> -24

36 -> 157 -> -120 -> -25

36 -> 157 -> -120 -> -26

36 -> 157 -> -120 -> -27

36 -> 157 -> -120 -> -28

36 -> 157 -> -120 -> -29

36 -> 157 -> -120 -> -30

36 -> 157 -> -120 -> -31

36 -> 157 -> -120 -> -33

36 -> 157 -> -120 -> -34

36 -> 157 -> -120 -> -35

36 -> 157 -> -120 -> -36

36 -> 157 -> -120 -> -37

36 -> 157 -> -120 -> -38

36 -> 157 -> -120 -> -39

36 -> 157 -> -120 -> -40

36 -> 157 -> -120 -> -41

36 -> 157 -> -120 -> -42

36 -> 157 -> -120 -> -43

36 -> 157 -> -120 -> -44

36 -> 157 -> -120 -> -45

36 -> 157 -> -120 -> -46

36 -> 157 -> -120 -> -47

36 -> 157 -> -120 -> -48

36 -> 157 -> -120 -> -49

36 -> 157 -> -120 -> -51

36 -> 157 -> -120 -> -52

36 -> 157 -> -120 -> -55

36 -> 157 -> -120 -> -57

36 -> 157 -> -120 -> -58

36 -> 157 -> -120 -> -59

36 -> 157 -> -120 -> -60

36 -> 157 -> -120 -> -61

36 -> 157 -> -120 -> -62

36 -> 157 -> -120 -> -63

36 -> 157 -> -120 -> -64

36 -> 157 -> -120 -> -65

36 -> 157 -> -120 -> -66

36 -> 157 -> -120 -> -67

36 -> 157 -> -120 -> -69

36 -> 157 -> -120 -> -70

36 -> 157 -> -120 -> -71

36 -> 157 -> -120 -> -72

36 -> 157 -> -120 -> -76

36 -> 157 -> -120 -> -77

36 -> 157 -> -120 -> -78

36 -> 157 -> -120 -> -79

36 -> 157 -> -120 -> -82

36 -> 157 -> -120 -> -83

36 -> 157 -> -120 -> -84

36 -> 157 -> -120 -> -85

36 -> 157 -> -120 -> -86

36 -> 157 -> -120 -> -87

36 -> 157 -> -120 -> -88

36 -> 157 -> -120 -> -89

36 -> 157 -> -120 -> -90

36 -> 157 -> -120 -> -91

36 -> 157 -> -120 -> -92

36 -> 157 -> -120 -> -93

36 -> 157 -> -120 -> -94

36 -> 157 -> -120 -> -95

36 -> 157 -> -120 -> -96

36 -> 157 -> -120 -> -97

36 -> 157 -> -120 -> -98

36 -> 157 -> -120 -> -100

36 -> 157 -> -120 -> -102

36 -> 157 -> -120 -> -104

36 -> 157 -> -120 -> -105

36 -> 157 -> -120 -> -107

36 -> 157 -> -120 -> -108

36 -> 157 -> -120 -> -109

36 -> 157 -> -120 -> -110

36 -> 157 -> -120 -> -111

36 -> 157 -> -120 -> -113

36 -> 157 -> -120 -> -115

36 -> 157 -> -120 -> -116

36 -> 157 -> -120 -> -117

36 -> 157 -> -120 -> -118

36 -> 157 -> -120 -> -119

36 -> 157 -> -120 -> -121

36 -> 157 -> -120 -> -122

36 -> 157 -> -120 -> -123

36 -> 157 -> -120 -> -124

36 -> 157 -> -120 -> -125

36 -> 157 -> -120 -> -126

36 -> 157 -> -120 -> -127

36 -> 157 -> -120 -> -128

36 -> 157 -> -120 -> -129

36 -> 157 -> -120 -> -130

36 -> 157 -> -120 -> -131

36 -> 157 -> -120 -> -132

36 -> 157 -> -120 -> -134

36 -> 157 -> -120 -> -135

36 -> 157 -> -120 -> -136

36 -> 157 -> -120 -> -137

36 -> 157 -> -120 -> -138

36 -> 157 -> -120 -> -139

36 -> 157 -> -120 -> -140

36 -> 157 -> -120 -> -141

36 -> 157 -> -120 -> -142

36 -> 157 -> -120 -> -143

36 -> 157 -> -120 -> -144

36 -> 157 -> -120 -> -146

36 -> 157 -> -120 -> -147

36 -> 157 -> -120 -> -148

36 -> 157 -> -120 -> -149

36 -> 157 -> -120 -> -150

36 -> 157 -> -120 -> -151

36 -> 157 -> -120 -> -152

36 -> 157 -> -120 -> -153

36 -> 157 -> -120 -> -155

36 -> 157 -> -120 -> -156

36 -> 157 -> -121 -> -1

36 -> 157 -> -121 -> -3

36 -> 157 -> -121 -> -4

36 -> 157 -> -121 -> -8

36 -> 157 -> -121 -> -9

36 -> 157 -> -121 -> -10

36 -> 157 -> -121 -> -19

36 -> 157 -> -121 -> -20

36 -> 157 -> -121 -> -22

36 -> 157 -> -121 -> -24

36 -> 157 -> -121 -> -26

36 -> 157 -> -121 -> -27

36 -> 157 -> -121 -> -28

36 -> 157 -> -121 -> -29

36 -> 157 -> -121 -> -31

36 -> 157 -> -121 -> -33

36 -> 157 -> -121 -> -39

36 -> 157 -> -121 -> -44

36 -> 157 -> -121 -> -45

36 -> 157 -> -121 -> -46

36 -> 157 -> -121 -> -47

36 -> 157 -> -121 -> -49

36 -> 157 -> -121 -> -58

36 -> 157 -> -121 -> -60

36 -> 157 -> -121 -> -61

36 -> 157 -> -121 -> -62

36 -> 157 -> -121 -> -63

36 -> 157 -> -121 -> -66

36 -> 157 -> -121 -> -68

36 -> 157 -> -121 -> -69

36 -> 157 -> -121 -> -73

36 -> 157 -> -121 -> -77

36 -> 157 -> -121 -> -78

36 -> 157 -> -121 -> -81

36 -> 157 -> -121 -> -84

36 -> 157 -> -121 -> -86

36 -> 157 -> -121 -> -88

36 -> 157 -> -121 -> -91

36 -> 157 -> -121 -> -96

36 -> 157 -> -121 -> -98

36 -> 157 -> -121 -> -99

36 -> 157 -> -121 -> -101

36 -> 157 -> -121 -> -107

36 -> 157 -> -121 -> -117

36 -> 157 -> -121 -> -118

36 -> 157 -> -121 -> -119

36 -> 157 -> -121 -> -120

36 -> 157 -> -121 -> -122

36 -> 157 -> -121 -> -124

36 -> 157 -> -121 -> -131

36 -> 157 -> -121 -> -132

36 -> 157 -> -121 -> -133

36 -> 157 -> -121 -> -135

36 -> 157 -> -121 -> -136

36 -> 157 -> -121 -> -137

36 -> 157 -> -121 -> -141

36 -> 157 -> -121 -> -145

36 -> 157 -> -121 -> -147

36 -> 157 -> -121 -> -150

36 -> 157 -> -121 -> -156

36 -> 157 -> -122 -> -1

36 -> 157 -> -122 -> -3

36 -> 157 -> -122 -> -4

36 -> 157 -> -122 -> -8

36 -> 157 -> -122 -> -9

36 -> 157 -> -122 -> -10

36 -> 157 -> -122 -> -19

36 -> 157 -> -122 -> -20

36 -> 157 -> -122 -> -22

36 -> 157 -> -122 -> -24

36 -> 157 -> -122 -> -26

36 -> 157 -> -122 -> -27

36 -> 157 -> -122 -> -28

36 -> 157 -> -122 -> -29

36 -> 157 -> -122 -> -31

36 -> 157 -> -122 -> -33

36 -> 157 -> -122 -> -39

36 -> 157 -> -122 -> -44

36 -> 157 -> -122 -> -45

36 -> 157 -> -122 -> -46

36 -> 157 -> -122 -> -47

36 -> 157 -> -122 -> -49

36 -> 157 -> -122 -> -58

36 -> 157 -> -122 -> -60

36 -> 157 -> -122 -> -61

36 -> 157 -> -122 -> -62

36 -> 157 -> -122 -> -63

36 -> 157 -> -122 -> -66

36 -> 157 -> -122 -> -68

36 -> 157 -> -122 -> -69

36 -> 157 -> -122 -> -73

36 -> 157 -> -122 -> -77

36 -> 157 -> -122 -> -78

36 -> 157 -> -122 -> -81

36 -> 157 -> -122 -> -84

36 -> 157 -> -122 -> -86

36 -> 157 -> -122 -> -88

36 -> 157 -> -122 -> -91

36 -> 157 -> -122 -> -96

36 -> 157 -> -122 -> -98

36 -> 157 -> -122 -> -99

36 -> 157 -> -122 -> -101

36 -> 157 -> -122 -> -107

36 -> 157 -> -122 -> -117

36 -> 157 -> -122 -> -118

36 -> 157 -> -122 -> -119

36 -> 157 -> -122 -> -120

36 -> 157 -> -122 -> -121

36 -> 157 -> -122 -> -124

36 -> 157 -> -122 -> -131

36 -> 157 -> -122 -> -132

36 -> 157 -> -122 -> -133

36 -> 157 -> -122 -> -135

36 -> 157 -> -122 -> -136

36 -> 157 -> -122 -> -137

36 -> 157 -> -122 -> -141

36 -> 157 -> -122 -> -145

36 -> 157 -> -122 -> -147

36 -> 157 -> -122 -> -150

36 -> 157 -> -122 -> -156

36 -> 157 -> -133

36 -> 157 -> -133 -> -1

36 -> 157 -> -133 -> -3

36 -> 157 -> -133 -> -19

36 -> 157 -> -133 -> -20

36 -> 157 -> -133 -> -22

36 -> 157 -> -133 -> -26

36 -> 157 -> -133 -> -27

36 -> 157 -> -133 -> -28

36 -> 157 -> -133 -> -29

36 -> 157 -> -133 -> -32

36 -> 157 -> -133 -> -36

36 -> 157 -> -133 -> -39

36 -> 157 -> -133 -> -40

36 -> 157 -> -133 -> -48

36 -> 157 -> -133 -> -56

36 -> 157 -> -133 -> -61

36 -> 157 -> -133 -> -62

36 -> 157 -> -133 -> -63

36 -> 157 -> -133 -> -64

36 -> 157 -> -133 -> -68

36 -> 157 -> -133 -> -73

36 -> 157 -> -133 -> -78

36 -> 157 -> -133 -> -79

36 -> 157 -> -133 -> -81

36 -> 157 -> -133 -> -86

36 -> 157 -> -133 -> -94

36 -> 157 -> -133 -> -95

36 -> 157 -> -133 -> -98

36 -> 157 -> -133 -> -99

36 -> 157 -> -133 -> -101

36 -> 157 -> -133 -> -106

36 -> 157 -> -133 -> -107

36 -> 157 -> -133 -> -114

36 -> 157 -> -133 -> -117

36 -> 157 -> -133 -> -118

36 -> 157 -> -133 -> -119

36 -> 157 -> -133 -> -121

36 -> 157 -> -133 -> -122

36 -> 157 -> -133 -> -123

36 -> 157 -> -133 -> -124

36 -> 157 -> -133 -> -125

36 -> 157 -> -133 -> -137

36 -> 157 -> -133 -> -145

36 -> 157 -> -133 -> -146

36 -> 157 -> -133 -> -147

36 -> 157 -> -133 -> -150

36 -> 157 -> -133 -> -154

36 -> 157 -> -133 -> -156

36 -> 161 -> 3 -> -32

36 -> 161 -> 3 -> -42

36 -> 161 -> 4 -> -101

36 -> 161 -> 6 -> -73

36 -> 161 -> 7 -> -54

36 -> 161 -> 9 -> -6

36 -> 161 -> 14 -> -54

36 -> 161 -> 15 -> -68

36 -> 161 -> 17 -> -32

36 -> 161 -> 19 -> -73

36 -> 161 -> 21 -> -73

36 -> 161 -> 21 -> -101

36 -> 161 -> 21 -> -154

36 -> 161 -> 22 -> -152

36 -> 161 -> 24 -> -49

36 -> 161 -> 25 -> -132

36 -> 161 -> 27 -> -19

36 -> 161 -> 27 -> -20

36 -> 161 -> 27 -> -32

36 -> 161 -> 27 -> -62

36 -> 161 -> 27 -> -68

36 -> 161 -> 27 -> -73

36 -> 161 -> 27 -> -81

36 -> 161 -> 27 -> -98

36 -> 161 -> 27 -> -99

36 -> 161 -> 27 -> -101

36 -> 161 -> 27 -> -107

36 -> 161 -> 27 -> -124

36 -> 161 -> 27 -> -133

36 -> 161 -> 27 -> -145

36 -> 161 -> 28 -> -81

36 -> 161 -> 29 -> -133

36 -> 161 -> 30 -> -1

36 -> 161 -> 30 -> -3

36 -> 161 -> 30 -> -32

36 -> 161 -> 30 -> -39

36 -> 161 -> 30 -> -63

36 -> 161 -> 30 -> -73

36 -> 161 -> 30 -> -81

36 -> 161 -> 30 -> -99

36 -> 161 -> 30 -> -101

36 -> 161 -> 30 -> -106

36 -> 161 -> 30 -> -117

36 -> 161 -> 30 -> -133

36 -> 161 -> 30 -> -137

36 -> 161 -> 30 -> -150

36 -> 161 -> 30 -> -156

36 -> 161 -> 32 -> -9

36 -> 161 -> 33 -> -145

36 -> 161 -> 38 -> -55

36 -> 161 -> 38 -> -56

36 -> 161 -> 38 -> -105

36 -> 161 -> 39 -> -101

36 -> 161 -> 40 -> -73

36 -> 161 -> 41 -> -6

36 -> 161 -> 42 -> -133

36 -> 161 -> 43 -> -73

36 -> 161 -> 43 -> -99

36 -> 161 -> 43 -> -101

36 -> 161 -> 44 -> -29

36 -> 161 -> 44 -> -48

36 -> 161 -> 44 -> -81

36 -> 161 -> 44 -> -89

36 -> 161 -> 44 -> -99

36 -> 161 -> 44 -> -114

36 -> 161 -> 45 -> -73

36 -> 161 -> 48 -> -99

36 -> 161 -> 49 -> -15

36 -> 161 -> 55 -> -12

36 -> 161 -> 60 -> -64

36 -> 161 -> 61 -> -27

36 -> 161 -> 64 -> -10

36 -> 161 -> 65 -> -4

36 -> 161 -> 66 -> -136

36 -> 161 -> 67 -> -135

36 -> 161 -> 68 -> -73

36 -> 161 -> 69 -> -77

36 -> 161 -> 73 -> -32

36 -> 161 -> 74 -> -112

36 -> 161 -> 75 -> -1

36 -> 161 -> 75 -> -29

36 -> 161 -> 76 -> -81

36 -> 161 -> 77 -> -73

36 -> 161 -> 79 -> -73

36 -> 161 -> 81 -> -73

36 -> 161 -> 82 -> -30

36 -> 161 -> 82 -> -32

36 -> 161 -> 82 -> -35

36 -> 161 -> 82 -> -101

36 -> 161 -> 83 -> -125

36 -> 161 -> 84 -> -73

36 -> 161 -> 87 -> -73

36 -> 161 -> 88 -> -83

36 -> 161 -> 89 -> -6

36 -> 161 -> 89 -> -73

36 -> 161 -> 89 -> -81

36 -> 161 -> 89 -> -83

36 -> 161 -> 89 -> -86

36 -> 161 -> 89 -> -99

36 -> 161 -> 89 -> -101

36 -> 161 -> 89 -> -114

36 -> 161 -> 89 -> -155

36 -> 161 -> 91 -> -101

36 -> 161 -> 92 -> -133

36 -> 161 -> 97 -> -15

36 -> 161 -> 103 -> -78

36 -> 161 -> 105 -> -32

36 -> 161 -> 105 -> -146

36 -> 161 -> 107 -> -73

36 -> 161 -> 107 -> -99

36 -> 161 -> 108 -> -80

36 -> 161 -> 113 -> -73

36 -> 161 -> 114 -> -73

36 -> 161 -> 115 -> -17

36 -> 161 -> 115 -> -32

36 -> 161 -> 115 -> -68

36 -> 161 -> 115 -> -73

36 -> 161 -> 115 -> -92

36 -> 161 -> 116 -> -73

36 -> 161 -> 117 -> -73

36 -> 161 -> 117 -> -101

36 -> 161 -> 118 -> -73

36 -> 161 -> 120 -> -133

36 -> 161 -> 122 -> -56

36 -> 161 -> 123 -> -73

36 -> 161 -> 128 -> -32

36 -> 161 -> 128 -> -95

36 -> 161 -> 129 -> -32

36 -> 161 -> 130 -> -73

36 -> 161 -> 133 -> -101

36 -> 161 -> 134 -> -99

36 -> 161 -> 136 -> -20

36 -> 161 -> 139 -> -15

36 -> 161 -> 141 -> -51

36 -> 161 -> 141 -> -100

36 -> 161 -> 141 -> -127

36 -> 161 -> 142 -> -154

36 -> 161 -> 144 -> -7

36 -> 161 -> 144 -> -110

36 -> 161 -> 144 -> -130

36 -> 161 -> 146 -> -20

36 -> 161 -> 148 -> -32

36 -> 161 -> 149 -> -73

36 -> 161 -> 150 -> -91

36 -> 161 -> 151 -> -101

36 -> 161 -> 152 -> -36

36 -> 161 -> 153 -> -2

36 -> 161 -> 160 -> -129

36 -> 161 -> 162 -> -133

36 -> 161 -> 163 -> -8

36 -> 161 -> 169 -> -99

36 -> 161 -> 170 -> -138

36 -> 161 -> 171 -> -13

36 -> 161 -> 172 -> -58

36 -> 161 -> 173 -> -154

36 -> 161 -> 174 -> -114

36 -> 161 -> 176 -> -33

36 -> 161 -> 177 -> -106

36 -> 161 -> 179 -> -22

36 -> 161 -> 179 -> -147

36 -> 161 -> 180 -> -20

36 -> 161 -> 181 -> -84

36 -> 161 -> 182 -> -73

36 -> 161 -> 186 -> -99

36 -> 161 -> 188 -> -24

36 -> 161 -> 189 -> -73

36 -> 161 -> 190 -> -43

36 -> 161 -> 190 -> -73

36 -> 161 -> 190 -> -86

36 -> 161 -> 190 -> -88

36 -> 161 -> 190 -> -144

36 -> 161 -> -78

36 -> 161 -> -78 -> -1

36 -> 161 -> -78 -> -3

36 -> 161 -> -78 -> -4

36 -> 161 -> -78 -> -6

36 -> 161 -> -78 -> -8

36 -> 161 -> -78 -> -9

36 -> 161 -> -78 -> -10

36 -> 161 -> -78 -> -17

36 -> 161 -> -78 -> -19

36 -> 161 -> -78 -> -20

36 -> 161 -> -78 -> -22

36 -> 161 -> -78 -> -24

36 -> 161 -> -78 -> -26

36 -> 161 -> -78 -> -27

36 -> 161 -> -78 -> -28

36 -> 161 -> -78 -> -29

36 -> 161 -> -78 -> -30

36 -> 161 -> -78 -> -31

36 -> 161 -> -78 -> -32

36 -> 161 -> -78 -> -33

36 -> 161 -> -78 -> -35

36 -> 161 -> -78 -> -36

36 -> 161 -> -78 -> -39

36 -> 161 -> -78 -> -43

36 -> 161 -> -78 -> -44

36 -> 161 -> -78 -> -45

36 -> 161 -> -78 -> -46

36 -> 161 -> -78 -> -47

36 -> 161 -> -78 -> -48

36 -> 161 -> -78 -> -49

36 -> 161 -> -78 -> -51

36 -> 161 -> -78 -> -56

36 -> 161 -> -78 -> -58

36 -> 161 -> -78 -> -60

36 -> 161 -> -78 -> -61

36 -> 161 -> -78 -> -62

36 -> 161 -> -78 -> -63

36 -> 161 -> -78 -> -64

36 -> 161 -> -78 -> -66

36 -> 161 -> -78 -> -68

36 -> 161 -> -78 -> -69

36 -> 161 -> -78 -> -73

36 -> 161 -> -78 -> -77

36 -> 161 -> -78 -> -81

36 -> 161 -> -78 -> -83

36 -> 161 -> -78 -> -84

36 -> 161 -> -78 -> -86

36 -> 161 -> -78 -> -88

36 -> 161 -> -78 -> -89

36 -> 161 -> -78 -> -91

36 -> 161 -> -78 -> -92

36 -> 161 -> -78 -> -95

36 -> 161 -> -78 -> -96

36 -> 161 -> -78 -> -98

36 -> 161 -> -78 -> -99

36 -> 161 -> -78 -> -100

36 -> 161 -> -78 -> -101

36 -> 161 -> -78 -> -106

36 -> 161 -> -78 -> -107

36 -> 161 -> -78 -> -114

36 -> 161 -> -78 -> -117

36 -> 161 -> -78 -> -118

36 -> 161 -> -78 -> -119

36 -> 161 -> -78 -> -120

36 -> 161 -> -78 -> -121

36 -> 161 -> -78 -> -122

36 -> 161 -> -78 -> -124

36 -> 161 -> -78 -> -127

36 -> 161 -> -78 -> -131

36 -> 161 -> -78 -> -132

36 -> 161 -> -78 -> -133

36 -> 161 -> -78 -> -135

36 -> 161 -> -78 -> -136

36 -> 161 -> -78 -> -137

36 -> 161 -> -78 -> -141

36 -> 161 -> -78 -> -144

36 -> 161 -> -78 -> -145

36 -> 161 -> -78 -> -146

36 -> 161 -> -78 -> -147

36 -> 161 -> -78 -> -150

36 -> 161 -> -78 -> -154

36 -> 161 -> -78 -> -155

36 -> 161 -> -78 -> -156

36 -> 179 -> 3 -> -32

36 -> 179 -> 3 -> -42

36 -> 179 -> 4 -> -101

36 -> 179 -> 6 -> -73

36 -> 179 -> 21 -> -73

36 -> 179 -> 21 -> -101

36 -> 179 -> 21 -> -154

36 -> 179 -> 27 -> -19

36 -> 179 -> 27 -> -20

36 -> 179 -> 27 -> -32

36 -> 179 -> 27 -> -62

36 -> 179 -> 27 -> -68

36 -> 179 -> 27 -> -73

36 -> 179 -> 27 -> -81

36 -> 179 -> 27 -> -98

36 -> 179 -> 27 -> -99

36 -> 179 -> 27 -> -101

36 -> 179 -> 27 -> -107

36 -> 179 -> 27 -> -124

36 -> 179 -> 27 -> -133

36 -> 179 -> 27 -> -145

36 -> 179 -> 29 -> -133

36 -> 179 -> 30 -> -1

36 -> 179 -> 30 -> -3

36 -> 179 -> 30 -> -32

36 -> 179 -> 30 -> -39

36 -> 179 -> 30 -> -63

36 -> 179 -> 30 -> -73

36 -> 179 -> 30 -> -81

36 -> 179 -> 30 -> -99

36 -> 179 -> 30 -> -101

36 -> 179 -> 30 -> -106

36 -> 179 -> 30 -> -117

36 -> 179 -> 30 -> -133

36 -> 179 -> 30 -> -137

36 -> 179 -> 30 -> -150

36 -> 179 -> 30 -> -156

36 -> 179 -> 34 -> -32

36 -> 179 -> 34 -> -71

36 -> 179 -> 34 -> -113

36 -> 179 -> 37 -> -73

36 -> 179 -> 38 -> -55

36 -> 179 -> 38 -> -56

36 -> 179 -> 38 -> -105

36 -> 179 -> 39 -> -101

36 -> 179 -> 40 -> -73

36 -> 179 -> 43 -> -73

36 -> 179 -> 43 -> -99

36 -> 179 -> 43 -> -101

36 -> 179 -> 52 -> -32

36 -> 179 -> 60 -> -64

36 -> 179 -> 61 -> -27

36 -> 179 -> 62 -> -99

36 -> 179 -> 75 -> -1

36 -> 179 -> 75 -> -29

36 -> 179 -> 76 -> -81

36 -> 179 -> 77 -> -73

36 -> 179 -> 78 -> -73

36 -> 179 -> 92 -> -133

36 -> 179 -> 99 -> -73

36 -> 179 -> 103 -> -78

36 -> 179 -> 105 -> -32

36 -> 179 -> 105 -> -146

36 -> 179 -> 107 -> -73

36 -> 179 -> 107 -> -99

36 -> 179 -> 109 -> -20

36 -> 179 -> 109 -> -73

36 -> 179 -> 109 -> -99

36 -> 179 -> 109 -> -101

36 -> 179 -> 110 -> -56

36 -> 179 -> 115 -> -17

36 -> 179 -> 115 -> -32

36 -> 179 -> 115 -> -68

36 -> 179 -> 115 -> -73

36 -> 179 -> 115 -> -92

36 -> 179 -> 117 -> -73

36 -> 179 -> 117 -> -101

36 -> 179 -> 122 -> -56

36 -> 179 -> 128 -> -32

36 -> 179 -> 128 -> -95

36 -> 179 -> 129 -> -32

36 -> 179 -> 136 -> -20

36 -> 179 -> 138 -> -20

36 -> 179 -> 138 -> -61

36 -> 179 -> 138 -> -73

36 -> 179 -> 138 -> -98

36 -> 179 -> 138 -> -133

36 -> 179 -> 140 -> -99

36 -> 179 -> 140 -> -133

36 -> 179 -> 144 -> -7

36 -> 179 -> 144 -> -110

36 -> 179 -> 144 -> -130

36 -> 179 -> 146 -> -20

36 -> 179 -> 152 -> -36

36 -> 179 -> 157 -> -26

36 -> 179 -> 157 -> -28

36 -> 179 -> 157 -> -32

36 -> 179 -> 157 -> -68

36 -> 179 -> 157 -> -86

36 -> 179 -> 157 -> -99

36 -> 179 -> 157 -> -101

36 -> 179 -> 157 -> -118

36 -> 179 -> 157 -> -119

36 -> 179 -> 157 -> -120

36 -> 179 -> 157 -> -121

36 -> 179 -> 157 -> -122

36 -> 179 -> 157 -> -133

36 -> 179 -> 161 -> -78

36 -> 179 -> 169 -> -99

36 -> 179 -> 180 -> -20

36 -> 179 -> 182 -> -73

36 -> 179 -> 186 -> -99

36 -> 179 -> -22 -> -1

36 -> 179 -> -22 -> -3

36 -> 179 -> -22 -> -4

36 -> 179 -> -22 -> -7

36 -> 179 -> -22 -> -8

36 -> 179 -> -22 -> -9

36 -> 179 -> -22 -> -10

36 -> 179 -> -22 -> -17

36 -> 179 -> -22 -> -19

36 -> 179 -> -22 -> -20

36 -> 179 -> -22 -> -24

36 -> 179 -> -22 -> -26

36 -> 179 -> -22 -> -27

36 -> 179 -> -22 -> -28

36 -> 179 -> -22 -> -29

36 -> 179 -> -22 -> -31

36 -> 179 -> -22 -> -32

36 -> 179 -> -22 -> -33

36 -> 179 -> -22 -> -36

36 -> 179 -> -22 -> -39

36 -> 179 -> -22 -> -42

36 -> 179 -> -22 -> -43

36 -> 179 -> -22 -> -44

36 -> 179 -> -22 -> -45

36 -> 179 -> -22 -> -46

36 -> 179 -> -22 -> -47

36 -> 179 -> -22 -> -49

36 -> 179 -> -22 -> -55

36 -> 179 -> -22 -> -56

36 -> 179 -> -22 -> -58

36 -> 179 -> -22 -> -60

36 -> 179 -> -22 -> -61

36 -> 179 -> -22 -> -62

36 -> 179 -> -22 -> -63

36 -> 179 -> -22 -> -64

36 -> 179 -> -22 -> -66

36 -> 179 -> -22 -> -68

36 -> 179 -> -22 -> -69

36 -> 179 -> -22 -> -71

36 -> 179 -> -22 -> -73

36 -> 179 -> -22 -> -77

36 -> 179 -> -22 -> -78

36 -> 179 -> -22 -> -81

36 -> 179 -> -22 -> -84

36 -> 179 -> -22 -> -86

36 -> 179 -> -22 -> -88

36 -> 179 -> -22 -> -91

36 -> 179 -> -22 -> -92

36 -> 179 -> -22 -> -95

36 -> 179 -> -22 -> -96

36 -> 179 -> -22 -> -98

36 -> 179 -> -22 -> -99

36 -> 179 -> -22 -> -101

36 -> 179 -> -22 -> -105

36 -> 179 -> -22 -> -106

36 -> 179 -> -22 -> -107

36 -> 179 -> -22 -> -110

36 -> 179 -> -22 -> -113

36 -> 179 -> -22 -> -117

36 -> 179 -> -22 -> -118

36 -> 179 -> -22 -> -119

36 -> 179 -> -22 -> -120

36 -> 179 -> -22 -> -121

36 -> 179 -> -22 -> -122

36 -> 179 -> -22 -> -124

36 -> 179 -> -22 -> -130

36 -> 179 -> -22 -> -131

36 -> 179 -> -22 -> -132

36 -> 179 -> -22 -> -133

36 -> 179 -> -22 -> -135

36 -> 179 -> -22 -> -136

36 -> 179 -> -22 -> -137

36 -> 179 -> -22 -> -141

36 -> 179 -> -22 -> -144

36 -> 179 -> -22 -> -145

36 -> 179 -> -22 -> -147

36 -> 179 -> -22 -> -150

36 -> 179 -> -22 -> -156

36 -> 179 -> -147 -> -1

36 -> 179 -> -147 -> -3

36 -> 179 -> -147 -> -4

36 -> 179 -> -147 -> -7

36 -> 179 -> -147 -> -8

36 -> 179 -> -147 -> -9

36 -> 179 -> -147 -> -10

36 -> 179 -> -147 -> -17

36 -> 179 -> -147 -> -19

36 -> 179 -> -147 -> -20

36 -> 179 -> -147 -> -22

36 -> 179 -> -147 -> -24

36 -> 179 -> -147 -> -26

36 -> 179 -> -147 -> -27

36 -> 179 -> -147 -> -28

36 -> 179 -> -147 -> -29

36 -> 179 -> -147 -> -31

36 -> 179 -> -147 -> -32

36 -> 179 -> -147 -> -33

36 -> 179 -> -147 -> -36

36 -> 179 -> -147 -> -39

36 -> 179 -> -147 -> -42

36 -> 179 -> -147 -> -43

36 -> 179 -> -147 -> -44

36 -> 179 -> -147 -> -45

36 -> 179 -> -147 -> -46

36 -> 179 -> -147 -> -47

36 -> 179 -> -147 -> -49

36 -> 179 -> -147 -> -55

36 -> 179 -> -147 -> -56

36 -> 179 -> -147 -> -58

36 -> 179 -> -147 -> -60

36 -> 179 -> -147 -> -61

36 -> 179 -> -147 -> -62

36 -> 179 -> -147 -> -63

36 -> 179 -> -147 -> -64

36 -> 179 -> -147 -> -66

36 -> 179 -> -147 -> -68

36 -> 179 -> -147 -> -69

36 -> 179 -> -147 -> -71

36 -> 179 -> -147 -> -73

36 -> 179 -> -147 -> -77

36 -> 179 -> -147 -> -78

36 -> 179 -> -147 -> -81

36 -> 179 -> -147 -> -84

36 -> 179 -> -147 -> -86

36 -> 179 -> -147 -> -88

36 -> 179 -> -147 -> -91

36 -> 179 -> -147 -> -92

36 -> 179 -> -147 -> -95

36 -> 179 -> -147 -> -96

36 -> 179 -> -147 -> -98

36 -> 179 -> -147 -> -99

36 -> 179 -> -147 -> -101

36 -> 179 -> -147 -> -105

36 -> 179 -> -147 -> -106

36 -> 179 -> -147 -> -107

36 -> 179 -> -147 -> -110

36 -> 179 -> -147 -> -113

36 -> 179 -> -147 -> -117

36 -> 179 -> -147 -> -118

36 -> 179 -> -147 -> -119

36 -> 179 -> -147 -> -120

36 -> 179 -> -147 -> -121

36 -> 179 -> -147 -> -122

36 -> 179 -> -147 -> -124

36 -> 179 -> -147 -> -130

36 -> 179 -> -147 -> -131

36 -> 179 -> -147 -> -132

36 -> 179 -> -147 -> -133

36 -> 179 -> -147 -> -135

36 -> 179 -> -147 -> -136

36 -> 179 -> -147 -> -137

36 -> 179 -> -147 -> -141

36 -> 179 -> -147 -> -144

36 -> 179 -> -147 -> -145

36 -> 179 -> -147 -> -150

36 -> 179 -> -147 -> -156

36 -> 180 -> 3 -> -32

36 -> 180 -> 3 -> -42

36 -> 180 -> 4 -> -101

36 -> 180 -> 7 -> -54

36 -> 180 -> 8 -> -20

36 -> 180 -> 8 -> -88

36 -> 180 -> 9 -> -6

36 -> 180 -> 14 -> -54

36 -> 180 -> 15 -> -68

36 -> 180 -> 19 -> -73

36 -> 180 -> 21 -> -73

36 -> 180 -> 21 -> -101

36 -> 180 -> 21 -> -154

36 -> 180 -> 22 -> -152

36 -> 180 -> 24 -> -49

36 -> 180 -> 25 -> -132

36 -> 180 -> 27 -> -19

36 -> 180 -> 27 -> -20

36 -> 180 -> 27 -> -32

36 -> 180 -> 27 -> -62

36 -> 180 -> 27 -> -68

36 -> 180 -> 27 -> -73

36 -> 180 -> 27 -> -81

36 -> 180 -> 27 -> -98

36 -> 180 -> 27 -> -99

36 -> 180 -> 27 -> -101

36 -> 180 -> 27 -> -107

36 -> 180 -> 27 -> -124

36 -> 180 -> 27 -> -133

36 -> 180 -> 27 -> -145

36 -> 180 -> 28 -> -81

36 -> 180 -> 29 -> -133

36 -> 180 -> 30 -> -1

36 -> 180 -> 30 -> -3

36 -> 180 -> 30 -> -32

36 -> 180 -> 30 -> -39

36 -> 180 -> 30 -> -63

36 -> 180 -> 30 -> -73

36 -> 180 -> 30 -> -81

36 -> 180 -> 30 -> -99

36 -> 180 -> 30 -> -101

36 -> 180 -> 30 -> -106

36 -> 180 -> 30 -> -117

36 -> 180 -> 30 -> -133

36 -> 180 -> 30 -> -137

36 -> 180 -> 30 -> -150

36 -> 180 -> 30 -> -156

36 -> 180 -> 32 -> -9

36 -> 180 -> 33 -> -145

36 -> 180 -> 39 -> -101

36 -> 180 -> 41 -> -6

36 -> 180 -> 42 -> -133

36 -> 180 -> 44 -> -29

36 -> 180 -> 44 -> -48

36 -> 180 -> 44 -> -81

36 -> 180 -> 44 -> -89

36 -> 180 -> 44 -> -99

36 -> 180 -> 44 -> -114

36 -> 180 -> 45 -> -73

36 -> 180 -> 48 -> -99

36 -> 180 -> 49 -> -15

36 -> 180 -> 55 -> -12

36 -> 180 -> 60 -> -64

36 -> 180 -> 64 -> -10

36 -> 180 -> 65 -> -4

36 -> 180 -> 66 -> -136

36 -> 180 -> 67 -> -135

36 -> 180 -> 68 -> -73

36 -> 180 -> 69 -> -77

36 -> 180 -> 73 -> -32

36 -> 180 -> 74 -> -112

36 -> 180 -> 75 -> -1

36 -> 180 -> 75 -> -29

36 -> 180 -> 76 -> -81

36 -> 180 -> 77 -> -73

36 -> 180 -> 79 -> -73

36 -> 180 -> 81 -> -73

36 -> 180 -> 84 -> -73

36 -> 180 -> 87 -> -73

36 -> 180 -> 88 -> -83

36 -> 180 -> 89 -> -6

36 -> 180 -> 89 -> -73

36 -> 180 -> 89 -> -81

36 -> 180 -> 89 -> -83

36 -> 180 -> 89 -> -86

36 -> 180 -> 89 -> -99

36 -> 180 -> 89 -> -101

36 -> 180 -> 89 -> -114

36 -> 180 -> 89 -> -155

36 -> 180 -> 91 -> -101

36 -> 180 -> 92 -> -133

36 -> 180 -> 97 -> -15

36 -> 180 -> 103 -> -78

36 -> 180 -> 105 -> -32

36 -> 180 -> 105 -> -146

36 -> 180 -> 107 -> -73

36 -> 180 -> 107 -> -99

36 -> 180 -> 108 -> -80

36 -> 180 -> 113 -> -73

36 -> 180 -> 116 -> -73

36 -> 180 -> 118 -> -73

36 -> 180 -> 120 -> -133

36 -> 180 -> 122 -> -56

36 -> 180 -> 123 -> -73

36 -> 180 -> 128 -> -32

36 -> 180 -> 128 -> -95

36 -> 180 -> 129 -> -32

36 -> 180 -> 130 -> -73

36 -> 180 -> 134 -> -99

36 -> 180 -> 136 -> -20

36 -> 180 -> 139 -> -15

36 -> 180 -> 142 -> -154

36 -> 180 -> 146 -> -20

36 -> 180 -> 148 -> -32

36 -> 180 -> 149 -> -73

36 -> 180 -> 150 -> -91

36 -> 180 -> 153 -> -2

36 -> 180 -> 160 -> -129

36 -> 180 -> 161 -> -78

36 -> 180 -> 162 -> -133

36 -> 180 -> 163 -> -8

36 -> 180 -> 170 -> -138

36 -> 180 -> 171 -> -13

36 -> 180 -> 172 -> -58

36 -> 180 -> 176 -> -33

36 -> 180 -> 177 -> -106

36 -> 180 -> 179 -> -22

36 -> 180 -> 179 -> -147

36 -> 180 -> 181 -> -84

36 -> 180 -> 186 -> -99

36 -> 180 -> 188 -> -24

36 -> 180 -> 189 -> -73

36 -> 180 -> -20

36 -> 180 -> -20 -> -1

36 -> 180 -> -20 -> -3

36 -> 180 -> -20 -> -6

36 -> 180 -> -20 -> -16

36 -> 180 -> -20 -> -17

36 -> 180 -> -20 -> -19

36 -> 180 -> -20 -> -22

36 -> 180 -> -20 -> -26

36 -> 180 -> -20 -> -27

36 -> 180 -> -20 -> -28

36 -> 180 -> -20 -> -29

36 -> 180 -> -20 -> -32

36 -> 180 -> -20 -> -39

36 -> 180 -> -20 -> -43

36 -> 180 -> -20 -> -48

36 -> 180 -> -20 -> -56

36 -> 180 -> -20 -> -61

36 -> 180 -> -20 -> -62

36 -> 180 -> -20 -> -63

36 -> 180 -> -20 -> -64

36 -> 180 -> -20 -> -68

36 -> 180 -> -20 -> -70

36 -> 180 -> -20 -> -73

36 -> 180 -> -20 -> -74

36 -> 180 -> -20 -> -75

36 -> 180 -> -20 -> -78

36 -> 180 -> -20 -> -81

36 -> 180 -> -20 -> -82

36 -> 180 -> -20 -> -83

36 -> 180 -> -20 -> -86

36 -> 180 -> -20 -> -88

36 -> 180 -> -20 -> -89

36 -> 180 -> -20 -> -92

36 -> 180 -> -20 -> -95

36 -> 180 -> -20 -> -98

36 -> 180 -> -20 -> -99

36 -> 180 -> -20 -> -101

36 -> 180 -> -20 -> -106

36 -> 180 -> -20 -> -107

36 -> 180 -> -20 -> -114

36 -> 180 -> -20 -> -117

36 -> 180 -> -20 -> -118

36 -> 180 -> -20 -> -119

36 -> 180 -> -20 -> -121

36 -> 180 -> -20 -> -122

36 -> 180 -> -20 -> -124

36 -> 180 -> -20 -> -125

36 -> 180 -> -20 -> -133

36 -> 180 -> -20 -> -137

36 -> 180 -> -20 -> -140

36 -> 180 -> -20 -> -143

36 -> 180 -> -20 -> -144

36 -> 180 -> -20 -> -145

36 -> 180 -> -20 -> -146

36 -> 180 -> -20 -> -147

36 -> 180 -> -20 -> -150

36 -> 180 -> -20 -> -152

36 -> 180 -> -20 -> -154

36 -> 180 -> -20 -> -155

36 -> 180 -> -20 -> -156

36 -> 182 -> 7 -> -54

36 -> 182 -> 9 -> -6

36 -> 182 -> 14 -> -54

36 -> 182 -> 15 -> -68

36 -> 182 -> 19 -> -73

36 -> 182 -> 21 -> -73

36 -> 182 -> 21 -> -101

36 -> 182 -> 21 -> -154

36 -> 182 -> 22 -> -152

36 -> 182 -> 24 -> -49

36 -> 182 -> 25 -> -132

36 -> 182 -> 26 -> -14

36 -> 182 -> 27 -> -19

36 -> 182 -> 27 -> -20

36 -> 182 -> 27 -> -32

36 -> 182 -> 27 -> -62

36 -> 182 -> 27 -> -68

36 -> 182 -> 27 -> -73

36 -> 182 -> 27 -> -81

36 -> 182 -> 27 -> -98

36 -> 182 -> 27 -> -99

36 -> 182 -> 27 -> -101

36 -> 182 -> 27 -> -107

36 -> 182 -> 27 -> -124

36 -> 182 -> 27 -> -133

36 -> 182 -> 27 -> -145

36 -> 182 -> 28 -> -81

36 -> 182 -> 30 -> -1

36 -> 182 -> 30 -> -3

36 -> 182 -> 30 -> -32

36 -> 182 -> 30 -> -39

36 -> 182 -> 30 -> -63

36 -> 182 -> 30 -> -73

36 -> 182 -> 30 -> -81

36 -> 182 -> 30 -> -99

36 -> 182 -> 30 -> -101

36 -> 182 -> 30 -> -106

36 -> 182 -> 30 -> -117

36 -> 182 -> 30 -> -133

36 -> 182 -> 30 -> -137

36 -> 182 -> 30 -> -150

36 -> 182 -> 30 -> -156

36 -> 182 -> 32 -> -9

36 -> 182 -> 33 -> -145

36 -> 182 -> 34 -> -32

36 -> 182 -> 34 -> -71

36 -> 182 -> 34 -> -113

36 -> 182 -> 37 -> -73

36 -> 182 -> 41 -> -6

36 -> 182 -> 42 -> -133

36 -> 182 -> 44 -> -29

36 -> 182 -> 44 -> -48

36 -> 182 -> 44 -> -81

36 -> 182 -> 44 -> -89

36 -> 182 -> 44 -> -99

36 -> 182 -> 44 -> -114

36 -> 182 -> 45 -> -73

36 -> 182 -> 48 -> -99

36 -> 182 -> 49 -> -15

36 -> 182 -> 55 -> -12

36 -> 182 -> 60 -> -64

36 -> 182 -> 61 -> -27

36 -> 182 -> 63 -> -32

36 -> 182 -> 64 -> -10

36 -> 182 -> 65 -> -4

36 -> 182 -> 66 -> -136

36 -> 182 -> 67 -> -135

36 -> 182 -> 68 -> -73

36 -> 182 -> 69 -> -77

36 -> 182 -> 71 -> -32

36 -> 182 -> 73 -> -32

36 -> 182 -> 74 -> -112

36 -> 182 -> 76 -> -81

36 -> 182 -> 77 -> -73

36 -> 182 -> 78 -> -73

36 -> 182 -> 79 -> -73

36 -> 182 -> 84 -> -73

36 -> 182 -> 87 -> -73

36 -> 182 -> 88 -> -83

36 -> 182 -> 91 -> -101

36 -> 182 -> 92 -> -133

36 -> 182 -> 97 -> -15

36 -> 182 -> 99 -> -73

36 -> 182 -> 105 -> -32

36 -> 182 -> 105 -> -146

36 -> 182 -> 107 -> -73

36 -> 182 -> 107 -> -99

36 -> 182 -> 108 -> -80

36 -> 182 -> 113 -> -73

36 -> 182 -> 114 -> -73

36 -> 182 -> 115 -> -17

36 -> 182 -> 115 -> -32

36 -> 182 -> 115 -> -68

36 -> 182 -> 115 -> -73

36 -> 182 -> 115 -> -92

36 -> 182 -> 116 -> -73

36 -> 182 -> 117 -> -73

36 -> 182 -> 117 -> -101

36 -> 182 -> 118 -> -73

36 -> 182 -> 120 -> -133

36 -> 182 -> 122 -> -56

36 -> 182 -> 123 -> -73

36 -> 182 -> 128 -> -32

36 -> 182 -> 128 -> -95

36 -> 182 -> 129 -> -32

36 -> 182 -> 130 -> -73

36 -> 182 -> 134 -> -99

36 -> 182 -> 136 -> -20

36 -> 182 -> 138 -> -20

36 -> 182 -> 138 -> -61

36 -> 182 -> 138 -> -73

36 -> 182 -> 138 -> -98

36 -> 182 -> 138 -> -133

36 -> 182 -> 139 -> -15

36 -> 182 -> 142 -> -154

36 -> 182 -> 148 -> -32

36 -> 182 -> 149 -> -73

36 -> 182 -> 150 -> -91

36 -> 182 -> 152 -> -36

36 -> 182 -> 153 -> -2

36 -> 182 -> 155 -> -73

36 -> 182 -> 160 -> -129

36 -> 182 -> 161 -> -78

36 -> 182 -> 162 -> -133

36 -> 182 -> 163 -> -8

36 -> 182 -> 168 -> -73

36 -> 182 -> 168 -> -102

36 -> 182 -> 169 -> -99

36 -> 182 -> 170 -> -138

36 -> 182 -> 171 -> -13

36 -> 182 -> 172 -> -58

36 -> 182 -> 175 -> -32

36 -> 182 -> 175 -> -34

36 -> 182 -> 176 -> -33

36 -> 182 -> 177 -> -106

36 -> 182 -> 179 -> -22

36 -> 182 -> 179 -> -147

36 -> 182 -> 181 -> -84

36 -> 182 -> 186 -> -99

36 -> 182 -> 188 -> -24

36 -> 182 -> 189 -> -73

36 -> 182 -> -73

36 -> 182 -> -73 -> -1

36 -> 182 -> -73 -> -3

36 -> 182 -> -73 -> -6

36 -> 182 -> -73 -> -17

36 -> 182 -> -73 -> -19

36 -> 182 -> -73 -> -20

36 -> 182 -> -73 -> -22

36 -> 182 -> -73 -> -26

36 -> 182 -> -73 -> -27

36 -> 182 -> -73 -> -28

36 -> 182 -> -73 -> -29

36 -> 182 -> -73 -> -30

36 -> 182 -> -73 -> -32

36 -> 182 -> -73 -> -35

36 -> 182 -> -73 -> -39

36 -> 182 -> -73 -> -43

36 -> 182 -> -73 -> -48

36 -> 182 -> -73 -> -56

36 -> 182 -> -73 -> -61

36 -> 182 -> -73 -> -62

36 -> 182 -> -73 -> -63

36 -> 182 -> -73 -> -64

36 -> 182 -> -73 -> -68

36 -> 182 -> -73 -> -71

36 -> 182 -> -73 -> -78

36 -> 182 -> -73 -> -81

36 -> 182 -> -73 -> -83

36 -> 182 -> -73 -> -86

36 -> 182 -> -73 -> -88

36 -> 182 -> -73 -> -89

36 -> 182 -> -73 -> -92

36 -> 182 -> -73 -> -95

36 -> 182 -> -73 -> -98

36 -> 182 -> -73 -> -99

36 -> 182 -> -73 -> -101

36 -> 182 -> -73 -> -106

36 -> 182 -> -73 -> -107

36 -> 182 -> -73 -> -113

36 -> 182 -> -73 -> -114

36 -> 182 -> -73 -> -117

36 -> 182 -> -73 -> -118

36 -> 182 -> -73 -> -119

36 -> 182 -> -73 -> -121

36 -> 182 -> -73 -> -122

36 -> 182 -> -73 -> -124

36 -> 182 -> -73 -> -133

36 -> 182 -> -73 -> -137

36 -> 182 -> -73 -> -144

36 -> 182 -> -73 -> -145

36 -> 182 -> -73 -> -146

36 -> 182 -> -73 -> -147

36 -> 182 -> -73 -> -150

36 -> 182 -> -73 -> -154

36 -> 182 -> -73 -> -155

36 -> 182 -> -73 -> -156

36 -> 186 -> 3 -> -32

36 -> 186 -> 3 -> -42

36 -> 186 -> 4 -> -101

36 -> 186 -> 6 -> -73

36 -> 186 -> 7 -> -54

36 -> 186 -> 9 -> -6

36 -> 186 -> 14 -> -54

36 -> 186 -> 15 -> -68

36 -> 186 -> 19 -> -73

36 -> 186 -> 21 -> -73

36 -> 186 -> 21 -> -101

36 -> 186 -> 21 -> -154

36 -> 186 -> 22 -> -152

36 -> 186 -> 24 -> -49

36 -> 186 -> 25 -> -132

36 -> 186 -> 27 -> -19

36 -> 186 -> 27 -> -20

36 -> 186 -> 27 -> -32

36 -> 186 -> 27 -> -62

36 -> 186 -> 27 -> -68

36 -> 186 -> 27 -> -73

36 -> 186 -> 27 -> -81

36 -> 186 -> 27 -> -98

36 -> 186 -> 27 -> -99

36 -> 186 -> 27 -> -101

36 -> 186 -> 27 -> -107

36 -> 186 -> 27 -> -124

36 -> 186 -> 27 -> -133

36 -> 186 -> 27 -> -145

36 -> 186 -> 28 -> -81

36 -> 186 -> 29 -> -133

36 -> 186 -> 30 -> -1

36 -> 186 -> 30 -> -3

36 -> 186 -> 30 -> -32

36 -> 186 -> 30 -> -39

36 -> 186 -> 30 -> -63

36 -> 186 -> 30 -> -73

36 -> 186 -> 30 -> -81

36 -> 186 -> 30 -> -99

36 -> 186 -> 30 -> -101

36 -> 186 -> 30 -> -106

36 -> 186 -> 30 -> -117

36 -> 186 -> 30 -> -133

36 -> 186 -> 30 -> -137

36 -> 186 -> 30 -> -150

36 -> 186 -> 30 -> -156

36 -> 186 -> 32 -> -9

36 -> 186 -> 33 -> -145

36 -> 186 -> 34 -> -32

36 -> 186 -> 34 -> -71

36 -> 186 -> 34 -> -113

36 -> 186 -> 37 -> -73

36 -> 186 -> 38 -> -55

36 -> 186 -> 38 -> -56

36 -> 186 -> 38 -> -105

36 -> 186 -> 39 -> -101

36 -> 186 -> 40 -> -73

36 -> 186 -> 41 -> -6

36 -> 186 -> 42 -> -133

36 -> 186 -> 43 -> -73

36 -> 186 -> 43 -> -99

36 -> 186 -> 43 -> -101

36 -> 186 -> 45 -> -73

36 -> 186 -> 48 -> -99

36 -> 186 -> 49 -> -15

36 -> 186 -> 55 -> -12

36 -> 186 -> 60 -> -64

36 -> 186 -> 61 -> -27

36 -> 186 -> 62 -> -99

36 -> 186 -> 63 -> -32

36 -> 186 -> 64 -> -10

36 -> 186 -> 65 -> -4

36 -> 186 -> 66 -> -136

36 -> 186 -> 67 -> -135

36 -> 186 -> 68 -> -73

36 -> 186 -> 69 -> -77

36 -> 186 -> 73 -> -32

36 -> 186 -> 74 -> -112

36 -> 186 -> 75 -> -1

36 -> 186 -> 75 -> -29

36 -> 186 -> 76 -> -81

36 -> 186 -> 77 -> -73

36 -> 186 -> 78 -> -73

36 -> 186 -> 79 -> -73

36 -> 186 -> 84 -> -73

36 -> 186 -> 87 -> -73

36 -> 186 -> 88 -> -83

36 -> 186 -> 91 -> -101

36 -> 186 -> 92 -> -133

36 -> 186 -> 97 -> -15

36 -> 186 -> 99 -> -73

36 -> 186 -> 103 -> -78

36 -> 186 -> 105 -> -32

36 -> 186 -> 105 -> -146

36 -> 186 -> 107 -> -73

36 -> 186 -> 107 -> -99

36 -> 186 -> 108 -> -80

36 -> 186 -> 109 -> -20

36 -> 186 -> 109 -> -73

36 -> 186 -> 109 -> -99

36 -> 186 -> 109 -> -101

36 -> 186 -> 110 -> -56

36 -> 186 -> 113 -> -73

36 -> 186 -> 115 -> -17

36 -> 186 -> 115 -> -32

36 -> 186 -> 115 -> -68

36 -> 186 -> 115 -> -73

36 -> 186 -> 115 -> -92

36 -> 186 -> 116 -> -73

36 -> 186 -> 117 -> -73

36 -> 186 -> 117 -> -101

36 -> 186 -> 118 -> -73

36 -> 186 -> 120 -> -133

36 -> 186 -> 122 -> -56

36 -> 186 -> 123 -> -73

36 -> 186 -> 128 -> -32

36 -> 186 -> 128 -> -95

36 -> 186 -> 129 -> -32

36 -> 186 -> 130 -> -73

36 -> 186 -> 136 -> -20

36 -> 186 -> 138 -> -20

36 -> 186 -> 138 -> -61

36 -> 186 -> 138 -> -73

36 -> 186 -> 138 -> -98

36 -> 186 -> 138 -> -133

36 -> 186 -> 139 -> -15

36 -> 186 -> 140 -> -99

36 -> 186 -> 140 -> -133

36 -> 186 -> 142 -> -154

36 -> 186 -> 146 -> -20

36 -> 186 -> 148 -> -32

36 -> 186 -> 149 -> -73

36 -> 186 -> 150 -> -91

36 -> 186 -> 153 -> -2

36 -> 186 -> 157 -> -26

36 -> 186 -> 157 -> -28

36 -> 186 -> 157 -> -32

36 -> 186 -> 157 -> -68

36 -> 186 -> 157 -> -86

36 -> 186 -> 157 -> -99

36 -> 186 -> 157 -> -101

36 -> 186 -> 157 -> -118

36 -> 186 -> 157 -> -119

36 -> 186 -> 157 -> -120

36 -> 186 -> 157 -> -121

36 -> 186 -> 157 -> -122

36 -> 186 -> 157 -> -133

36 -> 186 -> 160 -> -129

36 -> 186 -> 161 -> -78

36 -> 186 -> 162 -> -133

36 -> 186 -> 163 -> -8

36 -> 186 -> 170 -> -138

36 -> 186 -> 171 -> -13

36 -> 186 -> 172 -> -58

36 -> 186 -> 176 -> -33

36 -> 186 -> 177 -> -106

36 -> 186 -> 179 -> -22

36 -> 186 -> 179 -> -147

36 -> 186 -> 180 -> -20

36 -> 186 -> 181 -> -84

36 -> 186 -> 182 -> -73

36 -> 186 -> 188 -> -24

36 -> 186 -> 189 -> -73

36 -> 186 -> -99

36 -> 186 -> -99 -> -1

36 -> 186 -> -99 -> -3

36 -> 186 -> -99 -> -6

36 -> 186 -> -99 -> -17

36 -> 186 -> -99 -> -18

36 -> 186 -> -99 -> -19

36 -> 186 -> -99 -> -20

36 -> 186 -> -99 -> -22

36 -> 186 -> -99 -> -26

36 -> 186 -> -99 -> -27

36 -> 186 -> -99 -> -28

36 -> 186 -> -99 -> -29

36 -> 186 -> -99 -> -30

36 -> 186 -> -99 -> -32

36 -> 186 -> -99 -> -35

36 -> 186 -> -99 -> -36

36 -> 186 -> -99 -> -39

36 -> 186 -> -99 -> -41

36 -> 186 -> -99 -> -43

36 -> 186 -> -99 -> -48

36 -> 186 -> -99 -> -51

36 -> 186 -> -99 -> -56

36 -> 186 -> -99 -> -61

36 -> 186 -> -99 -> -62

36 -> 186 -> -99 -> -63

36 -> 186 -> -99 -> -64

36 -> 186 -> -99 -> -68

36 -> 186 -> -99 -> -73

36 -> 186 -> -99 -> -78

36 -> 186 -> -99 -> -81

36 -> 186 -> -99 -> -83

36 -> 186 -> -99 -> -86

36 -> 186 -> -99 -> -88

36 -> 186 -> -99 -> -89

36 -> 186 -> -99 -> -92

36 -> 186 -> -99 -> -95

36 -> 186 -> -99 -> -97

36 -> 186 -> -99 -> -98

36 -> 186 -> -99 -> -100

36 -> 186 -> -99 -> -101

36 -> 186 -> -99 -> -106

36 -> 186 -> -99 -> -107

36 -> 186 -> -99 -> -109

36 -> 186 -> -99 -> -111

36 -> 186 -> -99 -> -114

36 -> 186 -> -99 -> -117

36 -> 186 -> -99 -> -118

36 -> 186 -> -99 -> -119

36 -> 186 -> -99 -> -121

36 -> 186 -> -99 -> -122

36 -> 186 -> -99 -> -124

36 -> 186 -> -99 -> -127

36 -> 186 -> -99 -> -133

36 -> 186 -> -99 -> -137

36 -> 186 -> -99 -> -144

36 -> 186 -> -99 -> -145

36 -> 186 -> -99 -> -146

36 -> 186 -> -99 -> -147

36 -> 186 -> -99 -> -150

36 -> 186 -> -99 -> -151

36 -> 186 -> -99 -> -154

36 -> 186 -> -99 -> -155

36 -> 186 -> -99 -> -156

36 -> 189 -> 2 -> -32

36 -> 189 -> 4 -> -101

36 -> 189 -> 6 -> -73

36 -> 189 -> 7 -> -54

36 -> 189 -> 9 -> -6

36 -> 189 -> 12 -> -32

36 -> 189 -> 13 -> -32

36 -> 189 -> 13 -> -73

36 -> 189 -> 14 -> -54

36 -> 189 -> 15 -> -68

36 -> 189 -> 16 -> -5

36 -> 189 -> 17 -> -32

36 -> 189 -> 18 -> -73

36 -> 189 -> 18 -> -89

36 -> 189 -> 19 -> -73

36 -> 189 -> 21 -> -73

36 -> 189 -> 21 -> -101

36 -> 189 -> 21 -> -154

36 -> 189 -> 22 -> -152

36 -> 189 -> 24 -> -49

36 -> 189 -> 25 -> -132

36 -> 189 -> 26 -> -14

36 -> 189 -> 28 -> -81

36 -> 189 -> 29 -> -133

36 -> 189 -> 31 -> -1

36 -> 189 -> 32 -> -9

36 -> 189 -> 33 -> -145

36 -> 189 -> 37 -> -73

36 -> 189 -> 39 -> -101

36 -> 189 -> 40 -> -73

36 -> 189 -> 41 -> -6

36 -> 189 -> 42 -> -133

36 -> 189 -> 43 -> -73

36 -> 189 -> 43 -> -99

36 -> 189 -> 43 -> -101

36 -> 189 -> 45 -> -73

36 -> 189 -> 46 -> -32

36 -> 189 -> 48 -> -99

36 -> 189 -> 49 -> -15

36 -> 189 -> 50 -> -54

36 -> 189 -> 51 -> -2

36 -> 189 -> 52 -> -32

36 -> 189 -> 53 -> -133

36 -> 189 -> 54 -> -53

36 -> 189 -> 55 -> -12

36 -> 189 -> 56 -> -115

36 -> 189 -> 57 -> -103

36 -> 189 -> 58 -> -90

36 -> 189 -> 59 -> -148

36 -> 189 -> 60 -> -64

36 -> 189 -> 61 -> -27

36 -> 189 -> 62 -> -99

36 -> 189 -> 63 -> -32

36 -> 189 -> 64 -> -10

36 -> 189 -> 65 -> -4

36 -> 189 -> 66 -> -136

36 -> 189 -> 67 -> -135

36 -> 189 -> 68 -> -73

36 -> 189 -> 69 -> -77

36 -> 189 -> 70 -> -52

36 -> 189 -> 71 -> -32

36 -> 189 -> 73 -> -32

36 -> 189 -> 74 -> -112

36 -> 189 -> 76 -> -81

36 -> 189 -> 77 -> -73

36 -> 189 -> 78 -> -73

36 -> 189 -> 79 -> -73

36 -> 189 -> 80 -> -32

36 -> 189 -> 81 -> -73

36 -> 189 -> 83 -> -125

36 -> 189 -> 84 -> -73

36 -> 189 -> 85 -> -12

36 -> 189 -> 87 -> -73

36 -> 189 -> 88 -> -83

36 -> 189 -> 90 -> -103

36 -> 189 -> 91 -> -101

36 -> 189 -> 92 -> -133

36 -> 189 -> 93 -> -145

36 -> 189 -> 94 -> -13

36 -> 189 -> 96 -> -73

36 -> 189 -> 97 -> -15

36 -> 189 -> 99 -> -73

36 -> 189 -> 101 -> -64

36 -> 189 -> 102 -> -116

36 -> 189 -> 103 -> -78

36 -> 189 -> 104 -> -2

36 -> 189 -> 106 -> -65

36 -> 189 -> 107 -> -73

36 -> 189 -> 107 -> -99

36 -> 189 -> 108 -> -80

36 -> 189 -> 110 -> -56

36 -> 189 -> 112 -> -54

36 -> 189 -> 113 -> -73

36 -> 189 -> 114 -> -73

36 -> 189 -> 116 -> -73

36 -> 189 -> 117 -> -73

36 -> 189 -> 117 -> -101

36 -> 189 -> 118 -> -73

36 -> 189 -> 120 -> -133

36 -> 189 -> 122 -> -56

36 -> 189 -> 123 -> -73

36 -> 189 -> 125 -> -104

36 -> 189 -> 126 -> -59

36 -> 189 -> 127 -> -93

36 -> 189 -> 129 -> -32

36 -> 189 -> 130 -> -73

36 -> 189 -> 132 -> -32

36 -> 189 -> 133 -> -101

36 -> 189 -> 134 -> -99

36 -> 189 -> 135 -> -21

36 -> 189 -> 135 -> -73

36 -> 189 -> 135 -> -139

36 -> 189 -> 136 -> -20

36 -> 189 -> 139 -> -15

36 -> 189 -> 142 -> -154

36 -> 189 -> 143 -> -50

36 -> 189 -> 145 -> -125

36 -> 189 -> 146 -> -20

36 -> 189 -> 147 -> -32

36 -> 189 -> 148 -> -32

36 -> 189 -> 149 -> -73

36 -> 189 -> 150 -> -91

36 -> 189 -> 151 -> -101

36 -> 189 -> 152 -> -36

36 -> 189 -> 153 -> -2

36 -> 189 -> 155 -> -73

36 -> 189 -> 158 -> -72

36 -> 189 -> 160 -> -129

36 -> 189 -> 161 -> -78

36 -> 189 -> 162 -> -133

36 -> 189 -> 163 -> -8

36 -> 189 -> 164 -> -134

36 -> 189 -> 165 -> -54

36 -> 189 -> 168 -> -73

36 -> 189 -> 168 -> -102

36 -> 189 -> 169 -> -99

36 -> 189 -> 170 -> -138

36 -> 189 -> 171 -> -13

36 -> 189 -> 172 -> -58

36 -> 189 -> 173 -> -154

36 -> 189 -> 174 -> -114

36 -> 189 -> 176 -> -33

36 -> 189 -> 177 -> -106

36 -> 189 -> 178 -> -69

36 -> 189 -> 180 -> -20

36 -> 189 -> 181 -> -84

36 -> 189 -> 182 -> -73

36 -> 189 -> 186 -> -99

36 -> 189 -> 187 -> -53

36 -> 189 -> 188 -> -24

36 -> 189 -> -73

36 -> 189 -> -73 -> -1

36 -> 189 -> -73 -> -3

36 -> 189 -> -73 -> -6

36 -> 189 -> -73 -> -17

36 -> 189 -> -73 -> -19

36 -> 189 -> -73 -> -20

36 -> 189 -> -73 -> -22

36 -> 189 -> -73 -> -26

36 -> 189 -> -73 -> -27

36 -> 189 -> -73 -> -28

36 -> 189 -> -73 -> -29

36 -> 189 -> -73 -> -30

36 -> 189 -> -73 -> -32

36 -> 189 -> -73 -> -35

36 -> 189 -> -73 -> -39

36 -> 189 -> -73 -> -43

36 -> 189 -> -73 -> -48

36 -> 189 -> -73 -> -56

36 -> 189 -> -73 -> -61

36 -> 189 -> -73 -> -62

36 -> 189 -> -73 -> -63

36 -> 189 -> -73 -> -64

36 -> 189 -> -73 -> -68

36 -> 189 -> -73 -> -71

36 -> 189 -> -73 -> -78

36 -> 189 -> -73 -> -81

36 -> 189 -> -73 -> -83

36 -> 189 -> -73 -> -86

36 -> 189 -> -73 -> -88

36 -> 189 -> -73 -> -89

36 -> 189 -> -73 -> -92

36 -> 189 -> -73 -> -95

36 -> 189 -> -73 -> -98

36 -> 189 -> -73 -> -99

36 -> 189 -> -73 -> -101

36 -> 189 -> -73 -> -106

36 -> 189 -> -73 -> -107

36 -> 189 -> -73 -> -113

36 -> 189 -> -73 -> -114

36 -> 189 -> -73 -> -117

36 -> 189 -> -73 -> -118

36 -> 189 -> -73 -> -119

36 -> 189 -> -73 -> -121

36 -> 189 -> -73 -> -122

36 -> 189 -> -73 -> -124

36 -> 189 -> -73 -> -133

36 -> 189 -> -73 -> -137

36 -> 189 -> -73 -> -144

36 -> 189 -> -73 -> -145

36 -> 189 -> -73 -> -146

36 -> 189 -> -73 -> -147

36 -> 189 -> -73 -> -150

36 -> 189 -> -73 -> -154

36 -> 189 -> -73 -> -155

36 -> 189 -> -73 -> -156

36 -> -20

36 -> -20 -> 8 -> -88

36 -> -20 -> 10 -> -16

36 -> -20 -> 10 -> -32

36 -> -20 -> 10 -> -70

36 -> -20 -> 10 -> -74

36 -> -20 -> 10 -> -75

36 -> -20 -> 10 -> -140

36 -> -20 -> 10 -> -143

36 -> -20 -> 27 -> -19

36 -> -20 -> 27 -> -32

36 -> -20 -> 27 -> -62

36 -> -20 -> 27 -> -68

36 -> -20 -> 27 -> -73

36 -> -20 -> 27 -> -81

36 -> -20 -> 27 -> -98

36 -> -20 -> 27 -> -99

36 -> -20 -> 27 -> -101

36 -> -20 -> 27 -> -107

36 -> -20 -> 27 -> -124

36 -> -20 -> 27 -> -133

36 -> -20 -> 27 -> -145

36 -> -20 -> 109 -> -73

36 -> -20 -> 109 -> -99

36 -> -20 -> 109 -> -101

36 -> -20 -> 138 -> -61

36 -> -20 -> 138 -> -73

36 -> -20 -> 138 -> -98

36 -> -20 -> 138 -> -133

36 -> -20 -> -1

36 -> -20 -> -1 -> -2

36 -> -20 -> -1 -> -3

36 -> -20 -> -1 -> -6

36 -> -20 -> -1 -> -12

36 -> -20 -> -1 -> -19

36 -> -20 -> -1 -> -22

36 -> -20 -> -1 -> -26

36 -> -20 -> -1 -> -27

36 -> -20 -> -1 -> -28

36 -> -20 -> -1 -> -29

36 -> -20 -> -1 -> -30

36 -> -20 -> -1 -> -32

36 -> -20 -> -1 -> -35

36 -> -20 -> -1 -> -36

36 -> -20 -> -1 -> -39

36 -> -20 -> -1 -> -42

36 -> -20 -> -1 -> -48

36 -> -20 -> -1 -> -51

36 -> -20 -> -1 -> -55

36 -> -20 -> -1 -> -56

36 -> -20 -> -1 -> -62

36 -> -20 -> -1 -> -63

36 -> -20 -> -1 -> -64

36 -> -20 -> -1 -> -68

36 -> -20 -> -1 -> -71

36 -> -20 -> -1 -> -73

36 -> -20 -> -1 -> -76

36 -> -20 -> -1 -> -78

36 -> -20 -> -1 -> -80

36 -> -20 -> -1 -> -81

36 -> -20 -> -1 -> -83

36 -> -20 -> -1 -> -86

36 -> -20 -> -1 -> -88

36 -> -20 -> -1 -> -89

36 -> -20 -> -1 -> -95

36 -> -20 -> -1 -> -98

36 -> -20 -> -1 -> -99

36 -> -20 -> -1 -> -100

36 -> -20 -> -1 -> -101

36 -> -20 -> -1 -> -105

36 -> -20 -> -1 -> -106

36 -> -20 -> -1 -> -107

36 -> -20 -> -1 -> -112

36 -> -20 -> -1 -> -113

36 -> -20 -> -1 -> -114

36 -> -20 -> -1 -> -116

36 -> -20 -> -1 -> -117

36 -> -20 -> -1 -> -118

36 -> -20 -> -1 -> -119

36 -> -20 -> -1 -> -121

36 -> -20 -> -1 -> -122

36 -> -20 -> -1 -> -124

36 -> -20 -> -1 -> -127

36 -> -20 -> -1 -> -133

36 -> -20 -> -1 -> -137

36 -> -20 -> -1 -> -145

36 -> -20 -> -1 -> -146

36 -> -20 -> -1 -> -147

36 -> -20 -> -1 -> -150

36 -> -20 -> -1 -> -154

36 -> -20 -> -1 -> -155

36 -> -20 -> -1 -> -156

36 -> -20 -> -3

36 -> -20 -> -3 -> -1

36 -> -20 -> -3 -> -4

36 -> -20 -> -3 -> -6

36 -> -20 -> -3 -> -7

36 -> -20 -> -3 -> -8

36 -> -20 -> -3 -> -9

36 -> -20 -> -3 -> -10

36 -> -20 -> -3 -> -17

36 -> -20 -> -3 -> -18

36 -> -20 -> -3 -> -19

36 -> -20 -> -3 -> -22

36 -> -20 -> -3 -> -24

36 -> -20 -> -3 -> -26

36 -> -20 -> -3 -> -27

36 -> -20 -> -3 -> -28

36 -> -20 -> -3 -> -29

36 -> -20 -> -3 -> -30

36 -> -20 -> -3 -> -31

36 -> -20 -> -3 -> -32

36 -> -20 -> -3 -> -33

36 -> -20 -> -3 -> -35

36 -> -20 -> -3 -> -36

36 -> -20 -> -3 -> -39

36 -> -20 -> -3 -> -42

36 -> -20 -> -3 -> -43

36 -> -20 -> -3 -> -44

36 -> -20 -> -3 -> -45

36 -> -20 -> -3 -> -46

36 -> -20 -> -3 -> -47

36 -> -20 -> -3 -> -48

36 -> -20 -> -3 -> -49

36 -> -20 -> -3 -> -51

36 -> -20 -> -3 -> -55

36 -> -20 -> -3 -> -56

36 -> -20 -> -3 -> -58

36 -> -20 -> -3 -> -60

36 -> -20 -> -3 -> -61

36 -> -20 -> -3 -> -62

36 -> -20 -> -3 -> -63

36 -> -20 -> -3 -> -64

36 -> -20 -> -3 -> -66

36 -> -20 -> -3 -> -68

36 -> -20 -> -3 -> -69

36 -> -20 -> -3 -> -73

36 -> -20 -> -3 -> -77

36 -> -20 -> -3 -> -78

36 -> -20 -> -3 -> -81

36 -> -20 -> -3 -> -83

36 -> -20 -> -3 -> -84

36 -> -20 -> -3 -> -86

36 -> -20 -> -3 -> -88

36 -> -20 -> -3 -> -89

36 -> -20 -> -3 -> -91

36 -> -20 -> -3 -> -92

36 -> -20 -> -3 -> -95

36 -> -20 -> -3 -> -96

36 -> -20 -> -3 -> -97

36 -> -20 -> -3 -> -98

36 -> -20 -> -3 -> -99

36 -> -20 -> -3 -> -100

36 -> -20 -> -3 -> -101

36 -> -20 -> -3 -> -105

36 -> -20 -> -3 -> -106

36 -> -20 -> -3 -> -107

36 -> -20 -> -3 -> -110

36 -> -20 -> -3 -> -114

36 -> -20 -> -3 -> -116

36 -> -20 -> -3 -> -117

36 -> -20 -> -3 -> -118

36 -> -20 -> -3 -> -119

36 -> -20 -> -3 -> -120

36 -> -20 -> -3 -> -121

36 -> -20 -> -3 -> -122

36 -> -20 -> -3 -> -124

36 -> -20 -> -3 -> -125

36 -> -20 -> -3 -> -127

36 -> -20 -> -3 -> -130

36 -> -20 -> -3 -> -131

36 -> -20 -> -3 -> -132

36 -> -20 -> -3 -> -133

36 -> -20 -> -3 -> -134

36 -> -20 -> -3 -> -135

36 -> -20 -> -3 -> -136

36 -> -20 -> -3 -> -137

36 -> -20 -> -3 -> -141

36 -> -20 -> -3 -> -144

36 -> -20 -> -3 -> -145

36 -> -20 -> -3 -> -146

36 -> -20 -> -3 -> -147

36 -> -20 -> -3 -> -150

36 -> -20 -> -3 -> -154

36 -> -20 -> -3 -> -155

36 -> -20 -> -3 -> -156

36 -> -20 -> -6

36 -> -20 -> -6 -> -1

36 -> -20 -> -6 -> -3

36 -> -20 -> -6 -> -7

36 -> -20 -> -6 -> -19

36 -> -20 -> -6 -> -29

36 -> -20 -> -6 -> -39

36 -> -20 -> -6 -> -48

36 -> -20 -> -6 -> -51

36 -> -20 -> -6 -> -62

36 -> -20 -> -6 -> -63

36 -> -20 -> -6 -> -64

36 -> -20 -> -6 -> -73

36 -> -20 -> -6 -> -78

36 -> -20 -> -6 -> -81

36 -> -20 -> -6 -> -83

36 -> -20 -> -6 -> -86

36 -> -20 -> -6 -> -89

36 -> -20 -> -6 -> -95

36 -> -20 -> -6 -> -98

36 -> -20 -> -6 -> -99

36 -> -20 -> -6 -> -100

36 -> -20 -> -6 -> -101

36 -> -20 -> -6 -> -106

36 -> -20 -> -6 -> -107

36 -> -20 -> -6 -> -110

36 -> -20 -> -6 -> -114

36 -> -20 -> -6 -> -117

36 -> -20 -> -6 -> -124

36 -> -20 -> -6 -> -127

36 -> -20 -> -6 -> -130

36 -> -20 -> -6 -> -137

36 -> -20 -> -6 -> -150

36 -> -20 -> -6 -> -154

36 -> -20 -> -6 -> -155

36 -> -20 -> -6 -> -156

36 -> -20 -> -16

36 -> -20 -> -16 -> -4

36 -> -20 -> -16 -> -8

36 -> -20 -> -16 -> -9

36 -> -20 -> -16 -> -10

36 -> -20 -> -16 -> -11

36 -> -20 -> -16 -> -13

36 -> -20 -> -16 -> -24

36 -> -20 -> -16 -> -31

36 -> -20 -> -16 -> -33

36 -> -20 -> -16 -> -34

36 -> -20 -> -16 -> -44

36 -> -20 -> -16 -> -45

36 -> -20 -> -16 -> -46

36 -> -20 -> -16 -> -47

36 -> -20 -> -16 -> -49

36 -> -20 -> -16 -> -52

36 -> -20 -> -16 -> -53

36 -> -20 -> -16 -> -58

36 -> -20 -> -16 -> -59

36 -> -20 -> -16 -> -60

36 -> -20 -> -16 -> -66

36 -> -20 -> -16 -> -67

36 -> -20 -> -16 -> -69

36 -> -20 -> -16 -> -70

36 -> -20 -> -16 -> -74

36 -> -20 -> -16 -> -75

36 -> -20 -> -16 -> -77

36 -> -20 -> -16 -> -80

36 -> -20 -> -16 -> -82

36 -> -20 -> -16 -> -84

36 -> -20 -> -16 -> -85

36 -> -20 -> -16 -> -90

36 -> -20 -> -16 -> -91

36 -> -20 -> -16 -> -96

36 -> -20 -> -16 -> -112

36 -> -20 -> -16 -> -120

36 -> -20 -> -16 -> -125

36 -> -20 -> -16 -> -131

36 -> -20 -> -16 -> -132

36 -> -20 -> -16 -> -135

36 -> -20 -> -16 -> -136

36 -> -20 -> -16 -> -138

36 -> -20 -> -16 -> -140

36 -> -20 -> -16 -> -141

36 -> -20 -> -16 -> -143

36 -> -20 -> -16 -> -152

36 -> -20 -> -16 -> -153

36 -> -20 -> -17

36 -> -20 -> -17 -> -3

36 -> -20 -> -17 -> -4

36 -> -20 -> -17 -> -7

36 -> -20 -> -17 -> -8

36 -> -20 -> -17 -> -9

36 -> -20 -> -17 -> -10

36 -> -20 -> -17 -> -18

36 -> -20 -> -17 -> -19

36 -> -20 -> -17 -> -22

36 -> -20 -> -17 -> -24

36 -> -20 -> -17 -> -30

36 -> -20 -> -17 -> -31

36 -> -20 -> -17 -> -32

36 -> -20 -> -17 -> -33

36 -> -20 -> -17 -> -35

36 -> -20 -> -17 -> -36

36 -> -20 -> -17 -> -39

36 -> -20 -> -17 -> -41

36 -> -20 -> -17 -> -43

36 -> -20 -> -17 -> -44

36 -> -20 -> -17 -> -45

36 -> -20 -> -17 -> -46

36 -> -20 -> -17 -> -47

36 -> -20 -> -17 -> -49

36 -> -20 -> -17 -> -58

36 -> -20 -> -17 -> -60

36 -> -20 -> -17 -> -61

36 -> -20 -> -17 -> -62

36 -> -20 -> -17 -> -63

36 -> -20 -> -17 -> -64

36 -> -20 -> -17 -> -66

36 -> -20 -> -17 -> -68

36 -> -20 -> -17 -> -69

36 -> -20 -> -17 -> -73

36 -> -20 -> -17 -> -77

36 -> -20 -> -17 -> -78

36 -> -20 -> -17 -> -81

36 -> -20 -> -17 -> -84

36 -> -20 -> -17 -> -88

36 -> -20 -> -17 -> -91

36 -> -20 -> -17 -> -92

36 -> -20 -> -17 -> -95

36 -> -20 -> -17 -> -96

36 -> -20 -> -17 -> -97

36 -> -20 -> -17 -> -98

36 -> -20 -> -17 -> -99

36 -> -20 -> -17 -> -101

36 -> -20 -> -17 -> -107

36 -> -20 -> -17 -> -109

36 -> -20 -> -17 -> -110

36 -> -20 -> -17 -> -111

36 -> -20 -> -17 -> -116

36 -> -20 -> -17 -> -117

36 -> -20 -> -17 -> -120

36 -> -20 -> -17 -> -124

36 -> -20 -> -17 -> -128

36 -> -20 -> -17 -> -129

36 -> -20 -> -17 -> -130

36 -> -20 -> -17 -> -131

36 -> -20 -> -17 -> -132

36 -> -20 -> -17 -> -134

36 -> -20 -> -17 -> -135

36 -> -20 -> -17 -> -136

36 -> -20 -> -17 -> -137

36 -> -20 -> -17 -> -141

36 -> -20 -> -17 -> -144

36 -> -20 -> -17 -> -147

36 -> -20 -> -17 -> -150

36 -> -20 -> -17 -> -151

36 -> -20 -> -17 -> -154

36 -> -20 -> -17 -> -156

36 -> -20 -> -19

36 -> -20 -> -19 -> -1

36 -> -20 -> -19 -> -3

36 -> -20 -> -19 -> -4

36 -> -20 -> -19 -> -5

36 -> -20 -> -19 -> -6

36 -> -20 -> -19 -> -7

36 -> -20 -> -19 -> -8

36 -> -20 -> -19 -> -9

36 -> -20 -> -19 -> -10

36 -> -20 -> -19 -> -17

36 -> -20 -> -19 -> -18

36 -> -20 -> -19 -> -22

36 -> -20 -> -19 -> -24

36 -> -20 -> -19 -> -26

36 -> -20 -> -19 -> -27

36 -> -20 -> -19 -> -28

36 -> -20 -> -19 -> -29

36 -> -20 -> -19 -> -30

36 -> -20 -> -19 -> -31

36 -> -20 -> -19 -> -32

36 -> -20 -> -19 -> -33

36 -> -20 -> -19 -> -35

36 -> -20 -> -19 -> -36

36 -> -20 -> -19 -> -39

36 -> -20 -> -19 -> -42

36 -> -20 -> -19 -> -43

36 -> -20 -> -19 -> -44

36 -> -20 -> -19 -> -45

36 -> -20 -> -19 -> -46

36 -> -20 -> -19 -> -47

36 -> -20 -> -19 -> -48

36 -> -20 -> -19 -> -49

36 -> -20 -> -19 -> -55

36 -> -20 -> -19 -> -56

36 -> -20 -> -19 -> -58

36 -> -20 -> -19 -> -60

36 -> -20 -> -19 -> -61

36 -> -20 -> -19 -> -62

36 -> -20 -> -19 -> -63

36 -> -20 -> -19 -> -64

36 -> -20 -> -19 -> -66

36 -> -20 -> -19 -> -68

36 -> -20 -> -19 -> -69

36 -> -20 -> -19 -> -71

36 -> -20 -> -19 -> -73

36 -> -20 -> -19 -> -77

36 -> -20 -> -19 -> -78

36 -> -20 -> -19 -> -81

36 -> -20 -> -19 -> -83

36 -> -20 -> -19 -> -84

36 -> -20 -> -19 -> -86

36 -> -20 -> -19 -> -88

36 -> -20 -> -19 -> -89

36 -> -20 -> -19 -> -91

36 -> -20 -> -19 -> -92

36 -> -20 -> -19 -> -93

36 -> -20 -> -19 -> -95

36 -> -20 -> -19 -> -96

36 -> -20 -> -19 -> -97

36 -> -20 -> -19 -> -98

36 -> -20 -> -19 -> -99

36 -> -20 -> -19 -> -101

36 -> -20 -> -19 -> -105

36 -> -20 -> -19 -> -106

36 -> -20 -> -19 -> -107

36 -> -20 -> -19 -> -110

36 -> -20 -> -19 -> -113

36 -> -20 -> -19 -> -114

36 -> -20 -> -19 -> -116

36 -> -20 -> -19 -> -117

36 -> -20 -> -19 -> -118

36 -> -20 -> -19 -> -119

36 -> -20 -> -19 -> -120

36 -> -20 -> -19 -> -121

36 -> -20 -> -19 -> -122

36 -> -20 -> -19 -> -124

36 -> -20 -> -19 -> -130

36 -> -20 -> -19 -> -131

36 -> -20 -> -19 -> -132

36 -> -20 -> -19 -> -133

36 -> -20 -> -19 -> -135

36 -> -20 -> -19 -> -136

36 -> -20 -> -19 -> -137

36 -> -20 -> -19 -> -141

36 -> -20 -> -19 -> -144

36 -> -20 -> -19 -> -145

36 -> -20 -> -19 -> -146

36 -> -20 -> -19 -> -147

36 -> -20 -> -19 -> -150

36 -> -20 -> -19 -> -154

36 -> -20 -> -19 -> -155

36 -> -20 -> -19 -> -156

36 -> -20 -> -22

36 -> -20 -> -22 -> -1

36 -> -20 -> -22 -> -3

36 -> -20 -> -22 -> -4

36 -> -20 -> -22 -> -7

36 -> -20 -> -22 -> -8

36 -> -20 -> -22 -> -9

36 -> -20 -> -22 -> -10

36 -> -20 -> -22 -> -17

36 -> -20 -> -22 -> -19

36 -> -20 -> -22 -> -24

36 -> -20 -> -22 -> -26

36 -> -20 -> -22 -> -27

36 -> -20 -> -22 -> -28

36 -> -20 -> -22 -> -29

36 -> -20 -> -22 -> -31

36 -> -20 -> -22 -> -32

36 -> -20 -> -22 -> -33

36 -> -20 -> -22 -> -36

36 -> -20 -> -22 -> -39

36 -> -20 -> -22 -> -42

36 -> -20 -> -22 -> -43

36 -> -20 -> -22 -> -44

36 -> -20 -> -22 -> -45

36 -> -20 -> -22 -> -46

36 -> -20 -> -22 -> -47

36 -> -20 -> -22 -> -49

36 -> -20 -> -22 -> -55

36 -> -20 -> -22 -> -56

36 -> -20 -> -22 -> -58

36 -> -20 -> -22 -> -60

36 -> -20 -> -22 -> -61

36 -> -20 -> -22 -> -62

36 -> -20 -> -22 -> -63

36 -> -20 -> -22 -> -64

36 -> -20 -> -22 -> -66

36 -> -20 -> -22 -> -68

36 -> -20 -> -22 -> -69

36 -> -20 -> -22 -> -71

36 -> -20 -> -22 -> -73

36 -> -20 -> -22 -> -77

36 -> -20 -> -22 -> -78

36 -> -20 -> -22 -> -81

36 -> -20 -> -22 -> -84

36 -> -20 -> -22 -> -86

36 -> -20 -> -22 -> -88

36 -> -20 -> -22 -> -91

36 -> -20 -> -22 -> -92

36 -> -20 -> -22 -> -95

36 -> -20 -> -22 -> -96

36 -> -20 -> -22 -> -98

36 -> -20 -> -22 -> -99

36 -> -20 -> -22 -> -101

36 -> -20 -> -22 -> -105

36 -> -20 -> -22 -> -106

36 -> -20 -> -22 -> -107

36 -> -20 -> -22 -> -110

36 -> -20 -> -22 -> -113

36 -> -20 -> -22 -> -117

36 -> -20 -> -22 -> -118

36 -> -20 -> -22 -> -119

36 -> -20 -> -22 -> -120

36 -> -20 -> -22 -> -121

36 -> -20 -> -22 -> -122

36 -> -20 -> -22 -> -124

36 -> -20 -> -22 -> -130

36 -> -20 -> -22 -> -131

36 -> -20 -> -22 -> -132

36 -> -20 -> -22 -> -133

36 -> -20 -> -22 -> -135

36 -> -20 -> -22 -> -136

36 -> -20 -> -22 -> -137

36 -> -20 -> -22 -> -141

36 -> -20 -> -22 -> -144

36 -> -20 -> -22 -> -145

36 -> -20 -> -22 -> -147

36 -> -20 -> -22 -> -150

36 -> -20 -> -22 -> -156

36 -> -20 -> -26

36 -> -20 -> -26 -> -1

36 -> -20 -> -26 -> -3

36 -> -20 -> -26 -> -4

36 -> -20 -> -26 -> -8

36 -> -20 -> -26 -> -9

36 -> -20 -> -26 -> -10

36 -> -20 -> -26 -> -19

36 -> -20 -> -26 -> -22

36 -> -20 -> -26 -> -24

36 -> -20 -> -26 -> -27

36 -> -20 -> -26 -> -28

36 -> -20 -> -26 -> -29

36 -> -20 -> -26 -> -31

36 -> -20 -> -26 -> -33

36 -> -20 -> -26 -> -39

36 -> -20 -> -26 -> -44

36 -> -20 -> -26 -> -45

36 -> -20 -> -26 -> -46

36 -> -20 -> -26 -> -47

36 -> -20 -> -26 -> -49

36 -> -20 -> -26 -> -58

36 -> -20 -> -26 -> -60

36 -> -20 -> -26 -> -61

36 -> -20 -> -26 -> -62

36 -> -20 -> -26 -> -63

36 -> -20 -> -26 -> -66

36 -> -20 -> -26 -> -68

36 -> -20 -> -26 -> -69

36 -> -20 -> -26 -> -73

36 -> -20 -> -26 -> -77

36 -> -20 -> -26 -> -78

36 -> -20 -> -26 -> -81

36 -> -20 -> -26 -> -84

36 -> -20 -> -26 -> -86

36 -> -20 -> -26 -> -88

36 -> -20 -> -26 -> -91

36 -> -20 -> -26 -> -96

36 -> -20 -> -26 -> -98

36 -> -20 -> -26 -> -99

36 -> -20 -> -26 -> -101

36 -> -20 -> -26 -> -107

36 -> -20 -> -26 -> -117

36 -> -20 -> -26 -> -118

36 -> -20 -> -26 -> -119

36 -> -20 -> -26 -> -120

36 -> -20 -> -26 -> -121

36 -> -20 -> -26 -> -122

36 -> -20 -> -26 -> -124

36 -> -20 -> -26 -> -131

36 -> -20 -> -26 -> -132

36 -> -20 -> -26 -> -133

36 -> -20 -> -26 -> -135

36 -> -20 -> -26 -> -136

36 -> -20 -> -26 -> -137

36 -> -20 -> -26 -> -141

36 -> -20 -> -26 -> -145

36 -> -20 -> -26 -> -147

36 -> -20 -> -26 -> -150

36 -> -20 -> -26 -> -156

36 -> -20 -> -27

36 -> -20 -> -27 -> -1

36 -> -20 -> -27 -> -3

36 -> -20 -> -27 -> -4

36 -> -20 -> -27 -> -8

36 -> -20 -> -27 -> -9

36 -> -20 -> -27 -> -10

36 -> -20 -> -27 -> -19

36 -> -20 -> -27 -> -22

36 -> -20 -> -27 -> -24

36 -> -20 -> -27 -> -26

36 -> -20 -> -27 -> -28

36 -> -20 -> -27 -> -29

36 -> -20 -> -27 -> -31

36 -> -20 -> -27 -> -33

36 -> -20 -> -27 -> -39

36 -> -20 -> -27 -> -44

36 -> -20 -> -27 -> -45

36 -> -20 -> -27 -> -46

36 -> -20 -> -27 -> -47

36 -> -20 -> -27 -> -49

36 -> -20 -> -27 -> -58

36 -> -20 -> -27 -> -60

36 -> -20 -> -27 -> -61

36 -> -20 -> -27 -> -62

36 -> -20 -> -27 -> -63

36 -> -20 -> -27 -> -66

36 -> -20 -> -27 -> -68

36 -> -20 -> -27 -> -69

36 -> -20 -> -27 -> -71

36 -> -20 -> -27 -> -73

36 -> -20 -> -27 -> -77

36 -> -20 -> -27 -> -78

36 -> -20 -> -27 -> -81

36 -> -20 -> -27 -> -84

36 -> -20 -> -27 -> -86

36 -> -20 -> -27 -> -88

36 -> -20 -> -27 -> -91

36 -> -20 -> -27 -> -95

36 -> -20 -> -27 -> -96

36 -> -20 -> -27 -> -98

36 -> -20 -> -27 -> -99

36 -> -20 -> -27 -> -106

36 -> -20 -> -27 -> -107

36 -> -20 -> -27 -> -113

36 -> -20 -> -27 -> -117

36 -> -20 -> -27 -> -118

36 -> -20 -> -27 -> -119

36 -> -20 -> -27 -> -120

36 -> -20 -> -27 -> -121

36 -> -20 -> -27 -> -122

36 -> -20 -> -27 -> -124

36 -> -20 -> -27 -> -131

36 -> -20 -> -27 -> -132

36 -> -20 -> -27 -> -133

36 -> -20 -> -27 -> -135

36 -> -20 -> -27 -> -136

36 -> -20 -> -27 -> -137

36 -> -20 -> -27 -> -141

36 -> -20 -> -27 -> -145

36 -> -20 -> -27 -> -147

36 -> -20 -> -27 -> -150

36 -> -20 -> -27 -> -156

36 -> -20 -> -28

36 -> -20 -> -28 -> -1

36 -> -20 -> -28 -> -3

36 -> -20 -> -28 -> -4

36 -> -20 -> -28 -> -8

36 -> -20 -> -28 -> -9

36 -> -20 -> -28 -> -10

36 -> -20 -> -28 -> -19

36 -> -20 -> -28 -> -22

36 -> -20 -> -28 -> -24

36 -> -20 -> -28 -> -26

36 -> -20 -> -28 -> -27

36 -> -20 -> -28 -> -29

36 -> -20 -> -28 -> -31

36 -> -20 -> -28 -> -33

36 -> -20 -> -28 -> -39

36 -> -20 -> -28 -> -44

36 -> -20 -> -28 -> -45

36 -> -20 -> -28 -> -46

36 -> -20 -> -28 -> -47

36 -> -20 -> -28 -> -49

36 -> -20 -> -28 -> -58

36 -> -20 -> -28 -> -60

36 -> -20 -> -28 -> -61

36 -> -20 -> -28 -> -62

36 -> -20 -> -28 -> -63

36 -> -20 -> -28 -> -66

36 -> -20 -> -28 -> -68

36 -> -20 -> -28 -> -69

36 -> -20 -> -28 -> -73

36 -> -20 -> -28 -> -77

36 -> -20 -> -28 -> -78

36 -> -20 -> -28 -> -81

36 -> -20 -> -28 -> -84

36 -> -20 -> -28 -> -86

36 -> -20 -> -28 -> -88

36 -> -20 -> -28 -> -91

36 -> -20 -> -28 -> -96

36 -> -20 -> -28 -> -98

36 -> -20 -> -28 -> -99

36 -> -20 -> -28 -> -101

36 -> -20 -> -28 -> -107

36 -> -20 -> -28 -> -117

36 -> -20 -> -28 -> -118

36 -> -20 -> -28 -> -119

36 -> -20 -> -28 -> -120

36 -> -20 -> -28 -> -121

36 -> -20 -> -28 -> -122

36 -> -20 -> -28 -> -124

36 -> -20 -> -28 -> -131

36 -> -20 -> -28 -> -132

36 -> -20 -> -28 -> -133

36 -> -20 -> -28 -> -135

36 -> -20 -> -28 -> -136

36 -> -20 -> -28 -> -137

36 -> -20 -> -28 -> -141

36 -> -20 -> -28 -> -145

36 -> -20 -> -28 -> -147

36 -> -20 -> -28 -> -150

36 -> -20 -> -28 -> -156

36 -> -20 -> -29

36 -> -20 -> -29 -> -1

36 -> -20 -> -29 -> -3

36 -> -20 -> -29 -> -4

36 -> -20 -> -29 -> -6

36 -> -20 -> -29 -> -8

36 -> -20 -> -29 -> -9

36 -> -20 -> -29 -> -10

36 -> -20 -> -29 -> -19

36 -> -20 -> -29 -> -22

36 -> -20 -> -29 -> -24

36 -> -20 -> -29 -> -26

36 -> -20 -> -29 -> -27

36 -> -20 -> -29 -> -28

36 -> -20 -> -29 -> -30

36 -> -20 -> -29 -> -31

36 -> -20 -> -29 -> -32

36 -> -20 -> -29 -> -33

36 -> -20 -> -29 -> -35

36 -> -20 -> -29 -> -39

36 -> -20 -> -29 -> -44

36 -> -20 -> -29 -> -45

36 -> -20 -> -29 -> -46

36 -> -20 -> -29 -> -47

36 -> -20 -> -29 -> -48

36 -> -20 -> -29 -> -49

36 -> -20 -> -29 -> -51

36 -> -20 -> -29 -> -56

36 -> -20 -> -29 -> -58

36 -> -20 -> -29 -> -60

36 -> -20 -> -29 -> -61

36 -> -20 -> -29 -> -62

36 -> -20 -> -29 -> -63

36 -> -20 -> -29 -> -64

36 -> -20 -> -29 -> -66

36 -> -20 -> -29 -> -68

36 -> -20 -> -29 -> -69

36 -> -20 -> -29 -> -73

36 -> -20 -> -29 -> -77

36 -> -20 -> -29 -> -78

36 -> -20 -> -29 -> -81

36 -> -20 -> -29 -> -83

36 -> -20 -> -29 -> -84

36 -> -20 -> -29 -> -86

36 -> -20 -> -29 -> -88

36 -> -20 -> -29 -> -89

36 -> -20 -> -29 -> -91

36 -> -20 -> -29 -> -95

36 -> -20 -> -29 -> -96

36 -> -20 -> -29 -> -98

36 -> -20 -> -29 -> -99

36 -> -20 -> -29 -> -100

36 -> -20 -> -29 -> -101

36 -> -20 -> -29 -> -106

36 -> -20 -> -29 -> -107

36 -> -20 -> -29 -> -114

36 -> -20 -> -29 -> -117

36 -> -20 -> -29 -> -118

36 -> -20 -> -29 -> -119

36 -> -20 -> -29 -> -120

36 -> -20 -> -29 -> -121

36 -> -20 -> -29 -> -122

36 -> -20 -> -29 -> -124

36 -> -20 -> -29 -> -127

36 -> -20 -> -29 -> -131

36 -> -20 -> -29 -> -132

36 -> -20 -> -29 -> -133

36 -> -20 -> -29 -> -135

36 -> -20 -> -29 -> -136

36 -> -20 -> -29 -> -137

36 -> -20 -> -29 -> -141

36 -> -20 -> -29 -> -145

36 -> -20 -> -29 -> -146

36 -> -20 -> -29 -> -147

36 -> -20 -> -29 -> -150

36 -> -20 -> -29 -> -154

36 -> -20 -> -29 -> -155

36 -> -20 -> -29 -> -156

36 -> -20 -> -32

36 -> -20 -> -32 -> -1

36 -> -20 -> -32 -> -3

36 -> -20 -> -32 -> -14

36 -> -20 -> -32 -> -17

36 -> -20 -> -32 -> -19

36 -> -20 -> -32 -> -22

36 -> -20 -> -32 -> -29

36 -> -20 -> -32 -> -30

36 -> -20 -> -32 -> -34

36 -> -20 -> -32 -> -35

36 -> -20 -> -32 -> -37

36 -> -20 -> -32 -> -38

36 -> -20 -> -32 -> -39

36 -> -20 -> -32 -> -43

36 -> -20 -> -32 -> -55

36 -> -20 -> -32 -> -56

36 -> -20 -> -32 -> -62

36 -> -20 -> -32 -> -63

36 -> -20 -> -32 -> -64

36 -> -20 -> -32 -> -68

36 -> -20 -> -32 -> -71

36 -> -20 -> -32 -> -73

36 -> -20 -> -32 -> -78

36 -> -20 -> -32 -> -81

36 -> -20 -> -32 -> -86

36 -> -20 -> -32 -> -92

36 -> -20 -> -32 -> -94

36 -> -20 -> -32 -> -95

36 -> -20 -> -32 -> -98

36 -> -20 -> -32 -> -99

36 -> -20 -> -32 -> -101

36 -> -20 -> -32 -> -103

36 -> -20 -> -32 -> -105

36 -> -20 -> -32 -> -106

36 -> -20 -> -32 -> -107

36 -> -20 -> -32 -> -113

36 -> -20 -> -32 -> -117

36 -> -20 -> -32 -> -124

36 -> -20 -> -32 -> -125

36 -> -20 -> -32 -> -133

36 -> -20 -> -32 -> -137

36 -> -20 -> -32 -> -144

36 -> -20 -> -32 -> -145

36 -> -20 -> -32 -> -146

36 -> -20 -> -32 -> -147

36 -> -20 -> -32 -> -150

36 -> -20 -> -32 -> -154

36 -> -20 -> -32 -> -156

36 -> -20 -> -39

36 -> -20 -> -39 -> -1

36 -> -20 -> -39 -> -3

36 -> -20 -> -39 -> -4

36 -> -20 -> -39 -> -6

36 -> -20 -> -39 -> -7

36 -> -20 -> -39 -> -8

36 -> -20 -> -39 -> -9

36 -> -20 -> -39 -> -10

36 -> -20 -> -39 -> -17

36 -> -20 -> -39 -> -18

36 -> -20 -> -39 -> -19

36 -> -20 -> -39 -> -22

36 -> -20 -> -39 -> -24

36 -> -20 -> -39 -> -26

36 -> -20 -> -39 -> -27

36 -> -20 -> -39 -> -28

36 -> -20 -> -39 -> -29

36 -> -20 -> -39 -> -30

36 -> -20 -> -39 -> -31

36 -> -20 -> -39 -> -32

36 -> -20 -> -39 -> -33

36 -> -20 -> -39 -> -35

36 -> -20 -> -39 -> -36

36 -> -20 -> -39 -> -42

36 -> -20 -> -39 -> -43

36 -> -20 -> -39 -> -44

36 -> -20 -> -39 -> -45

36 -> -20 -> -39 -> -46

36 -> -20 -> -39 -> -47

36 -> -20 -> -39 -> -48

36 -> -20 -> -39 -> -49

36 -> -20 -> -39 -> -51

36 -> -20 -> -39 -> -55

36 -> -20 -> -39 -> -56

36 -> -20 -> -39 -> -58

36 -> -20 -> -39 -> -60

36 -> -20 -> -39 -> -61

36 -> -20 -> -39 -> -62

36 -> -20 -> -39 -> -63

36 -> -20 -> -39 -> -64

36 -> -20 -> -39 -> -66

36 -> -20 -> -39 -> -68

36 -> -20 -> -39 -> -69

36 -> -20 -> -39 -> -73

36 -> -20 -> -39 -> -77

36 -> -20 -> -39 -> -78

36 -> -20 -> -39 -> -81

36 -> -20 -> -39 -> -83

36 -> -20 -> -39 -> -84

36 -> -20 -> -39 -> -86

36 -> -20 -> -39 -> -88

36 -> -20 -> -39 -> -89

36 -> -20 -> -39 -> -91

36 -> -20 -> -39 -> -92

36 -> -20 -> -39 -> -95

36 -> -20 -> -39 -> -96

36 -> -20 -> -39 -> -97

36 -> -20 -> -39 -> -98

36 -> -20 -> -39 -> -99

36 -> -20 -> -39 -> -100

36 -> -20 -> -39 -> -101

36 -> -20 -> -39 -> -105

36 -> -20 -> -39 -> -106

36 -> -20 -> -39 -> -107

36 -> -20 -> -39 -> -110

36 -> -20 -> -39 -> -114

36 -> -20 -> -39 -> -116

36 -> -20 -> -39 -> -117

36 -> -20 -> -39 -> -118

36 -> -20 -> -39 -> -119

36 -> -20 -> -39 -> -120

36 -> -20 -> -39 -> -121

36 -> -20 -> -39 -> -122

36 -> -20 -> -39 -> -124

36 -> -20 -> -39 -> -125

36 -> -20 -> -39 -> -127

36 -> -20 -> -39 -> -130

36 -> -20 -> -39 -> -131

36 -> -20 -> -39 -> -132

36 -> -20 -> -39 -> -133

36 -> -20 -> -39 -> -134

36 -> -20 -> -39 -> -135

36 -> -20 -> -39 -> -136

36 -> -20 -> -39 -> -137

36 -> -20 -> -39 -> -141

36 -> -20 -> -39 -> -144

36 -> -20 -> -39 -> -145

36 -> -20 -> -39 -> -146

36 -> -20 -> -39 -> -147

36 -> -20 -> -39 -> -150

36 -> -20 -> -39 -> -154

36 -> -20 -> -39 -> -155

36 -> -20 -> -39 -> -156

36 -> -20 -> -43

36 -> -20 -> -43 -> -3

36 -> -20 -> -43 -> -4

36 -> -20 -> -43 -> -7

36 -> -20 -> -43 -> -8

36 -> -20 -> -43 -> -9

36 -> -20 -> -43 -> -10

36 -> -20 -> -43 -> -17

36 -> -20 -> -43 -> -18

36 -> -20 -> -43 -> -19

36 -> -20 -> -43 -> -22

36 -> -20 -> -43 -> -24

36 -> -20 -> -43 -> -30

36 -> -20 -> -43 -> -31

36 -> -20 -> -43 -> -32

36 -> -20 -> -43 -> -33

36 -> -20 -> -43 -> -35

36 -> -20 -> -43 -> -36

36 -> -20 -> -43 -> -39

36 -> -20 -> -43 -> -41

36 -> -20 -> -43 -> -44

36 -> -20 -> -43 -> -45

36 -> -20 -> -43 -> -46

36 -> -20 -> -43 -> -47

36 -> -20 -> -43 -> -49

36 -> -20 -> -43 -> -58

36 -> -20 -> -43 -> -60

36 -> -20 -> -43 -> -61

36 -> -20 -> -43 -> -62

36 -> -20 -> -43 -> -63

36 -> -20 -> -43 -> -64

36 -> -20 -> -43 -> -66

36 -> -20 -> -43 -> -68

36 -> -20 -> -43 -> -69

36 -> -20 -> -43 -> -73

36 -> -20 -> -43 -> -77

36 -> -20 -> -43 -> -78

36 -> -20 -> -43 -> -81

36 -> -20 -> -43 -> -84

36 -> -20 -> -43 -> -88

36 -> -20 -> -43 -> -91

36 -> -20 -> -43 -> -92

36 -> -20 -> -43 -> -95

36 -> -20 -> -43 -> -96

36 -> -20 -> -43 -> -97

36 -> -20 -> -43 -> -98

36 -> -20 -> -43 -> -99

36 -> -20 -> -43 -> -101

36 -> -20 -> -43 -> -107

36 -> -20 -> -43 -> -109

36 -> -20 -> -43 -> -110

36 -> -20 -> -43 -> -111

36 -> -20 -> -43 -> -116

36 -> -20 -> -43 -> -117

36 -> -20 -> -43 -> -120

36 -> -20 -> -43 -> -124

36 -> -20 -> -43 -> -128

36 -> -20 -> -43 -> -129

36 -> -20 -> -43 -> -130

36 -> -20 -> -43 -> -131

36 -> -20 -> -43 -> -132

36 -> -20 -> -43 -> -134

36 -> -20 -> -43 -> -135

36 -> -20 -> -43 -> -136

36 -> -20 -> -43 -> -137

36 -> -20 -> -43 -> -141

36 -> -20 -> -43 -> -144

36 -> -20 -> -43 -> -147

36 -> -20 -> -43 -> -150

36 -> -20 -> -43 -> -151

36 -> -20 -> -43 -> -154

36 -> -20 -> -43 -> -156

36 -> -20 -> -48

36 -> -20 -> -48 -> -1

36 -> -20 -> -48 -> -3

36 -> -20 -> -48 -> -4

36 -> -20 -> -48 -> -6

36 -> -20 -> -48 -> -8

36 -> -20 -> -48 -> -9

36 -> -20 -> -48 -> -10

36 -> -20 -> -48 -> -19

36 -> -20 -> -48 -> -24

36 -> -20 -> -48 -> -29

36 -> -20 -> -48 -> -30

36 -> -20 -> -48 -> -31

36 -> -20 -> -48 -> -33

36 -> -20 -> -48 -> -35

36 -> -20 -> -48 -> -39

36 -> -20 -> -48 -> -44

36 -> -20 -> -48 -> -45

36 -> -20 -> -48 -> -46

36 -> -20 -> -48 -> -47

36 -> -20 -> -48 -> -49

36 -> -20 -> -48 -> -51

36 -> -20 -> -48 -> -58

36 -> -20 -> -48 -> -60

36 -> -20 -> -48 -> -62

36 -> -20 -> -48 -> -63

36 -> -20 -> -48 -> -64

36 -> -20 -> -48 -> -66

36 -> -20 -> -48 -> -69

36 -> -20 -> -48 -> -73

36 -> -20 -> -48 -> -77

36 -> -20 -> -48 -> -78

36 -> -20 -> -48 -> -81

36 -> -20 -> -48 -> -83

36 -> -20 -> -48 -> -84

36 -> -20 -> -48 -> -86

36 -> -20 -> -48 -> -89

36 -> -20 -> -48 -> -91

36 -> -20 -> -48 -> -95

36 -> -20 -> -48 -> -96

36 -> -20 -> -48 -> -98

36 -> -20 -> -48 -> -99

36 -> -20 -> -48 -> -100

36 -> -20 -> -48 -> -101

36 -> -20 -> -48 -> -106

36 -> -20 -> -48 -> -107

36 -> -20 -> -48 -> -114

36 -> -20 -> -48 -> -117

36 -> -20 -> -48 -> -120

36 -> -20 -> -48 -> -124

36 -> -20 -> -48 -> -127

36 -> -20 -> -48 -> -131

36 -> -20 -> -48 -> -132

36 -> -20 -> -48 -> -133

36 -> -20 -> -48 -> -135

36 -> -20 -> -48 -> -136

36 -> -20 -> -48 -> -137

36 -> -20 -> -48 -> -141

36 -> -20 -> -48 -> -146

36 -> -20 -> -48 -> -150

36 -> -20 -> -48 -> -154

36 -> -20 -> -48 -> -155

36 -> -20 -> -48 -> -156

36 -> -20 -> -56

36 -> -20 -> -56 -> -1

36 -> -20 -> -56 -> -3

36 -> -20 -> -56 -> -19

36 -> -20 -> -56 -> -22

36 -> -20 -> -56 -> -29

36 -> -20 -> -56 -> -32

36 -> -20 -> -56 -> -39

36 -> -20 -> -56 -> -42

36 -> -20 -> -56 -> -55

36 -> -20 -> -56 -> -62

36 -> -20 -> -56 -> -63

36 -> -20 -> -56 -> -64

36 -> -20 -> -56 -> -68

36 -> -20 -> -56 -> -71

36 -> -20 -> -56 -> -73

36 -> -20 -> -56 -> -78

36 -> -20 -> -56 -> -81

36 -> -20 -> -56 -> -95

36 -> -20 -> -56 -> -98

36 -> -20 -> -56 -> -99

36 -> -20 -> -56 -> -101

36 -> -20 -> -56 -> -105

36 -> -20 -> -56 -> -106

36 -> -20 -> -56 -> -107

36 -> -20 -> -56 -> -113

36 -> -20 -> -56 -> -117

36 -> -20 -> -56 -> -124

36 -> -20 -> -56 -> -133

36 -> -20 -> -56 -> -137

36 -> -20 -> -56 -> -142

36 -> -20 -> -56 -> -145

36 -> -20 -> -56 -> -146

36 -> -20 -> -56 -> -147

36 -> -20 -> -56 -> -150

36 -> -20 -> -56 -> -154

36 -> -20 -> -56 -> -156

36 -> -20 -> -61

36 -> -20 -> -61 -> -3

36 -> -20 -> -61 -> -4

36 -> -20 -> -61 -> -7

36 -> -20 -> -61 -> -8

36 -> -20 -> -61 -> -9

36 -> -20 -> -61 -> -10

36 -> -20 -> -61 -> -17

36 -> -20 -> -61 -> -19

36 -> -20 -> -61 -> -22

36 -> -20 -> -61 -> -24

36 -> -20 -> -61 -> -26

36 -> -20 -> -61 -> -27

36 -> -20 -> -61 -> -28

36 -> -20 -> -61 -> -29

36 -> -20 -> -61 -> -31

36 -> -20 -> -61 -> -33

36 -> -20 -> -61 -> -39

36 -> -20 -> -61 -> -43

36 -> -20 -> -61 -> -44

36 -> -20 -> -61 -> -45

36 -> -20 -> -61 -> -46

36 -> -20 -> -61 -> -47

36 -> -20 -> -61 -> -49

36 -> -20 -> -61 -> -58

36 -> -20 -> -61 -> -60

36 -> -20 -> -61 -> -62

36 -> -20 -> -61 -> -63

36 -> -20 -> -61 -> -66

36 -> -20 -> -61 -> -68

36 -> -20 -> -61 -> -69

36 -> -20 -> -61 -> -73

36 -> -20 -> -61 -> -77

36 -> -20 -> -61 -> -78

36 -> -20 -> -61 -> -81

36 -> -20 -> -61 -> -84

36 -> -20 -> -61 -> -86

36 -> -20 -> -61 -> -88

36 -> -20 -> -61 -> -91

36 -> -20 -> -61 -> -92

36 -> -20 -> -61 -> -96

36 -> -20 -> -61 -> -98

36 -> -20 -> -61 -> -99

36 -> -20 -> -61 -> -101

36 -> -20 -> -61 -> -107

36 -> -20 -> -61 -> -110

36 -> -20 -> -61 -> -117

36 -> -20 -> -61 -> -118

36 -> -20 -> -61 -> -119

36 -> -20 -> -61 -> -120

36 -> -20 -> -61 -> -121

36 -> -20 -> -61 -> -122

36 -> -20 -> -61 -> -124

36 -> -20 -> -61 -> -130

36 -> -20 -> -61 -> -131

36 -> -20 -> -61 -> -132

36 -> -20 -> -61 -> -133

36 -> -20 -> -61 -> -135

36 -> -20 -> -61 -> -136

36 -> -20 -> -61 -> -137

36 -> -20 -> -61 -> -141

36 -> -20 -> -61 -> -144

36 -> -20 -> -61 -> -145

36 -> -20 -> -61 -> -147

36 -> -20 -> -61 -> -150

36 -> -20 -> -61 -> -156

36 -> -20 -> -62

36 -> -20 -> -62 -> -1

36 -> -20 -> -62 -> -3

36 -> -20 -> -62 -> -4

36 -> -20 -> -62 -> -5

36 -> -20 -> -62 -> -6

36 -> -20 -> -62 -> -7

36 -> -20 -> -62 -> -8

36 -> -20 -> -62 -> -9

36 -> -20 -> -62 -> -10

36 -> -20 -> -62 -> -17

36 -> -20 -> -62 -> -18

36 -> -20 -> -62 -> -19

36 -> -20 -> -62 -> -22

36 -> -20 -> -62 -> -24

36 -> -20 -> -62 -> -26

36 -> -20 -> -62 -> -27

36 -> -20 -> -62 -> -28

36 -> -20 -> -62 -> -29

36 -> -20 -> -62 -> -30

36 -> -20 -> -62 -> -31

36 -> -20 -> -62 -> -32

36 -> -20 -> -62 -> -33

36 -> -20 -> -62 -> -35

36 -> -20 -> -62 -> -36

36 -> -20 -> -62 -> -39

36 -> -20 -> -62 -> -42

36 -> -20 -> -62 -> -43

36 -> -20 -> -62 -> -44

36 -> -20 -> -62 -> -45

36 -> -20 -> -62 -> -46

36 -> -20 -> -62 -> -47

36 -> -20 -> -62 -> -48

36 -> -20 -> -62 -> -49

36 -> -20 -> -62 -> -55

36 -> -20 -> -62 -> -56

36 -> -20 -> -62 -> -58

36 -> -20 -> -62 -> -60

36 -> -20 -> -62 -> -61

36 -> -20 -> -62 -> -63

36 -> -20 -> -62 -> -64

36 -> -20 -> -62 -> -66

36 -> -20 -> -62 -> -68

36 -> -20 -> -62 -> -69

36 -> -20 -> -62 -> -71

36 -> -20 -> -62 -> -73

36 -> -20 -> -62 -> -77

36 -> -20 -> -62 -> -78

36 -> -20 -> -62 -> -81

36 -> -20 -> -62 -> -83

36 -> -20 -> -62 -> -84

36 -> -20 -> -62 -> -86

36 -> -20 -> -62 -> -88

36 -> -20 -> -62 -> -89

36 -> -20 -> -62 -> -91

36 -> -20 -> -62 -> -92

36 -> -20 -> -62 -> -93

36 -> -20 -> -62 -> -95

36 -> -20 -> -62 -> -96

36 -> -20 -> -62 -> -97

36 -> -20 -> -62 -> -98

36 -> -20 -> -62 -> -99

36 -> -20 -> -62 -> -101

36 -> -20 -> -62 -> -105

36 -> -20 -> -62 -> -106

36 -> -20 -> -62 -> -107

36 -> -20 -> -62 -> -110

36 -> -20 -> -62 -> -113

36 -> -20 -> -62 -> -114

36 -> -20 -> -62 -> -116

36 -> -20 -> -62 -> -117

36 -> -20 -> -62 -> -118

36 -> -20 -> -62 -> -119

36 -> -20 -> -62 -> -120

36 -> -20 -> -62 -> -121

36 -> -20 -> -62 -> -122

36 -> -20 -> -62 -> -124

36 -> -20 -> -62 -> -130

36 -> -20 -> -62 -> -131

36 -> -20 -> -62 -> -132

36 -> -20 -> -62 -> -133

36 -> -20 -> -62 -> -135

36 -> -20 -> -62 -> -136

36 -> -20 -> -62 -> -137

36 -> -20 -> -62 -> -141

36 -> -20 -> -62 -> -144

36 -> -20 -> -62 -> -145

36 -> -20 -> -62 -> -146

36 -> -20 -> -62 -> -147

36 -> -20 -> -62 -> -150

36 -> -20 -> -62 -> -154

36 -> -20 -> -62 -> -155

36 -> -20 -> -62 -> -156

36 -> -20 -> -63

36 -> -20 -> -63 -> -1

36 -> -20 -> -63 -> -3

36 -> -20 -> -63 -> -4

36 -> -20 -> -63 -> -6

36 -> -20 -> -63 -> -7

36 -> -20 -> -63 -> -8

36 -> -20 -> -63 -> -9

36 -> -20 -> -63 -> -10

36 -> -20 -> -63 -> -17

36 -> -20 -> -63 -> -18

36 -> -20 -> -63 -> -19

36 -> -20 -> -63 -> -22

36 -> -20 -> -63 -> -24

36 -> -20 -> -63 -> -26

36 -> -20 -> -63 -> -27

36 -> -20 -> -63 -> -28

36 -> -20 -> -63 -> -29

36 -> -20 -> -63 -> -30

36 -> -20 -> -63 -> -31

36 -> -20 -> -63 -> -32

36 -> -20 -> -63 -> -33

36 -> -20 -> -63 -> -35

36 -> -20 -> -63 -> -36

36 -> -20 -> -63 -> -39

36 -> -20 -> -63 -> -42

36 -> -20 -> -63 -> -43

36 -> -20 -> -63 -> -44

36 -> -20 -> -63 -> -45

36 -> -20 -> -63 -> -46

36 -> -20 -> -63 -> -47

36 -> -20 -> -63 -> -48

36 -> -20 -> -63 -> -49

36 -> -20 -> -63 -> -51

36 -> -20 -> -63 -> -55

36 -> -20 -> -63 -> -56

36 -> -20 -> -63 -> -58

36 -> -20 -> -63 -> -60

36 -> -20 -> -63 -> -61

36 -> -20 -> -63 -> -62

36 -> -20 -> -63 -> -64

36 -> -20 -> -63 -> -66

36 -> -20 -> -63 -> -68

36 -> -20 -> -63 -> -69

36 -> -20 -> -63 -> -73

36 -> -20 -> -63 -> -77

36 -> -20 -> -63 -> -78

36 -> -20 -> -63 -> -81

36 -> -20 -> -63 -> -83

36 -> -20 -> -63 -> -84

36 -> -20 -> -63 -> -86

36 -> -20 -> -63 -> -88

36 -> -20 -> -63 -> -89

36 -> -20 -> -63 -> -91

36 -> -20 -> -63 -> -92

36 -> -20 -> -63 -> -95

36 -> -20 -> -63 -> -96

36 -> -20 -> -63 -> -97

36 -> -20 -> -63 -> -98

36 -> -20 -> -63 -> -99

36 -> -20 -> -63 -> -100

36 -> -20 -> -63 -> -101

36 -> -20 -> -63 -> -105

36 -> -20 -> -63 -> -106

36 -> -20 -> -63 -> -107

36 -> -20 -> -63 -> -110

36 -> -20 -> -63 -> -114

36 -> -20 -> -63 -> -116

36 -> -20 -> -63 -> -117

36 -> -20 -> -63 -> -118

36 -> -20 -> -63 -> -119

36 -> -20 -> -63 -> -120

36 -> -20 -> -63 -> -121

36 -> -20 -> -63 -> -122

36 -> -20 -> -63 -> -124

36 -> -20 -> -63 -> -125

36 -> -20 -> -63 -> -127

36 -> -20 -> -63 -> -130

36 -> -20 -> -63 -> -131

36 -> -20 -> -63 -> -132

36 -> -20 -> -63 -> -133

36 -> -20 -> -63 -> -134

36 -> -20 -> -63 -> -135

36 -> -20 -> -63 -> -136

36 -> -20 -> -63 -> -137

36 -> -20 -> -63 -> -141

36 -> -20 -> -63 -> -144

36 -> -20 -> -63 -> -145

36 -> -20 -> -63 -> -146

36 -> -20 -> -63 -> -147

36 -> -20 -> -63 -> -150

36 -> -20 -> -63 -> -154

36 -> -20 -> -63 -> -155

36 -> -20 -> -63 -> -156

36 -> -20 -> -64

36 -> -20 -> -64 -> -1

36 -> -20 -> -64 -> -3

36 -> -20 -> -64 -> -4

36 -> -20 -> -64 -> -6

36 -> -20 -> -64 -> -8

36 -> -20 -> -64 -> -9

36 -> -20 -> -64 -> -10

36 -> -20 -> -64 -> -17

36 -> -20 -> -64 -> -19

36 -> -20 -> -64 -> -22

36 -> -20 -> -64 -> -24

36 -> -20 -> -64 -> -29

36 -> -20 -> -64 -> -30

36 -> -20 -> -64 -> -31

36 -> -20 -> -64 -> -32

36 -> -20 -> -64 -> -33

36 -> -20 -> -64 -> -35

36 -> -20 -> -64 -> -36

36 -> -20 -> -64 -> -39

36 -> -20 -> -64 -> -41

36 -> -20 -> -64 -> -43

36 -> -20 -> -64 -> -44

36 -> -20 -> -64 -> -45

36 -> -20 -> -64 -> -46

36 -> -20 -> -64 -> -47

36 -> -20 -> -64 -> -48

36 -> -20 -> -64 -> -49

36 -> -20 -> -64 -> -51

36 -> -20 -> -64 -> -56

36 -> -20 -> -64 -> -58

36 -> -20 -> -64 -> -60

36 -> -20 -> -64 -> -62

36 -> -20 -> -64 -> -63

36 -> -20 -> -64 -> -66

36 -> -20 -> -64 -> -69

36 -> -20 -> -64 -> -73

36 -> -20 -> -64 -> -77

36 -> -20 -> -64 -> -78

36 -> -20 -> -64 -> -81

36 -> -20 -> -64 -> -83

36 -> -20 -> -64 -> -84

36 -> -20 -> -64 -> -86

36 -> -20 -> -64 -> -89

36 -> -20 -> -64 -> -91

36 -> -20 -> -64 -> -92

36 -> -20 -> -64 -> -95

36 -> -20 -> -64 -> -96

36 -> -20 -> -64 -> -98

36 -> -20 -> -64 -> -99

36 -> -20 -> -64 -> -100

36 -> -20 -> -64 -> -101

36 -> -20 -> -64 -> -106

36 -> -20 -> -64 -> -107

36 -> -20 -> -64 -> -109

36 -> -20 -> -64 -> -111

36 -> -20 -> -64 -> -114

36 -> -20 -> -64 -> -117

36 -> -20 -> -64 -> -120

36 -> -20 -> -64 -> -124

36 -> -20 -> -64 -> -127

36 -> -20 -> -64 -> -131

36 -> -20 -> -64 -> -132

36 -> -20 -> -64 -> -133

36 -> -20 -> -64 -> -135

36 -> -20 -> -64 -> -136

36 -> -20 -> -64 -> -137

36 -> -20 -> -64 -> -141

36 -> -20 -> -64 -> -144

36 -> -20 -> -64 -> -146

36 -> -20 -> -64 -> -147

36 -> -20 -> -64 -> -150

36 -> -20 -> -64 -> -151

36 -> -20 -> -64 -> -154

36 -> -20 -> -64 -> -155

36 -> -20 -> -64 -> -156

36 -> -20 -> -68

36 -> -20 -> -68 -> -1

36 -> -20 -> -68 -> -2

36 -> -20 -> -68 -> -3

36 -> -20 -> -68 -> -17

36 -> -20 -> -68 -> -19

36 -> -20 -> -68 -> -22

36 -> -20 -> -68 -> -26

36 -> -20 -> -68 -> -27

36 -> -20 -> -68 -> -28

36 -> -20 -> -68 -> -29

36 -> -20 -> -68 -> -32

36 -> -20 -> -68 -> -39

36 -> -20 -> -68 -> -41

36 -> -20 -> -68 -> -43

36 -> -20 -> -68 -> -56

36 -> -20 -> -68 -> -61

36 -> -20 -> -68 -> -62

36 -> -20 -> -68 -> -63

36 -> -20 -> -68 -> -71

36 -> -20 -> -68 -> -73

36 -> -20 -> -68 -> -78

36 -> -20 -> -68 -> -80

36 -> -20 -> -68 -> -81

36 -> -20 -> -68 -> -86

36 -> -20 -> -68 -> -88

36 -> -20 -> -68 -> -92

36 -> -20 -> -68 -> -98

36 -> -20 -> -68 -> -99

36 -> -20 -> -68 -> -101

36 -> -20 -> -68 -> -106

36 -> -20 -> -68 -> -107

36 -> -20 -> -68 -> -109

36 -> -20 -> -68 -> -111

36 -> -20 -> -68 -> -113

36 -> -20 -> -68 -> -117

36 -> -20 -> -68 -> -118

36 -> -20 -> -68 -> -119

36 -> -20 -> -68 -> -121

36 -> -20 -> -68 -> -122

36 -> -20 -> -68 -> -124

36 -> -20 -> -68 -> -128

36 -> -20 -> -68 -> -129

36 -> -20 -> -68 -> -133

36 -> -20 -> -68 -> -137

36 -> -20 -> -68 -> -142

36 -> -20 -> -68 -> -144

36 -> -20 -> -68 -> -145

36 -> -20 -> -68 -> -146

36 -> -20 -> -68 -> -147

36 -> -20 -> -68 -> -150

36 -> -20 -> -68 -> -151

36 -> -20 -> -68 -> -156

36 -> -20 -> -70

36 -> -20 -> -70 -> -4

36 -> -20 -> -70 -> -8

36 -> -20 -> -70 -> -9

36 -> -20 -> -70 -> -10

36 -> -20 -> -70 -> -11

36 -> -20 -> -70 -> -13

36 -> -20 -> -70 -> -16

36 -> -20 -> -70 -> -24

36 -> -20 -> -70 -> -31

36 -> -20 -> -70 -> -33

36 -> -20 -> -70 -> -34

36 -> -20 -> -70 -> -44

36 -> -20 -> -70 -> -45

36 -> -20 -> -70 -> -46

36 -> -20 -> -70 -> -47

36 -> -20 -> -70 -> -49

36 -> -20 -> -70 -> -52

36 -> -20 -> -70 -> -53

36 -> -20 -> -70 -> -58

36 -> -20 -> -70 -> -59

36 -> -20 -> -70 -> -60

36 -> -20 -> -70 -> -66

36 -> -20 -> -70 -> -67

36 -> -20 -> -70 -> -69

36 -> -20 -> -70 -> -74

36 -> -20 -> -70 -> -75

36 -> -20 -> -70 -> -77

36 -> -20 -> -70 -> -80

36 -> -20 -> -70 -> -82

36 -> -20 -> -70 -> -84

36 -> -20 -> -70 -> -85

36 -> -20 -> -70 -> -90

36 -> -20 -> -70 -> -91

36 -> -20 -> -70 -> -96

36 -> -20 -> -70 -> -112

36 -> -20 -> -70 -> -120

36 -> -20 -> -70 -> -125

36 -> -20 -> -70 -> -131

36 -> -20 -> -70 -> -132

36 -> -20 -> -70 -> -135

36 -> -20 -> -70 -> -136

36 -> -20 -> -70 -> -138

36 -> -20 -> -70 -> -140

36 -> -20 -> -70 -> -141

36 -> -20 -> -70 -> -143

36 -> -20 -> -70 -> -152

36 -> -20 -> -70 -> -153

36 -> -20 -> -73

36 -> -20 -> -73 -> -1

36 -> -20 -> -73 -> -3

36 -> -20 -> -73 -> -6

36 -> -20 -> -73 -> -17

36 -> -20 -> -73 -> -19

36 -> -20 -> -73 -> -22

36 -> -20 -> -73 -> -26

36 -> -20 -> -73 -> -27

36 -> -20 -> -73 -> -28

36 -> -20 -> -73 -> -29

36 -> -20 -> -73 -> -30

36 -> -20 -> -73 -> -32

36 -> -20 -> -73 -> -35

36 -> -20 -> -73 -> -39

36 -> -20 -> -73 -> -43

36 -> -20 -> -73 -> -48

36 -> -20 -> -73 -> -56

36 -> -20 -> -73 -> -61

36 -> -20 -> -73 -> -62

36 -> -20 -> -73 -> -63

36 -> -20 -> -73 -> -64

36 -> -20 -> -73 -> -68

36 -> -20 -> -73 -> -71

36 -> -20 -> -73 -> -78

36 -> -20 -> -73 -> -81

36 -> -20 -> -73 -> -83

36 -> -20 -> -73 -> -86

36 -> -20 -> -73 -> -88

36 -> -20 -> -73 -> -89

36 -> -20 -> -73 -> -92

36 -> -20 -> -73 -> -95

36 -> -20 -> -73 -> -98

36 -> -20 -> -73 -> -99

36 -> -20 -> -73 -> -101

36 -> -20 -> -73 -> -106

36 -> -20 -> -73 -> -107

36 -> -20 -> -73 -> -113

36 -> -20 -> -73 -> -114

36 -> -20 -> -73 -> -117

36 -> -20 -> -73 -> -118

36 -> -20 -> -73 -> -119

36 -> -20 -> -73 -> -121

36 -> -20 -> -73 -> -122

36 -> -20 -> -73 -> -124

36 -> -20 -> -73 -> -133

36 -> -20 -> -73 -> -137

36 -> -20 -> -73 -> -144

36 -> -20 -> -73 -> -145

36 -> -20 -> -73 -> -146

36 -> -20 -> -73 -> -147

36 -> -20 -> -73 -> -150

36 -> -20 -> -73 -> -154

36 -> -20 -> -73 -> -155

36 -> -20 -> -73 -> -156

36 -> -20 -> -74

36 -> -20 -> -74 -> -11

36 -> -20 -> -74 -> -13

36 -> -20 -> -74 -> -16

36 -> -20 -> -74 -> -25

36 -> -20 -> -74 -> -52

36 -> -20 -> -74 -> -53

36 -> -20 -> -74 -> -59

36 -> -20 -> -74 -> -67

36 -> -20 -> -74 -> -70

36 -> -20 -> -74 -> -75

36 -> -20 -> -74 -> -80

36 -> -20 -> -74 -> -82

36 -> -20 -> -74 -> -85

36 -> -20 -> -74 -> -90

36 -> -20 -> -74 -> -101

36 -> -20 -> -74 -> -112

36 -> -20 -> -74 -> -115

36 -> -20 -> -74 -> -125

36 -> -20 -> -74 -> -138

36 -> -20 -> -74 -> -140

36 -> -20 -> -74 -> -143

36 -> -20 -> -74 -> -152

36 -> -20 -> -74 -> -153

36 -> -20 -> -75

36 -> -20 -> -75 -> -11

36 -> -20 -> -75 -> -13

36 -> -20 -> -75 -> -16

36 -> -20 -> -75 -> -25

36 -> -20 -> -75 -> -52

36 -> -20 -> -75 -> -53

36 -> -20 -> -75 -> -59

36 -> -20 -> -75 -> -67

36 -> -20 -> -75 -> -70

36 -> -20 -> -75 -> -74

36 -> -20 -> -75 -> -80

36 -> -20 -> -75 -> -82

36 -> -20 -> -75 -> -85

36 -> -20 -> -75 -> -90

36 -> -20 -> -75 -> -101

36 -> -20 -> -75 -> -112

36 -> -20 -> -75 -> -115

36 -> -20 -> -75 -> -125

36 -> -20 -> -75 -> -138

36 -> -20 -> -75 -> -140

36 -> -20 -> -75 -> -143

36 -> -20 -> -75 -> -152

36 -> -20 -> -75 -> -153

36 -> -20 -> -78

36 -> -20 -> -78 -> -1

36 -> -20 -> -78 -> -3

36 -> -20 -> -78 -> -4

36 -> -20 -> -78 -> -6

36 -> -20 -> -78 -> -8

36 -> -20 -> -78 -> -9

36 -> -20 -> -78 -> -10

36 -> -20 -> -78 -> -17

36 -> -20 -> -78 -> -19

36 -> -20 -> -78 -> -22

36 -> -20 -> -78 -> -24

36 -> -20 -> -78 -> -26

36 -> -20 -> -78 -> -27

36 -> -20 -> -78 -> -28

36 -> -20 -> -78 -> -29

36 -> -20 -> -78 -> -30

36 -> -20 -> -78 -> -31

36 -> -20 -> -78 -> -32

36 -> -20 -> -78 -> -33

36 -> -20 -> -78 -> -35

36 -> -20 -> -78 -> -36

36 -> -20 -> -78 -> -39

36 -> -20 -> -78 -> -43

36 -> -20 -> -78 -> -44

36 -> -20 -> -78 -> -45

36 -> -20 -> -78 -> -46

36 -> -20 -> -78 -> -47

36 -> -20 -> -78 -> -48

36 -> -20 -> -78 -> -49

36 -> -20 -> -78 -> -51

36 -> -20 -> -78 -> -56

36 -> -20 -> -78 -> -58

36 -> -20 -> -78 -> -60

36 -> -20 -> -78 -> -61

36 -> -20 -> -78 -> -62

36 -> -20 -> -78 -> -63

36 -> -20 -> -78 -> -64

36 -> -20 -> -78 -> -66

36 -> -20 -> -78 -> -68

36 -> -20 -> -78 -> -69

36 -> -20 -> -78 -> -73

36 -> -20 -> -78 -> -77

36 -> -20 -> -78 -> -81

36 -> -20 -> -78 -> -83

36 -> -20 -> -78 -> -84

36 -> -20 -> -78 -> -86

36 -> -20 -> -78 -> -88

36 -> -20 -> -78 -> -89

36 -> -20 -> -78 -> -91

36 -> -20 -> -78 -> -92

36 -> -20 -> -78 -> -95

36 -> -20 -> -78 -> -96

36 -> -20 -> -78 -> -98

36 -> -20 -> -78 -> -99

36 -> -20 -> -78 -> -100

36 -> -20 -> -78 -> -101

36 -> -20 -> -78 -> -106

36 -> -20 -> -78 -> -107

36 -> -20 -> -78 -> -114

36 -> -20 -> -78 -> -117

36 -> -20 -> -78 -> -118

36 -> -20 -> -78 -> -119

36 -> -20 -> -78 -> -120

36 -> -20 -> -78 -> -121

36 -> -20 -> -78 -> -122

36 -> -20 -> -78 -> -124

36 -> -20 -> -78 -> -127

36 -> -20 -> -78 -> -131

36 -> -20 -> -78 -> -132

36 -> -20 -> -78 -> -133

36 -> -20 -> -78 -> -135

36 -> -20 -> -78 -> -136

36 -> -20 -> -78 -> -137

36 -> -20 -> -78 -> -141

36 -> -20 -> -78 -> -144

36 -> -20 -> -78 -> -145

36 -> -20 -> -78 -> -146

36 -> -20 -> -78 -> -147

36 -> -20 -> -78 -> -150

36 -> -20 -> -78 -> -154

36 -> -20 -> -78 -> -155

36 -> -20 -> -78 -> -156

36 -> -20 -> -81

36 -> -20 -> -81 -> -1

36 -> -20 -> -81 -> -3

36 -> -20 -> -81 -> -6

36 -> -20 -> -81 -> -7

36 -> -20 -> -81 -> -17

36 -> -20 -> -81 -> -18

36 -> -20 -> -81 -> -19

36 -> -20 -> -81 -> -22

36 -> -20 -> -81 -> -26

36 -> -20 -> -81 -> -27

36 -> -20 -> -81 -> -28

36 -> -20 -> -81 -> -29

36 -> -20 -> -81 -> -30

36 -> -20 -> -81 -> -32

36 -> -20 -> -81 -> -35

36 -> -20 -> -81 -> -36

36 -> -20 -> -81 -> -39

36 -> -20 -> -81 -> -42

36 -> -20 -> -81 -> -43

36 -> -20 -> -81 -> -48

36 -> -20 -> -81 -> -51

36 -> -20 -> -81 -> -55

36 -> -20 -> -81 -> -56

36 -> -20 -> -81 -> -61

36 -> -20 -> -81 -> -62

36 -> -20 -> -81 -> -63

36 -> -20 -> -81 -> -64

36 -> -20 -> -81 -> -68

36 -> -20 -> -81 -> -71

36 -> -20 -> -81 -> -73

36 -> -20 -> -81 -> -78

36 -> -20 -> -81 -> -83

36 -> -20 -> -81 -> -86

36 -> -20 -> -81 -> -88

36 -> -20 -> -81 -> -89

36 -> -20 -> -81 -> -92

36 -> -20 -> -81 -> -95

36 -> -20 -> -81 -> -97

36 -> -20 -> -81 -> -98

36 -> -20 -> -81 -> -99

36 -> -20 -> -81 -> -100

36 -> -20 -> -81 -> -101

36 -> -20 -> -81 -> -105

36 -> -20 -> -81 -> -106

36 -> -20 -> -81 -> -107

36 -> -20 -> -81 -> -110

36 -> -20 -> -81 -> -113

36 -> -20 -> -81 -> -114

36 -> -20 -> -81 -> -116

36 -> -20 -> -81 -> -117

36 -> -20 -> -81 -> -118

36 -> -20 -> -81 -> -119

36 -> -20 -> -81 -> -121

36 -> -20 -> -81 -> -122

36 -> -20 -> -81 -> -124

36 -> -20 -> -81 -> -127

36 -> -20 -> -81 -> -130

36 -> -20 -> -81 -> -133

36 -> -20 -> -81 -> -134

36 -> -20 -> -81 -> -137

36 -> -20 -> -81 -> -144

36 -> -20 -> -81 -> -145

36 -> -20 -> -81 -> -146

36 -> -20 -> -81 -> -147

36 -> -20 -> -81 -> -150

36 -> -20 -> -81 -> -154

36 -> -20 -> -81 -> -155

36 -> -20 -> -81 -> -156

36 -> -20 -> -82

36 -> -20 -> -82 -> -4

36 -> -20 -> -82 -> -8

36 -> -20 -> -82 -> -9

36 -> -20 -> -82 -> -10

36 -> -20 -> -82 -> -11

36 -> -20 -> -82 -> -13

36 -> -20 -> -82 -> -16

36 -> -20 -> -82 -> -24

36 -> -20 -> -82 -> -25

36 -> -20 -> -82 -> -31

36 -> -20 -> -82 -> -33

36 -> -20 -> -82 -> -44

36 -> -20 -> -82 -> -45

36 -> -20 -> -82 -> -46

36 -> -20 -> -82 -> -47

36 -> -20 -> -82 -> -49

36 -> -20 -> -82 -> -52

36 -> -20 -> -82 -> -53

36 -> -20 -> -82 -> -58

36 -> -20 -> -82 -> -59

36 -> -20 -> -82 -> -60

36 -> -20 -> -82 -> -66

36 -> -20 -> -82 -> -67

36 -> -20 -> -82 -> -69

36 -> -20 -> -82 -> -70

36 -> -20 -> -82 -> -72

36 -> -20 -> -82 -> -74

36 -> -20 -> -82 -> -75

36 -> -20 -> -82 -> -77

36 -> -20 -> -82 -> -80

36 -> -20 -> -82 -> -84

36 -> -20 -> -82 -> -85

36 -> -20 -> -82 -> -90

36 -> -20 -> -82 -> -91

36 -> -20 -> -82 -> -96

36 -> -20 -> -82 -> -112

36 -> -20 -> -82 -> -115

36 -> -20 -> -82 -> -120

36 -> -20 -> -82 -> -125

36 -> -20 -> -82 -> -131

36 -> -20 -> -82 -> -132

36 -> -20 -> -82 -> -135

36 -> -20 -> -82 -> -136

36 -> -20 -> -82 -> -138

36 -> -20 -> -82 -> -140

36 -> -20 -> -82 -> -141

36 -> -20 -> -82 -> -143

36 -> -20 -> -82 -> -152

36 -> -20 -> -82 -> -153

36 -> -20 -> -83

36 -> -20 -> -83 -> -1

36 -> -20 -> -83 -> -3

36 -> -20 -> -83 -> -4

36 -> -20 -> -83 -> -6

36 -> -20 -> -83 -> -7

36 -> -20 -> -83 -> -8

36 -> -20 -> -83 -> -9

36 -> -20 -> -83 -> -10

36 -> -20 -> -83 -> -19

36 -> -20 -> -83 -> -24

36 -> -20 -> -83 -> -29

36 -> -20 -> -83 -> -31

36 -> -20 -> -83 -> -33

36 -> -20 -> -83 -> -39

36 -> -20 -> -83 -> -44

36 -> -20 -> -83 -> -45

36 -> -20 -> -83 -> -46

36 -> -20 -> -83 -> -47

36 -> -20 -> -83 -> -48

36 -> -20 -> -83 -> -49

36 -> -20 -> -83 -> -51

36 -> -20 -> -83 -> -58

36 -> -20 -> -83 -> -60

36 -> -20 -> -83 -> -62

36 -> -20 -> -83 -> -63

36 -> -20 -> -83 -> -64

36 -> -20 -> -83 -> -66

36 -> -20 -> -83 -> -69

36 -> -20 -> -83 -> -73

36 -> -20 -> -83 -> -77

36 -> -20 -> -83 -> -78

36 -> -20 -> -83 -> -81

36 -> -20 -> -83 -> -84

36 -> -20 -> -83 -> -86

36 -> -20 -> -83 -> -89

36 -> -20 -> -83 -> -91

36 -> -20 -> -83 -> -95

36 -> -20 -> -83 -> -96

36 -> -20 -> -83 -> -98

36 -> -20 -> -83 -> -99

36 -> -20 -> -83 -> -100

36 -> -20 -> -83 -> -101

36 -> -20 -> -83 -> -106

36 -> -20 -> -83 -> -107

36 -> -20 -> -83 -> -110

36 -> -20 -> -83 -> -114

36 -> -20 -> -83 -> -117

36 -> -20 -> -83 -> -120

36 -> -20 -> -83 -> -124

36 -> -20 -> -83 -> -127

36 -> -20 -> -83 -> -130

36 -> -20 -> -83 -> -131

36 -> -20 -> -83 -> -132

36 -> -20 -> -83 -> -135

36 -> -20 -> -83 -> -136

36 -> -20 -> -83 -> -137

36 -> -20 -> -83 -> -141

36 -> -20 -> -83 -> -150

36 -> -20 -> -83 -> -154

36 -> -20 -> -83 -> -155

36 -> -20 -> -83 -> -156

36 -> -20 -> -86

36 -> -20 -> -86 -> -1

36 -> -20 -> -86 -> -3

36 -> -20 -> -86 -> -6

36 -> -20 -> -86 -> -7

36 -> -20 -> -86 -> -19

36 -> -20 -> -86 -> -22

36 -> -20 -> -86 -> -26

36 -> -20 -> -86 -> -27

36 -> -20 -> -86 -> -28

36 -> -20 -> -86 -> -29

36 -> -20 -> -86 -> -32

36 -> -20 -> -86 -> -39

36 -> -20 -> -86 -> -40

36 -> -20 -> -86 -> -48

36 -> -20 -> -86 -> -51

36 -> -20 -> -86 -> -61

36 -> -20 -> -86 -> -62

36 -> -20 -> -86 -> -63

36 -> -20 -> -86 -> -64

36 -> -20 -> -86 -> -68

36 -> -20 -> -86 -> -73

36 -> -20 -> -86 -> -78

36 -> -20 -> -86 -> -79

36 -> -20 -> -86 -> -81

36 -> -20 -> -86 -> -83

36 -> -20 -> -86 -> -88

36 -> -20 -> -86 -> -89

36 -> -20 -> -86 -> -94

36 -> -20 -> -86 -> -95

36 -> -20 -> -86 -> -98

36 -> -20 -> -86 -> -99

36 -> -20 -> -86 -> -100

36 -> -20 -> -86 -> -101

36 -> -20 -> -86 -> -106

36 -> -20 -> -86 -> -107

36 -> -20 -> -86 -> -110

36 -> -20 -> -86 -> -114

36 -> -20 -> -86 -> -117

36 -> -20 -> -86 -> -118

36 -> -20 -> -86 -> -119

36 -> -20 -> -86 -> -120

36 -> -20 -> -86 -> -121

36 -> -20 -> -86 -> -122

36 -> -20 -> -86 -> -123

36 -> -20 -> -86 -> -124

36 -> -20 -> -86 -> -125

36 -> -20 -> -86 -> -127

36 -> -20 -> -86 -> -130

36 -> -20 -> -86 -> -133

36 -> -20 -> -86 -> -137

36 -> -20 -> -86 -> -145

36 -> -20 -> -86 -> -147

36 -> -20 -> -86 -> -150

36 -> -20 -> -86 -> -154

36 -> -20 -> -86 -> -155

36 -> -20 -> -86 -> -156

36 -> -20 -> -88

36 -> -20 -> -88 -> -1

36 -> -20 -> -88 -> -3

36 -> -20 -> -88 -> -4

36 -> -20 -> -88 -> -8

36 -> -20 -> -88 -> -9

36 -> -20 -> -88 -> -10

36 -> -20 -> -88 -> -11

36 -> -20 -> -88 -> -17

36 -> -20 -> -88 -> -19

36 -> -20 -> -88 -> -22

36 -> -20 -> -88 -> -24

36 -> -20 -> -88 -> -26

36 -> -20 -> -88 -> -27

36 -> -20 -> -88 -> -28

36 -> -20 -> -88 -> -29

36 -> -20 -> -88 -> -31

36 -> -20 -> -88 -> -33

36 -> -20 -> -88 -> -39

36 -> -20 -> -88 -> -43

36 -> -20 -> -88 -> -44

36 -> -20 -> -88 -> -45

36 -> -20 -> -88 -> -46

36 -> -20 -> -88 -> -47

36 -> -20 -> -88 -> -49

36 -> -20 -> -88 -> -58

36 -> -20 -> -88 -> -60

36 -> -20 -> -88 -> -61

36 -> -20 -> -88 -> -62

36 -> -20 -> -88 -> -63

36 -> -20 -> -88 -> -66

36 -> -20 -> -88 -> -67

36 -> -20 -> -88 -> -68

36 -> -20 -> -88 -> -69

36 -> -20 -> -88 -> -73

36 -> -20 -> -88 -> -77

36 -> -20 -> -88 -> -78

36 -> -20 -> -88 -> -81

36 -> -20 -> -88 -> -84

36 -> -20 -> -88 -> -86

36 -> -20 -> -88 -> -89

36 -> -20 -> -88 -> -91

36 -> -20 -> -88 -> -92

36 -> -20 -> -88 -> -96

36 -> -20 -> -88 -> -98

36 -> -20 -> -88 -> -99

36 -> -20 -> -88 -> -106

36 -> -20 -> -88 -> -107

36 -> -20 -> -88 -> -115

36 -> -20 -> -88 -> -117

36 -> -20 -> -88 -> -118

36 -> -20 -> -88 -> -119

36 -> -20 -> -88 -> -120

36 -> -20 -> -88 -> -121

36 -> -20 -> -88 -> -122

36 -> -20 -> -88 -> -124

36 -> -20 -> -88 -> -131

36 -> -20 -> -88 -> -132

36 -> -20 -> -88 -> -135

36 -> -20 -> -88 -> -136

36 -> -20 -> -88 -> -137

36 -> -20 -> -88 -> -141

36 -> -20 -> -88 -> -144

36 -> -20 -> -88 -> -147

36 -> -20 -> -88 -> -150

36 -> -20 -> -88 -> -156

36 -> -20 -> -89

36 -> -20 -> -89 -> -1

36 -> -20 -> -89 -> -3

36 -> -20 -> -89 -> -4

36 -> -20 -> -89 -> -6

36 -> -20 -> -89 -> -8

36 -> -20 -> -89 -> -9

36 -> -20 -> -89 -> -10

36 -> -20 -> -89 -> -11

36 -> -20 -> -89 -> -19

36 -> -20 -> -89 -> -24

36 -> -20 -> -89 -> -29

36 -> -20 -> -89 -> -31

36 -> -20 -> -89 -> -33

36 -> -20 -> -89 -> -39

36 -> -20 -> -89 -> -44

36 -> -20 -> -89 -> -45

36 -> -20 -> -89 -> -46

36 -> -20 -> -89 -> -47

36 -> -20 -> -89 -> -48

36 -> -20 -> -89 -> -49

36 -> -20 -> -89 -> -51

36 -> -20 -> -89 -> -53

36 -> -20 -> -89 -> -58

36 -> -20 -> -89 -> -60

36 -> -20 -> -89 -> -62

36 -> -20 -> -89 -> -63

36 -> -20 -> -89 -> -64

36 -> -20 -> -89 -> -65

36 -> -20 -> -89 -> -66

36 -> -20 -> -89 -> -67

36 -> -20 -> -89 -> -69

36 -> -20 -> -89 -> -73

36 -> -20 -> -89 -> -77

36 -> -20 -> -89 -> -78

36 -> -20 -> -89 -> -81

36 -> -20 -> -89 -> -83

36 -> -20 -> -89 -> -84

36 -> -20 -> -89 -> -86

36 -> -20 -> -89 -> -88

36 -> -20 -> -89 -> -91

36 -> -20 -> -89 -> -95

36 -> -20 -> -89 -> -96

36 -> -20 -> -89 -> -98

36 -> -20 -> -89 -> -99

36 -> -20 -> -89 -> -100

36 -> -20 -> -89 -> -101

36 -> -20 -> -89 -> -106

36 -> -20 -> -89 -> -107

36 -> -20 -> -89 -> -114

36 -> -20 -> -89 -> -115

36 -> -20 -> -89 -> -117

36 -> -20 -> -89 -> -120

36 -> -20 -> -89 -> -124

36 -> -20 -> -89 -> -127

36 -> -20 -> -89 -> -131

36 -> -20 -> -89 -> -132

36 -> -20 -> -89 -> -135

36 -> -20 -> -89 -> -136

36 -> -20 -> -89 -> -137

36 -> -20 -> -89 -> -141

36 -> -20 -> -89 -> -150

36 -> -20 -> -89 -> -153

36 -> -20 -> -89 -> -154

36 -> -20 -> -89 -> -155

36 -> -20 -> -89 -> -156

36 -> -20 -> -92

36 -> -20 -> -92 -> -3

36 -> -20 -> -92 -> -4

36 -> -20 -> -92 -> -7

36 -> -20 -> -92 -> -8

36 -> -20 -> -92 -> -9

36 -> -20 -> -92 -> -10

36 -> -20 -> -92 -> -17

36 -> -20 -> -92 -> -18

36 -> -20 -> -92 -> -19

36 -> -20 -> -92 -> -22

36 -> -20 -> -92 -> -24

36 -> -20 -> -92 -> -30

36 -> -20 -> -92 -> -31

36 -> -20 -> -92 -> -32

36 -> -20 -> -92 -> -33

36 -> -20 -> -92 -> -35

36 -> -20 -> -92 -> -36

36 -> -20 -> -92 -> -39

36 -> -20 -> -92 -> -41

36 -> -20 -> -92 -> -43

36 -> -20 -> -92 -> -44

36 -> -20 -> -92 -> -45

36 -> -20 -> -92 -> -46

36 -> -20 -> -92 -> -47

36 -> -20 -> -92 -> -49

36 -> -20 -> -92 -> -58

36 -> -20 -> -92 -> -60

36 -> -20 -> -92 -> -61

36 -> -20 -> -92 -> -62

36 -> -20 -> -92 -> -63

36 -> -20 -> -92 -> -64

36 -> -20 -> -92 -> -66

36 -> -20 -> -92 -> -68

36 -> -20 -> -92 -> -69

36 -> -20 -> -92 -> -73

36 -> -20 -> -92 -> -77

36 -> -20 -> -92 -> -78

36 -> -20 -> -92 -> -81

36 -> -20 -> -92 -> -84

36 -> -20 -> -92 -> -88

36 -> -20 -> -92 -> -91

36 -> -20 -> -92 -> -95

36 -> -20 -> -92 -> -96

36 -> -20 -> -92 -> -97

36 -> -20 -> -92 -> -98

36 -> -20 -> -92 -> -99

36 -> -20 -> -92 -> -101

36 -> -20 -> -92 -> -107

36 -> -20 -> -92 -> -109

36 -> -20 -> -92 -> -110

36 -> -20 -> -92 -> -111

36 -> -20 -> -92 -> -116

36 -> -20 -> -92 -> -117

36 -> -20 -> -92 -> -120

36 -> -20 -> -92 -> -124

36 -> -20 -> -92 -> -128

36 -> -20 -> -92 -> -129

36 -> -20 -> -92 -> -130

36 -> -20 -> -92 -> -131

36 -> -20 -> -92 -> -132

36 -> -20 -> -92 -> -134

36 -> -20 -> -92 -> -135

36 -> -20 -> -92 -> -136

36 -> -20 -> -92 -> -137

36 -> -20 -> -92 -> -141

36 -> -20 -> -92 -> -144

36 -> -20 -> -92 -> -147

36 -> -20 -> -92 -> -150

36 -> -20 -> -92 -> -151

36 -> -20 -> -92 -> -154

36 -> -20 -> -92 -> -156

36 -> -20 -> -95

36 -> -20 -> -95 -> -1

36 -> -20 -> -95 -> -3

36 -> -20 -> -95 -> -4

36 -> -20 -> -95 -> -6

36 -> -20 -> -95 -> -7

36 -> -20 -> -95 -> -8

36 -> -20 -> -95 -> -9

36 -> -20 -> -95 -> -10

36 -> -20 -> -95 -> -17

36 -> -20 -> -95 -> -19

36 -> -20 -> -95 -> -22

36 -> -20 -> -95 -> -24

36 -> -20 -> -95 -> -27

36 -> -20 -> -95 -> -29

36 -> -20 -> -95 -> -30

36 -> -20 -> -95 -> -31

36 -> -20 -> -95 -> -32

36 -> -20 -> -95 -> -33

36 -> -20 -> -95 -> -35

36 -> -20 -> -95 -> -36

36 -> -20 -> -95 -> -39

36 -> -20 -> -95 -> -43

36 -> -20 -> -95 -> -44

36 -> -20 -> -95 -> -45

36 -> -20 -> -95 -> -46

36 -> -20 -> -95 -> -47

36 -> -20 -> -95 -> -48

36 -> -20 -> -95 -> -49

36 -> -20 -> -95 -> -51

36 -> -20 -> -95 -> -55

36 -> -20 -> -95 -> -56

36 -> -20 -> -95 -> -58

36 -> -20 -> -95 -> -60

36 -> -20 -> -95 -> -62

36 -> -20 -> -95 -> -63

36 -> -20 -> -95 -> -64

36 -> -20 -> -95 -> -66

36 -> -20 -> -95 -> -69

36 -> -20 -> -95 -> -73

36 -> -20 -> -95 -> -77

36 -> -20 -> -95 -> -78

36 -> -20 -> -95 -> -81

36 -> -20 -> -95 -> -83

36 -> -20 -> -95 -> -84

36 -> -20 -> -95 -> -86

36 -> -20 -> -95 -> -89

36 -> -20 -> -95 -> -91

36 -> -20 -> -95 -> -92

36 -> -20 -> -95 -> -96

36 -> -20 -> -95 -> -98

36 -> -20 -> -95 -> -99

36 -> -20 -> -95 -> -100

36 -> -20 -> -95 -> -101

36 -> -20 -> -95 -> -105

36 -> -20 -> -95 -> -106

36 -> -20 -> -95 -> -107

36 -> -20 -> -95 -> -110

36 -> -20 -> -95 -> -114

36 -> -20 -> -95 -> -117

36 -> -20 -> -95 -> -120

36 -> -20 -> -95 -> -124

36 -> -20 -> -95 -> -127

36 -> -20 -> -95 -> -130

36 -> -20 -> -95 -> -131

36 -> -20 -> -95 -> -132

36 -> -20 -> -95 -> -133

36 -> -20 -> -95 -> -135

36 -> -20 -> -95 -> -136

36 -> -20 -> -95 -> -137

36 -> -20 -> -95 -> -141

36 -> -20 -> -95 -> -144

36 -> -20 -> -95 -> -146

36 -> -20 -> -95 -> -147

36 -> -20 -> -95 -> -150

36 -> -20 -> -95 -> -154

36 -> -20 -> -95 -> -155

36 -> -20 -> -95 -> -156

36 -> -20 -> -98

36 -> -20 -> -98 -> -1

36 -> -20 -> -98 -> -3

36 -> -20 -> -98 -> -4

36 -> -20 -> -98 -> -6

36 -> -20 -> -98 -> -7

36 -> -20 -> -98 -> -8

36 -> -20 -> -98 -> -9

36 -> -20 -> -98 -> -10

36 -> -20 -> -98 -> -17

36 -> -20 -> -98 -> -19

36 -> -20 -> -98 -> -22

36 -> -20 -> -98 -> -24

36 -> -20 -> -98 -> -26

36 -> -20 -> -98 -> -27

36 -> -20 -> -98 -> -28

36 -> -20 -> -98 -> -29

36 -> -20 -> -98 -> -30

36 -> -20 -> -98 -> -31

36 -> -20 -> -98 -> -32

36 -> -20 -> -98 -> -33

36 -> -20 -> -98 -> -35

36 -> -20 -> -98 -> -36

36 -> -20 -> -98 -> -39

36 -> -20 -> -98 -> -42

36 -> -20 -> -98 -> -43

36 -> -20 -> -98 -> -44

36 -> -20 -> -98 -> -45

36 -> -20 -> -98 -> -46

36 -> -20 -> -98 -> -47

36 -> -20 -> -98 -> -48

36 -> -20 -> -98 -> -49

36 -> -20 -> -98 -> -51

36 -> -20 -> -98 -> -55

36 -> -20 -> -98 -> -56

36 -> -20 -> -98 -> -58

36 -> -20 -> -98 -> -60

36 -> -20 -> -98 -> -61

36 -> -20 -> -98 -> -62

36 -> -20 -> -98 -> -63

36 -> -20 -> -98 -> -64

36 -> -20 -> -98 -> -66

36 -> -20 -> -98 -> -68

36 -> -20 -> -98 -> -69

36 -> -20 -> -98 -> -71

36 -> -20 -> -98 -> -73

36 -> -20 -> -98 -> -77

36 -> -20 -> -98 -> -78

36 -> -20 -> -98 -> -81

36 -> -20 -> -98 -> -83

36 -> -20 -> -98 -> -84

36 -> -20 -> -98 -> -86

36 -> -20 -> -98 -> -88

36 -> -20 -> -98 -> -89

36 -> -20 -> -98 -> -91

36 -> -20 -> -98 -> -92

36 -> -20 -> -98 -> -95

36 -> -20 -> -98 -> -96

36 -> -20 -> -98 -> -99

36 -> -20 -> -98 -> -100

36 -> -20 -> -98 -> -101

36 -> -20 -> -98 -> -105

36 -> -20 -> -98 -> -106

36 -> -20 -> -98 -> -107

36 -> -20 -> -98 -> -110

36 -> -20 -> -98 -> -113

36 -> -20 -> -98 -> -114

36 -> -20 -> -98 -> -117

36 -> -20 -> -98 -> -118

36 -> -20 -> -98 -> -119

36 -> -20 -> -98 -> -120

36 -> -20 -> -98 -> -121

36 -> -20 -> -98 -> -122

36 -> -20 -> -98 -> -124

36 -> -20 -> -98 -> -127

36 -> -20 -> -98 -> -130

36 -> -20 -> -98 -> -131

36 -> -20 -> -98 -> -132

36 -> -20 -> -98 -> -133

36 -> -20 -> -98 -> -135

36 -> -20 -> -98 -> -136

36 -> -20 -> -98 -> -137

36 -> -20 -> -98 -> -141

36 -> -20 -> -98 -> -144

36 -> -20 -> -98 -> -145

36 -> -20 -> -98 -> -146

36 -> -20 -> -98 -> -147

36 -> -20 -> -98 -> -150

36 -> -20 -> -98 -> -154

36 -> -20 -> -98 -> -155

36 -> -20 -> -98 -> -156

36 -> -20 -> -99

36 -> -20 -> -99 -> -1

36 -> -20 -> -99 -> -3

36 -> -20 -> -99 -> -6

36 -> -20 -> -99 -> -17

36 -> -20 -> -99 -> -18

36 -> -20 -> -99 -> -19

36 -> -20 -> -99 -> -22

36 -> -20 -> -99 -> -26

36 -> -20 -> -99 -> -27

36 -> -20 -> -99 -> -28

36 -> -20 -> -99 -> -29

36 -> -20 -> -99 -> -30

36 -> -20 -> -99 -> -32

36 -> -20 -> -99 -> -35

36 -> -20 -> -99 -> -36

36 -> -20 -> -99 -> -39

36 -> -20 -> -99 -> -41

36 -> -20 -> -99 -> -43

36 -> -20 -> -99 -> -48

36 -> -20 -> -99 -> -51

36 -> -20 -> -99 -> -56

36 -> -20 -> -99 -> -61

36 -> -20 -> -99 -> -62

36 -> -20 -> -99 -> -63

36 -> -20 -> -99 -> -64

36 -> -20 -> -99 -> -68

36 -> -20 -> -99 -> -73

36 -> -20 -> -99 -> -78

36 -> -20 -> -99 -> -81

36 -> -20 -> -99 -> -83

36 -> -20 -> -99 -> -86

36 -> -20 -> -99 -> -88

36 -> -20 -> -99 -> -89

36 -> -20 -> -99 -> -92

36 -> -20 -> -99 -> -95

36 -> -20 -> -99 -> -97

36 -> -20 -> -99 -> -98

36 -> -20 -> -99 -> -100

36 -> -20 -> -99 -> -101

36 -> -20 -> -99 -> -106

36 -> -20 -> -99 -> -107

36 -> -20 -> -99 -> -109

36 -> -20 -> -99 -> -111

36 -> -20 -> -99 -> -114

36 -> -20 -> -99 -> -117

36 -> -20 -> -99 -> -118

36 -> -20 -> -99 -> -119

36 -> -20 -> -99 -> -121

36 -> -20 -> -99 -> -122

36 -> -20 -> -99 -> -124

36 -> -20 -> -99 -> -127

36 -> -20 -> -99 -> -133

36 -> -20 -> -99 -> -137

36 -> -20 -> -99 -> -144

36 -> -20 -> -99 -> -145

36 -> -20 -> -99 -> -146

36 -> -20 -> -99 -> -147

36 -> -20 -> -99 -> -150

36 -> -20 -> -99 -> -151

36 -> -20 -> -99 -> -154

36 -> -20 -> -99 -> -155

36 -> -20 -> -99 -> -156

36 -> -20 -> -101

36 -> -20 -> -101 -> -1

36 -> -20 -> -101 -> -3

36 -> -20 -> -101 -> -6

36 -> -20 -> -101 -> -17

36 -> -20 -> -101 -> -19

36 -> -20 -> -101 -> -22

36 -> -20 -> -101 -> -26

36 -> -20 -> -101 -> -28

36 -> -20 -> -101 -> -29

36 -> -20 -> -101 -> -30

36 -> -20 -> -101 -> -32

36 -> -20 -> -101 -> -35

36 -> -20 -> -101 -> -36

36 -> -20 -> -101 -> -39

36 -> -20 -> -101 -> -41

36 -> -20 -> -101 -> -43

36 -> -20 -> -101 -> -48

36 -> -20 -> -101 -> -51

36 -> -20 -> -101 -> -56

36 -> -20 -> -101 -> -61

36 -> -20 -> -101 -> -62

36 -> -20 -> -101 -> -63

36 -> -20 -> -101 -> -64

36 -> -20 -> -101 -> -68

36 -> -20 -> -101 -> -73

36 -> -20 -> -101 -> -74

36 -> -20 -> -101 -> -75

36 -> -20 -> -101 -> -78

36 -> -20 -> -101 -> -81

36 -> -20 -> -101 -> -83

36 -> -20 -> -101 -> -86

36 -> -20 -> -101 -> -89

36 -> -20 -> -101 -> -92

36 -> -20 -> -101 -> -95

36 -> -20 -> -101 -> -98

36 -> -20 -> -101 -> -99

36 -> -20 -> -101 -> -100

36 -> -20 -> -101 -> -106

36 -> -20 -> -101 -> -107

36 -> -20 -> -101 -> -109

36 -> -20 -> -101 -> -111

36 -> -20 -> -101 -> -114

36 -> -20 -> -101 -> -117

36 -> -20 -> -101 -> -118

36 -> -20 -> -101 -> -119

36 -> -20 -> -101 -> -121

36 -> -20 -> -101 -> -122

36 -> -20 -> -101 -> -124

36 -> -20 -> -101 -> -125

36 -> -20 -> -101 -> -127

36 -> -20 -> -101 -> -133

36 -> -20 -> -101 -> -137

36 -> -20 -> -101 -> -144

36 -> -20 -> -101 -> -145

36 -> -20 -> -101 -> -146

36 -> -20 -> -101 -> -147

36 -> -20 -> -101 -> -150

36 -> -20 -> -101 -> -151

36 -> -20 -> -101 -> -154

36 -> -20 -> -101 -> -155

36 -> -20 -> -101 -> -156

36 -> -20 -> -106

36 -> -20 -> -106 -> -1

36 -> -20 -> -106 -> -3

36 -> -20 -> -106 -> -6

36 -> -20 -> -106 -> -18

36 -> -20 -> -106 -> -19

36 -> -20 -> -106 -> -22

36 -> -20 -> -106 -> -27

36 -> -20 -> -106 -> -29

36 -> -20 -> -106 -> -30

36 -> -20 -> -106 -> -32

36 -> -20 -> -106 -> -35

36 -> -20 -> -106 -> -36

36 -> -20 -> -106 -> -39

36 -> -20 -> -106 -> -42

36 -> -20 -> -106 -> -48

36 -> -20 -> -106 -> -51

36 -> -20 -> -106 -> -55

36 -> -20 -> -106 -> -56

36 -> -20 -> -106 -> -62

36 -> -20 -> -106 -> -63

36 -> -20 -> -106 -> -64

36 -> -20 -> -106 -> -68

36 -> -20 -> -106 -> -73

36 -> -20 -> -106 -> -78

36 -> -20 -> -106 -> -81

36 -> -20 -> -106 -> -83

36 -> -20 -> -106 -> -86

36 -> -20 -> -106 -> -88

36 -> -20 -> -106 -> -89

36 -> -20 -> -106 -> -95

36 -> -20 -> -106 -> -97

36 -> -20 -> -106 -> -98

36 -> -20 -> -106 -> -99

36 -> -20 -> -106 -> -100

36 -> -20 -> -106 -> -101

36 -> -20 -> -106 -> -105

36 -> -20 -> -106 -> -107

36 -> -20 -> -106 -> -114

36 -> -20 -> -106 -> -117

36 -> -20 -> -106 -> -124

36 -> -20 -> -106 -> -127

36 -> -20 -> -106 -> -133

36 -> -20 -> -106 -> -134

36 -> -20 -> -106 -> -137

36 -> -20 -> -106 -> -142

36 -> -20 -> -106 -> -145

36 -> -20 -> -106 -> -146

36 -> -20 -> -106 -> -147

36 -> -20 -> -106 -> -150

36 -> -20 -> -106 -> -154

36 -> -20 -> -106 -> -155

36 -> -20 -> -106 -> -156

36 -> -20 -> -107

36 -> -20 -> -107 -> -1

36 -> -20 -> -107 -> -3

36 -> -20 -> -107 -> -4

36 -> -20 -> -107 -> -5

36 -> -20 -> -107 -> -6

36 -> -20 -> -107 -> -7

36 -> -20 -> -107 -> -8

36 -> -20 -> -107 -> -9

36 -> -20 -> -107 -> -10

36 -> -20 -> -107 -> -17

36 -> -20 -> -107 -> -18

36 -> -20 -> -107 -> -19

36 -> -20 -> -107 -> -22

36 -> -20 -> -107 -> -24

36 -> -20 -> -107 -> -26

36 -> -20 -> -107 -> -27

36 -> -20 -> -107 -> -28

36 -> -20 -> -107 -> -29

36 -> -20 -> -107 -> -30

36 -> -20 -> -107 -> -31

36 -> -20 -> -107 -> -32

36 -> -20 -> -107 -> -33

36 -> -20 -> -107 -> -35

36 -> -20 -> -107 -> -36

36 -> -20 -> -107 -> -39

36 -> -20 -> -107 -> -42

36 -> -20 -> -107 -> -43

36 -> -20 -> -107 -> -44

36 -> -20 -> -107 -> -45

36 -> -20 -> -107 -> -46

36 -> -20 -> -107 -> -47

36 -> -20 -> -107 -> -48

36 -> -20 -> -107 -> -49

36 -> -20 -> -107 -> -55

36 -> -20 -> -107 -> -56

36 -> -20 -> -107 -> -58

36 -> -20 -> -107 -> -60

36 -> -20 -> -107 -> -61

36 -> -20 -> -107 -> -62

36 -> -20 -> -107 -> -63

36 -> -20 -> -107 -> -64

36 -> -20 -> -107 -> -66

36 -> -20 -> -107 -> -68

36 -> -20 -> -107 -> -69

36 -> -20 -> -107 -> -71

36 -> -20 -> -107 -> -73

36 -> -20 -> -107 -> -77

36 -> -20 -> -107 -> -78

36 -> -20 -> -107 -> -81

36 -> -20 -> -107 -> -83

36 -> -20 -> -107 -> -84

36 -> -20 -> -107 -> -86

36 -> -20 -> -107 -> -88

36 -> -20 -> -107 -> -89

36 -> -20 -> -107 -> -91

36 -> -20 -> -107 -> -92

36 -> -20 -> -107 -> -93

36 -> -20 -> -107 -> -95

36 -> -20 -> -107 -> -96

36 -> -20 -> -107 -> -97

36 -> -20 -> -107 -> -98

36 -> -20 -> -107 -> -99

36 -> -20 -> -107 -> -101

36 -> -20 -> -107 -> -105

36 -> -20 -> -107 -> -106

36 -> -20 -> -107 -> -110

36 -> -20 -> -107 -> -113

36 -> -20 -> -107 -> -114

36 -> -20 -> -107 -> -116

36 -> -20 -> -107 -> -117

36 -> -20 -> -107 -> -118

36 -> -20 -> -107 -> -119

36 -> -20 -> -107 -> -120

36 -> -20 -> -107 -> -121

36 -> -20 -> -107 -> -122

36 -> -20 -> -107 -> -124

36 -> -20 -> -107 -> -130

36 -> -20 -> -107 -> -131

36 -> -20 -> -107 -> -132

36 -> -20 -> -107 -> -133

36 -> -20 -> -107 -> -135

36 -> -20 -> -107 -> -136

36 -> -20 -> -107 -> -137

36 -> -20 -> -107 -> -141

36 -> -20 -> -107 -> -144

36 -> -20 -> -107 -> -145

36 -> -20 -> -107 -> -146

36 -> -20 -> -107 -> -147

36 -> -20 -> -107 -> -150

36 -> -20 -> -107 -> -154

36 -> -20 -> -107 -> -155

36 -> -20 -> -107 -> -156

36 -> -20 -> -114

36 -> -20 -> -114 -> -1

36 -> -20 -> -114 -> -3

36 -> -20 -> -114 -> -6

36 -> -20 -> -114 -> -7

36 -> -20 -> -114 -> -19

36 -> -20 -> -114 -> -29

36 -> -20 -> -114 -> -30

36 -> -20 -> -114 -> -35

36 -> -20 -> -114 -> -39

36 -> -20 -> -114 -> -48

36 -> -20 -> -114 -> -51

36 -> -20 -> -114 -> -62

36 -> -20 -> -114 -> -63

36 -> -20 -> -114 -> -64

36 -> -20 -> -114 -> -73

36 -> -20 -> -114 -> -78

36 -> -20 -> -114 -> -81

36 -> -20 -> -114 -> -83

36 -> -20 -> -114 -> -86

36 -> -20 -> -114 -> -89

36 -> -20 -> -114 -> -95

36 -> -20 -> -114 -> -98

36 -> -20 -> -114 -> -99

36 -> -20 -> -114 -> -100

36 -> -20 -> -114 -> -101

36 -> -20 -> -114 -> -106

36 -> -20 -> -114 -> -107

36 -> -20 -> -114 -> -110

36 -> -20 -> -114 -> -117

36 -> -20 -> -114 -> -124

36 -> -20 -> -114 -> -127

36 -> -20 -> -114 -> -130

36 -> -20 -> -114 -> -133

36 -> -20 -> -114 -> -137

36 -> -20 -> -114 -> -146

36 -> -20 -> -114 -> -150

36 -> -20 -> -114 -> -154

36 -> -20 -> -114 -> -155

36 -> -20 -> -114 -> -156

36 -> -20 -> -117

36 -> -20 -> -117 -> -1

36 -> -20 -> -117 -> -3

36 -> -20 -> -117 -> -4

36 -> -20 -> -117 -> -6

36 -> -20 -> -117 -> -7

36 -> -20 -> -117 -> -8

36 -> -20 -> -117 -> -9

36 -> -20 -> -117 -> -10

36 -> -20 -> -117 -> -17

36 -> -20 -> -117 -> -18

36 -> -20 -> -117 -> -19

36 -> -20 -> -117 -> -22

36 -> -20 -> -117 -> -24

36 -> -20 -> -117 -> -26

36 -> -20 -> -117 -> -27

36 -> -20 -> -117 -> -28

36 -> -20 -> -117 -> -29

36 -> -20 -> -117 -> -30

36 -> -20 -> -117 -> -31

36 -> -20 -> -117 -> -32

36 -> -20 -> -117 -> -33

36 -> -20 -> -117 -> -35

36 -> -20 -> -117 -> -36

36 -> -20 -> -117 -> -39

36 -> -20 -> -117 -> -42

36 -> -20 -> -117 -> -43

36 -> -20 -> -117 -> -44

36 -> -20 -> -117 -> -45

36 -> -20 -> -117 -> -46

36 -> -20 -> -117 -> -47

36 -> -20 -> -117 -> -48

36 -> -20 -> -117 -> -49

36 -> -20 -> -117 -> -51

36 -> -20 -> -117 -> -55

36 -> -20 -> -117 -> -56

36 -> -20 -> -117 -> -58

36 -> -20 -> -117 -> -60

36 -> -20 -> -117 -> -61

36 -> -20 -> -117 -> -62

36 -> -20 -> -117 -> -63

36 -> -20 -> -117 -> -64

36 -> -20 -> -117 -> -66

36 -> -20 -> -117 -> -68

36 -> -20 -> -117 -> -69

36 -> -20 -> -117 -> -73
[truncated: 239,944 more chars]
