# Supplementary material for: BPLLDA: Predicting lncRNA-Disease Associations Based on Simple Paths With Limited Lengths in a Heterogeneous Network
Source: Front Genet. 2018 Oct 16;9:411. doi: 10.3389/fgene.2018.00411 (PMC6232683; doi:10.3389/fgene.2018.00411)
Supplement: Supplementary file 4 [file Table_4.DOCX]

Supplementary Material

**BPLLDA:  predicting lncRNA-disease associations based on simple**

**paths with limited length on a heterogeneous network**

Xiaofang Xiao^1#^, Wen Zhu^2#^, Bo Liao^1,2*^, Junlin Xu^1^, Changlong Gu^1^, Binbin Ji^2^, Yuhua Yao^2^, Lihong Peng^3^, Jialiang Yang ^2,4*^

^1^ College of Information Science and Engineering, Hunan University, Changsha, Hunan, 410082, P.R. China

^2^ School of Mathematics and Statistics, Hainan Normal University, Haikou, 570100, P.R. China

^3^ School of Computer Science, Hunan University of Technology, Zhuzhou, Hunan, 412007, P.R. China

^4^ Icahn Institute for Genomics and Multiscale Biology, Icahn School of Medicine at Mount Sinai, New York, NY 10029, USA

**^#^** The authors contributed equally to this study

*** Correspondence:**Jialiang Yang [jialiang.yang@mssm.edu](mailto:jialiang.yang@mssm.edu)

Bo Liao [dragonbw@163.com](mailto:dragonbw@163.com)

# Supplementary Data

**Supplementary Table 1.** The path of cervical cancer. (DOCX)

**Supplementary Table 2.** Names of diseases involved in known lncRNA-disease associations. (XLSX)

**Supplementary Table 3.** Names of lncRNAs involved in known lncRNA-disease associations. (XLSX)

# 2 Supplementary Figures


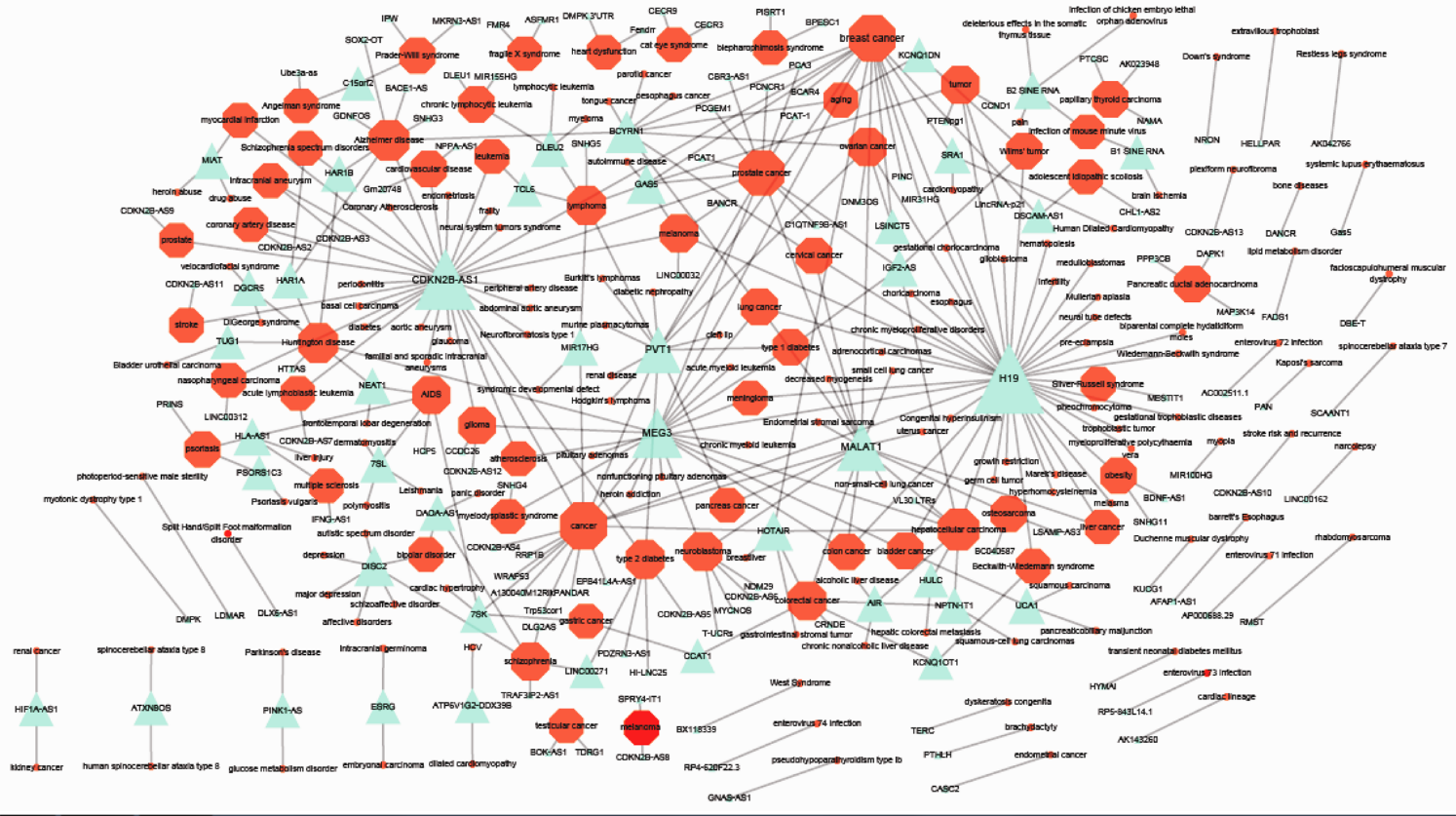


**Supplementary Figure 1.** The lncRNA-disease associations network, based on 352 confirmed lncRNA-disease associations. Circle nodes represent diseases and triangle nodes represent lncRNAs. Edges between lncRNAs and diseases represent known association.
