# Supplementary figures and images for: BPLLDA: Predicting lncRNA-Disease Associations Based on Simple Paths With Limited Lengths in a Heterogeneous Network
Source: Front Genet. 2018 Oct 16;9:411. doi: 10.3389/fgene.2018.00411 (PMC6232683; doi:10.3389/fgene.2018.00411)

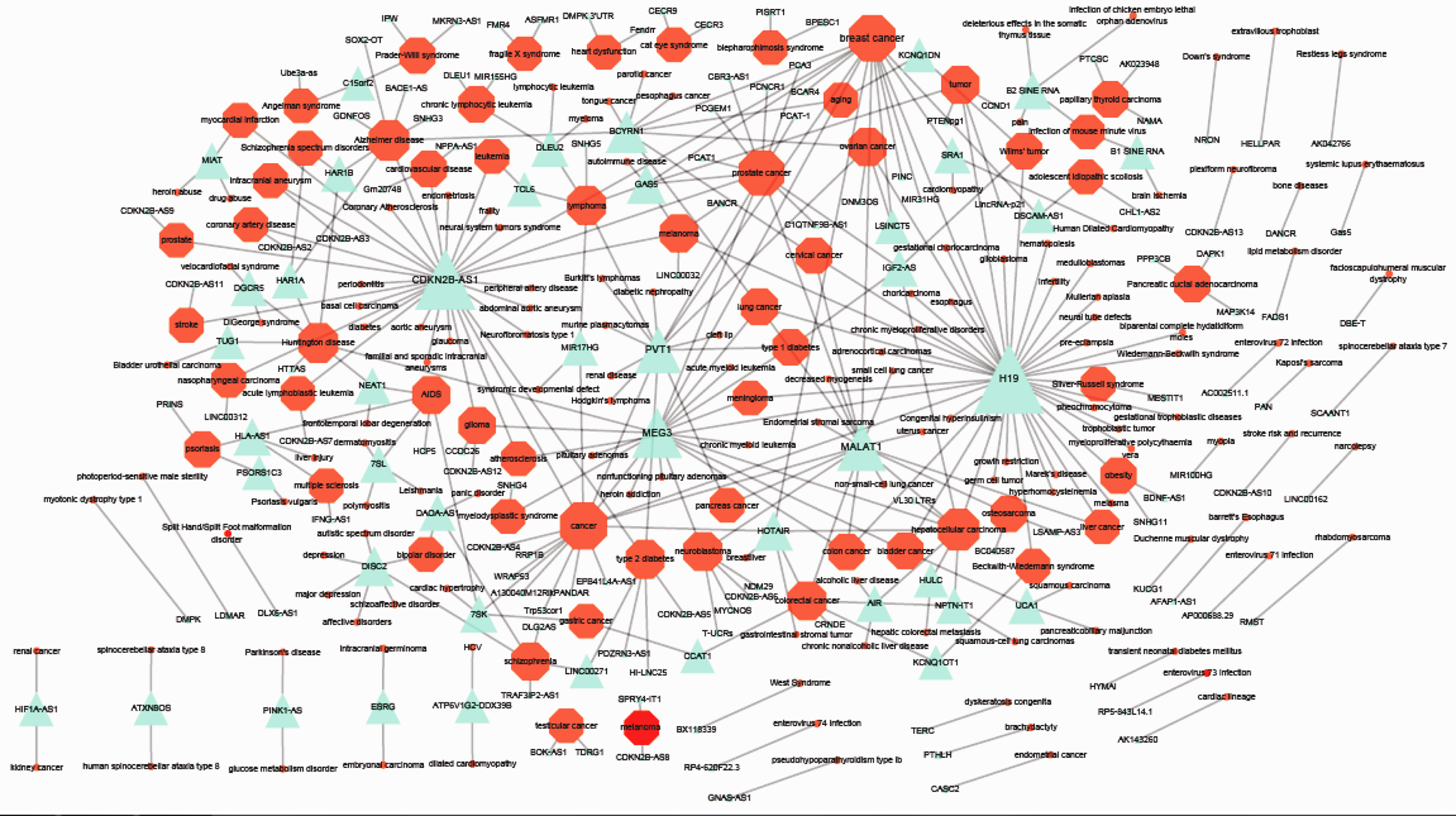

Supplement: Supplementary file 5 [file Image_1.TIF]
